# A comparative genome analysis of the *Bacillota* (*Firmicutes*) class *Dehalobacteriia*

**DOI:** 10.1099/mgen.0.001039

**Published:** 2023-06-09

**Authors:** Young C. Song, Sophie I. Holland, Matthew Lee, Gao Chen, Frank E. Löffler, Michael J. Manefield, Philip Hugenholtz, Ulrike Kappler

**Affiliations:** ^1^​ The University of Queensland, School of Chemistry and Molecular Biosciences, Australian Centre for Ecogenomics, St Lucia, Queensland, 4072, Australia; ^2^​ School of Civil and Environmental Engineering, UNSW Sydney, Sydney, NSW, 2052, Australia; ^3^​ Center for Environmental Biotechnology, Department of Civil and Environmental Engineering, University of Tennessee, Knoxville, Tennessee, USA; ^4^​ Department of Microbiology, Department of Bioengineering and Soil Science, University of Tennessee, Knoxville, Tennessee, USA; ^5^​ Oak Ridge National Laboratory, Biosciences Division, Oak Ridge, Tennessee, USA; ^6^​ The University of Queensland, School of Chemistry and Molecular Biosciences, St Lucia, Queensland, 4072, Australia; ^†^​ Present address: School of Engineering & Physical Sciences, Heriot-Watt University, Edinburgh, UK

**Keywords:** *Dehalobacteriia*, comparative genomics, metabolic reconstruction, dichloromethane degradation, proteome

## Abstract

*Dehalobacterium formicoaceticum* is recognized for its ability to anaerobically ferment dichloromethane (DCM), and a catabolic model has recently been proposed. *D. formicoaceticum* is currently the only axenic representative of its class, the *Dehalobacteriia*, according to the Genome Taxonomy Database. However, substantial additional diversity has been revealed in this lineage through culture-independent exploration of anoxic habitats. Here we performed a comparative analysis of 10 members of the *Dehalobacteriia,* representing three orders, and infer that anaerobic DCM degradation appears to be a recently acquired trait only present in some members of the order *Dehalobacteriales*. Inferred traits common to the class include the use of amino acids as carbon and energy sources for growth, energy generation via a remarkable range of putative electron-bifurcating protein complexes and the presence of S-layers. The ability of *D. formicoaceticum* to grow on serine without DCM was experimentally confirmed and a high abundance of the electron-bifurcating protein complexes and S-layer proteins was noted when this organism was grown on DCM. We suggest that members of the *Dehalobacteriia* are low-abundance fermentative scavengers in anoxic habitats.

## Data Summary

The authors confirm all supporting data and protocols have been provided within the article or through Supplementary Data files. Raw peptide data have been deposited in the PRIDE database [1] under accession PXD038410.

**Impact Statement**

Evolutionary and ecological context is important to understand model microorganisms. Here we show that anaerobic dichloromethane degradation, a key metabolic trait of *Dehalobacterium formicoaceticum*, is a relatively recent acquisition and not representative of the lineage to which this bacterium belongs. By contrast, the potential to use amino acids as carbon and energy sources is widespread in the class *Dehalobacteriia*, a trait we were able to experimentally confirm in *D. formicoaceticum*. We suggest that *Dehalobacteriia* are primarily low-abundance fermentative scavengers in anoxic ecosystems.

## Introduction

Metagenomics and, more recently, metagenome-assembled genomes (MAGs) are revealing the scope and functional potential of the microbial world [2–4]. This is true even for apparently well-studied lineages such as the phylum *Bacillota* (formerly *Firmicutes *). According to the Genome Taxonomy Database (GTDB), a rank-normalized genome-based taxonomy, the *Bacillota* comprises several class-level lineages that are exclusively or predominantly represented by MAGs [2]. A case in point is the class *Dehalobacteriia* that is currently represented by a single axenic culture, *Dehalobacterium formicoaceticum*, so called due to its unusual ability to anaerobically ferment dichloromethane (DCM) to formate and acetate. Note that *D. formicoaceticum* is classified by LPSN [5] as a member of the family *Peptococcaceae *, which encompasses 25 validly published genera with cultured representatives (https://lpsn.dsmz.de/family/peptococcaceae). However, these genera are phylogenetically diverse and belong to multiple classes according to the rank-normalized GTDB taxonomy [2].

DCM is a chlorinated C1 compound that is widely used as an industrial solvent, resulting in substantial groundwater contamination [6, 7]. It is also produced naturally in environments such as oceans, wetlands and soils and contributes to climate warming [6, 7]. While the phylogenetic distribution and mechanism of aerobic DCM oxidation are well established [8, 9], the same cannot be said for anaerobic DCM metabolism. To date, three genera of anaerobic bacteria are known to degrade DCM: *D. formicoaceticum* [10], ‘*Ca*. Forminomas warabiya’ [11, 12] and ‘*Ca*. Dichloromethanomonas elyunquensis’ [13]. Both *D. formicoaceticum* and ‘*Ca*. F. warabiya’ are members of the *Dehalobacteriia*, while ‘*Ca*. D. elyunquensis’ belongs to a neighbouring *Bacillota* class, the *Desulfitobacteriia*, as defined by GTDB. Recently, a model has been proposed for anaerobic DCM degradation that is based on proteomic and genomic analyses. In this model, the methylene chloride catabolism (Mec) proteins, which include several corrinoid-dependent methyltransferases, convert DCM into Wood–Ljungdahl pathway (WLP) intermediates, such as methylene- or methyl-tetrahydrofolate (THF) [14]. The final metabolic products of anaerobic DCM degradation include formate, acetate and inorganic chloride, as previously described [5–7, 10–14].

It is not known whether anaerobic DCM degradation is common to all members of the *Dehalobacteriia*, and the broader metabolic properties of this class have not been investigated. Here, we performed a phylogenetic and metabolic reconstruction of publicly available *Dehalobacteriia* genomes representing three orders within this class. Anaerobic DCM degradation may be a recently acquired trait only found in some members of the order *Dehalobacteriales*. By contrast, all members of the *Dehalobacteriia* appear to rely on a combination of a glycine cleavage system and the WLP for carbon fixation and energy generation. A number of potential electron-bifurcating complexes were highly conserved and highly expressed in *D. formicoaceticum* growing on DCM, suggesting their metabolic importance. While *D. formicoaceticum* is known as an obligate DCM degrader, we demonstrated its ability to grow on serine in the absence of DCM, an ability that appears to be conserved within the class. The distribution of *Dehalobacteriia* 16S rRNA genes in the environment suggests that members of this lineage are found in anoxic habitats in low abundance, which combined with the metabolic information suggest that *Dehalobacteriia* are fermentative scavengers.

## Methods

### Data retrieval

According to GTDB release 06-RS202 (April 2021), there are 17 species representative genomes in the class *Dehalobacteriia*, with an estimated average completeness of 81.8 % and contamination of 0.5 % (Table S1, available in the online version of this article). For the purpose of this study, nine genomes with an estimated completeness greater than or equal to the average were selected for metabolic inference (Table S1). Despite having an estimated completeness lower than the average (i.e. 78.5 %), UBA4997 was also added to this study, as it is one of four genomes in *Dehalobacteriia* that possess 16S small-subunit rRNA (16S SSU rRNA), which was used for relative abundance analysis as described below. Out of these ten sequences, the genomes DUPU01 and RUG14212 were introduced into *Dehalobacteriia* in GTDB 06-RS202, while the remaining eight genomes, including those of the isolate *D. formicoaceticum* [10, 14] and mixed-culture member ‘*Ca*. F. warabiya’ [11, 12], were introduced to this class in the previous GTDB release, 04-RS89 (June 2019). For the purpose of metabolic inference, Prokka [15] was used. Detailed steps of these bioinformatics analyses are described in subsequent sections.

### Relative abundance analysis

A set of 11 near full-length 16S SSU rRNA gene sequences obtained from four *Dehalobacteriia* genomes (*D. formicoaceticum*, ‘*Ca*. F. warabiya’, UBA4997 and UBA5757) were checked against a set of commonly used broadly conserved SSU rRNA primers [16] to determine the likelihood of primer bias in rRNA-based environmental surveys. With the exception of 803F, all primers should be able to PCR-amplify *Dehalobacteriia* SSU rRNA genes (Table S2). Using nucleotide blast (blastn) [17], these sequences were then searched against the database downloaded from archives of the silva SSU rRNA gene database (release 138.1, 510 508 total sequences [18]) to conservatively (≥ 95 % identity) identify 16S rRNA gene sequences belonging to the *Dehalobacteriia* (currently not annotated in silva). An E-value of 1e-3, maximum target hits of 10 and tabular output format (i.e. *-evalue 1e-3 -outfmt 6 -max_target_seqs 10*) were used as parameters for this search operation. Full 16S rRNA gene amplicon datasets associated with the identified *Dehalobacteriia* sequences (Table S3) were obtained using their publication information where available. The sequences that were not published were excluded from subsequent analysis. The relative abundance of *Dehalobacteriia* sequences in each dataset was then determined as the percentage of total sequences in the study.

### Metabolic inference of *Dehalobacteriia*

*Dehalobacteriia* MAG or isolate genomes were annotated using Prokka with --*kingdom Bacteria* and *--addgenes* parameters, where the latter is used to add gene features (e.g. gene names, enzyme commission ID and protein name) for each detected coding region. A list of metabolic pathways inferred to be present in the *Dehalobacteriia* genomes was generated by importing the GenBank (GBK) files generated from Prokka to Pathway Tools software [19]. In addition, sequences of detected proteins were also searched against KO (KEGG Orthology [20]) using BlastKOALA (KEGG Orthology and Links Annotation [21]) to generate a list of KO identifications for each *Dehalobacteriia* genome (Table S4). To determine the presence of membrane-bound redox complexes and cytoplasmic hydrogenases, a subset of proteins identified by Prokka were further searched against the NCBI non-redundant (nr) database using protein blast (blastp). The conserved domain database (CDD [22]) associated with the top hit of each query was used to confirm the identification of protein function assignment for *Dehalobacteriia* by comparison with biochemically characterized proteins, such as *Rhodobacter* nitrogen fixation (Rnf) complex [23], *Sporomusa* type ferredoxin-dependent transhydrogenase (Stn [24]) and electron-bifurcating hydrogenase (Hnd [25]). Conservation of the hydrogenase proteins detected from the *Dehalobacteriia* genomes was visualized as a gene-neighbourhood diagram using Clinker [26].

The protein sequences detected from the *Dehalobacteriia* genomes using Prokka were also searched against a customized protein database of the *mec* gene cassette [14] to predict the functional potential of the genomes in relation to anaerobic DCM fermentation. A blastp search with the following parameters was used for this operation: an e-value of 1e-3, maximum target hits of 10 and tabular output format.

### Phylogenetic inference

*Dehalobacteriia* MAGs and isolate genomes, along with species-representative genomes in the neighbouring *Bacillota* class-level lineages, as defined in GTDB, were used for phylogenetic inference. The phylogenetic tree was built using IQ-Tree [27] with protein mixture model C60 and 1000 bootstrap cycles as parameter for the maximumum-likelihood (ML) inference.

The GenBank files generated for each of the *Dehalobacteriia* genomes using Prokka were used as input files for Clinker [26] to generate two gene-neighbourhood diagrams, one depicting the conservation of the redox complexes detected in *Dehalobacteriia* and the other showing the conservation of the *mec* cassette present in *D. formicoaceticum*, ‘*Ca*. F. warabiya’ and ‘*Ca*. Dichloromethanomonas elyunquensis’ [14]. A minimum amino acid identity of 30% was used to generate the Clinker plot [26].

### Analytical assays

#### Bacterial cultures and growth conditions

*D. formicoaceticum* (ATCC 700118) was cultivated at 30 °C in a defined, anoxic, bicarbonate-buffered mineral salt medium containing DCM as the sole energy source, as described by Chen and colleagues [28]. Medium preparation followed the methodology described by Holland and colleagues [12] with modification. In brief, 1 litre of medium contained CaCl_2_.H_2_O (0.1 g), MgCl_2_.6H_2_O (0.1 g), NH_4_Cl (1.5 g), NaH_2_PO_4_ (0.6 g), KCl (0.1 g) and 1 ml each of trace element solutions A and B (Tables S5 and S6) as described previously [29]. The medium was purged with N_2_ gas for 1 h followed by addition of 2.5 g NaHCO_3_ and sparging with N_2_/CO_2_ (80 : 20) to achieve a pH between 6.8 and 7.0. The pH-adjusted medium (50 ml) was then dispensed into nitrogen-sparged 60 ml serum bottles. The serum bottles were immediately sealed with a Teflon-coated rubber cap and metal crimp, sparged with N_2_/CO_2_ (80 : 20) for another 5 min and autoclaved for 20 min at 121 °C.

Before use, the following reagents were aseptically added to each bottle: 500 µl of 5 gl^−1^ yeast extract solution (50 mg l^−1^ final concentration) and 50 µl of 1000× vitamin solution (Table S7 [30]). DCM and/or serine were added to the standard medium as carbon sources with final concentrations of 1 and 10 mM, respectively. Serum bottles were inoculated with 2 % (v/v) *D. formicoaceticum* and incubated statically in the dark at 30°C for up to 31 days [10]. Cultures were resupplied with DCM and/or serine once, to the same concentration, when the initial substrate pulse was depleted.

#### Determination of DCM concentrations by GC

DCM was quantified on a Shimadzu GC-2010 gas chromatograph equipped with a flame ionzation detector (GC-FID) and a GS-Q column (30 m×0.32 mm; Agilent Technologies). Headspace samples (100 µl) were withdrawn directly from culture flasks using a lockable, gas-tight syringe and manually injected. The oven was initially at 150 °C, then raised by 30 °C min^−1^ to 250°C. The inlet temperature was 250 °C, split ratio 1 : 10 and FID temperature 250 °C. The carrier gas was helium (3 ml min^−1^). A minimum three-point calibration curve was used. DCM concentrations are reported as the nominal concentration in each serum bottle, calculated from the headspace concentration using Henry’s Law dimensionless solubility constant (Hcc=0.107 at 30 °C), as per the OSWER method [31].

#### Determination of volatile fatty acid concentrations by GC

The volatile fatty acids (VFAs) acetate, formate, butyrate and propionate were quantified as described by Holland *et al*. [12]. VFAs were derivatized to their ethyl esters by combining 500 µl of liquid culture sample, 200 µl of ethanol (100%), and 200 µl of 1 M H_2_SO_4_ in 10 ml glass vials, which was sealed with Teflon/butyl septa and screw caps, and then incubated at 60 °C for 45 min. Ethyl esters were separated using a Shimadzu GC-2010 GC-FID fitted with a DB-FFAP column (30 m×0.320 mm; Agilent Technologies) and a PAL LHS2-xt-Shim autosampler (Shimadzu). The samples were agitated at 80 °C for 5 min, before 250 µl of sample headspace was injected. The oven was held at a constant temperature of 40 °C for 6 min, the carrier gas was helium (2 ml min^−1^) and other parameters were as described above for DCM quantification. A six-point calibration curve was used for acetate, butyrate, propionate and formate (0, 0.5, 1, 5, 10 and 15 mM).

#### Determination of serine concentrations by GC-triple quadrupole MS (GC-TQMS)

Determination of serine in culture samples used the method of Villas-Boas and colleagues [32] with modifications. In a 1.5 ml glass vial, 50 µl of culture sample, 250 µl NaOH (1 %), 250 µl ethanol (100 %), 5 µl alanine (10 mM; internal standard) and 50 µl pyridine were combined before 20 µl ethyl chloroformate (Ecf) was added to the mixture, followed by shaking for 20 s. A second 20 µl aliquot of Ecf was added and the vial was shaken again for 20 s. DCM (1 ml) was added, and the vial was shaken. After phase separation the upper, aqueous phase was removed, and a small amount of anhydrous Na_2_SO_4_ was added to remove water. The solvent phase was transferred to a fresh vial for analysis on an Agilent 7890A gas chromatograph system fitted with a 7000A triple quadrupole mass spectrometer (GC-TQMS). The TQMS was operated in the multiple reaction monitoring (MRM) mode. Precursor–product ion transitions (*m*/*z*) and the collision energy (eV) were 116.2→72.1 (*m*/*z*), 10 (eV) for alanine and 114.0→86.0 (*m*/*z*), 10 (eV) for serine [32].

#### Preparation of protein extracts for proteomic analyses

Cultures were harvested after a 31 day incubation, when approximately 80 % of the second substrate pulse had been depleted. The culture suspension (~40 ml) was transferred to an ice-cold plastic Falcon tube and centrifuged at 3270 relative centrifugal force (rcf) for 45 min at 4 °C. The supernatant was removed, leaving 1 ml to suspend the cell pellet. The concentrated cell suspension was transferred to 1.5 ml microfuge tubes and centrifuged at 14 000 rcf for 10 min at 4 °C. After removing the supernatant, the pellets were suspended in 100 µl of SDS-MOPS protein extraction buffer [50 mM MOPS, pH 7.9; 150 mM NaCl; 100 µM EDTA; 100 µM MgCl_2_; 4 % (w/v) SDS]. The mixture was transferred to a 2 ml bead-beating tube that contained 0.06 g glass beads (0.5 mm) and a 6.35 mm ceramic sphere. The samples were processed for 5 min at 1 800 r.p.m. in a PowerLyser (Qiagen), and followed by centrifugation at 16 000 rcf for 10 min at 4 °C. Supernatants were transferred to clean 1.5 ml tubes and again centrifuged at 14 000 rcf for 10 min at 4 °C. This supernatant – the final crude extract – was transferred to a 1.5 ml tube and stored at −20 °C until further analysis. Protein quantification was performed using the MicroBCA (ThermoFisherTM) assay as per the manufacturer’s instructions. Typical protein concentrations of cell extracts were between 0.6 and 1.7 µg ml^−1^. Tryptic peptide samples were prepared using a modified version of a filter-assisted sample preparation (FASP) protocol [33]. Detailed steps of the FASP protocol are available in the Supplementary Information.

#### Liquid chromatography

Peptides were separated by nanoLC using an UltiMate nanoRSLC UPLC and autosampler system (Dionex). Samples (2.5 µl) were concentrated and desalted on a micro C18 precolumn (300 µm×5 mm; Dionex) with H_2_O/CH_3_CN (98 : 2, 0.2 % trifluoroacetic acid) at 15 µl min^−1^. After a 4 min wash, the pre-column was switched (Valco 10 port UPLC valve; Valco) into line with a fritless nano column (75 µm×~15 cm) containing C18AQ media (1.9μ, 120 Å; Dr Maisch). Peptides were eluted using a linear gradient of H_2_O/CH_3_CN (98 : 2, 0.1 % formic acid) to H_2_O/CH_3_CN (64 : 36, 0.1 % formic acid) at 200 nl min^−1^ over 30 min. High voltage (2000 V) was applied to a low-volume titanium union (Valco) and the tip was positioned ~0.5 cm from the heated capillary (*T*=275 °C) of an Orbitrap Fusion Lumos (Thermo Electron Bremen, Germany) mass spectrometer. Positive ions were generated by electrospray and the Fusion Lumos was operated in data-dependent acquisition mode (DDA).

A survey scan of *m*/*z* 350–1750 was acquired (resolution=120 000 at *m*/*z* 200, with an accumulation target value of 400 000 ions) and lockmass-enabled (*m*/*z* 445.12003). Data-dependent tandem MS analysis was performed using a top-speed approach (cycle time of 2 s). MS2 spectra were fragmented by high-energy collision dissociation (HCD; NCE=30) activation mode and the ion-trap was selected as the mass analyser. The intensity threshold for fragmentation was set to 25 000 units. A dynamic exclusion of 20 s was applied with a mass tolerance of 10 p.p.m.

#### Protein identification

The peptide sequence data obtained from the three replicates of the *D. formicoaceticum* sample grown in DCM-containing medium were mapped against the ORFs of the *D. formicoaceticum* genome available in GTDB release R06-202, using ProteinPilot software (version 5.0.2; AB Sciex, 2018). The ProteinPilot results were filtered using a custom R script (R version 3.4.3 [34]) to generate a list of proteins which were identified in at least two replicates of each test set and had an average ‘unused’ peptide score ≥2 (i.e. 99 % confidence of identification).

## Results and discussion

### Environmental distribution of *Dehalobacteriia*

**Fig. 1.**
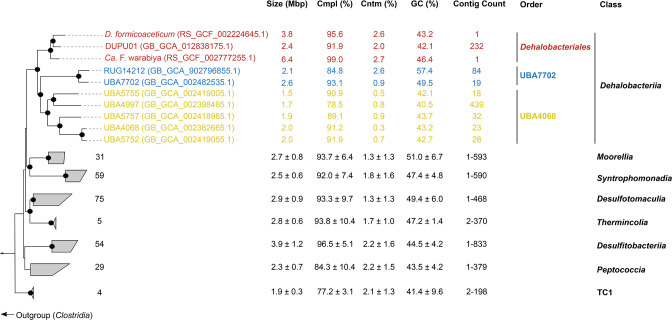
Phylogenetic relationships between members of the *Dehalobacteriia* and neighbouring *Bacillota* classes as defined by the GTDB. The maximum-likelihood (ML) tree was inferred based on alignment of 120 concatenated marker genes downloaded from GTDB R06-R202. IQ-Tree with protein mixture model C60 and 1000 bootstrap cycles were used as parameters for ML inference. Bootstrap values indicated by black dots on each node of the tree are all ≥90 %. Also shown are the genome size (Mb), estimated genome completion (%), estimated genome contamination (%), GC content (%) and contig count in respective order. The number of representatives in each neighbouring class is shown beside each grey wedge.

As of GTDB release 06-RS202, three orders are recognized within the class *Dehalobacteriia*: *Dehalobacteriales*, UBA7702 and UBA4068 (Fig. 1). The only characterized members of this class belong to the order *Dehalobacteriales*: *D. formicoaceticum* and ‘*Ca*. F. warabiya’ (Fig. 1; Table S1). Analysis of metadata associated with *Dehalobacteriia* genomes suggests that they are limited to anoxic habitats consistent with the strictly anaerobic lifestyle of the characterized representatives of this class and its neighbouring GTDB-defined classes *Desulfitobacteriia*, *Moorellia*, *Peptococcia*, *Syntrophomonadia* and *Desulfotomaculia* [35–38]. Documented habitats include an Alberta oil sands tailing pond [39], the bovine rumen [40], anaerobic digester sludge [41], an alcohol fermentation pit [42] and a methanol-fed electric cell anode biofilm [43]. To further evaluate the ecological distribution of this group, we explored 16S rRNA gene amplicon data present in the silva database. However, silva does not currently include *Dehalobacteriia* in its classification scheme. Therefore, we identified 16S rRNA gene sequences putatively belonging to this class by similarity searches using all available 16S rRNA gene sequences derived from genomes classified as *Dehalobacteriia* in GTDB (06-RS202). Only 46 of the 510 508 reference sequences in silva (v138) had ≥95 % similarity to GTDB *Dehalobacteriia* 16S rRNA sequences, all of which were associated with anoxic or low-oxygen habitats, including the murine caecum [44–46], the human digestive tract [47], the insect gut [48], a hydrocarbon-contaminated aquifer [49] and a mud volcano [50]. The predominant inferred metabolic activity of the microbial communities occurring in these habitats is fermentation of metabolites such as VFAs (e.g. propionate, butyrate) and amino acids producing acetate, ethanol, formate, CO_2_ or H_2_ as end products [51, 52]. In each of these environmental surveys, the *Dehalobacteriia* sequences typically comprised «1 % of the total 16S rRNA gene sequences obtained (Table S3), suggesting that this class is not only restricted to anoxic habitats, but also has relatively low environmental abundance.

### Anaerobic dichloromethane degradation

**Fig. 2.**
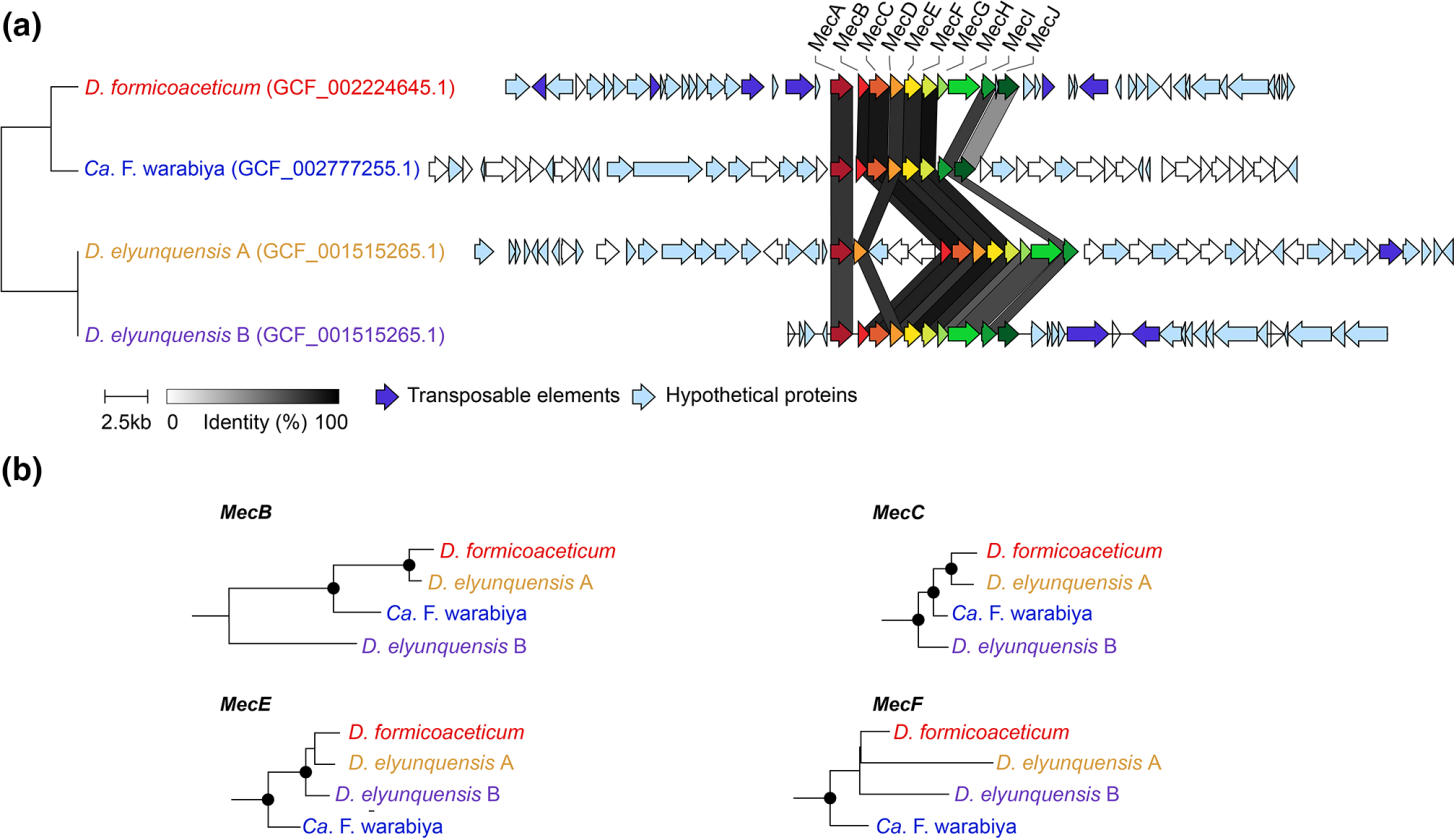
**(a**) Gene neighbourhoods of the methylene chloride catabolism (*mec*) cassette and (b) phylogenetic relationships of selected *mec* genes in the *Dehalobacteriia* (*Dehalobacterium formicoaceticum* and ‘*Ca*. Formimonas warabiya’) and *Desulfitobacteriia* (‘*Ca*. Dichloromethanomonas elyunquensis’). Filled circles indicate >90 % bootstrap support.

Current knowledge of *Dehalobacteriia* metabolism comes from the only pure culture representative, *D. formicoaceticum,* and a highly enriched culture dominated by ‘*Ca*. F. warabiya’. Both bacteria are notable for their ability to anaerobically degrade DCM. Recently, a pathway for anaerobic DCM degradation has been proposed based on comparative genomics and shotgun proteomics that identified a cassette comprising 8–10 genes called the methylene chloride catabolism (*mec*) cassette [14]. This cassette is present in two of the three members of the order *Dehalobacteriales* (*D. formicoaceticum* and ‘*Ca*. F warabiya’; Fig. 2), and in *‘Ca*. Dichloromethanomonas elyunquensis’, a member of the neighbouring GTDB-defined class *Desulfitobacteriia*. None of the *Dehalobacteriia* MAGs included in this study were found to harbour close homologues of the *mec* cassette (>30 % amino acid identity), suggesting that anaerobic DCM degradation is not ancestral to the *Dehalobacteriia*. Twenty-nine additional genomes classified as *Dehalobacteriia* in the latest GTDB release (08-RS214), not included in the present analysis, also lack close homologues of the *mec* cassette (data not shown). Phylogenetic and gene neighbourhood analysis of the *mec* cassette in *D. formicoaceticum,* ‘*Ca*. F. warabiya’ and ‘*Ca*. D. elyunquensis’ support this inference as there is no conservation of flanking regions and individual *mec* genes have different evolutionary histories (Fig. 2b) suggesting that the *mec* cassettes were present in the *Dehalobacteriia* no earlier than the common ancestor of the order *Dehalobacteriales* and possibly acquired later than that. Furthermore, the flanking regions include transposable elements and many small hypothetical genes, suggesting that the *mec* cassettes are located in mobile accessory elements (Fig. 2a). The absence of an *mec* cassette in DUPU01, the third member of the order *Dehalobacteriales*, may therefore be correct although it is also possible that it was missed as the genome is only in draft (232 contigs, 92 % estimated completeness; Fig. 1). Anaerobic DCM degradation via the *mec* cassette is predicted to produce methylene-THF, which is an intermediate of the WLP and connects DCM degradation directly to a dominant carbon turnover pathway in these bacteria.

### C1 metabolism in *Dehalobacteriia* mostly relies on an incomplete WLP and the glycine synthase complex

**Fig. 3.**
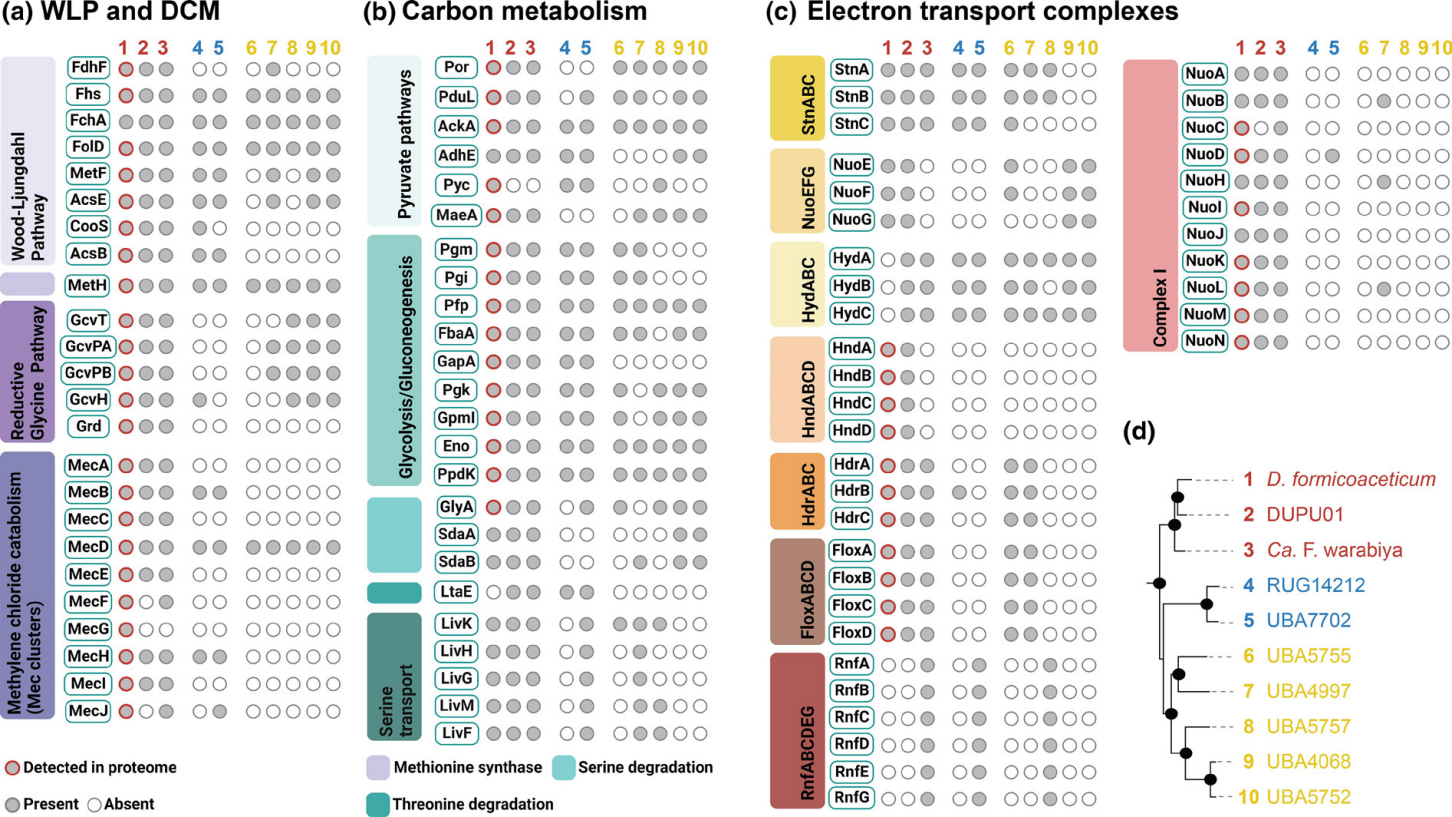
Distribution of key inferred metabolic traits in the *Dehalobacteriia*. (**a**) Carbon fixation and DCM degradation-related functions, (**b**) central carbon metabolism, (**c**) potential electron bifurcating complexes and (d) legend showing phylogenetic relationships of *Dehalobacteriia* genomes colour-coded by order (red, *Dehalobacteriales*; blue, UBA7702; yellow, UBA4068). The figure was generated using BioRender (https://biorender.com).

In contrast to the *mec* genes involved in DCM degradation, elements of the WLP were commonly found in the *Dehalobacteriia* (Fig. 3a). The complete WLP enables fixation of CO_2_ and production of acetyl-CoA. With the exception of formate dehydrogenase (FdhF), the methyl-branch of the WLP is conserved in all analysed *Dehalobacteriia*. The complete pathway is present in the orders *Dehalobacteriales* and UBA7702 (Fig. 3a), where the carbonyl branch of the WLP comprises a bifunctional carbon monoxide dehydrogenase/acetyl-CoA synthase (AcsAB) that is related to similar enzyme complexes previously described in *Moorella* and *Clostridium* species [53]. The absence of the *acsAB* genes in the order UBA4068 is unusual, as the WLP has been proposed as the major pathway for CO_2_ fixation in related *Bacillota *. However, an absence of several key genes of the WLP has been previously described for the organohalide-respiring bacterial species *Dehalococcoides mccartyi *, a member of the phylum *Chloroflexota* [54]. In *Dehalococcoides mccartyi *, *acsA*, *metF* and *fdhF* were absent, and as a result, no CO_2_ fixation occurred. Instead, external formate was assimilated into glycine and methionine via the reactions catalysed by glycine or methionine synthase [54].

Unlike *Dehalococcoides mccartyi *, *fdhF* and *metF* genes are present in most *Dehalobacteriia*, but homologues of *acsAB* are absent in the order UBA4068. Based on the genes that are present, we propose that members of UBA4068 fix CO_2_ via the reversible reaction of the FdhF [55], with 5,10-methylene tetrahydrofolate being used as a substrate for the glycine cleavage system (GCS). The four enzymes of the GCS, i.e. the aminomethyltransferase GcvT (EC 2.1.2.10; K00605), glycine cleavage protein H (GcvH; K02437), and glycine dehydrogenase subunits 1 and 2 (GcvPA and GcvPB; EC 1.4.4.2; K00282 and K00283), catalyse the formation of glycine that can subsequently be converted to serine and then pyruvate via the reactions of GlyA and SdaAB that are conserved in several *Dehalobacteriia* (Fig. 3a). In *D. formicoaceticum* cultures, ^13^C-glycine was detected as a metabolite during growth with ^13^C-DCM, which supported the presence and function of the GCS [56]. Pyruvate is a central metabolite that can then be used in a variety of metabolic pathways to synthesize new cellular components, but it can also support substrate-level phosphorylation. In this case, pyruvate would be converted to acetyl-CoA and acetyl-phosphate via the reactions catalysed by pyruvate:ferredoxin oxidoreductase (Por; EC 1.2.7.1; K03737) and PduL (EC 2.3.1.222; K015024). Acetyl-phosphate can then be converted to acetate via acetate kinase (AckA), leading to the production of ATP. In the *Dehalobacteriales*, a glycine reductase (Grd, EC 1.21.4.2; K10670-72; K21576-77) is also present that allows direct production of acetyl-phosphate from glycine (Fig. 3a). C1 metabolism in the *Dehalobacteriales* is thus similar to what has been proposed for *Clostridium drakei* [57], while the other two orders of the *Dehalobacteriia* have a more limited carbon fixation repertoire, where carbon assimilation requires formation of serine as an intermediate.

**Fig. 4.**
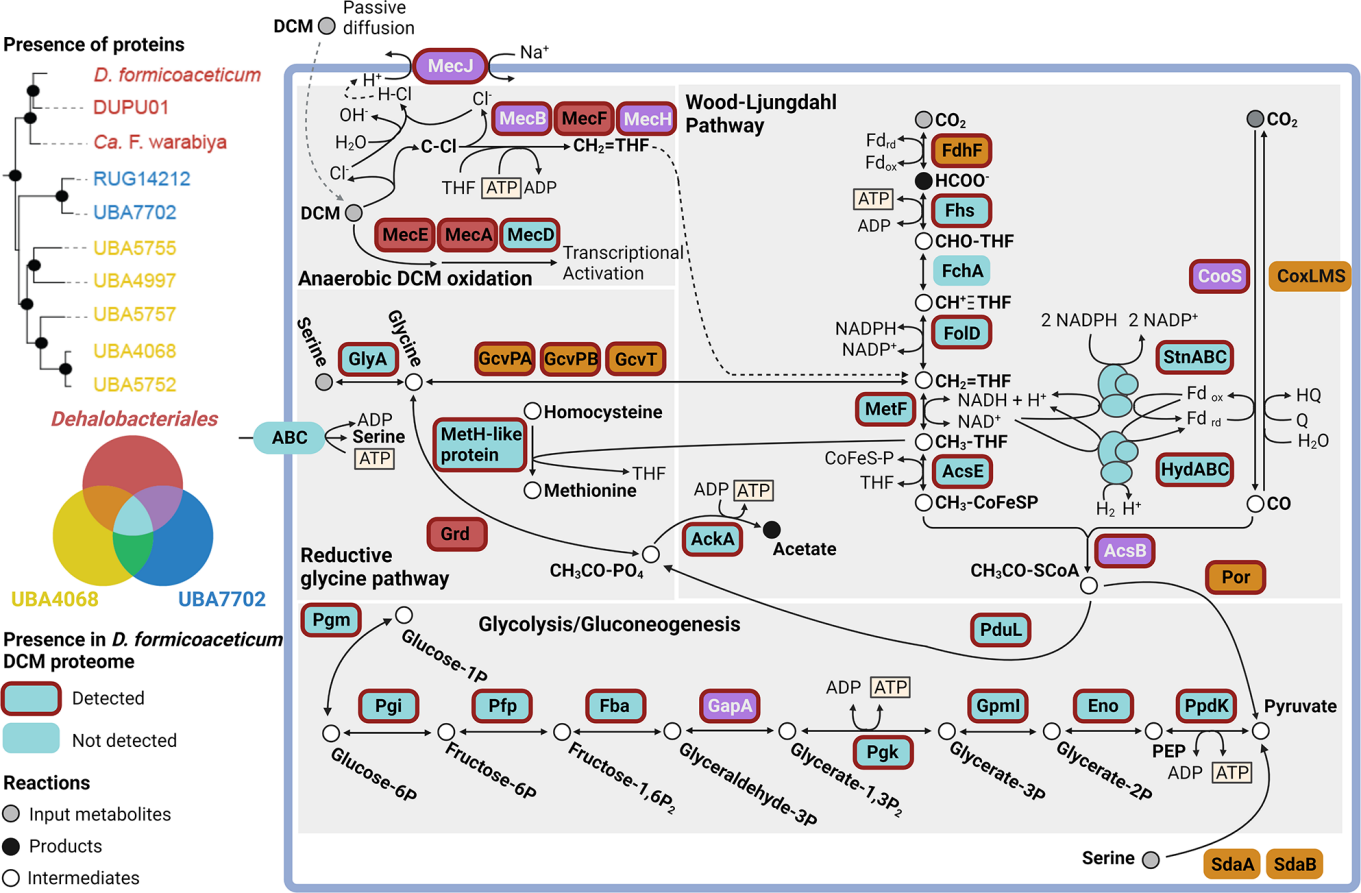
Overview of key metabolic features detected in members of the *Dehalobacteriia*. Protein names are shown in coloured boxes that indicate the orders of the *Dehalobabteriia* in which the proteins are detected (see Venn diagram on the left-hand side). Proteins detected in the *D. formicoaceticum* proteome are indicated by a red outline.

Interestingly, with the exception of the order UBA7702, all examined *Dehalobacteriia* genomes contain operons that encode homologues of the so-called ‘aerobic’, molybdenum-containing CoxLMS carbon monoxide dehydrogenase that converts CO to CO_2_, but is not known to catalyse the reverse reaction (Fig. 4 [57]). In bacteria with a WLP, CO is formed as a by-product of the acetyl-synthase reaction and has been shown to cause growth inhibition in *Dehalococcoides mccartyi *, which only contains a partial WLP [54]. We propose that in the *Dehalobacteriia*, the reaction of ‘aerobic’ CODH could prevent endogenous CO poisoning.

### Pyruvate metabolism

As set out above, pyruvate is a key intermediate in all members of the *Dehalobacteriia*. Once formed, pyruvate can be used by *Dehalobacteriia* for gluconeogenesis (Figs 3b and 4) and to generate intermediates for the pentose phosphate pathway and TCA cycle (e.g. oxaloacetate and malate). These intermediates probably play a role in the biosynthesis of amino acids, as the TCA cycle is incomplete in all members of the *Dehalobacteriia* (Fig. S1). Pyruvate also enables the formation of various fermentative end products. All members of the *Dehalobacteriia* should be able to produce acetate via the reaction of Por described above, while ethanol production is possible via the acetaldehyde alcohol dehydrogenase (AdhE; EC 1.2.1.10; K04072) that is found in members of all three orders of the *Dehalobacteriia*.

### Energy conservation via membrane-bound redox complexes

**Fig. 5.**
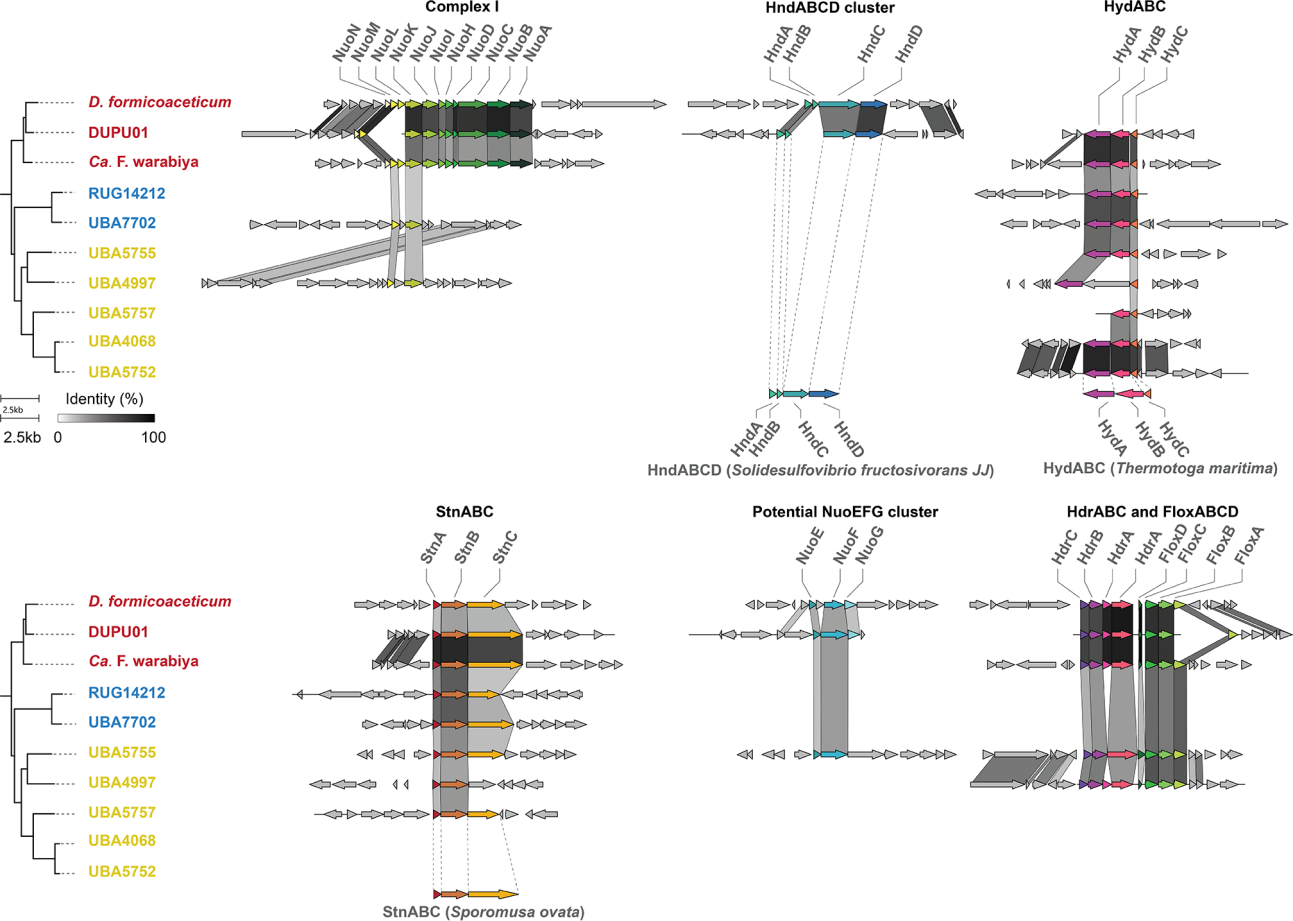
Gene neighbourhood diagrams of redox complexes identified in *Dehalobacteriia* genomes. The greyscale bands indicate amino acid identities between the corresponding pair of genes (a minimum amino acid identity of 30 % was used to connect genes between genomes).

Only members of the order *Dehalobacteriales* have the potential for energy conservation via standard oxidative phosphorylation as they encode the ubiquitous, membrane-bound ATP synthase and 11 Nuo subunits necessary to form a functional NADH dehydrogenase/complex I [58] (Fig. 5). However, all *Dehalobacteriia* genomes encode putative electron-bifurcating protein complexes known to mediate redox processes in related bacteria [24, 25].

When investigating the presence of Complex I homologues, we discovered genes encoding NuoE- and NuoF-like proteins that are homologues of the HydA/HndA and HydB/HndC proteins that occur in electron-bifurcating hydrogenases [24]. Further investigation showed that at least one putative electron-bifurcating hydrogenase is found in all *Dehalobacteriia* genomes. A hydrogenase resembling HydABC from *Thermotoga maritima* [59] is most commonly found in members of the group, although in UBA5757, the gene cluster appears to be incomplete and therefore potentially non-functional (Fig. 5). In *D. formicoaceticum*, the HydABC complex appears to have been replaced by an Hnd-like hydrogenase complex (HndABCD; EC 1.12.1.3; K17992, K18330-2) that has been characterized as catalysing the reduction of NAD^+^ and ferredoxin in *Desulfovibrio fructosovorans* [24, 25]. Interestingly, the DUPU01 genome contains copies of both gene clusters, suggesting functional redundancy (Fig. 5). The presence of conserved HydABC/HndABCD gene clusters in *Dehalobacteriia* indicates that this lineage may use hydrogenase-driven reduction of NAD^+^ and ferredoxin to supply reducing equivalents for CO_2_ fixation via the WLP or conversion of acetyl-CoA to pyruvate catalysed by Por.

In addition to the two hydrogenase-like complexes, we identified a gene cluster that appears to encode an StnABC transhydrogenase-like complex [24] and is present in seven of the ten analysed *Dehalobacteriia* genomes, only being absent in some members of the order UBA4068 (Figs 3c and 4). In this complex, the NuoE- and NuoF-like subunits occur together with a third subunit that contains a GltD domain (Fig. 5), suggesting that it may be able to interconvert the oxidized and reduced forms of NAD^+^, NAD(P)^+^ and ferredoxin as described for *Sporomusa* [24]. The genes encoding this complex occur together with a gene encoding a cytoplasmic, Mo-containing formate dehydrogenase; however, the functional significance of this arrangement is unclear at present. A third gene cluster is present in members of the orders *Dehalobacteriales* and UB4068 and consists of the NuoE- and NuoF-like subunits as well as a subunit with homology to NuoG and other iron–sulphur cluster proteins (Fig. 5). We were unable to identify characterized, close homologues of this gene cluster, but it is possible that this complex may function as a hydrogenase, similar to HndABC. Despite the presence of multiple hydrogenases and the potential in some orders to fix CO_2_ via the formate dehydrogenase reaction, the ability to grow as hydrogen-dependent chemolithotrophs has been excluded for *D. formicoaceticum* and ‘*Ca*. F. warabiya’ [9, 10].

Two additional redox complexes that have been demonstrated to be involved in redox reactions in sulphate-reducing bacteria [60] are present in the *Dehalobacteriales* and some members of the order UBA4068. These gene clusters encode an HdrABC heterodisulphide reductase-like complex, which is associated with genes encoding a Flox-type NADH dehydrogenase that has been proposed to deliver electrons to the Hdr system (Fig. 5). The role of the HdrABC and Flox complexes in *Dehalobacteriia* is currently unknown but could involve providing reducing equivalents to the WLP.

### Amino acid metabolism

As already indicated, CO_2_ fixation in *Dehalobacteriia* via the WLP appears to be connected to the synthesis of glycine/serine and possibly methionine as end products (Fig. 4). The inferred reliance of *Dehalobacteriia* on glycine and serine as intermediates in carbon assimilation suggests that they may be able to use these amino acids as carbon sources. Externally supplied glycine could be converted to serine using serine/glycine hydroxymethyltransferase (GlyA; EC 2.1.2.1; K00600) that is conserved in most members of this class, and both externally supplied and endogenously produced serine is predicted to be assimilated as pyruvate after conversion by l-serine dehydratase (SdaAB; EC 4.3.1.17; K01752) as described above. In the *Dehalobacteriales*, glycine could alternatively be converted to acetyl-phosphate by glycine reductase. With the exception of *D. formicoaceticum,* members of the *Dehalobacteriales* and UBA7702 orders may also be able to convert threonine to glycine for assimilation using threonine aldolase (LtaE; K01620; EC 4.1.2.48). This is an interesting discovery, as growth on amino acids alone has so far not been demonstrated for the well-characterized isolated representative of this group, *D. formicoaceticum*, and warrants further investigation.

### Experimental verification of metabolic inferences

To verify some of our metabolic inferences for the *Dehalobacteriia*, we generated proteome data (*n*=3 biological replicates) for *D. formicoaceticum* grown with DCM as the only energy source. An average of 1294±43 proteins (false discovery rate of 1 %) were detected in the replicate samples, representing 1340 unique proteins. Of these, 1012 proteins (Table S8) or 28 % of the theoretical *D. formicoaceticum* proteome were detected in all three DCM-culture replicates and used for further analyses. Of these 1012 proteins, 702 could be assigned a KEGG ID, collectively representing 22 functional protein categories (Tables S9 and S10). The five most abundant categories were ‘Genetic information processing’ (16.4 % of the KEGG-annotated proteins, 115 proteins), followed by ‘Amino acid metabolism’ (9.6 %, 97 proteins), ‘Energy metabolism’ (8.9 %, 90 proteins), ‘Metabolism of cofactors and vitamins (8.3 %, 84 proteins)’ and ‘Carbohydrate metabolism’ (7.9 %, 80 proteins). These categories are all associated with basic cellular function, which is expected.

All enzymes needed for both the methyl- and carbonyl- branches of the WLP, the degradation of DCM via Mec proteins and enzymes involved in the reductive glycine pathway (i.e. GCS and glycine reductase) were identified and most were present in the top 10 % of detected proteins in all three biological replicates (5/8 WLP, 5/6 Mec proteins and 6/13 proteins of the reductive glycine pathway) (Table S8). The presence of Mec, WLP and reductive glycine pathway proteins in the proteomics data is consistent with results described by Murdoch *et al.* [12], confirming the importance of C1-assimilation pathways in DCM metabolism of *D. formicoaceticum*. Furthermore, the presence of serine hydroxymethyltransferase (GlyA) and glycine reductase (Grd) suggests that *D. formicoaceticum* may be able to assimilate serine into biomass as predicted from metabolic reconstruction (Fig. 4). Our prediction that methionine is a product of the WLP was supported by the observation that a methionine synthase (MetH)-related protein was also highly abundant (Table S8). Production of acetate, a known end product of *D. formicoaceticum* metabolism, is probably catalysed by AckA and PduL that were also present in the top 10 % of detected proteins.

In addition to C1 metabolism-related proteins, several proteins involved in amino acid transport and metabolism were highly abundant. This included an NADP^+^-specific glutamate dehydrogenase (GdhA; EC 1.4.1.4; K00262), a branched-chain amino acid aminotransferase (IlvE; EC 2.6.1.42; K00826), as well as tryptophan synthase (EC 4.2.1.20; K01695 and K01696), diaminopimelate decarboxylase (LysA; EC 4.1.1.20; K01586) and ll-diaminopimelate aminotransferase (K10206; EC 2.6.1.83). A substrate-binding protein of an ATP-binding cassette (ABC) transporter with specificity for leucine, isoleucine and valine (LivK; K01999) was also detected (Table S4). This suggests that amino acids play an important role in *D. formicoaceticum* metabolism even during growth on DCM-containing minimal medium. In this medium the small amount of yeast extract added would have provided some limited access to an external supply of amino acids. Unexpectedly, the top 10 % of proteins also contained a taurine:pyruvate aminotransferase and a sulfoacetaldehyde-acetyltransferase (Table S8) that allow the conversion of taurine and pyruvate to acetyl-phosphate and sulphite via the intermediate sulfoacetaldehyde, indicating that *D. formicoaceticum* may be able to use taurine as a carbon source.

### Energy conservation via redox complexes

Proteins of several of the putative electron-bifurcating complexes described above were also abundant in the proteome. All four subunits of the putative cytoplasmic [FeFe] hydrogenase Hnd (HndABCD) were present in the top 10 % of detected proteins (Table S11). Similarly, all subunits of the protein complex that resembles the *Sporomusa* StnABC transhydrogenase were present in the top 10 % of expressed proteins (Tables S8 and S11). Other electron-bifurcating complexes that do not use hydrogen as an electron donor, including HdrABC heterodisulphide reductase/FloxABCD were also highly abundant (Tables S8 and S11). Overall, the abundance of proteins from electron-bifurcating complexes was similar to core WLP proteins, suggesting that they play key roles in energy conservation of *D. formicoaceticum* and that hydrogenase activity may be linked to DCM degradation.

### S-layer proteins are abundant in *D. formicoaceticum* during growth on DCM

**Fig. 6.**
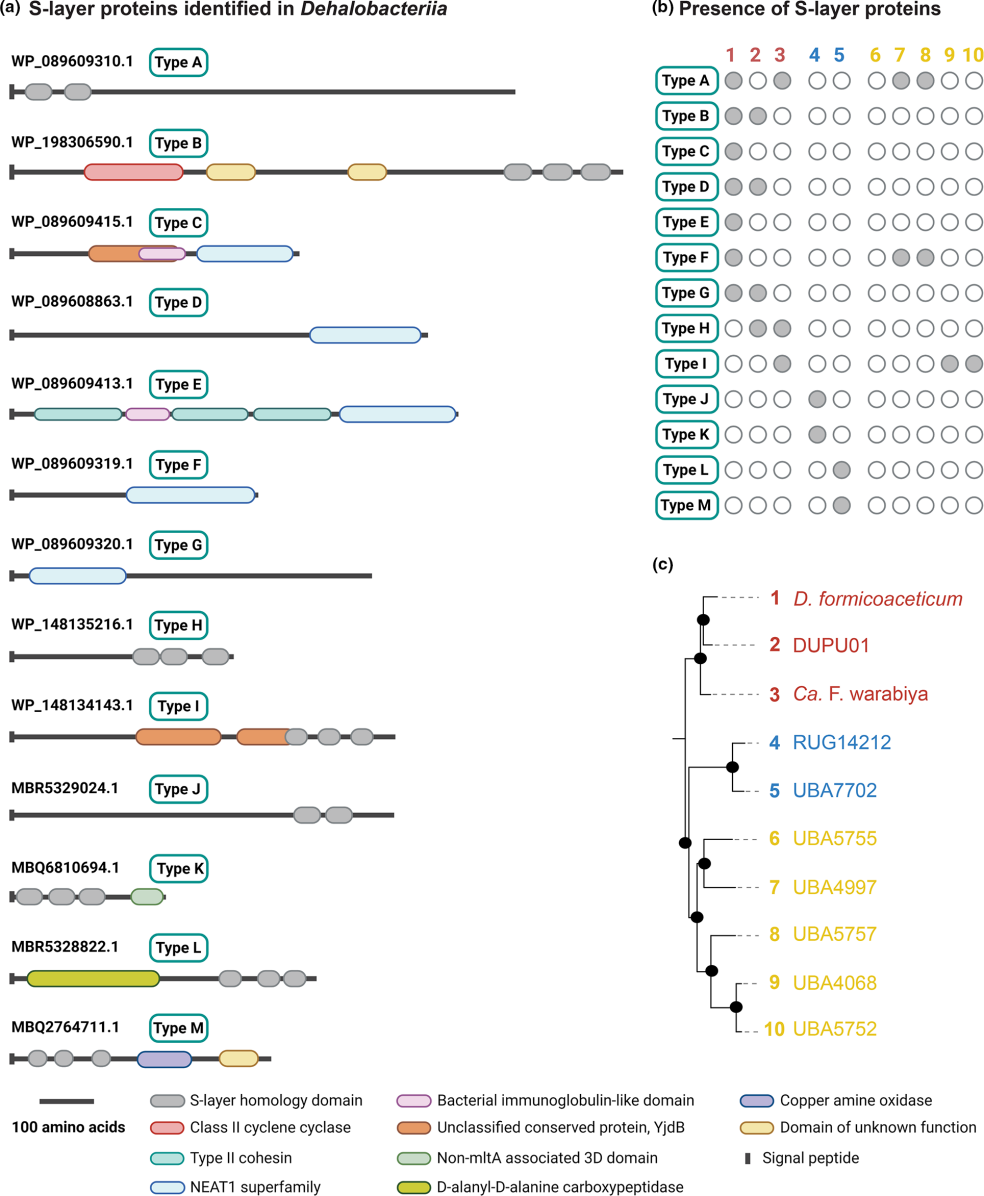
**(a**) S-layer proteins identified in *Dehalobacteriia* genomes. (**b**) Identification of S-layer proteins in *Dehalobacteriia* genomes based on blastp homology search against *D. formicoaceticum* proteins. Filled and open circles indicate presence and absence of proteins respectively. (**c**) Legend showing phylogenetic relationships of *Dehalobacteriia* genomes colour-coded by order (Fig. 1).

In addition to proteins involved in energy and carbon metabolism, seven surface layer (S-layer) proteins (SLPs; WP_089609413.1, WP_089608863.1, WP_089609310.1, WP_198306590.1, WP_089609319.1 and WP_089609320.1) were present in the top 10 % of detected proteins (Table S8), demonstrating that, as in many other bacteria, SLPs are highly abundant in *D. formicoaceticum*. SLPs typically contain one or more S-layer homology domain (pfam00395) at either the N- or C-terminus of the protein (Fig. 6) with a number of other domains known to occur that determine SLP function [61]. Firmicute SLPs are best characterized in *Bacillus anthracis* and *Clostridioides difficile *, where they facilitate functions such as growth phase adaptation, and interactions with host cells and other surfaces [62]. However, *D. formicoaceticum* SLPs had low similarity to *B. anthracis* and *C. difficile* SLPs (<35 % amino acid identity), and this was limited to the S-layer homology or NEAr Transporter (NEAT; cd06920) domains representing between 16 and 34 % of the protein. NEAT domains have been suggested to play a role in iron transport [63]. The only exception was SLP WP_089609319.1, for which an alignment of the protein sequence to its closest homologue in *B. anthracis* (WP_140420396.1) covered 68 % of the sequence length with an amino acid identity of 30 %.

While S-layer homology and NEAT domains were commonly found in the *D. formicoaceticum* SLPs, we also identified other associated domains (Fig. 6). One SLP (WP_089609413.1) has multiple ‘Type II cohesion domains (cd08547)’ that have been shown to play a role in the attachment of the cellulose-degrading cellulosome to the bacterial cell wall. This SLP also contains a single bacterial IG-like domain (pfam02368), which mediates host interactions in *Escherichia coli* and has also been detected in SLPs from *Bacillus* species [63]. However, most of the *D. formicoaceticum* SLPs, including the most highly abundant one (WP_198306590.1), lack identifiable domains and therefore their function remains unclear. It is interesting to note, however, that while SLPs from *B. anthracis* appear to always contain three consecutive S-layer homology domains, this is only the case for one of the *D. formicoaceticum* SLPs. In summary, we show that SLPs found in *D. formicoaceticum* are highly expressed during growth on DCM but may also play key roles under other growth conditions. Additionally, they have novel protein domain structures that add to our knowledge of SLPs and warrant biochemical characterization.

Metabolic reconstruction indicated that *Dehalobacteriia* including *D. formicoaceticum*, which is known as an obligate DCM-degrader [10], may be able to use amino acids such as serine as a carbon and energy source. To test this, we grew *D. formicoaceticum* in mineral medium containing 10 mM serine as the carbon source or 10 mM serine in combination with 2 mM DCM for 31 days, using three biological replicate cultures for each condition.

**Fig. 7.**
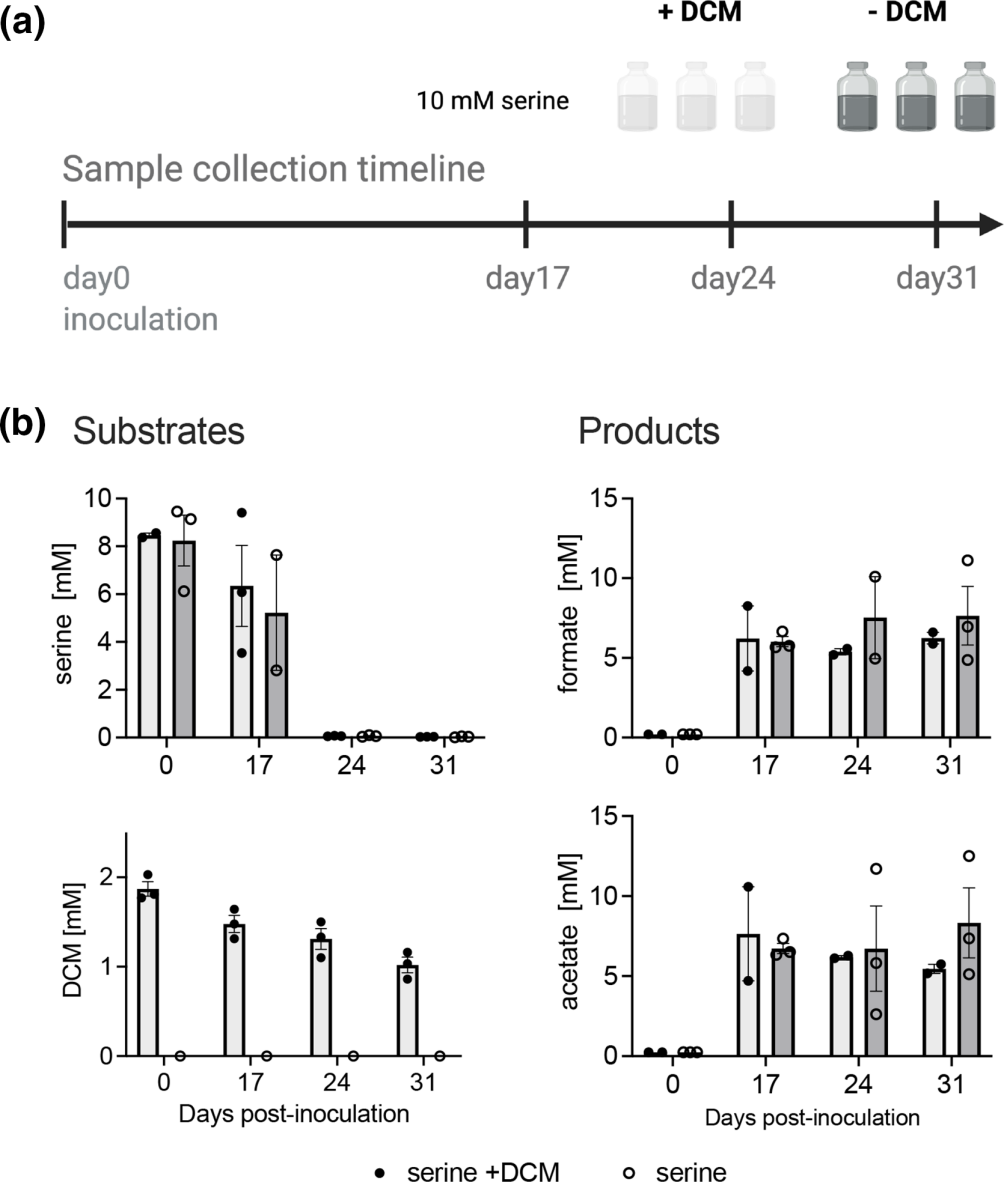
Serine degradation by *D. formicoaceticum* in the presence or absence of DCM. (**a**) Experimental set-up. (**b**) GC-MS measurement of serine, formate, acetate and DCM in both DCM and non-DCM samples over a 31 day cultivation period.

In all cultures, turbidity indicated bacterial growth and on day 17 post-inoculation only 0.06±0.01 and 0.07±0.03 mM serine remained in samples with and without DCM, respectively. In cultures with added DCM, the average DCM concentration decreased from 1.9±0.1 to 1.5±0.1 mM at day 17. Serine-only cultures produced acetate (5.4±3.7 mM) and formate (6.5±4.8 mM) as the dominant end products. These products are consistent with cultures containing DCM only, although differing from the 2 : 1 molar ratio of formate and acetate previously observed [10]. Cultures containing both serine and DCM also produced these end products (7.3±0.6 mM acetate, 8.1±0.6 mM formate), which was expected due to the presence of DCM. On day 17, all cultures were supplemented with an additional 10 mM serine and 2 mM DCM that continued to be consumed until day 31 (Fig. 7).

These observations indicate that *D. formicoaceticum* is able to use serine as a growth substrate in the absence of DCM, consistent with the metabolic reconstruction for members of the *Dehalobacteriia* (Fig. 4). Serine metabolism may have proceeded along various routes including assimilation into the WLP using the glycine cleavage system, or the SdaAB enzyme system. We note, however, that in the proteomics data we collected, high levels of expression were only observed for the reductive glycine system, while SdaAB was not present (Table S4). Furthermore, the proteins necessary to convert serine to the observed end products, acetate and formate, are conserved in the orders of *Dehalobacteriia*, and expressed in *D. formicoaceticum* during growth in the presence of DCM alone, which might indicate that they are constitutively produced (Figs 3a and 4; Table S8). Metabolism of serine is a novel expansion of the known substrate range of *D. formicoaceticum*, which has been considered an obligate DCM degrader for over 20 years [10]. ‘*Ca.* F. warabiya’ has also been reported to grow with substrates other than DCM, including choline, glycine betaine and methanol [12]. Notably, DCM consumption proceeded at a slower rate than usual in the DCM and serine cultures, suggesting that serine may be used preferentially, or cause inhibition of DCM metabolism.

## Concluding remarks

In summary, we show that anaerobic DCM degradation, regarded as a defining trait of *D. formicoaceticum*, is probably only present in two of the ten investigated members of the class *Dehalobacteriia* and, moreover, appears to have been recently acquired via horizontal gene transfer. By contrast, use of serine as a growth substrate, and use of the glycine cleavage pathway and WLP in central carbon metabolism appear to be broadly conserved in this class. A number of predicted electron-bifurcating complexes identified in the genomes of these bacteria appear to support fermentative metabolism and warrant further investigation. The validity of these inferences was confirmed using the only pure culture representative of the *Dehalobacteriia*, *D. formicoaceticum*, which was able to grow on serine in the absence of DCM. Taken together, our data indicate that DCM degradation is a recently acquired and restricted trait in the *Dehalobacteriia* and that amino acid fermentation is a widespread and ancestral trait in this lineage.

## Supplementary Data

Supplementary material 1

Supplementary material 2
