# Corrigendum: ‘A comparative genome analysis of the *Bacillota (Firmicutes*) class *Dehalobacteriia’*

**DOI:** 10.1099/mgen.0.001092

**Published:** 2023-08-09

**Authors:** Young C. Song, Sophie I. Holland, Matthew Lee, Gao Chen, Julian Zaugg, Frank E. Löffler, Michael J. Manefield, Philip Hugenholtz, Ulrike Kappler

**Affiliations:** ^1^​ The University of Queensland, School of Chemistry and Molecular Biosciences, Australian Centre for Ecogenomics, St Lucia, Queensland, 4072, Australia; ^2^​ School of Civil and Environmental Engineering, UNSW Sydney, Sydney, NSW, 2052, Australia; ^3^​ Center for Environmental Biotechnology, Department of Civil and Environmental Engineering, University of Tennessee, Knoxville, Tennessee, USA; ^4^​ Department of Microbiology, Department of Bioengineering and Soil Science, University of Tennessee, Knoxville, Tennessee, USA; ^5^​ Oak Ridge National Laboratory, Biosciences Division, Oak Ridge, Tennessee, USA; ^6^​ The University of Queensland, School of Chemistry and Molecular Biosciences, St Lucia, Queensland, 4072, Australia; ^†^​ Present address: School of Engineering & Physical Sciences, Heriot-Watt University, Edinburgh, UK

## Full-Text

In the published version of the article, Dr Julian Zaugg was omitted as a co-author, and now has been added as an author in fifth position. His contributions to the manuscript (implementation of bioinformatics pipelines used for functional inference and proteomics analysis) occurred very early during the project and consequently he was inadvertently omitted. This has not impacted the Acknowledgments or Conflicts of Interest for the manuscript.

The authors apologise for any inconvenience caused by these errors.

