# Supplementary material for: A comparative genome analysis of the Bacillota (Firmicutes) class Dehalobacteriia
Source: Microb Genom. 2023 Jun 9;9(6):mgen001039. doi: 10.1099/mgen.0.001039 (PMC10327494; doi:10.1099/mgen.0.001039)

Table S1. List of *Dehalobacteriia* genomes used in the analysis (in green rows)

Types of the genomes provided in this table are isolate, mixed culture and MAG
Cmpl. : CheckM completion rate
Cntmn: ChecM contamination rate

Order	Genome	Genome Assembly ID	Size (Mb)	Cmpl. (%)	Cntmn (%)	GC (%)	CD (%)	Protein Count	16S	Ref.
o__Dehalobacteriales	*D.formicoaceticum*	GCF_002224645.1	3.8	95.6	2.6	43.2	86.1	3,620	Yes	9
	Ca . F. warabiya	GCF_002777255.1	6.4	98.9	2.7	46.4	87.5	5,804	Yes	11
	DUPU01	GCA_012838175.1	2.4	91.9	2.0	42.1	88.8	2,447	No	38
o__UBA4068	HGM13862	GCF_900760825.1	1.7	79.9	2.7	47.6	87.1	1,878	No	N/A
	AS06rmzACSIP_243	GCA_012515255.1	1.3	71.2	2.0	55.4	88.6	1,371	No	38
	UBA5752	GCA_002419055.1	2.1	91.9	0.7	42.7	88.8	1,965	No	36
	UBA4068	GCA_002382665.1	2.0	91.2	0.3	43.2	89.0	1,966	No	36
	UBA5757	GCA_002418965.1	1.9	89.1	0.9	43.8	86.2	1,808	Yes	36
	Lab288P3bin203	GCA_009783495.1	1.1	59.6	1.8	55.7	87.6	1.315	No	38
	Nt197P3bin103	GCA_009783415.1	2.8	64.2	2.5	46.6	86.8	2,197	No	38
	Nt197P4bin8	GCA_009778255.1	2.2	70.8	1.7	42.9	90.0	2,024	No	38
	Nt197P3bin19	GCA_009782975.1	0.9	57.8	0.9	52.2	90.0	968	No	38
	UBA4997	GCA_002398485.1	1.7	78.5	0.8	40.5	74.9	1,568	Yes	39
	UBA5755	GCA_002419005.1	1.5	90.9	0.5	42.2	89.7	1,456	No	36
o__UBA7702	UBA7702	GCA_002482535.1	2.6	93.1	0.9	49.5	88.8	2,402	No	40
	RUG14212	GCA_902796855.1	2.1	84.8	2.6	57.4	88.8	1,936	No	37
	RUG11176	GCA_902766525.1	1.5	80.5	0.9	49.9	90.4	1,649	No	37

Table S2. List of primer sequences matched against 16S sequences from *Dehalobacteriia*

a. Primer sequences from Engelbrekston et al., ISMEJ 2010, representative of commonly used 16S rRNA gene primers
b. Metacharacters: K=GT, M=AC, R=AG, S=CG, W=AG, Y=CT
c. matches, mismatches

16S rRNA primer (a)	5'-3' sequence (b)	Dehalobacteriia sequence (c)	Variants	Comments
27F	AGAGTTTGATCMTGGCTCAG	AGAGTTTGATCCTGGCTCAG	f__UBA5755: AGAGTTTGATCCTGGATCAG	Should amplify, with exception of variant
357F	CTCCTACGGGAGGCAGCAG	CTCCTACGGGAGGCAGCAG	none	Should amplify
530F	GTGCCAGCMGCCGCGG	GTGCCAGCAGCCGCGG	none	Should amplify
803F	ATTAGATACCCTGGTAGTC	ATTAGATACCCCGGTAATC	none	Probably won't amplify with mismatches near 3' end
926F	AAACTYAAAKGAATTGACGG	AAACTCAAAGGAATTGACGG	none	Should amplify
1114F	GCAACGAGCGCAACCC	GCAACGAGCGCAACCC	none	Should amplify
342R	CTGCTGCSYCCCGTAG	CTGCTGCCTCCCGTAG	none	Should amplify
519R	GWATTACCGCGGCKGCTG	GTATTACCGCGGCTGCTG	none	Should amplify even with mismatch at 5' end
787R	CTACCAGGGTATCTAAT	TTACCGGGGTATCTAAT	none	May amplify, noting mismatches closer to 5' end
907R	CCGTCAATTCMTTTRAGTTT	CCGTCAATTCCTTTGAGTTT	none	Should amplify
1100R	GGGTTGCGCTCGTTG	GGGTTGCGCTCGTTG	none	Should amplify
1392R	ACGGGCGGTGTGTRC	ACGGGCGGTGTGTAC	none	Should amplify

Table S3. List of 16S rRNA gene amplicon datasets associated with the identified *Dehalobacteriia* sequences

5% raw num: raw number of sequences that share 95% or higher similarity to *Dehalobacteriia* sequences

5% perc: proportion pf the ''5% raw num" sequences out of total downloaded sequences from each study

Isolation Source	Reference	Total Downloaded Sequences	5% raw num	5% perc
Mouse cecum	41	7223	7	0.10
Cecal contents from Mus musculus strain C57BL/6J; sample lean_donor, leptin genotype +/+	42	4157	6	0.14
Cecum of mother 1 mouse offspring with genotype ob/ob	43	Offspring: 3998	Offspring: 5	0.13
Cecum of mother 1 mouse offspring with genotype ob/ob	43	Mother: 1120	Mother: 2	0.18
Human feces; subj10; 12 weeks; carb-r diet	44	6909	1	0.01
Insect gut	45	623	3	0.48
Push core sediment sample from the vadose zone of a hydrocarbon contaminated aquifer	46	486	3	0.62
Mud volcano	47	185	2	1.08

Table S4. List of KEGG Orthology IDs and their presence/absence in Dehalobacteriia genomes based on BlastKOALA search

KO_ID	Description	*D. formicoaceticum*	*Ca* . F. warabiya	DUPU01	UBA7702	RUG14212	UBA4997	UBA5755	UBA5757	UBA4068	UBA5752
K00001	E1.1.1.1, adh; alcohol dehydrogenase [EC:1.1.1.1]	0	0	1	0	2	0	0	1	1	1
K00003	hom; homoserine dehydrogenase [EC:1.1.1.3]	1	1	2	1	1	1	1	1	1	1
K00004	BDH, butB; (R,R)-butanediol dehydrogenase / meso-butanediol dehydrogenase / diacetyl reductase [EC:1.1.1.4 1.1.1.- 1.1.1.303]	0	0	1	0	0	0	0	0	0	0
K00008	SORD, gutB; L-iditol 2-dehydrogenase [EC:1.1.1.14]	0	0	3	1	1	0	0	0	0	0
K00012	UGDH, ugd; UDPglucose 6-dehydrogenase [EC:1.1.1.22]	1	0	2	0	0	0	0	0	0	0
K00013	hisD; histidinol dehydrogenase [EC:1.1.1.23]	1	1	3	1	1	1	0	1	1	1
K00014	aroE; shikimate dehydrogenase [EC:1.1.1.25]	1	1	2	1	1	2	0	1	1	1
K00015	gyaR, GOR1; glyoxylate reductase [EC:1.1.1.26]	0	0	0	0	0	0	1	0	0	0
K00016	LDH, ldh; L-lactate dehydrogenase [EC:1.1.1.27]	0	0	0	1	0	0	0	0	0	0
K00018	hprA; glycerate dehydrogenase [EC:1.1.1.29]	0	0	1	0	0	0	0	1	0	0
K00027	ME2, sfcA, maeA; malate dehydrogenase (oxaloacetate-decarboxylating) [EC:1.1.1.38]	1	1	4	0	0	0	1	1	1	1
K00030	IDH3; isocitrate dehydrogenase (NAD+) [EC:1.1.1.41]	0	0	0	0	0	1	0	0	0	0
K00031	IDH1, IDH2, icd; isocitrate dehydrogenase [EC:1.1.1.42]	1	0	3	0	0	0	0	1	0	0
K00034	gdh; glucose 1-dehydrogenase [EC:1.1.1.47]	0	0	1	0	0	0	0	0	0	0
K00038	E1.1.1.53; 3alpha(or 20beta)-hydroxysteroid dehydrogenase [EC:1.1.1.53]	0	0	0	1	1	0	0	0	0	0
K00046	idnO; gluconate 5-dehydrogenase [EC:1.1.1.69]	0	0	0	0	0	0	0	2	0	0
K00052	leuB, IMDH; 3-isopropylmalate dehydrogenase [EC:1.1.1.85]	1	1	1	0	1	1	0	2	1	1
K00053	ilvC; ketol-acid reductoisomerase [EC:1.1.1.86]	1	1	1	0	1	1	1	1	1	1
K00057	gpsA; glycerol-3-phosphate dehydrogenase (NAD(P)+) [EC:1.1.1.94]	2	1	1	0	1	1	1	1	1	1
K00058	serA, PHGDH; D-3-phosphoglycerate dehydrogenase / 2-oxoglutarate reductase [EC:1.1.1.95 1.1.1.399]	1	1	3	2	1	1	1	1	1	1
K00059	fabG, OAR1; 3-oxoacyl-[acyl-carrier protein] reductase [EC:1.1.1.100]	3	2	8	0	4	2	1	3	2	1
K00060	tdh; threonine 3-dehydrogenase [EC:1.1.1.103]	0	0	1	0	0	0	0	0	0	0
K00065	kduD; 2-dehydro-3-deoxy-D-gluconate 5-dehydrogenase [EC:1.1.1.127]	0	0	2	0	0	0	0	0	0	0
K00067	rfbD, rmlD; dTDP-4-dehydrorhamnose reductase [EC:1.1.1.133]	0	0	0	0	1	0	0	0	0	0
K00073	allD; ureidoglycolate dehydrogenase (NAD+) [EC:1.1.1.350]	0	0	1	0	0	0	0	0	0	0
K00074	paaH, hbd, fadB, mmgB; 3-hydroxybutyryl-CoA dehydrogenase [EC:1.1.1.157]	0	0	5	0	0	0	0	0	1	1
K00075	murB; UDP-N-acetylmuramate dehydrogenase [EC:1.3.1.98]	1	1	1	1	1	1	1	1	1	1
K00077	panE, apbA; 2-dehydropantoate 2-reductase [EC:1.1.1.169]	0	0	2	0	1	0	1	1	2	2
K00087	ygeS, xdhA; xanthine dehydrogenase molybdenum-binding subunit [EC:1.17.1.4]	5	2	18	0	0	2	1	2	5	4
K00088	IMPDH, guaB; IMP dehydrogenase [EC:1.1.1.205]	1	2	2	1	1	1	1	2	1	1
K00090	ghrB; glyoxylate/hydroxypyruvate/2-ketogluconate reductase [EC:1.1.1.79 1.1.1.81 1.1.1.215]	0	0	1	0	0	0	0	0	0	0
K00099	dxr; 1-deoxy-D-xylulose-5-phosphate reductoisomerase [EC:1.1.1.267]	1	1	1	1	1	1	1	1	1	1
K00100	bdhAB; butanol dehydrogenase [EC:1.1.1.-]	0	0	1	0	0	0	0	0	0	0
K00102	LDHD, dld; D-lactate dehydrogenase (cytochrome) [EC:1.1.2.4]	0	0	0	0	0	0	0	1	1	1
K00104	glcD; glycolate dehydrogenase FAD-linked subunit [EC:1.1.99.14]	1	0	7	0	0	0	0	0	0	0
K00111	glpA, glpD; glycerol-3-phosphate dehydrogenase [EC:1.1.5.3]	0	0	2	1	1	0	0	0	0	0
K00112	glpB; glycerol-3-phosphate dehydrogenase subunit B [EC:1.1.5.3]	0	0	1	0	0	0	0	0	0	0
K00113	glpC; glycerol-3-phosphate dehydrogenase subunit C	0	0	1	0	0	0	0	0	0	0
K00121	frmA, ADH5, adhC; S-(hydroxymethyl)glutathione dehydrogenase / alcohol dehydrogenase [EC:1.1.1.284 1.1.1.1]	0	0	1	0	0	0	1	0	0	0
K00123	fdoG, fdhF, fdwA; formate dehydrogenase major subunit [EC:1.17.1.9]	2	2	2	0	0	0	1	0	0	0
K00124	fdoH, fdsB; formate dehydrogenase iron-sulfur subunit	0	1	0	0	1	1	0	0	0	0
K00128	ALDH; aldehyde dehydrogenase (NAD+) [EC:1.2.1.3]	0	0	2	1	2	0	1	0	1	0
K00130	betB, gbsA; betaine-aldehyde dehydrogenase [EC:1.2.1.8]	0	0	1	0	0	0	0	0	0	0
K00131	gapN; glyceraldehyde-3-phosphate dehydrogenase (NADP+) [EC:1.2.1.9]	0	0	0	0	0	0	1	0	0	0
K00132	E1.2.1.10; acetaldehyde dehydrogenase (acetylating) [EC:1.2.1.10]	2	1	1	1	1	0	0	0	0	0
K00133	asd; aspartate-semialdehyde dehydrogenase [EC:1.2.1.11]	1	1	2	1	1	1	0	1	1	1
K00134	GAPDH, gapA; glyceraldehyde 3-phosphate dehydrogenase (phosphorylating) [EC:1.2.1.12]	1	1	1	1	1	1	1	1	1	1
K00135	gabD; succinate-semialdehyde dehydrogenase / glutarate-semialdehyde dehydrogenase [EC:1.2.1.16 1.2.1.79 1.2.1.20]	1	0	2	0	0	0	0	0	0	0
K00140	mmsA, iolA, ALDH6A1; malonate-semialdehyde dehydrogenase (acetylating) / methylmalonate-semialdehyde dehydrogenase [EC:1.2.1.18 1.2.1.27]	1	0	1	0	0	0	0	0	0	0
K00145	argC; N-acetyl-gamma-glutamyl-phosphate reductase [EC:1.2.1.38]	1	1	1	1	1	1	1	1	1	1
K00147	proA; glutamate-5-semialdehyde dehydrogenase [EC:1.2.1.41]	1	0	1	1	1	1	0	1	1	1
K00169	porA; pyruvate ferredoxin oxidoreductase alpha subunit [EC:1.2.7.1]	1	1	3	0	0	1	1	1	2	2
K00170	porB; pyruvate ferredoxin oxidoreductase beta subunit [EC:1.2.7.1]	1	1	2	0	0	1	1	1	2	2
K00171	porD; pyruvate ferredoxin oxidoreductase delta subunit [EC:1.2.7.1]	1	1	3	0	0	1	0	1	2	2
K00172	porC, porG; pyruvate ferredoxin oxidoreductase gamma subunit [EC:1.2.7.1]	1	1	3	0	0	1	1	1	2	2
K00174	korA, oorA, oforA; 2-oxoglutarate/2-oxoacid ferredoxin oxidoreductase subunit alpha [EC:1.2.7.3 1.2.7.11]	1	1	7	3	3	1	2	1	1	1
K00175	korB, oorB, oforB; 2-oxoglutarate/2-oxoacid ferredoxin oxidoreductase subunit beta [EC:1.2.7.3 1.2.7.11]	1	1	7	3	3	1	2	1	1	1
K00176	korD, oorD; 2-oxoglutarate ferredoxin oxidoreductase subunit delta [EC:1.2.7.3]	0	1	6	3	3	0	2	1	1	1
K00177	korC, oorC; 2-oxoglutarate ferredoxin oxidoreductase subunit gamma [EC:1.2.7.3]	0	1	7	3	3	1	2	1	1	1
K00179	iorA; indolepyruvate ferredoxin oxidoreductase, alpha subunit [EC:1.2.7.8]	1	2	4	2	2	1	3	2	1	1
K00180	iorB; indolepyruvate ferredoxin oxidoreductase, beta subunit [EC:1.2.7.8]	1	2	4	2	2	1	3	2	1	1
K00184	dmsB; dimethyl sulfoxide reductase iron-sulfur subunit	1	1	2	0	0	0	0	0	0	0
K00185	dmsC; dimethyl sulfoxide reductase membrane subunit	1	1	1	0	0	0	0	0	0	0
K00194	cdhD, acsD; acetyl-CoA decarbonylase/synthase, CODH/ACS complex subunit delta [EC:2.1.1.245]	1	1	1	1	1	0	0	0	0	0
K00196	cooF; anaerobic carbon-monoxide dehydrogenase iron sulfur subunit	1	1	2	1	1	0	0	0	0	1
K00197	cdhE, acsC; acetyl-CoA decarbonylase/synthase, CODH/ACS complex subunit gamma [EC:2.1.1.245]	1	1	1	1	1	0	0	0	0	0
K00198	cooS, acsA; anaerobic carbon-monoxide dehydrogenase catalytic subunit [EC:1.2.7.4]	3	2	3	2	1	0	0	0	0	0
K00200	fwdA, fmdA; formylmethanofuran dehydrogenase subunit A [EC:1.2.7.12]	0	0	1	0	0	0	0	0	0	0
K00201	fwdB, fmdB; formylmethanofuran dehydrogenase subunit B [EC:1.2.7.12]	0	0	1	0	0	0	0	0	0	0
K00203	fwdD, fmdD; formylmethanofuran dehydrogenase subunit D [EC:1.2.7.12]	0	0	1	0	0	0	0	0	0	0
K00215	dapB; 4-hydroxy-tetrahydrodipicolinate reductase [EC:1.17.1.8]	1	1	1	1	2	1	1	1	1	1
K00219	fadH; 2,4-dienoyl-CoA reductase (NADPH2) [EC:1.3.1.34]	0	0	3	0	0	0	2	1	0	1
K00230	hemG; menaquinone-dependent protoporphyrinogen oxidase [EC:1.3.5.3]	0	1	1	0	0	0	0	0	1	1
K00231	PPOX, hemY; protoporphyrinogen/coproporphyrinogen III oxidase [EC:1.3.3.4 1.3.3.15]	1	1	1	0	0	0	0	0	0	0
K00239	sdhA, frdA; succinate dehydrogenase flavoprotein subunit [EC:1.3.5.1]	0	0	6	1	1	1	1	0	0	0
K00240	sdhB, frdB; succinate dehydrogenase iron-sulfur subunit [EC:1.3.5.1]	0	0	3	1	1	1	1	0	0	0
K00241	sdhC, frdC; succinate dehydrogenase cytochrome b subunit	0	0	3	1	1	1	1	0	0	0
K00244	frdA; succinate dehydrogenase flavoprotein subunit [EC:1.3.5.1]	0	0	0	0	0	0	0	1	0	0
K00248	ACADS, bcd; butyryl-CoA dehydrogenase [EC:1.3.8.1]	0	0	2	0	0	0	0	1	0	0
K00261	GLUD1_2, gdhA; glutamate dehydrogenase (NAD(P)+) [EC:1.4.1.3]	0	0	0	0	0	1	0	0	0	0
K00262	E1.4.1.4, gdhA; glutamate dehydrogenase (NADP+) [EC:1.4.1.4]	1	2	2	1	1	1	0	1	1	1
K00263	E1.4.1.9; leucine dehydrogenase [EC:1.4.1.9]	0	0	2	0	0	0	0	0	0	0
K00265	gltB; glutamate synthase (NADPH) large chain [EC:1.4.1.13]	0	0	1	0	0	0	0	0	0	0
K00266	gltD; glutamate synthase (NADPH) small chain [EC:1.4.1.13]	0	1	2	1	0	0	0	0	1	1
K00278	nadB; L-aspartate oxidase [EC:1.4.3.16]	0	0	1	0	1	1	1	1	1	2
K00282	gcvPA; glycine dehydrogenase subunit 1 [EC:1.4.4.2]	1	1	2	0	0	0	1	1	1	1
K00283	gcvPB; glycine dehydrogenase subunit 2 [EC:1.4.4.2]	1	1	2	0	0	0	1	1	1	1
K00284	GLU, gltS; glutamate synthase (ferredoxin) [EC:1.4.7.1]	0	0	0	1	0	0	0	0	0	0
K00286	proC; pyrroline-5-carboxylate reductase [EC:1.5.1.2]	1	1	2	1	1	1	1	1	1	1
K00287	DHFR, folA; dihydrofolate reductase [EC:1.5.1.3]	1	0	0	0	0	0	1	0	0	0
K00290	LYS1; saccharopine dehydrogenase (NAD+, L-lysine forming) [EC:1.5.1.7]	1	0	1	0	1	1	0	1	1	1
K00294	E1.2.1.88; 1-pyrroline-5-carboxylate dehydrogenase [EC:1.2.1.88]	1	0	3	0	0	0	0	1	0	0
K00297	metF, MTHFR; methylenetetrahydrofolate reductase (NADH) [EC:1.5.1.54]	2	1	1	1	1	1	1	1	1	1
K00298	ceo; N5-(carboxyethyl)ornithine synthase [EC:1.5.1.24]	0	0	0	0	0	0	0	0	1	1
K00302	soxA; sarcosine oxidase, subunit alpha [EC:1.5.3.24 1.5.3.1]	2	2	4	1	1	0	0	0	0	0
K00303	soxB; sarcosine oxidase, subunit beta [EC:1.5.3.24 1.5.3.1]	1	1	2	0	0	0	0	0	0	0
K00313	fixC; electron transfer flavoprotein-quinone oxidoreductase [EC:1.5.5.-]	3	1	2	0	0	0	2	1	0	0
K00324	pntA; H+-translocating NAD(P) transhydrogenase subunit alpha [EC:1.6.1.2 7.1.1.1]	2	3	2	0	0	0	0	0	0	0
K00325	pntB; H+-translocating NAD(P) transhydrogenase subunit beta [EC:1.6.1.2 7.1.1.1]	1	1	1	0	0	0	1	0	0	0
K00330	nuoA; NADH-quinone oxidoreductase subunit A [EC:7.1.1.2]	1	1	1	0	0	0	1	0	1	1
K00331	nuoB; NADH-quinone oxidoreductase subunit B [EC:7.1.1.2]	1	1	1	0	0	0	0	0	0	0
K00332	nuoC; NADH-quinone oxidoreductase subunit C [EC:7.1.1.2]	1	0	1	0	0	0	0	0	0	0
K00333	nuoD; NADH-quinone oxidoreductase subunit D [EC:7.1.1.2]	1	1	1	0	0	0	1	0	1	1
K00334	nuoE; NADH-quinone oxidoreductase subunit E [EC:7.1.1.2]	1	4	4	3	2	1	3	2	2	2
K00335	nuoF; NADH-quinone oxidoreductase subunit F [EC:7.1.1.2]	3	4	5	3	2	2	1	2	2	2
K00336	nuoG; NADH-quinone oxidoreductase subunit G [EC:7.1.1.2]	1	3	3	2	2	2	1	0	1	0
K00337	nuoH; NADH-quinone oxidoreductase subunit H [EC:7.1.1.2]	1	1	1	0	0	0	1	0	1	1
K00338	nuoI; NADH-quinone oxidoreductase subunit I [EC:7.1.1.2]	1	1	1	0	0	0	0	0	0	0
K00339	nuoJ; NADH-quinone oxidoreductase subunit J [EC:7.1.1.2]	1	1	1	0	0	0	1	0	1	1
K00340	nuoK; NADH-quinone oxidoreductase subunit K [EC:7.1.1.2]	1	1	1	0	0	0	0	0	1	0
K00341	nuoL; NADH-quinone oxidoreductase subunit L [EC:7.1.1.2]	1	1	1	0	0	0	0	0	0	0
K00342	nuoM; NADH-quinone oxidoreductase subunit M [EC:7.1.1.2]	1	1	1	0	0	0	0	0	1	0
K00343	nuoN; NADH-quinone oxidoreductase subunit N [EC:7.1.1.2]	1	1	1	0	0	0	0	0	0	0
K00354	E1.6.99.1; NADPH2 dehydrogenase [EC:1.6.99.1]	1	0	2	1	0	0	0	0	0	0
K00355	NQO1; NAD(P)H dehydrogenase (quinone) [EC:1.6.5.2]	0	0	0	0	1	0	0	0	0	0
K00362	nirB; nitrite reductase (NADH) large subunit [EC:1.7.1.15]	0	0	0	0	1	0	0	1	1	1
K00374	narI, narV; nitrate reductase gamma subunit [EC:1.7.5.1 1.7.99.-]	1	1	1	0	0	0	0	0	0	0
K00375	K00375; GntR family transcriptional regulator / MocR family aminotransferase	1	0	1	0	0	1	1	1	0	0
K00382	DLD, lpd, pdhD; dihydrolipoamide dehydrogenase [EC:1.8.1.4]	3	1	2	0	0	0	2	2	1	2
K00384	trxB, TRR; thioredoxin reductase (NADPH) [EC:1.8.1.9]	3	2	7	1	1	1	1	1	1	1
K00385	asrC; anaerobic sulfite reductase subunit C	0	0	2	0	0	0	0	0	0	0
K00390	cysH; phosphoadenosine phosphosulfate reductase [EC:1.8.4.8 1.8.4.10]	0	0	0	0	0	0	0	0	0	1
K00425	cydA; cytochrome bd ubiquinol oxidase subunit I [EC:7.1.1.7]	0	0	1	0	0	0	0	0	0	0

K00426	cydB; cytochrome bd ubiquinol oxidase subunit II [EC:7.1.1.7]	0	0	1	0	0	0	0	0	0	0
K00432	gpx, btuE, bsaA; glutathione peroxidase [EC:1.11.1.9]	0	0	0	1	0	0	0	0	0	0
K00433	cpo; non-heme chloroperoxidase [EC:1.11.1.10]	0	0	1	1	0	0	0	1	1	1
K00436	hoxH; NAD-reducing hydrogenase large subunit [EC:1.12.1.2]	0	0	0	0	0	0	1	1	2	1
K00441	frhB; coenzyme F420 hydrogenase subunit beta [EC:1.12.98.1]	0	0	1	0	0	0	0	0	0	0
K00459	ncd2, npd; nitronate monooxygenase [EC:1.13.12.16]	1	0	1	0	0	0	0	0	1	1
K00525	E1.17.4.1A, nrdA, nrdE; ribonucleoside-diphosphate reductase alpha chain [EC:1.17.4.1]	1	1	0	1	1	0	1	1	1	1
K00528	fpr; ferredoxin/flavodoxin---NADP+ reductase [EC:1.18.1.2 1.19.1.1]	0	0	0	0	0	0	0	0	1	1
K00533	E1.12.7.2L; ferredoxin hydrogenase large subunit [EC:1.12.7.2]	0	1	1	0	0	0	0	0	0	0
K00537	arsC; arsenate reductase (glutaredoxin) [EC:1.20.4.1]	0	1	1	1	0	0	1	0	0	0
K00548	metH, MTR; 5-methyltetrahydrofolate--homocysteine methyltransferase [EC:2.1.1.13]	11	6	56	5	9	2	5	2	3	3
K00554	trmD; tRNA (guanine37-N1)-methyltransferase [EC:2.1.1.228]	1	1	1	1	1	1	1	1	1	1
K00558	DNMT1, dcm; DNA (cytosine-5)-methyltransferase 1 [EC:2.1.1.37]	1	0	0	0	1	0	0	0	0	1
K00560	thyA, TYMS; thymidylate synthase [EC:2.1.1.45]	1	1	1	1	1	1	2	1	1	0
K00563	rlmA1; 23S rRNA (guanine745-N1)-methyltransferase [EC:2.1.1.187]	1	0	1	0	0	0	0	0	0	0
K00566	mnmA, trmU; tRNA-uridine 2-sulfurtransferase [EC:2.8.1.13]	1	1	1	1	1	1	1	1	1	2
K00567	ogt, MGMT; methylated-DNA-[protein]-cysteine S-methyltransferase [EC:2.1.1.63]	1	1	2	1	2	1	1	1	1	1
K00568	ubiG; 2-polyprenyl-6-hydroxyphenyl methylase / 3-demethylubiquinone-9 3-methyltransferase [EC:2.1.1.222 2.1.1.64]	0	0	0	0	0	0	0	0	1	1
K00571	E2.1.1.72; site-specific DNA-methyltransferase (adenine-specific) [EC:2.1.1.72]	0	0	0	1	0	0	0	1	2	1
K00573	E2.1.1.77, pcm; protein-L-isoaspartate(D-aspartate) O-methyltransferase [EC:2.1.1.77]	1	1	1	0	0	0	0	0	0	0
K00574	cfa; cyclopropane-fatty-acyl-phospholipid synthase [EC:2.1.1.79]	1	1	1	0	0	0	0	0	0	0
K00575	cheR; chemotaxis protein methyltransferase CheR [EC:2.1.1.80]	0	2	3	0	1	0	1	0	1	1
K00584	mtrH; tetrahydromethanopterin S-methyltransferase subunit H [EC:2.1.1.86]	1	0	3	0	0	0	0	0	0	0
K00588	E2.1.1.104; caffeoyl-CoA O-methyltransferase [EC:2.1.1.104]	1	1	1	0	0	0	0	0	0	0
K00590	E2.1.1.113; site-specific DNA-methyltransferase (cytosine-N4-specific) [EC:2.1.1.113]	0	0	2	0	0	0	0	0	0	0
K00595	cobL-cbiET; precorrin-6B C5,15-methyltransferase / cobalt-precorrin-6B C5,C15-methyltransferase [EC:2.1.1.132 2.1.1.289 2.1.1.196]	0	0	1	1	1	0	1	0	1	1
K00600	glyA, SHMT; glycine hydroxymethyltransferase [EC:2.1.2.1]	1	1	2	0	1	1	0	1	1	1
K00602	purH; phosphoribosylaminoimidazolecarboxamide formyltransferase / IMP cyclohydrolase [EC:2.1.2.3 3.5.4.10]	1	1	1	1	1	1	1	1	2	2
K00603	fctD; glutamate formiminotransferase / 5-formyltetrahydrofolate cyclo-ligase [EC:2.1.2.5 6.3.3.2]	0	0	1	0	0	0	0	1	1	1
K00604	MTFMT, fmt; methionyl-tRNA formyltransferase [EC:2.1.2.9]	1	1	1	1	1	1	1	1	1	1
K00605	gcvT, AMT; aminomethyltransferase [EC:2.1.2.10]	1	1	1	0	0	0	0	1	1	1
K00606	panB; 3-methyl-2-oxobutanoate hydroxymethyltransferase [EC:2.1.2.11]	1	0	8	0	0	1	1	1	1	1
K00609	pyrB, PYR2; aspartate carbamoyltransferase catalytic subunit [EC:2.1.3.2]	1	1	1	1	1	1	1	1	1	1
K00611	OTC, argF, argI; ornithine carbamoyltransferase [EC:2.1.3.3]	1	1	1	1	1	1	1	1	1	1
K00615	E2.2.1.1, tktA, tktB; transketolase [EC:2.2.1.1]	2	2	2	2	2	2	1	2	2	2
K00616	E2.2.1.2, talA, talB; transaldolase [EC:2.2.1.2]	1	1	1	1	1	1	0	0	0	0
K00619	argA; amino-acid N-acetyltransferase [EC:2.3.1.1]	1	0	1	0	0	1	1	1	0	0
K00620	argJ; glutamate N-acetyltransferase / amino-acid N-acetyltransferase [EC:2.3.1.35 2.3.1.1]	1	1	1	1	1	1	1	1	1	1
K00621	GNPNAT1, GNA1; glucosamine-phosphate N-acetyltransferase [EC:2.3.1.4]	0	0	1	0	0	0	0	0	0	0
K00625	pta; phosphate acetyltransferase [EC:2.3.1.8]	0	0	0	0	0	0	0	1	0	0
K00626	ACAT, atoB; acetyl-CoA C-acetyltransferase [EC:2.3.1.9]	0	0	6	0	0	0	0	3	0	0
K00634	ptb; phosphate butyryltransferase [EC:2.3.1.19]	0	0	1	0	0	0	0	1	0	0
K00638	catB; chloramphenicol O-acetyltransferase type B [EC:2.3.1.28]	0	0	0	0	0	0	0	0	0	1
K00639	kbl, GCAT; glycine C-acetyltransferase [EC:2.3.1.29]	0	0	2	0	0	0	0	0	0	0
K00640	cysE; serine O-acetyltransferase [EC:2.3.1.30]	1	1	1	1	1	1	0	1	1	1
K00645	fabD, MCAT, MCT1; [acyl-carrier-protein] S-malonyltransferase [EC:2.3.1.39]	1	1	1	0	1	1	0	1	1	1
K00648	fabH; 3-oxoacyl-[acyl-carrier-protein] synthase III [EC:2.3.1.180]	3	2	1	0	0	1	1	1	1	1
K00651	metA; homoserine O-succinyltransferase/O-acetyltransferase [EC:2.3.1.46 2.3.1.31]	1	1	1	1	1	0	0	1	0	1
K00655	plsC; 1-acyl-sn-glycerol-3-phosphate acyltransferase [EC:2.3.1.51]	1	1	1	1	1	1	1	1	1	1
K00656	E2.3.1.54, pflD; formate C-acetyltransferase [EC:2.3.1.54]	4	2	2	0	0	1	1	0	0	0
K00657	speG, SAT; diamine N-acetyltransferase [EC:2.3.1.57]	0	0	1	0	1	0	0	0	0	0
K00661	maa; maltose O-acetyltransferase [EC:2.3.1.79]	2	1	0	0	1	0	0	1	0	0
K00666	K00666; fatty-acyl-CoA synthase [EC:6.2.1.-]	3	2	4	0	1	1	1	1	1	1
K00672	ftr; formylmethanofuran--tetrahydromethanopterin N-formyltransferase [EC:2.3.1.101]	0	0	2	0	0	0	0	0	0	0
K00686	tgl; protein-glutamine gamma-glutamyltransferase [EC:2.3.2.13]	0	0	0	0	0	0	0	1	1	1
K00688	PYG, glgP; glycogen phosphorylase [EC:2.4.1.1]	3	2	2	0	0	0	0	0	0	0
K00700	GBE1, glgB; 1,4-alpha-glucan branching enzyme [EC:2.4.1.18]	1	1	1	0	0	0	0	0	0	0
K00703	glgA; starch synthase [EC:2.4.1.21]	1	1	1	0	0	0	0	0	0	0
K00705	malQ; 4-alpha-glucanotransferase [EC:2.4.1.25]	1	1	1	0	0	0	0	0	0	0
K00721	DPM1; dolichol-phosphate mannosyltransferase [EC:2.4.1.83]	0	0	1	1	3	1	1	1	2	2
K00756	pdp; pyrimidine-nucleoside phosphorylase [EC:2.4.2.2]	1	1	1	1	1	1	1	1	1	1
K00759	APRT, apt; adenine phosphoribosyltransferase [EC:2.4.2.7]	0	0	0	0	0	0	0	0	1	1
K00760	hprT, hpt, HPRT1; hypoxanthine phosphoribosyltransferase [EC:2.4.2.8]	2	1	1	1	1	1	1	1	1	1
K00761	upp, UPRT; uracil phosphoribosyltransferase [EC:2.4.2.9]	1	1	1	1	1	0	0	0	0	0
K00762	pyrE; orotate phosphoribosyltransferase [EC:2.4.2.10]	1	1	2	1	1	1	1	1	1	1
K00763	pncB, NAPRT1; nicotinate phosphoribosyltransferase [EC:6.3.4.21]	1	1	1	1	1	0	0	0	1	0
K00764	purF, PPAT; amidophosphoribosyltransferase [EC:2.4.2.14]	2	0	2	1	1	2	1	2	1	1
K00765	hisG; ATP phosphoribosyltransferase [EC:2.4.2.17]	1	0	0	1	1	1	0	1	1	1
K00766	trpD; anthranilate phosphoribosyltransferase [EC:2.4.2.18]	2	0	2	1	2	1	0	0	1	1
K00767	nadC, QPRT; nicotinate-nucleotide pyrophosphorylase (carboxylating) [EC:2.4.2.19]	0	0	1	0	1	1	0	1	1	2
K00768	E2.4.2.21, cobU, cobT; nicotinate-nucleotide--dimethylbenzimidazole phosphoribosyltransferase [EC:2.4.2.21]	2	1	1	1	1	0	0	0	0	0
K00772	mtaP, MTAP; 5'-methylthioadenosine phosphorylase [EC:2.4.2.28]	1	1	1	1	1	0	1	1	2	2
K00773	tgt; queuine tRNA-ribosyltransferase [EC:2.4.2.29]	1	0	1	1	1	1	1	1	1	1
K00782	lldG; L-lactate dehydrogenase complex protein LldG	0	0	0	0	0	1	0	0	0	0
K00783	rlmH; 23S rRNA (pseudouridine1915-N3)-methyltransferase [EC:2.1.1.177]	1	1	1	1	1	1	1	1	1	1
K00784	rnz; ribonuclease Z [EC:3.1.26.11]	2	1	1	1	1	0	1	1	1	1
K00786	GALT29A; beta-1,6-galactosyltransferase [EC:2.4.1.-]	1	0	0	0	0	0	0	1	2	1
K00788	thiE; thiamine-phosphate pyrophosphorylase [EC:2.5.1.3]	1	0	1	2	1	1	1	0	1	0
K00789	metK, MAT; S-adenosylmethionine synthetase [EC:2.5.1.6]	3	1	1	1	1	1	0	1	1	1
K00790	murA; UDP-N-acetylglucosamine 1-carboxyvinyltransferase [EC:2.5.1.7]	3	3	3	2	2	2	2	2	2	2
K00791	miaA, TRIT1; tRNA dimethylallyltransferase [EC:2.5.1.75]	1	1	1	1	1	1	1	1	1	1
K00793	ribE, RIB5; riboflavin synthase [EC:2.5.1.9]	1	0	1	1	1	1	1	0	1	1
K00794	ribH, RIB4; 6,7-dimethyl-8-ribityllumazine synthase [EC:2.5.1.78]	1	0	1	1	1	1	0	0	1	1
K00796	folP; dihydropteroate synthase [EC:2.5.1.15]	1	1	2	1	1	1	1	1	1	1
K00797	speE, SRM, SPE3; spermidine synthase [EC:2.5.1.16]	1	1	3	1	1	1	0	1	1	1
K00798	MMAB, pduO; cob(I)alamin adenosyltransferase [EC:2.5.1.17]	0	1	1	0	0	0	0	0	0	0
K00800	aroA; 3-phosphoshikimate 1-carboxyvinyltransferase [EC:2.5.1.19]	1	1	1	1	1	1	1	1	1	1
K00805	hepS; heptaprenyl diphosphate synthase component 1 [EC:2.5.1.30]	3	2	3	1	2	1	1	1	0	1
K00806	uppS; undecaprenyl diphosphate synthase [EC:2.5.1.31]	1	1	1	1	1	1	1	2	1	1
K00812	aspB; aspartate aminotransferase [EC:2.6.1.1]	0	0	2	0	0	0	0	0	0	0
K00817	hisC; histidinol-phosphate aminotransferase [EC:2.6.1.9]	1	1	5	2	2	1	1	1	1	1
K00819	rocD, OAT; ornithine--oxo-acid transaminase [EC:2.6.1.13]	0	0	2	0	0	0	0	0	0	0
K00820	glmS, GFPT; glutamine---fructose-6-phosphate transaminase (isomerizing) [EC:2.6.1.16]	1	1	1	1	1	1	1	1	1	1
K00821	argD; acetylornithine/N-succinyldiaminopimelate aminotransferase [EC:2.6.1.11 2.6.1.17]	1	1	1	1	1	1	1	1	1	1
K00823	puuE; 4-aminobutyrate aminotransferase [EC:2.6.1.19]	0	0	1	0	0	0	0	0	0	0
K00826	E2.6.1.42, ilvE; branched-chain amino acid aminotransferase [EC:2.6.1.42]	1	1	2	1	1	1	1	1	1	1
K00830	AGXT; alanine-glyoxylate transaminase / serine-glyoxylate transaminase / serine-pyruvate transaminase [EC:2.6.1.44 2.6.1.45 2.6.1.51]	0	0	0	1	1	0	0	0	0	0
K00831	serC, PSAT1; phosphoserine aminotransferase [EC:2.6.1.52]	1	1	1	1	1	1	0	0	0	0
K00832	tyrB; aromatic-amino-acid transaminase [EC:2.6.1.57]	1	1	1	0	0	0	0	0	0	0
K00833	bioA; adenosylmethionine---8-amino-7-oxononanoate aminotransferase [EC:2.6.1.62]	0	0	0	0	1	0	0	0	0	0
K00844	HK; hexokinase [EC:2.7.1.1]	1	1	1	0	0	0	0	0	0	0
K00850	pfkA, PFK; 6-phosphofructokinase 1 [EC:2.7.1.11]	1	1	1	0	0	0	0	0	0	0
K00852	rbsK, RBKS; ribokinase [EC:2.7.1.15]	0	1	1	0	0	0	1	0	0	0
K00854	xylB, XYLB; xylulokinase [EC:2.7.1.17]	1	0	2	0	0	0	0	0	0	0
K00858	ppnK, NADK; NAD+ kinase [EC:2.7.1.23]	1	1	2	1	1	1	1	1	1	1
K00859	coaE; dephospho-CoA kinase [EC:2.7.1.24]	1	1	1	1	1	1	1	1	1	1
K00864	glpK, GK; glycerol kinase [EC:2.7.1.30]	0	0	1	1	1	1	1	0	0	0
K00865	glxK, garK; glycerate 2-kinase [EC:2.7.1.165]	0	0	1	0	0	0	0	0	0	0
K00868	pdxK, pdxY; pyridoxine kinase [EC:2.7.1.35]	2	1	1	1	1	1	1	0	1	1
K00872	thrB; homoserine kinase [EC:2.7.1.39]	1	1	1	0	0	1	1	1	0	0
K00873	PK, pyk; pyruvate kinase [EC:2.7.1.40]	1	1	2	1	0	0	0	0	1	1
K00876	udk, UCK; uridine kinase [EC:2.7.1.48]	3	1	1	1	1	1	1	1	1	1
K00878	thiM; hydroxyethylthiazole kinase [EC:2.7.1.50]	1	0	1	0	1	1	1	0	1	0
K00887	dgkA; undecaprenol kinase [EC:2.7.1.66]	1	0	0	0	1	1	1	1	1	1
K00891	aroK, aroL; shikimate kinase [EC:2.7.1.71]	1	1	2	3	3	1	1	2	1	1
K00901	dgkA, DGK; diacylglycerol kinase (ATP) [EC:2.7.1.107]	0	1	1	1	0	0	0	0	0	0
K00903	epsB, capB; protein-tyrosine kinase [EC:2.7.10.3]	1	1	1	0	0	1	0	0	0	0
K00919	ispE; 4-diphosphocytidyl-2-C-methyl-D-erythritol kinase [EC:2.7.1.148]	1	1	1	1	1	1	1	1	1	1
K00925	ackA; acetate kinase [EC:2.7.2.1]	1	1	1	1	1	2	1	1	1	1
K00926	arcC; carbamate kinase [EC:2.7.2.2]	0	1	1	0	1	0	1	0	0	0
K00927	PGK, pgk; phosphoglycerate kinase [EC:2.7.2.3]	1	1	1	1	1	1	0	1	1	1
K00928	lysC; aspartate kinase [EC:2.7.2.4]	2	2	2	1	1	1	2	1	1	1
K00929	buk; butyrate kinase [EC:2.7.2.7]	0	0	3	0	0	0	1	1	0	0
K00930	argB; acetylglutamate kinase [EC:2.7.2.8]	1	1	1	1	1	1	1	1	1	1
K00931	proB; glutamate 5-kinase [EC:2.7.2.11]	1	0	1	1	1	1	1	1	1	1
K00936	pdtaS; two-component system, sensor histidine kinase PdtaS [EC:2.7.13.3]	0	0	2	0	0	0	0	0	0	0

K00937	ppk1; polyphosphate kinase [EC:2.7.4.1]	1	0	1	0	0	0	0	1	0	0
K00939	adk, AK; adenylate kinase [EC:2.7.4.3]	1	1	1	1	1	1	1	1	1	1
K00940	ndk, NME; nucleoside-diphosphate kinase [EC:2.7.4.6]	1	1	1	0	1	0	0	0	1	1
K00941	thiD; hydroxymethylpyrimidine/phosphomethylpyrimidine kinase [EC:2.7.1.49 2.7.4.7]	1	0	1	1	1	1	1	0	1	0
K00942	gmk, GUK1; guanylate kinase [EC:2.7.4.8]	1	1	1	1	1	1	1	1	1	1
K00943	tmk, DTYMK; dTMP kinase [EC:2.7.4.9]	1	1	1	1	1	1	1	1	1	1
K00945	cmk; CMP/dCMP kinase [EC:2.7.4.25]	1	1	1	2	2	1	1	1	1	1
K00948	PRPS, prsA; ribose-phosphate pyrophosphokinase [EC:2.7.6.1]	1	1	1	1	1	1	1	1	1	1
K00949	thiN, TPK1, THI80; thiamine pyrophosphokinase [EC:2.7.6.2]	1	1	1	1	1	1	1	1	1	1
K00950	folK; 2-amino-4-hydroxy-6-hydroxymethyldihydropteridine diphosphokinase [EC:2.7.6.3]	1	1	1	1	1	1	1	1	1	1
K00951	relA; GTP pyrophosphokinase [EC:2.7.6.5]	1	1	1	1	1	1	1	1	1	1
K00954	E2.7.7.3A, coaD, kdtB; pantetheine-phosphate adenylyltransferase [EC:2.7.7.3]	1	1	1	1	1	1	1	1	1	1
K00962	pnp, PNPT1; polyribonucleotide nucleotidyltransferase [EC:2.7.7.8]	1	0	1	1	1	1	1	1	1	1
K00963	UGP2, galU, galF; UTP--glucose-1-phosphate uridylyltransferase [EC:2.7.7.9]	1	0	1	0	0	0	0	0	0	0
K00966	GMPP; mannose-1-phosphate guanylyltransferase [EC:2.7.7.13]	0	0	0	0	0	1	0	0	0	0
K00969	nadD; nicotinate-nucleotide adenylyltransferase [EC:2.7.7.18]	1	1	1	1	1	1	1	1	1	1
K00971	manC, cpsB; mannose-1-phosphate guanylyltransferase [EC:2.7.7.13]	1	1	2	1	1	1	1	0	0	0
K00973	rfbA, rmlA, rffH; glucose-1-phosphate thymidylyltransferase [EC:2.7.7.24]	2	0	2	1	2	0	1	0	0	0
K00974	cca; tRNA nucleotidyltransferase (CCA-adding enzyme) [EC:2.7.7.72 3.1.3.- 3.1.4.-]	2	1	2	1	1	1	1	1	1	1
K00975	glgC; glucose-1-phosphate adenylyltransferase [EC:2.7.7.27]	3	3	3	0	0	1	0	0	1	1
K00978	rfbF; glucose-1-phosphate cytidylyltransferase [EC:2.7.7.33]	0	0	1	0	1	0	0	0	0	0
K00981	E2.7.7.41, CDS1, CDS2, cdsA; phosphatidate cytidylyltransferase [EC:2.7.7.41]	1	0	1	1	1	1	0	1	1	1
K00986	ltrA; RNA-directed DNA polymerase [EC:2.7.7.49]	2	0	9	0	0	0	0	0	0	0
K00989	rph; ribonuclease PH [EC:2.7.7.56]	1	1	1	1	1	0	1	0	0	0
K00991	ispD; 2-C-methyl-D-erythritol 4-phosphate cytidylyltransferase [EC:2.7.7.60]	1	1	1	0	0	1	0	1	1	1
K00995	pgsA, PGS1; CDP-diacylglycerol---glycerol-3-phosphate 3-phosphatidyltransferase [EC:2.7.8.5]	1	1	3	1	1	1	1	1	1	1
K00996	rfbP; undecaprenyl-phosphate galactose phosphotransferase [EC:2.7.8.6]	1	0	0	0	0	0	0	0	0	0
K00997	acpS; holo-[acyl-carrier protein] synthase [EC:2.7.8.7]	1	1	1	1	1	0	1	0	0	0
K01000	mraY; phospho-N-acetylmuramoyl-pentapeptide-transferase [EC:2.7.8.13]	1	1	1	1	1	1	0	1	1	1
K01006	ppdK; pyruvate, orthophosphate dikinase [EC:2.7.9.1]	1	1	1	1	1	1	0	1	1	1
K01007	pps, ppsA; pyruvate, water dikinase [EC:2.7.9.2]	1	1	7	0	0	0	0	0	0	0
K01008	selD, SEPHS; selenide, water dikinase [EC:2.7.9.3]	1	1	1	0	0	1	1	1	1	1
K01012	bioB; biotin synthase [EC:2.8.1.6]	0	1	3	3	2	1	1	0	1	1
K01023	assT; arylsulfate sulfotransferase [EC:2.8.2.22]	3	0	0	0	0	0	0	0	0	0
K01034	atoD; acetate CoA/acetoacetate CoA-transferase alpha subunit [EC:2.8.3.8 2.8.3.9]	0	0	2	0	0	0	0	2	0	0
K01035	atoA; acetate CoA/acetoacetate CoA-transferase beta subunit [EC:2.8.3.8 2.8.3.9]	0	0	1	0	0	0	0	2	0	0
K01039	gctA; glutaconate CoA-transferase, subunit A [EC:2.8.3.12]	1	0	3	0	0	0	0	0	0	0
K01040	gctB; glutaconate CoA-transferase, subunit B [EC:2.8.3.12]	1	0	3	0	0	0	0	0	0	0
K01042	selA; L-seryl-tRNA(Ser) seleniumtransferase [EC:2.9.1.1]	1	1	1	0	0	1	1	1	1	1
K01046	lip, TGL2; triacylglycerol lipase [EC:3.1.1.3]	0	0	2	0	0	0	0	0	1	1
K01048	pldB; lysophospholipase [EC:3.1.1.5]	0	0	0	1	0	0	1	0	0	0
K01055	pcaD; 3-oxoadipate enol-lactonase [EC:3.1.1.24]	0	0	1	0	0	0	0	0	1	1
K01056	PTH1, PTRH1, pth, spoVC; peptidyl-tRNA hydrolase, PTH1 family [EC:3.1.1.29]	1	1	1	1	1	1	1	1	1	1
K01061	E3.1.1.45; carboxymethylenebutenolidase [EC:3.1.1.45]	0	0	1	0	0	0	0	0	0	0
K01066	aes; acetyl esterase [EC:3.1.1.-]	1	0	0	0	0	1	0	1	1	1
K01069	gloB, gloC, HAGH; hydroxyacylglutathione hydrolase [EC:3.1.2.6]	2	2	3	1	1	1	1	2	1	1
K01071	MCH; medium-chain acyl-[acyl-carrier-protein] hydrolase [EC:3.1.2.21]	0	0	1	0	0	0	0	0	0	0
K01073	E3.1.2.20; acyl-CoA hydrolase [EC:3.1.2.20]	1	0	0	0	0	0	0	0	0	0
K01077	E3.1.3.1, phoA, phoB; alkaline phosphatase [EC:3.1.3.1]	1	0	2	0	0	0	0	0	0	1
K01081	E3.1.3.5; 5'-nucleotidase [EC:3.1.3.5]	0	0	0	0	0	0	0	0	1	1
K01090	E3.1.3.16; protein phosphatase [EC:3.1.3.16]	0	0	1	0	0	0	0	0	0	0
K01091	gph; phosphoglycolate phosphatase [EC:3.1.3.18]	0	2	3	3	5	0	0	1	1	1
K01092	E3.1.3.25, IMPA, suhB; myo-inositol-1(or 4)-monophosphatase [EC:3.1.3.25]	1	0	1	0	0	0	0	0	0	0
K01096	pgpB; phosphatidylglycerophosphatase B [EC:3.1.3.27 3.6.1.75 3.1.3.4 3.6.1.27]	0	0	0	1	1	0	0	0	0	0
K01104	E3.1.3.48; protein-tyrosine phosphatase [EC:3.1.3.48]	2	2	2	2	4	2	1	1	0	0
K01114	plc; phospholipase C [EC:3.1.4.3]	0	0	1	0	0	0	0	0	0	0
K01118	acpD, azoR; FMN-dependent NADH-azoreductase [EC:1.7.1.17]	0	0	0	0	0	1	0	1	0	0
K01126	E3.1.4.46, glpQ, ugpQ; glycerophosphoryl diester phosphodiesterase [EC:3.1.4.46]	0	0	1	1	1	0	0	0	2	1
K01129	dgt; dGTPase [EC:3.1.5.1]	1	1	2	1	1	1	1	1	1	1
K01138	K01138; uncharacterized sulfatase [EC:3.1.6.-]	0	0	0	0	0	0	0	1	0	0
K01142	E3.1.11.2, xthA; exodeoxyribonuclease III [EC:3.1.11.2]	1	1	1	0	0	1	1	1	0	0
K01151	nfo; deoxyribonuclease IV [EC:3.1.21.2]	1	1	1	1	1	0	0	1	2	1
K01153	hsdR; type I restriction enzyme, R subunit [EC:3.1.21.3]	1	0	0	0	2	0	0	1	0	1
K01154	hsdS; type I restriction enzyme, S subunit [EC:3.1.21.3]	1	0	0	0	2	0	0	1	0	1
K01155	E3.1.21.4; type II restriction enzyme [EC:3.1.21.4]	1	0	0	1	0	0	0	0	0	0
K01159	ruvC; crossover junction endodeoxyribonuclease RuvC [EC:3.1.21.10]	1	1	1	1	1	1	1	1	1	1
K01160	rusA; crossover junction endodeoxyribonuclease RusA [EC:3.1.21.10]	0	0	0	0	0	0	0	0	0	1
K01163	K01163; uncharacterized protein	1	0	0	0	0	1	1	1	1	1
K01200	pulA; pullulanase [EC:3.2.1.41]	0	0	1	0	0	0	0	0	0	0
K01207	nagZ; beta-N-acetylhexosaminidase [EC:3.2.1.52]	0	1	1	0	0	0	0	0	1	1
K01239	E3.2.2.1; purine nucleosidase [EC:3.2.2.1]	0	0	1	0	0	0	0	0	0	0
K01246	tag; DNA-3-methyladenine glycosylase I [EC:3.2.2.20]	0	0	1	0	0	0	0	0	0	0
K01247	alkA; DNA-3-methyladenine glycosylase II [EC:3.2.2.21]	1	0	0	0	0	0	0	0	0	0
K01251	AHCY, ahcY; adenosylhomocysteinase [EC:3.13.2.1]	1	1	1	1	1	0	0	0	0	0
K01259	pip; proline iminopeptidase [EC:3.4.11.5]	0	0	0	0	1	0	0	0	0	0
K01262	pepP; Xaa-Pro aminopeptidase [EC:3.4.11.9]	1	1	1	1	1	0	1	0	1	1
K01265	map; methionyl aminopeptidase [EC:3.4.11.18]	2	1	2	1	1	1	1	1	1	1
K01266	dmpA, dap; D-aminopeptidase [EC:3.4.11.19]	0	0	0	1	1	0	0	0	0	0
K01269	yhfE; aminopeptidase [EC:3.4.11.-]	0	0	1	0	0	0	0	0	0	0
K01271	pepQ; Xaa-Pro dipeptidase [EC:3.4.13.9]	0	1	5	0	1	1	0	2	1	1
K01273	DPEP; membrane dipeptidase [EC:3.4.13.19]	1	1	2	1	1	1	1	1	1	1
K01297	ldcA; muramoyltetrapeptide carboxypeptidase [EC:3.4.17.13]	0	0	0	0	0	0	0	0	1	0
K01305	iadA; beta-aspartyl-dipeptidase (metallo-type) [EC:3.4.19.-]	1	1	1	0	0	0	0	0	0	0
K01308	yqgT; g-D-glutamyl-meso-diaminopimelate peptidase [EC:3.4.19.11]	0	0	0	0	0	1	0	0	0	0
K01338	lon; ATP-dependent Lon protease [EC:3.4.21.53]	3	2	2	2	2	2	1	1	1	1
K01356	lexA; repressor LexA [EC:3.4.21.88]	2	0	4	2	2	2	2	1	0	0
K01358	clpP, CLPP; ATP-dependent Clp protease, protease subunit [EC:3.4.21.92]	1	1	2	1	2	1	1	1	1	2
K01409	OSGEP, KAE1, QRI7; N6-L-threonylcarbamoyladenine synthase [EC:2.3.1.234]	2	2	2	2	2	1	2	2	1	1
K01419	hslV, clpQ; ATP-dependent HslUV protease, peptidase subunit HslV [EC:3.4.25.2]	1	1	1	1	1	1	1	1	1	1
K01420	fnr; CRP/FNR family transcriptional regulator, anaerobic regulatory protein	2	0	0	0	1	0	0	1	1	1
K01421	yhgE; putative membrane protein	0	0	0	2	1	0	0	0	0	0
K01424	E3.5.1.1, ansA, ansB; L-asparaginase [EC:3.5.1.1]	0	1	1	0	1	0	0	1	1	1
K01436	yhaA; amidohydrolase [EC:3.5.1.-]	1	1	2	0	0	0	1	1	0	0
K01438	argE; acetylornithine deacetylase [EC:3.5.1.16]	0	0	5	0	0	0	0	0	0	0
K01439	dapE; succinyl-diaminopimelate desuccinylase [EC:3.5.1.18]	2	0	4	0	0	1	1	1	1	1
K01442	cbh; choloylglycine hydrolase [EC:3.5.1.24]	0	0	1	0	0	0	0	0	0	0
K01443	nagA, AMDHD2; N-acetylglucosamine-6-phosphate deacetylase [EC:3.5.1.25]	0	0	1	0	0	0	0	0	0	0
K01448	amiABC; N-acetylmuramoyl-L-alanine amidase [EC:3.5.1.28]	3	3	6	1	1	2	1	2	1	1
K01449	cwlJ, sleB; N-acetylmuramoyl-L-alanine amidase [EC:3.5.1.28]	2	2	2	1	1	2	0	2	2	2
K01452	E3.5.1.41; chitin deacetylase [EC:3.5.1.41]	0	0	1	0	0	0	0	0	0	0
K01462	PDF, def; peptide deformylase [EC:3.5.1.88]	1	1	2	1	1	1	1	1	1	1
K01464	DPYS, dht, hydA; dihydropyrimidinase [EC:3.5.2.2]	0	1	3	0	0	0	0	1	0	0
K01465	URA4, pyrC; dihydroorotase [EC:3.5.2.3]	1	1	1	1	1	0	0	0	0	0
K01466	allB; allantoinase [EC:3.5.2.5]	0	0	1	0	0	1	0	1	1	1
K01468	hutI, AMDHD1; imidazolonepropionase [EC:3.5.2.7]	1	1	2	0	0	0	0	0	0	0
K01470	E3.5.2.10; creatinine amidohydrolase [EC:3.5.2.10]	0	0	1	0	0	0	0	2	0	0
K01473	hyuA; N-methylhydantoinase A [EC:3.5.2.14]	0	2	4	0	2	0	0	0	0	0
K01474	hyuB; N-methylhydantoinase B [EC:3.5.2.14]	0	2	4	0	2	0	0	0	0	0
K01476	E3.5.3.1, rocF, arg; arginase [EC:3.5.3.1]	1	0	0	0	0	0	0	0	0	0
K01478	arcA; arginine deiminase [EC:3.5.3.6]	0	0	2	0	1	0	0	0	0	0
K01480	speB; agmatinase [EC:3.5.3.11]	1	0	1	0	2	1	0	1	1	1
K01482	DDAH, ddaH; dimethylargininase [EC:3.5.3.18]	0	0	1	0	0	0	0	0	0	0
K01485	codA; cytosine/creatinine deaminase [EC:3.5.4.1 3.5.4.21]	0	0	0	0	0	0	0	1	0	0
K01486	ade; adenine deaminase [EC:3.5.4.2]	1	0	1	1	1	0	0	1	0	0
K01487	guaD, GDA; guanine deaminase [EC:3.5.4.3]	2	0	1	0	0	0	1	1	0	0
K01489	cdd, CDA; cytidine deaminase [EC:3.5.4.5]	1	1	1	1	1	1	0	1	1	1
K01491	folD; methylenetetrahydrofolate dehydrogenase (NADP+) / methenyltetrahydrofolate cyclohydrolase [EC:1.5.1.5 3.5.4.9]	1	1	1	1	1	1	1	1	1	1
K01493	comEB; dCMP deaminase [EC:3.5.4.12]	1	1	1	1	1	1	1	1	1	1
K01495	GCH1, folE; GTP cyclohydrolase IA [EC:3.5.4.16]	1	1	2	1	1	1	1	1	1	1
K01496	hisI; phosphoribosyl-AMP cyclohydrolase [EC:3.5.4.19]	0	0	0	0	0	1	0	0	0	0
K01499	mch; methenyltetrahydromethanopterin cyclohydrolase [EC:3.5.4.27]	1	0	2	0	0	0	0	0	0	0
K01501	E3.5.5.1; nitrilase [EC:3.5.5.1]	0	0	1	1	0	0	0	1	0	0
K01507	ppa; inorganic pyrophosphatase [EC:3.6.1.1]	0	0	1	1	1	0	0	1	0	0
K01515	nudF; ADP-ribose pyrophosphatase [EC:3.6.1.13 3.6.1.-]	1	1	1	0	0	0	0	0	0	0
K01520	dut, DUT; dUTP pyrophosphatase [EC:3.6.1.23]	1	1	1	1	1	1	1	1	1	1
K01523	hisE; phosphoribosyl-ATP pyrophosphohydrolase [EC:3.6.1.31]	1	0	1	0	0	1	0	0	0	0

K01524	ppx-gppA; exopolyphosphatase / guanosine-5'-triphosphate,3'-diphosphate pyrophosphatase [EC:3.6.1.11 3.6.1.40]	2	1	3	1	1	1	1	2	1	1
K01531	mgtA, mgtB; P-type Mg2+ transporter [EC:7.2.2.14]	1	0	0	0	2	0	0	0	0	0
K01533	copB; P-type Cu2+ transporter [EC:7.2.2.9]	1	0	0	1	0	0	0	0	0	0
K01534	zntA; Zn2+/Cd2+-exporting ATPase [EC:7.2.2.12 7.2.2.21]	3	2	3	4	1	0	0	1	1	1
K01537	ATP2C; P-type Ca2+ transporter type 2C [EC:7.2.2.10]	1	1	3	1	3	2	0	2	2	3
K01546	kdpA; potassium-transporting ATPase potassium-binding subunit	1	0	1	0	0	0	0	0	0	0
K01547	kdpB; potassium-transporting ATPase ATP-binding subunit [EC:7.2.2.6]	1	0	1	0	0	0	0	0	0	0
K01548	kdpC; potassium-transporting ATPase KdpC subunit	1	0	1	0	0	0	0	0	0	0
K01551	arsA, ASNA1, GET3; arsenite/tail-anchored protein-transporting ATPase [EC:7.3.2.7 7.3.-.-]	0	0	1	0	0	0	0	0	0	0
K01560	E3.8.1.2; 2-haloacid dehalogenase [EC:3.8.1.2]	1	0	0	0	0	0	0	0	0	0
K01571	oadA; oxaloacetate decarboxylase (Na+ extruding) subunit alpha [EC:7.2.4.2]	1	1	1	0	0	1	1	1	1	1
K01575	alsD, budA, aldC; acetolactate decarboxylase [EC:4.1.1.5]	0	0	1	0	0	0	0	1	1	1
K01579	panD; aspartate 1-decarboxylase [EC:4.1.1.11]	1	0	1	0	0	1	1	1	1	1
K01582	E4.1.1.18, ldcC, cadA; lysine decarboxylase [EC:4.1.1.18]	0	0	0	0	0	0	0	1	0	0
K01585	speA; arginine decarboxylase [EC:4.1.1.19]	2	1	2	1	1	2	0	1	2	2
K01586	lysA; diaminopimelate decarboxylase [EC:4.1.1.20]	1	1	1	1	1	1	1	1	1	1
K01588	purE; 5-(carboxyamino)imidazole ribonucleotide mutase [EC:5.4.99.18]	1	1	1	1	1	1	1	1	1	1
K01591	pyrF; orotidine-5'-phosphate decarboxylase [EC:4.1.1.23]	1	1	1	1	1	1	1	1	1	1
K01596	E4.1.1.32, pckA, PCK; phosphoenolpyruvate carboxykinase (GTP) [EC:4.1.1.32]	0	0	0	0	0	0	0	0	1	1
K01599	hemE, UROD; uroporphyrinogen decarboxylase [EC:4.1.1.37]	6	3	10	0	4	0	1	0	0	0
K01607	pcaC; 4-carboxymuconolactone decarboxylase [EC:4.1.1.44]	1	0	0	1	0	0	0	0	0	0
K01609	trpC; indole-3-glycerol phosphate synthase [EC:4.1.1.48]	1	0	1	0	1	1	0	0	1	1
K01610	E4.1.1.49, pckA; phosphoenolpyruvate carboxykinase (ATP) [EC:4.1.1.49]	0	0	2	0	0	1	1	1	0	0
K01611	speD, AMD1; S-adenosylmethionine decarboxylase [EC:4.1.1.50]	1	1	1	1	1	1	0	1	1	1
K01613	psd, PISD; phosphatidylserine decarboxylase [EC:4.1.1.65]	1	1	1	0	0	0	0	0	0	0
K01615	gcdA; glutaconyl-CoA decarboxylase subunit alpha [EC:7.2.4.5]	0	0	1	0	0	0	0	0	0	0
K01619	deoC, DERA; deoxyribose-phosphate aldolase [EC:4.1.2.4]	1	1	1	1	1	0	0	1	1	1
K01620	ltaE; threonine aldolase [EC:4.1.2.48]	0	1	2	1	1	0	0	0	0	0
K01621	xfp, xpk; xylulose-5-phosphate/fructose-6-phosphate phosphoketolase [EC:4.1.2.9 4.1.2.22]	0	0	0	0	0	1	0	0	1	0
K01622	K01622; fructose 1,6-bisphosphate aldolase/phosphatase [EC:4.1.2.13 3.1.3.11]	0	1	1	0	0	0	0	0	0	0
K01624	FBA, fbaA; fructose-bisphosphate aldolase, class II [EC:4.1.2.13]	1	1	1	1	1	1	1	0	1	1
K01626	E2.5.1.54, aroF, aroG, aroH; 3-deoxy-7-phosphoheptulonate synthase [EC:2.5.1.54]	1	0	0	0	1	0	0	0	0	0
K01628	fucA; L-fuculose-phosphate aldolase [EC:4.1.2.17]	1	1	3	1	1	1	0	1	1	1
K01633	folB; 7,8-dihydroneopterin aldolase/epimerase/oxygenase [EC:4.1.2.25 5.1.99.8 1.13.11.81]	1	1	1	1	1	1	1	1	1	1
K01640	HMGCL, hmgL; hydroxymethylglutaryl-CoA lyase [EC:4.1.3.4]	0	0	1	0	0	0	0	0	0	0
K01643	citF; citrate lyase subunit alpha / citrate CoA-transferase [EC:2.8.3.10]	0	0	3	0	0	0	0	0	0	0
K01644	citE; citrate lyase subunit beta / citryl-CoA lyase [EC:4.1.3.34]	0	0	4	0	0	0	0	0	0	0
K01646	citD; citrate lyase subunit gamma (acyl carrier protein)	0	0	3	0	0	0	0	0	0	0
K01647	CS, gltA; citrate synthase [EC:2.3.3.1]	1	0	0	0	0	0	0	0	0	0
K01649	leuA, IMS; 2-isopropylmalate synthase [EC:2.3.3.13]	2	2	3	1	2	3	1	2	1	2
K01652	E2.2.1.6L, ilvB, ilvG, ilvI; acetolactate synthase I/II/III large subunit [EC:2.2.1.6]	2	2	4	1	3	2	1	1	2	2
K01653	E2.2.1.6S, ilvH, ilvN; acetolactate synthase I/III small subunit [EC:2.2.1.6]	2	2	2	1	2	1	2	2	2	2
K01657	trpE; anthranilate synthase component I [EC:4.1.3.27]	1	0	1	0	1	1	0	0	0	0
K01658	trpG; anthranilate synthase component II [EC:4.1.3.27]	1	0	2	0	1	1	0	0	0	0
K01661	menB; naphthoate synthase [EC:4.1.3.36]	0	0	1	0	0	0	0	0	0	0
K01662	dxs; 1-deoxy-D-xylulose-5-phosphate synthase [EC:2.2.1.7]	1	1	1	1	1	1	1	1	1	1
K01664	pabA; para-aminobenzoate synthetase component II [EC:2.6.1.85]	1	0	0	1	1	0	0	0	0	0
K01665	pabB; para-aminobenzoate synthetase component I [EC:2.6.1.85]	1	0	1	1	1	0	0	0	0	0
K01673	cynT, can; carbonic anhydrase [EC:4.2.1.1]	1	0	0	0	0	0	0	1	0	0
K01677	E4.2.1.2AA, fumA; fumarate hydratase subunit alpha [EC:4.2.1.2]	1	1	5	1	1	2	0	1	1	1
K01678	E4.2.1.2AB, fumB; fumarate hydratase subunit beta [EC:4.2.1.2]	1	1	5	1	1	2	1	1	1	1
K01681	ACO, acnA; aconitate hydratase [EC:4.2.1.3]	1	1	6	0	0	1	0	1	0	0
K01683	araD; D-arabinonate dehydratase [EC:4.2.1.5]	0	0	1	0	0	0	0	1	0	0
K01684	dgoD; galactonate dehydratase [EC:4.2.1.6]	0	0	0	0	0	0	1	0	0	0
K01687	ilvD; dihydroxy-acid dehydratase [EC:4.2.1.9]	1	1	3	0	1	1	1	1	1	1
K01689	ENO, eno; enolase [EC:4.2.1.11]	1	1	2	1	1	1	0	1	1	1
K01692	paaF, echA; enoyl-CoA hydratase [EC:4.2.1.17]	0	0	1	0	0	0	0	0	0	0
K01693	hisB; imidazoleglycerol-phosphate dehydratase [EC:4.2.1.19]	1	1	4	1	1	1	1	1	1	1
K01695	trpA; tryptophan synthase alpha chain [EC:4.2.1.20]	1	0	1	0	1	1	1	0	1	1
K01696	trpB; tryptophan synthase beta chain [EC:4.2.1.20]	1	0	1	0	1	1	1	0	1	2
K01698	hemB, ALAD; porphobilinogen synthase [EC:4.2.1.24]	1	1	1	1	1	0	1	0	0	0
K01699	pduC; propanediol dehydratase large subunit [EC:4.2.1.28]	0	1	1	0	0	0	0	0	0	0
K01703	leuC, IPMI-L; 3-isopropylmalate/(R)-2-methylmalate dehydratase large subunit [EC:4.2.1.33 4.2.1.35]	1	1	5	0	1	1	1	2	1	1
K01704	leuD, IPMI-S; 3-isopropylmalate/(R)-2-methylmalate dehydratase small subunit [EC:4.2.1.33 4.2.1.35]	1	1	5	0	1	1	1	2	1	1
K01709	rfbG; CDP-glucose 4,6-dehydratase [EC:4.2.1.45]	0	0	1	0	1	0	0	0	0	0
K01710	rfbB, rmlB, rffG; dTDP-glucose 4,6-dehydratase [EC:4.2.1.46]	2	1	4	0	1	0	0	1	0	0
K01712	hutU, UROC1; urocanate hydratase [EC:4.2.1.49]	0	0	1	0	0	0	0	0	0	0
K01714	dapA; 4-hydroxy-tetrahydrodipicolinate synthase [EC:4.3.3.7]	2	2	5	1	1	1	0	1	1	1
K01715	crt; enoyl-CoA hydratase [EC:4.2.1.17]	1	0	5	0	0	0	0	0	0	0
K01720	prpD; 2-methylcitrate dehydratase [EC:4.2.1.79]	0	1	2	0	0	0	0	0	0	0
K01733	thrC; threonine synthase [EC:4.2.3.1]	1	1	2	1	1	1	1	0	1	1
K01734	mgsA; methylglyoxal synthase [EC:4.2.3.3]	0	0	0	0	0	0	0	1	0	0
K01735	aroB; 3-dehydroquinate synthase [EC:4.2.3.4]	1	1	1	1	1	1	1	1	1	1
K01736	aroC; chorismate synthase [EC:4.2.3.5]	1	1	1	2	1	1	1	1	1	1
K01737	queD, ptpS, PTS; 6-pyruvoyltetrahydropterin/6-carboxytetrahydropterin synthase [EC:4.2.3.12 4.1.2.50]	1	1	1	0	0	0	0	0	0	0
K01738	cysK; cysteine synthase [EC:2.5.1.47]	2	1	3	1	2	1	0	2	2	2
K01739	metB; cystathionine gamma-synthase [EC:2.5.1.48]	0	0	1	0	0	0	0	0	0	0
K01740	metY; O-acetylhomoserine (thiol)-lyase [EC:2.5.1.49]	1	1	2	1	2	0	0	2	0	1
K01744	aspA; aspartate ammonia-lyase [EC:4.3.1.1]	0	1	2	0	0	0	1	0	0	0
K01745	hutH, HAL; histidine ammonia-lyase [EC:4.3.1.3]	0	0	1	0	0	0	0	0	0	0
K01746	E4.3.1.4; formiminotetrahydrofolate cyclodeaminase [EC:4.3.1.4]	1	1	2	1	1	1	1	1	1	1
K01749	hemC, HMBS; hydroxymethylbilane synthase [EC:2.5.1.61]	1	0	1	1	1	0	1	0	0	0
K01750	E4.3.1.12, ocd; ornithine cyclodeaminase [EC:4.3.1.12]	1	1	1	0	0	0	0	0	0	0
K01752	E4.3.1.17, sdaA, sdaB, tdcG; L-serine dehydratase [EC:4.3.1.17]	3	2	3	1	0	0	1	0	3	3
K01753	dsdA; D-serine dehydratase [EC:4.3.1.18]	0	0	1	0	0	0	0	0	0	0
K01754	E4.3.1.19, ilvA, tdcB; threonine dehydratase [EC:4.3.1.19]	0	1	0	0	1	0	1	1	0	0
K01755	argH, ASL; argininosuccinate lyase [EC:4.3.2.1]	1	1	1	1	1	1	1	1	1	1
K01756	purB, ADSL; adenylosuccinate lyase [EC:4.3.2.2]	1	1	2	1	1	1	2	1	1	1
K01758	CTH; cystathionine gamma-lyase [EC:4.4.1.1]	1	1	1	1	1	1	1	1	1	1
K01759	GLO1, gloA; lactoylglutathione lyase [EC:4.4.1.5]	2	0	2	1	1	0	0	1	1	1
K01760	metC; cysteine-S-conjugate beta-lyase [EC:4.4.1.13]	0	0	1	0	0	0	0	0	0	0
K01761	E4.4.1.11; methionine-gamma-lyase [EC:4.4.1.11]	0	0	1	0	0	0	0	0	0	0
K01770	ispF; 2-C-methyl-D-erythritol 2,4-cyclodiphosphate synthase [EC:4.6.1.12]	1	1	0	1	1	1	1	1	1	1
K01772	hemH, FECH; protoporphyrin/coproporphyrin ferrochelatase [EC:4.98.1.1 4.99.1.9]	1	1	1	0	0	0	0	0	0	0
K01775	alr; alanine racemase [EC:5.1.1.1]	2	2	2	1	1	2	2	2	2	2
K01776	murI; glutamate racemase [EC:5.1.1.3]	0	1	1	1	1	1	1	1	1	1
K01777	prdF; proline racemase [EC:5.1.1.4]	1	1	3	0	0	0	0	0	0	0
K01778	dapF; diaminopimelate epimerase [EC:5.1.1.7]	1	1	1	1	1	1	1	1	1	1
K01783	rpe, RPE; ribulose-phosphate 3-epimerase [EC:5.1.3.1]	1	1	1	1	1	1	1	1	1	1
K01784	galE, GALE; UDP-glucose 4-epimerase [EC:5.1.3.2]	1	2	2	0	1	0	0	0	0	0
K01790	rfbC, rmlC; dTDP-4-dehydrorhamnose 3,5-epimerase [EC:5.1.3.13]	1	0	0	0	1	0	0	0	0	0
K01791	wecB; UDP-N-acetylglucosamine 2-epimerase (non-hydrolysing) [EC:5.1.3.14]	1	1	1	1	1	1	1	1	1	1
K01799	nicE, maiA; maleate isomerase [EC:5.2.1.1]	0	0	1	0	0	0	0	0	0	0
K01803	TPI, tpiA; triosephosphate isomerase (TIM) [EC:5.3.1.1]	1	1	1	1	1	1	0	1	1	1
K01808	rpiB; ribose 5-phosphate isomerase B [EC:5.3.1.6]	1	1	1	1	1	1	1	1	1	1
K01809	manA, MPI; mannose-6-phosphate isomerase [EC:5.3.1.8]	0	0	0	1	1	1	0	0	0	0
K01810	GPI, pgi; glucose-6-phosphate isomerase [EC:5.3.1.9]	0	0	0	1	1	1	1	0	0	0
K01814	hisA; phosphoribosylformimino-5-aminoimidazole carboxamide ribotide isomerase [EC:5.3.1.16]	1	0	2	1	1	1	0	2	2	2
K01817	trpF; phosphoribosylanthranilate isomerase [EC:5.3.1.24]	1	1	1	0	1	1	0	0	1	1
K01821	praC, xylH; 4-oxalocrotonate tautomerase [EC:5.3.2.6]	1	1	2	1	0	1	1	1	1	1
K01829	dsbH; disulfide reductase [EC:1.8.-.-]	1	0	0	0	0	0	0	0	0	0
K01835	pgm; phosphoglucomutase [EC:5.4.2.2]	1	1	2	2	2	1	0	0	0	0
K01838	pgmB; beta-phosphoglucomutase [EC:5.4.2.6]	2	0	0	1	0	0	0	0	0	0
K01839	deoB; phosphopentomutase [EC:5.4.2.7]	1	1	1	1	1	1	0	1	1	1
K01843	kamA; lysine 2,3-aminomutase [EC:5.4.3.2]	0	0	2	0	1	1	0	0	1	1
K01844	kamD; beta-lysine 5,6-aminomutase alpha subunit [EC:5.4.3.3]	0	0	1	0	0	0	0	0	0	0
K01845	hemL; glutamate-1-semialdehyde 2,1-aminomutase [EC:5.4.3.8]	1	2	1	1	1	0	1	0	0	0
K01847	MUT; methylmalonyl-CoA mutase [EC:5.4.99.2]	0	0	0	0	0	0	0	0	2	2
K01848	E5.4.99.2A, mcmA1; methylmalonyl-CoA mutase, N-terminal domain [EC:5.4.99.2]	0	1	5	0	0	0	0	0	0	0
K01849	E5.4.99.2B, mcmA2; methylmalonyl-CoA mutase, C-terminal domain [EC:5.4.99.2]	0	1	5	0	0	0	1	0	0	0
K01858	INO1, ISYNA1; myo-inositol-1-phosphate synthase [EC:5.5.1.4]	0	1	0	0	0	0	0	0	0	0
K01866	YARS, tyrS; tyrosyl-tRNA synthetase [EC:6.1.1.1]	1	1	1	1	1	1	0	1	1	1
K01867	WARS, trpS; tryptophanyl-tRNA synthetase [EC:6.1.1.2]	1	1	1	1	1	1	1	1	1	1
K01868	TARS, thrS; threonyl-tRNA synthetase [EC:6.1.1.3]	1	1	1	2	1	1	0	1	1	1
K01869	LARS, leuS; leucyl-tRNA synthetase [EC:6.1.1.4]	1	1	1	1	1	1	0	1	1	1
K01870	IARS, ileS; isoleucyl-tRNA synthetase [EC:6.1.1.5]	1	1	1	1	1	1	1	1	1	1

K01872	AARS, alaS; alanyl-tRNA synthetase [EC:6.1.1.7]	0	1	1	1	1	1	0	1	1	1
K01873	VARS, valS; valyl-tRNA synthetase [EC:6.1.1.9]	1	1	1	1	1	1	1	1	1	1
K01874	MARS, metG; methionyl-tRNA synthetase [EC:6.1.1.10]	1	1	1	1	1	1	1	1	1	1
K01875	SARS, serS; seryl-tRNA synthetase [EC:6.1.1.11]	1	1	1	1	1	1	1	1	1	1
K01876	DARS2, aspS; aspartyl-tRNA synthetase [EC:6.1.1.12]	1	1	1	1	1	1	0	1	1	1
K01878	glyQ; glycyl-tRNA synthetase alpha chain [EC:6.1.1.14]	1	1	1	1	1	1	1	1	1	1
K01879	glyS; glycyl-tRNA synthetase beta chain [EC:6.1.1.14]	1	1	1	1	1	1	0	1	1	1
K01881	PARS, proS; prolyl-tRNA synthetase [EC:6.1.1.15]	1	1	1	1	1	1	1	1	1	1
K01883	CARS, cysS; cysteinyl-tRNA synthetase [EC:6.1.1.16]	1	1	1	1	1	1	1	1	1	1
K01885	EARS, gltX; glutamyl-tRNA synthetase [EC:6.1.1.17]	1	0	1	0	0	0	0	0	1	1
K01886	QARS, glnS; glutaminyl-tRNA synthetase [EC:6.1.1.18]	1	1	1	1	1	1	1	1	1	1
K01887	RARS, argS; arginyl-tRNA synthetase [EC:6.1.1.19]	1	1	1	1	1	1	1	1	1	1
K01889	FARSA, pheS; phenylalanyl-tRNA synthetase alpha chain [EC:6.1.1.20]	2	2	2	2	2	1	0	1	1	1
K01890	FARSB, pheT; phenylalanyl-tRNA synthetase beta chain [EC:6.1.1.20]	1	1	1	1	1	1	0	1	1	1
K01892	HARS, hisS; histidyl-tRNA synthetase [EC:6.1.1.21]	1	1	1	1	1	1	0	1	1	1
K01893	NARS, asnS; asparaginyl-tRNA synthetase [EC:6.1.1.22]	1	1	1	0	0	0	0	0	0	0
K01895	ACSS1_2, acs; acetyl-CoA synthetase [EC:6.2.1.1]	1	0	1	2	2	0	0	0	0	0
K01897	ACSL, fadD; long-chain acyl-CoA synthetase [EC:6.2.1.3]	0	0	2	2	0	1	0	0	1	2
K01902	sucD; succinyl-CoA synthetase alpha subunit [EC:6.2.1.5]	0	0	3	0	0	0	0	0	0	0
K01903	sucC; succinyl-CoA synthetase beta subunit [EC:6.2.1.5]	0	0	2	0	0	0	0	0	0	0
K01905	acdA; acetate---CoA ligase (ADP-forming) subunit alpha [EC:6.2.1.13]	0	0	1	0	0	0	0	0	0	0
K01910	citC; [citrate (pro-3S)-lyase] ligase [EC:6.2.1.22]	0	0	1	0	0	0	0	0	0	0
K01912	paaK; phenylacetate-CoA ligase [EC:6.2.1.30]	2	3	3	3	3	2	3	3	3	3
K01915	glnA, GLUL; glutamine synthetase [EC:6.3.1.2]	2	2	5	2	2	3	1	1	1	2
K01916	nadE; NAD+ synthase [EC:6.3.1.5]	0	0	0	1	0	1	0	0	0	0
K01918	panC; pantoate--beta-alanine ligase [EC:6.3.2.1]	1	0	1	0	0	1	1	1	1	1
K01919	gshA; glutamate--cysteine ligase [EC:6.3.2.2]	0	0	1	1	0	0	0	0	0	0
K01921	ddl; D-alanine-D-alanine ligase [EC:6.3.2.4]	1	1	1	1	1	1	0	1	1	1
K01923	purC; phosphoribosylaminoimidazole-succinocarboxamide synthase [EC:6.3.2.6]	2	2	1	1	1	1	1	1	1	1
K01924	murC; UDP-N-acetylmuramate--alanine ligase [EC:6.3.2.8]	1	1	1	1	1	1	1	1	1	1
K01925	murD; UDP-N-acetylmuramoylalanine--D-glutamate ligase [EC:6.3.2.9]	1	1	1	1	1	1	0	1	1	1
K01926	rex; redox-sensing transcriptional repressor	1	1	1	0	0	0	0	0	0	0
K01928	murE; UDP-N-acetylmuramoyl-L-alanyl-D-glutamate--2,6-diaminopimelate ligase [EC:6.3.2.13]	1	1	1	1	1	1	1	1	1	1
K01929	murF; UDP-N-acetylmuramoyl-tripeptide--D-alanyl-D-alanine ligase [EC:6.3.2.10]	1	1	1	1	1	1	1	1	1	1
K01933	purM; phosphoribosylformylglycinamidine cyclo-ligase [EC:6.3.3.1]	1	1	1	1	1	1	1	1	1	1
K01934	MTHFS; 5-formyltetrahydrofolate cyclo-ligase [EC:6.3.3.2]	1	1	1	1	1	1	1	0	0	0
K01935	bioD; dethiobiotin synthetase [EC:6.3.3.3]	0	0	0	0	1	0	0	0	0	0
K01937	pyrG, CTPS; CTP synthase [EC:6.3.4.2]	1	1	1	1	1	1	1	1	1	1
K01938	fhs; formate--tetrahydrofolate ligase [EC:6.3.4.3]	1	1	1	2	1	1	0	1	1	1
K01939	purA, ADSS; adenylosuccinate synthase [EC:6.3.4.4]	1	1	2	1	1	1	1	1	1	1
K01940	argG, ASS1; argininosuccinate synthase [EC:6.3.4.5]	1	1	1	1	1	1	1	1	1	1
K01945	purD; phosphoribosylamine---glycine ligase [EC:6.3.4.13]	1	1	1	1	1	1	1	1	1	1
K01950	E6.3.5.1, NADSYN1, QNS1, nadE; NAD+ synthase (glutamine-hydrolysing) [EC:6.3.5.1]	1	1	1	0	1	1	1	1	2	1
K01951	guaA, GMPS; GMP synthase (glutamine-hydrolysing) [EC:6.3.5.2]	2	2	2	2	1	1	0	1	1	1
K01952	PFAS, purL; phosphoribosylformylglycinamidine synthase [EC:6.3.5.3]	1	1	1	1	1	1	0	1	1	1
K01953	asnB, ASNS; asparagine synthase (glutamine-hydrolysing) [EC:6.3.5.4]	0	1	3	0	1	1	0	0	1	1
K01955	carB, CPA2; carbamoyl-phosphate synthase large subunit [EC:6.3.5.5]	3	0	4	1	1	3	0	1	2	2
K01956	carA, CPA1; carbamoyl-phosphate synthase small subunit [EC:6.3.5.5]	0	0	1	1	1	1	0	1	1	1
K01958	PC, pyc; pyruvate carboxylase [EC:6.4.1.1]	1	0	1	1	1	0	0	1	0	0
K01961	accC; acetyl-CoA carboxylase, biotin carboxylase subunit [EC:6.4.1.2 6.3.4.14]	0	0	0	0	1	1	0	0	0	0
K01962	accA; acetyl-CoA carboxylase carboxyl transferase subunit alpha [EC:6.4.1.2 2.1.3.15]	0	0	0	0	1	1	0	0	0	0
K01963	accD; acetyl-CoA carboxylase carboxyl transferase subunit beta [EC:6.4.1.2 2.1.3.15]	0	0	0	0	1	1	0	0	0	0
K01966	PCCB, pccB; propionyl-CoA carboxylase beta chain [EC:6.4.1.3 2.1.3.15]	1	1	4	0	0	1	1	1	1	1
K01971	ligD; bifunctional non-homologous end joining protein LigD [EC:6.5.1.1]	1	2	1	0	0	0	0	0	1	1
K01972	E6.5.1.2, ligA, ligB; DNA ligase (NAD+) [EC:6.5.1.2]	1	1	1	1	1	1	1	1	1	1
K01975	thpR; RNA 2',3'-cyclic 3'-phosphodiesterase [EC:3.1.4.58]	1	1	1	1	1	0	0	1	1	1
K01989	K01989; putative tryptophan/tyrosine transport system substrate-binding protein	1	1	1	1	1	0	0	0	0	0
K01990	ABC-2.A; ABC-2 type transport system ATP-binding protein	9	3	22	2	12	3	3	6	5	5
K01992	ABC-2.P; ABC-2 type transport system permease protein	9	3	15	2	9	2	2	1	1	1
K01993	ABC-2.TX; HlyD family secretion protein	2	1	3	0	0	0	1	1	0	0
K01994	gerE; LuxR family transcriptional regulator, transcriptional regulator of spore coat protein	0	0	1	0	0	0	0	0	0	0
K01995	livG; branched-chain amino acid transport system ATP-binding protein	2	2	11	1	0	0	2	0	0	0
K01996	livF; branched-chain amino acid transport system ATP-binding protein	2	3	12	1	0	0	2	1	0	0
K01997	livH; branched-chain amino acid transport system permease protein	2	2	10	1	0	0	2	0	0	0
K01998	livM; branched-chain amino acid transport system permease protein	2	2	10	1	0	0	1	1	0	0
K01999	livK; branched-chain amino acid transport system substrate-binding protein	1	2	16	1	0	1	2	1	0	0
K02000	proV; glycine betaine/proline transport system ATP-binding protein [EC:7.6.2.9]	0	0	0	0	1	0	1	1	0	0
K02001	proW; glycine betaine/proline transport system permease protein	0	0	0	0	1	0	1	1	0	0
K02002	proX; glycine betaine/proline transport system substrate-binding protein	0	1	1	0	1	0	1	1	0	0
K02003	ABC.CD.A; putative ABC transport system ATP-binding protein	6	2	14	3	5	1	2	2	7	5
K02004	ABC.CD.P; putative ABC transport system permease protein	6	2	15	3	5	1	2	2	7	5
K02005	ABC.CD.TX; HlyD family secretion protein	1	1	8	0	0	0	1	1	1	1
K02006	cbiO; cobalt/nickel transport system ATP-binding protein	2	1	4	2	2	0	1	0	1	1
K02007	cbiM; cobalt/nickel transport system permease protein	2	1	3	2	2	0	2	0	1	1
K02008	cbiQ; cobalt/nickel transport system permease protein	2	1	4	2	2	0	2	0	1	1
K02009	cbiN; cobalt/nickel transport protein	1	1	1	1	1	0	1	0	0	0
K02010	afuC, fbpC; iron(III) transport system ATP-binding protein [EC:7.2.2.7]	0	0	1	0	0	0	0	0	0	0
K02013	ABC.FEV.A; iron complex transport system ATP-binding protein [EC:7.2.2.-]	4	2	1	1	1	1	2	0	0	0
K02015	ABC.FEV.P; iron complex transport system permease protein	4	2	1	1	1	1	3	0	0	0
K02016	ABC.FEV.S; iron complex transport system substrate-binding protein	3	2	1	1	1	1	3	0	0	0
K02017	modC; molybdate transport system ATP-binding protein [EC:7.3.2.5]	1	1	1	0	0	2	1	1	2	2
K02018	modB; molybdate transport system permease protein	1	1	1	0	0	2	1	1	1	1
K02019	modE; molybdate transport system regulatory protein	2	1	1	0	0	0	1	0	2	2
K02020	modA; molybdate transport system substrate-binding protein	1	1	1	0	0	2	2	2	2	2
K02022	ABC.MR.TX; HlyD family secretion protein	0	0	1	0	0	0	0	0	0	0
K02025	ABC.MS.P; multiple sugar transport system permease protein	0	0	1	0	0	0	1	0	0	0
K02026	ABC.MS.P1; multiple sugar transport system permease protein	0	0	1	0	0	0	1	0	0	0
K02027	ABC.MS.S; multiple sugar transport system substrate-binding protein	0	0	3	0	0	0	0	0	0	0
K02028	ABC.PA.A; polar amino acid transport system ATP-binding protein [EC:7.4.2.1]	2	2	7	4	2	1	0	2	1	1
K02029	ABC.PA.P; polar amino acid transport system permease protein	3	3	7	4	2	1	0	2	1	1
K02030	ABC.PA.S; polar amino acid transport system substrate-binding protein	3	2	5	3	2	1	0	2	2	2
K02031	ddpD; peptide/nickel transport system ATP-binding protein	0	2	11	3	1	0	0	0	0	0
K02032	ddpF; peptide/nickel transport system ATP-binding protein	0	1	7	3	1	0	0	0	0	0
K02033	ABC.PE.P; peptide/nickel transport system permease protein	0	3	16	3	1	0	0	0	0	0
K02034	ABC.PE.P1; peptide/nickel transport system permease protein	0	2	16	1	1	0	0	0	0	0
K02035	ABC.PE.S; peptide/nickel transport system substrate-binding protein	0	3	17	3	1	0	0	0	0	0
K02036	pstB; phosphate transport system ATP-binding protein [EC:7.3.2.1]	1	0	2	0	1	1	0	1	1	1
K02037	pstC; phosphate transport system permease protein	1	0	2	0	1	1	1	1	1	1
K02038	pstA; phosphate transport system permease protein	1	0	2	0	1	1	1	1	1	1
K02039	phoU; phosphate transport system protein	1	0	1	0	1	1	1	1	1	1
K02040	pstS; phosphate transport system substrate-binding protein	1	0	2	0	1	1	1	1	1	1
K02041	phnC; phosphonate transport system ATP-binding protein [EC:7.3.2.2]	0	0	1	0	0	0	0	0	0	0
K02042	phnE; phosphonate transport system permease protein	0	0	2	0	0	0	0	0	0	0
K02044	phnD; phosphonate transport system substrate-binding protein	0	0	1	0	0	0	0	0	0	0
K02049	ABC.SN.A; NitT/TauT family transport system ATP-binding protein	3	3	1	0	1	3	2	0	2	2
K02050	ABC.SN.P; NitT/TauT family transport system permease protein	4	2	1	0	1	3	2	0	1	1
K02051	ABC.SN.S; NitT/TauT family transport system substrate-binding protein	3	2	1	0	1	3	2	0	1	1
K02052	ABC.SP.A; putative spermidine/putrescine transport system ATP-binding protein	0	0	2	0	0	0	0	0	0	0
K02053	ABC.SP.P; putative spermidine/putrescine transport system permease protein	0	0	3	0	0	0	0	0	0	0
K02054	ABC.SP.P1; putative spermidine/putrescine transport system permease protein	0	0	3	0	0	0	0	0	0	0
K02055	ABC.SP.S; putative spermidine/putrescine transport system substrate-binding protein	0	0	3	0	0	0	0	0	0	0
K02056	ABC.SS.A; simple sugar transport system ATP-binding protein [EC:7.5.2.-]	1	1	4	0	0	0	2	1	0	0
K02057	ABC.SS.P; simple sugar transport system permease protein	2	2	9	1	1	0	2	2	0	0
K02071	metN; D-methionine transport system ATP-binding protein	1	1	1	1	0	0	0	0	0	0
K02072	metI; D-methionine transport system permease protein	1	1	1	1	0	0	0	0	0	0
K02073	metQ; D-methionine transport system substrate-binding protein	1	1	1	1	0	0	0	0	0	0
K02078	acpP; acyl carrier protein	3	1	2	0	2	3	2	3	4	3
K02086	dnaD; DNA replication protein	1	1	1	1	1	1	1	1	1	1
K02099	araC; AraC family transcriptional regulator, arabinose operon regulatory protein	0	0	0	0	0	0	2	0	0	0
K02106	atoE; short-chain fatty acids transporter	0	2	0	0	0	0	0	0	0	0
K02108	ATPF0A, atpB; F-type H+-transporting ATPase subunit a	1	1	1	1	1	1	0	1	1	1
K02109	ATPF0B, atpF; F-type H+-transporting ATPase subunit b	1	1	1	1	1	0	1	1	1	0
K02110	ATPF0C, atpE; F-type H+-transporting ATPase subunit c	1	1	1	1	1	1	1	1	1	1
K02111	ATPF1A, atpA; F-type H+/Na+-transporting ATPase subunit alpha [EC:7.1.2.2 7.2.2.1]	0	1	1	1	1	1	1	1	1	1
K02112	ATPF1B, atpD; F-type H+/Na+-transporting ATPase subunit beta [EC:7.1.2.2 7.2.2.1]	1	1	1	1	1	1	1	1	1	1
K02113	ATPF1D, atpH; F-type H+-transporting ATPase subunit delta	1	1	1	1	1	1	1	1	1	1

K02114	ATPF1E, atpC; F-type H+-transporting ATPase subunit epsilon	1	1	1	1	1	1	1	1	1	1
K02115	ATPF1G, atpG; F-type H+-transporting ATPase subunit gamma	1	1	1	1	1	1	1	1	1	1
K02117	ATPVA, ntpA, atpA; V/A-type H+/Na+-transporting ATPase subunit A [EC:7.1.2.2 7.2.2.1]	0	0	0	0	0	1	0	0	0	0
K02118	ATPVB, ntpB, atpB; V/A-type H+/Na+-transporting ATPase subunit B	0	0	0	0	0	1	0	0	0	0
K02119	ATPVC, ntpC, atpC; V/A-type H+/Na+-transporting ATPase subunit C	0	0	0	0	0	1	0	0	0	0
K02120	ATPVD, ntpD, atpD; V/A-type H+/Na+-transporting ATPase subunit D	0	0	0	0	0	1	0	0	0	0
K02121	ATPVE, ntpE, atpE; V/A-type H+/Na+-transporting ATPase subunit E	0	0	0	0	0	1	0	0	0	0
K02122	ATPVF, ntpF, atpF; V/A-type H+/Na+-transporting ATPase subunit F	0	0	0	0	0	1	0	0	0	0
K02123	ATPVI, ntpI, atpI; V/A-type H+/Na+-transporting ATPase subunit I	0	0	0	0	0	1	0	0	0	0
K02124	ATPVK, ntpK, atpK; V/A-type H+/Na+-transporting ATPase subunit K	0	0	0	0	0	1	0	0	0	0
K02160	accB, bccP; acetyl-CoA carboxylase biotin carboxyl carrier protein	0	0	2	0	1	1	0	0	0	0
K02168	betT, betS; choline/glycine/proline betaine transport protein	1	0	1	1	3	0	0	0	0	0
K02171	blaI; BlaI family transcriptional regulator, penicillinase repressor	2	0	2	0	1	0	1	0	0	0
K02172	blaR1; bla regulator protein blaR1	0	0	1	0	0	0	0	0	0	0
K02188	cbiD; cobalt-precorrin-5B (C1)-methyltransferase [EC:2.1.1.195]	1	1	1	1	1	0	1	0	0	0
K02189	cbiG; cobalt-precorrin 5A hydrolase [EC:3.7.1.12]	0	1	1	1	1	0	1	0	0	0
K02190	cbiK; sirohydrochlorin cobaltochelatase [EC:4.99.1.3]	0	0	0	1	1	0	1	0	0	0
K02191	cbiT; cobalt-precorrin-6B (C15)-methyltransferase [EC:2.1.1.196]	1	1	1	0	0	0	0	0	0	0
K02193	ccmA; heme exporter protein A [EC:7.6.2.5]	1	1	0	0	0	0	0	0	0	0
K02194	ccmB; heme exporter protein B	1	1	0	0	0	0	0	0	0	0
K02195	ccmC; heme exporter protein C	1	1	0	0	0	0	0	0	0	0
K02197	ccmE; cytochrome c-type biogenesis protein CcmE	1	1	0	0	0	0	0	0	0	0
K02198	ccmF; cytochrome c-type biogenesis protein CcmF	1	1	0	0	0	0	0	0	0	0
K02199	ccmG, dsbE; cytochrome c biogenesis protein CcmG, thiol:disulfide interchange protein DsbE	0	0	0	1	0	0	0	0	0	0
K02204	thrB2; homoserine kinase type II [EC:2.7.1.39]	1	1	3	1	5	0	0	0	0	0
K02205	rocE, rocC; arginine/ornithine permease	0	1	0	0	0	0	0	0	0	0
K02221	yggT; YggT family protein	1	1	1	1	1	0	1	1	0	0
K02224	cobB-cbiA; cobyrinic acid a,c-diamide synthase [EC:6.3.5.9 6.3.5.11]	3	1	2	1	1	0	1	0	0	0
K02226	cobC, phpB; alpha-ribazole phosphatase [EC:3.1.3.73]	1	1	2	1	1	0	1	0	1	1
K02227	cbiB, cobD; adenosylcobinamide-phosphate synthase [EC:6.3.1.10]	1	1	1	1	1	0	1	0	0	0
K02231	cobP, cobU; adenosylcobinamide kinase / adenosylcobinamide-phosphate guanylyltransferase [EC:2.7.1.156 2.7.7.62]	1	1	1	1	1	0	1	0	0	0
K02232	cobQ, cbiP; adenosylcobyric acid synthase [EC:6.3.5.10]	1	1	1	1	1	0	1	0	0	0
K02233	E2.7.8.26, cobS, cobV; adenosylcobinamide-GDP ribazoletransferase [EC:2.7.8.26]	2	1	1	1	1	0	1	1	0	0
K02234	cobW; cobalamin biosynthesis protein CobW	0	0	2	0	0	0	0	0	0	0
K02237	comEA; competence protein ComEA	1	1	1	0	0	1	1	0	1	1
K02238	comEC; competence protein ComEC	2	1	1	1	1	1	1	1	1	1
K02241	comFB; competence protein ComFB	0	1	1	0	0	0	0	1	1	1
K02242	comFC; competence protein ComFC	1	1	0	1	1	1	1	1	1	1
K02278	cpaA, tadV; prepilin peptidase CpaA [EC:3.4.23.43]	0	1	1	0	0	0	0	0	0	0
K02279	cpaB, rcpC; pilus assembly protein CpaB	3	1	1	0	1	0	0	1	1	1
K02282	cpaE, tadZ; pilus assembly protein CpaE	0	0	1	0	0	0	0	1	1	1
K02283	cpaF, tadA; pilus assembly protein CpaF [EC:7.4.2.8]	3	1	1	0	1	0	0	1	1	1
K02292	crtO; beta-carotene ketolase (CrtO type)	0	0	1	0	0	0	0	0	0	0
K02304	MET8; precorrin-2 dehydrogenase / sirohydrochlorin ferrochelatase [EC:1.3.1.76 4.99.1.4]	1	1	1	0	1	0	1	0	0	0
K02313	dnaA; chromosomal replication initiator protein	1	1	1	1	1	1	0	1	1	1
K02314	dnaB; replicative DNA helicase [EC:5.6.2.3]	1	1	1	1	1	1	1	1	1	1
K02315	dnaC; DNA replication protein DnaC	1	1	1	2	2	1	1	1	1	1
K02316	dnaG; DNA primase [EC:2.7.7.101]	1	1	1	1	1	1	1	1	1	1
K02334	dpo; DNA polymerase bacteriophage-type [EC:2.7.7.7]	0	0	0	0	0	0	0	0	0	1
K02335	polA; DNA polymerase I [EC:2.7.7.7]	1	1	1	1	1	1	1	1	1	1
K02337	dnaE; DNA polymerase III subunit alpha [EC:2.7.7.7]	1	2	1	1	1	1	1	1	1	1
K02338	dnaN; DNA polymerase III subunit beta [EC:2.7.7.7]	1	1	1	1	1	1	1	1	1	1
K02340	holA; DNA polymerase III subunit delta [EC:2.7.7.7]	1	1	1	1	1	1	1	1	1	1
K02341	holB; DNA polymerase III subunit delta' [EC:2.7.7.7]	1	1	1	1	0	1	1	1	1	1
K02342	dnaQ; DNA polymerase III subunit epsilon [EC:2.7.7.7]	0	0	1	0	0	0	0	0	0	0
K02343	dnaX; DNA polymerase III subunit gamma/tau [EC:2.7.7.7]	1	1	1	1	1	1	0	1	1	1
K02346	dinB; DNA polymerase IV [EC:2.7.7.7]	3	1	2	1	1	0	0	1	1	1
K02347	polX, dpx; DNA polymerase (family X)	0	0	1	0	0	0	0	0	0	0
K02348	elaA; ElaA protein	1	0	0	0	0	0	0	1	0	0
K02355	fusA, GFM, EFG; elongation factor G	2	1	2	2	2	2	1	1	1	2
K02356	efp; elongation factor P	1	1	1	1	1	1	1	1	1	1
K02357	tsf, TSFM; elongation factor Ts	1	0	1	1	1	1	1	1	1	1
K02358	tuf, TUFM; elongation factor Tu	2	1	2	0	0	0	1	1	1	1
K02363	entE, dhbE, vibE, mxcE; 2,3-dihydroxybenzoate---[aryl-carrier protein] ligase [EC:6.3.2.14 6.2.1.71]	0	0	1	0	0	0	0	0	0	0
K02371	fabK; enoyl-[acyl-carrier protein] reductase II [EC:1.3.1.9]	1	1	1	0	1	1	2	1	1	1
K02372	fabZ; 3-hydroxyacyl-[acyl-carrier-protein] dehydratase [EC:4.2.1.59]	1	1	1	0	1	1	1	1	1	1
K02379	fdhD; FdhD protein	1	1	1	0	0	0	1	0	0	0
K02385	flbD; flagellar protein FlbD	0	1	1	0	0	0	1	0	1	1
K02387	flgB; flagellar basal-body rod protein FlgB	0	1	1	0	1	0	1	0	1	1
K02388	flgC; flagellar basal-body rod protein FlgC	0	1	1	0	1	0	1	0	1	1
K02389	flgD; flagellar basal-body rod modification protein FlgD	0	1	1	0	1	0	1	0	1	1
K02390	flgE; flagellar hook protein FlgE	0	1	1	0	1	0	1	0	2	1
K02392	flgG; flagellar basal-body rod protein FlgG	0	2	0	0	2	0	2	0	1	2
K02396	flgK; flagellar hook-associated protein 1	0	1	1	0	1	0	1	0	1	1
K02397	flgL; flagellar hook-associated protein 3 FlgL	0	1	0	0	1	0	0	0	0	0
K02398	flgM; negative regulator of flagellin synthesis FlgM	0	1	0	0	0	0	0	0	0	0
K02400	flhA; flagellar biosynthesis protein FlhA	0	1	1	0	1	0	1	0	1	1
K02401	flhB; flagellar biosynthesis protein FlhB	0	1	1	0	1	0	1	0	1	1
K02404	flhF; flagellar biosynthesis protein FlhF	0	1	1	0	0	0	0	0	0	0
K02405	fliA, whiG; RNA polymerase sigma factor FliA	0	1	1	0	1	0	1	0	1	1
K02406	fliC, hag; flagellin	0	0	1	0	1	0	0	0	1	1
K02407	fliD; flagellar hook-associated protein 2	0	0	0	0	0	0	0	0	1	1
K02408	fliE; flagellar hook-basal body complex protein FliE	0	1	1	0	1	0	1	0	1	1
K02409	fliF; flagellar M-ring protein FliF	0	1	1	0	1	0	1	0	1	1
K02410	fliG; flagellar motor switch protein FliG	0	1	1	0	1	0	1	0	1	1
K02412	fliI; flagellum-specific ATP synthase [EC:7.4.2.8]	0	1	0	0	1	0	1	0	1	1
K02413	fliJ; flagellar protein FliJ	0	1	1	0	0	0	0	0	0	0
K02415	fliL; flagellar protein FliL	0	1	1	0	0	0	0	0	0	0
K02416	fliM; flagellar motor switch protein FliM	0	1	1	0	1	0	1	0	1	1
K02417	fliN; flagellar motor switch protein FliN	0	1	2	0	1	0	1	0	1	1
K02418	fliO, fliZ; flagellar protein FliO/FliZ	0	1	0	0	0	0	0	0	0	0
K02419	fliP; flagellar biosynthesis protein FliP	0	1	1	0	1	0	1	0	1	1
K02420	fliQ; flagellar biosynthesis protein FliQ	0	1	1	0	1	0	1	0	1	1
K02421	fliR; flagellar biosynthesis protein FliR	0	1	1	0	1	0	1	0	1	1
K02422	fliS; flagellar secretion chaperone FliS	0	1	1	0	1	0	1	0	1	0
K02433	gatA, QRSL1; aspartyl-tRNA(Asn)/glutamyl-tRNA(Gln) amidotransferase subunit A [EC:6.3.5.6 6.3.5.7]	2	2	1	1	1	2	2	1	1	1
K02434	gatB, PET112; aspartyl-tRNA(Asn)/glutamyl-tRNA(Gln) amidotransferase subunit B [EC:6.3.5.6 6.3.5.7]	2	2	1	1	1	1	1	1	1	1
K02435	gatC, GATC; aspartyl-tRNA(Asn)/glutamyl-tRNA(Gln) amidotransferase subunit C [EC:6.3.5.6 6.3.5.7]	2	2	1	1	1	1	1	1	1	1
K02437	gcvH, GCSH; glycine cleavage system H protein	2	1	3	2	0	0	0	1	1	1
K02440	GLPF; glycerol uptake facilitator protein	0	1	1	0	0	0	0	0	0	0
K02443	glpP; glycerol uptake operon antiterminator	0	0	1	0	0	0	0	0	0	0
K02446	glpX; fructose-1,6-bisphosphatase II [EC:3.1.3.11]	0	1	1	1	1	0	0	0	0	0
K02450	gspA; general secretion pathway protein A	0	0	1	0	0	0	0	0	0	0
K02456	gspG; general secretion pathway protein G	1	2	1	0	0	0	0	0	0	0
K02469	gyrA; DNA gyrase subunit A [EC:5.6.2.2]	1	1	1	1	1	1	1	1	1	1
K02470	gyrB; DNA gyrase subunit B [EC:5.6.2.2]	1	1	1	1	1	1	1	1	1	1
K02472	wecC; UDP-N-acetyl-D-mannosaminuronic acid dehydrogenase [EC:1.1.1.336]	1	0	2	0	0	0	0	0	1	1
K02473	wbpP; UDP-N-acetylglucosamine/UDP-N-acetylgalactosamine 4-epimerase [EC:5.1.3.7 5.1.3.-]	0	0	0	0	0	1	0	0	0	0
K02474	wbpO; UDP-N-acetyl-D-glucosamine/UDP-N-acetyl-D-galactosamine dehydrogenase [EC:1.1.1.136 1.1.1.-]	0	0	0	0	0	1	0	0	0	0
K02477	K02477; two-component system, LytTR family, response regulator	2	0	1	0	0	0	0	0	0	0
K02478	K02478; two-component system, LytTR family, sensor kinase [EC:2.7.13.3]	0	0	1	0	0	0	0	0	0	0
K02479	K02479; two-component system, NarL family, response regulator	0	0	3	0	0	0	0	0	0	0
K02481	flgR; two-component system, NtrC family, response regulator	3	0	2	0	0	0	0	0	0	0
K02482	flgS; two-component system, NtrC family, sensor kinase [EC:2.7.13.3]	2	0	0	0	0	0	0	0	0	0
K02483	K02483; two-component system, OmpR family, response regulator	5	1	6	1	1	0	0	0	1	0
K02484	K02484; two-component system, OmpR family, sensor kinase [EC:2.7.13.3]	0	1	1	0	0	0	0	0	0	0
K02490	spo0F; two-component system, response regulator, stage 0 sporulation protein F	1	2	2	0	0	0	0	0	0	0
K02491	kinA; two-component system, sporulation sensor kinase A [EC:2.7.13.3]	1	0	0	0	0	0	0	0	0	0
K02492	hemA; glutamyl-tRNA reductase [EC:1.2.1.70]	1	1	2	1	1	0	1	0	0	0
K02493	hemK, prmC, HEMK; release factor glutamine methyltransferase [EC:2.1.1.297]	1	1	1	1	1	1	1	1	1	1
K02495	hemN, hemZ; oxygen-independent coproporphyrinogen III oxidase [EC:1.3.98.3]	0	0	0	1	0	0	0	0	0	0
K02499	yabN; tetrapyrrole methylase family protein / MazG family protein	1	1	1	1	1	1	1	1	1	1
K02500	hisF; imidazole glycerol-phosphate synthase subunit HisF [EC:4.3.2.10]	1	0	1	1	1	1	0	1	1	1
K02501	hisH; imidazole glycerol-phosphate synthase subunit HisH [EC:4.3.2.10]	1	0	1	1	1	1	0	1	1	1
K02502	hisZ; ATP phosphoribosyltransferase regulatory subunit	1	0	0	1	1	1	0	1	1	1
K02503	HINT1, hinT, hit; histidine triad (HIT) family protein	1	1	1	1	1	1	1	1	1	1

K02518	infA; translation initiation factor IF-1	1	1	1	1	1	1	1	1	1	1
K02519	infB, MTIF2; translation initiation factor IF-2	1	1	0	0	0	1	1	1	0	0
K02520	infC, MTIF3; translation initiation factor IF-3	1	1	1	1	1	1	0	1	1	1
K02526	kdgT; 2-keto-3-deoxygluconate permease	0	0	0	0	0	0	0	0	1	1
K02528	ksgA; 16S rRNA (adenine1518-N6/adenine1519-N6)-dimethyltransferase [EC:2.1.1.182]	1	1	1	1	1	1	1	1	1	1
K02529	lacI, galR; LacI family transcriptional regulator	0	0	3	0	0	0	0	0	0	0
K02535	lpxC; UDP-3-O-[3-hydroxymyristoyl] N-acetylglucosamine deacetylase [EC:3.5.1.108]	0	0	1	0	0	0	0	0	0	0
K02548	menA; 1,4-dihydroxy-2-naphthoate polyprenyltransferase [EC:2.5.1.74]	1	1	1	1	0	0	1	0	0	0
K02549	menC; o-succinylbenzoate synthase [EC:4.2.1.113]	0	0	1	0	0	0	0	0	0	0
K02556	motA; chemotaxis protein MotA	0	1	2	0	2	0	2	0	1	1
K02557	motB; chemotaxis protein MotB	0	1	1	0	2	0	2	0	1	1
K02563	murG; UDP-N-acetylglucosamine--N-acetylmuramyl-(pentapeptide) pyrophosphoryl-undecaprenol N-acetylglucosamine transferase [EC:2.4.1.227]	1	1	1	1	1	1	1	1	1	1
K02564	nagB, GNPDA; glucosamine-6-phosphate deaminase [EC:3.5.99.6]	1	1	0	0	0	0	1	0	0	0
K02566	nagD; 5'-nucleotidase [EC:3.1.3.5]	0	0	0	0	1	1	0	0	1	1
K02574	napH; ferredoxin-type protein NapH	0	0	0	0	1	0	0	0	0	0
K02575	NRT, narK, nrtP, nasA; MFS transporter, NNP family, nitrate/nitrite transporter	0	0	1	0	1	0	0	0	0	0
K02584	nifA; Nif-specific regulatory protein	0	0	1	0	0	0	0	0	0	0
K02585	nifB; nitrogen fixation protein NifB	0	0	1	0	0	0	0	0	0	0
K02586	nifD; nitrogenase molybdenum-iron protein alpha chain [EC:1.18.6.1]	0	0	2	0	0	0	0	0	0	0
K02588	nifH; nitrogenase iron protein NifH	1	0	1	0	0	0	0	0	0	0
K02591	nifK; nitrogenase molybdenum-iron protein beta chain [EC:1.18.6.1]	0	0	2	0	0	0	0	0	0	0
K02594	nifV; homocitrate synthase NifV [EC:2.3.3.14]	0	1	1	0	0	0	0	0	0	0
K02600	nusA; transcription termination/antitermination protein NusA	1	1	1	1	1	1	1	1	1	1
K02601	nusG; transcription termination/antitermination protein NusG	1	1	1	1	1	1	1	1	1	0
K02614	paaI; acyl-CoA thioesterase [EC:3.1.2.-]	2	0	0	0	0	1	1	0	0	0
K02617	paaY; phenylacetic acid degradation protein	0	0	0	1	0	0	1	1	0	0
K02619	pabC; 4-amino-4-deoxychorismate lyase [EC:4.1.3.38]	1	0	0	1	1	0	0	0	0	0
K02624	pcaR; IclR family transcriptional regulator, pca regulon regulatory protein	0	0	1	0	0	0	0	0	0	0
K02626	pdaD; arginine decarboxylase [EC:4.1.1.19]	0	0	1	0	0	0	0	0	0	0
K02647	cdaR; carbohydrate diacid regulator	0	0	1	0	0	0	0	0	0	0
K02650	pilA; type IV pilus assembly protein PilA	0	0	1	0	0	1	1	0	0	0
K02651	flp, pilA; pilus assembly protein Flp/PilA	0	1	1	0	0	0	0	0	1	1
K02652	pilB; type IV pilus assembly protein PilB	1	1	2	0	0	1	1	0	0	0
K02653	pilC; type IV pilus assembly protein PilC	1	1	2	0	0	1	1	0	0	0
K02654	pilD, pppA; leader peptidase (prepilin peptidase) / N-methyltransferase [EC:3.4.23.43 2.1.1.-]	3	1	2	0	0	1	1	0	1	1
K02662	pilM; type IV pilus assembly protein PilM	1	1	1	0	0	0	0	0	0	0
K02669	pilT; twitching motility protein PilT	1	1	2	1	0	1	0	0	0	0
K02687	prmA; ribosomal protein L11 methyltransferase [EC:2.1.1.-]	1	1	1	1	1	1	1	1	0	0
K02688	prpR; transcriptional regulator, propionate catabolism operon regulatory protein	0	0	3	0	0	0	0	0	0	0
K02823	pyrDII; dihydroorotate dehydrogenase electron transfer subunit	1	1	2	1	1	1	1	1	1	1
K02824	uraA, pyrP; uracil permease	2	2	2	1	1	0	0	0	0	0
K02825	pyrR; pyrimidine operon attenuation protein / uracil phosphoribosyltransferase [EC:2.4.2.9]	0	1	1	1	1	0	0	0	0	0
K02834	rbfA; ribosome-binding factor A	1	1	1	1	1	1	1	1	1	1
K02835	prfA, MTRF1, MRF1; peptide chain release factor 1	1	1	1	1	1	1	1	1	1	1
K02836	prfB; peptide chain release factor 2	1	1	1	1	1	1	1	1	1	1
K02838	frr, MRRF, RRF; ribosome recycling factor	1	1	1	1	1	1	1	1	1	1
K02839	prfH; peptide chain release factor	0	0	1	0	0	0	0	0	0	0
K02851	wecA, tagO, rfe; UDP-GlcNAc:undecaprenyl-phosphate/decaprenyl-phosphate GlcNAc-1-phosphate transferase [EC:2.7.8.33 2.7.8.35]	1	1	1	1	1	1	1	1	1	1
K02860	rimM; 16S rRNA processing protein RimM	1	1	1	1	1	1	1	1	1	1
K02863	RP-L1, MRPL1, rplA; large subunit ribosomal protein L1	1	1	1	1	1	1	1	1	1	0
K02864	RP-L10, MRPL10, rplJ; large subunit ribosomal protein L10	1	1	1	1	1	1	1	1	1	0
K02867	RP-L11, MRPL11, rplK; large subunit ribosomal protein L11	1	1	1	1	1	1	1	1	1	0
K02871	RP-L13, MRPL13, rplM; large subunit ribosomal protein L13	1	1	1	1	1	1	1	1	1	1
K02874	RP-L14, MRPL14, rplN; large subunit ribosomal protein L14	1	1	1	1	1	1	1	1	1	1
K02876	RP-L15, MRPL15, rplO; large subunit ribosomal protein L15	1	1	1	1	1	1	1	1	1	1
K02878	RP-L16, MRPL16, rplP; large subunit ribosomal protein L16	1	1	1	1	1	1	0	1	1	1
K02879	RP-L17, MRPL17, rplQ; large subunit ribosomal protein L17	1	1	1	1	1	1	1	1	1	1
K02881	RP-L18, MRPL18, rplR; large subunit ribosomal protein L18	1	1	1	1	1	1	1	1	1	1
K02884	RP-L19, MRPL19, rplS; large subunit ribosomal protein L19	1	1	1	1	1	1	1	1	1	1
K02886	RP-L2, MRPL2, rplB; large subunit ribosomal protein L2	1	1	1	1	1	1	1	1	1	1
K02887	RP-L20, MRPL20, rplT; large subunit ribosomal protein L20	1	1	1	1	1	1	0	1	1	1
K02888	RP-L21, MRPL21, rplU; large subunit ribosomal protein L21	1	1	1	1	1	1	1	1	1	1
K02890	RP-L22, MRPL22, rplV; large subunit ribosomal protein L22	1	1	1	1	1	1	1	1	1	1
K02892	RP-L23, MRPL23, rplW; large subunit ribosomal protein L23	1	1	1	1	1	1	1	1	1	1
K02895	RP-L24, MRPL24, rplX; large subunit ribosomal protein L24	1	1	1	1	1	1	1	1	1	1
K02897	RP-L25, rplY; large subunit ribosomal protein L25	1	1	1	1	1	0	0	1	1	1
K02899	RP-L27, MRPL27, rpmA; large subunit ribosomal protein L27	1	1	1	1	1	1	1	1	1	1
K02902	RP-L28, MRPL28, rpmB; large subunit ribosomal protein L28	2	2	2	2	2	2	2	2	2	2
K02904	RP-L29, rpmC; large subunit ribosomal protein L29	1	1	1	1	1	1	0	1	1	1
K02906	RP-L3, MRPL3, rplC; large subunit ribosomal protein L3	1	1	1	1	1	1	1	1	1	1
K02907	RP-L30, MRPL30, rpmD; large subunit ribosomal protein L30	1	1	1	1	1	1	1	1	1	1
K02909	RP-L31, rpmE; large subunit ribosomal protein L31	1	1	1	1	1	1	1	1	1	1
K02911	RP-L32, MRPL32, rpmF; large subunit ribosomal protein L32	0	0	1	0	0	1	1	1	0	1
K02913	RP-L33, MRPL33, rpmG; large subunit ribosomal protein L33	0	1	1	1	1	1	0	1	1	0
K02914	RP-L34, MRPL34, rpmH; large subunit ribosomal protein L34	0	0	1	1	0	1	1	1	1	1
K02916	RP-L35, MRPL35, rpmI; large subunit ribosomal protein L35	1	1	1	1	1	1	0	1	1	1
K02919	RP-L36, MRPL36, rpmJ; large subunit ribosomal protein L36	1	0	0	0	0	0	1	1	1	1
K02926	RP-L4, MRPL4, rplD; large subunit ribosomal protein L4	1	1	1	1	1	1	1	1	1	1
K02931	RP-L5, MRPL5, rplE; large subunit ribosomal protein L5	1	1	1	1	1	1	1	1	1	1
K02933	RP-L6, MRPL6, rplF; large subunit ribosomal protein L6	1	1	1	1	1	1	1	1	1	1
K02935	RP-L7, MRPL12, rplL; large subunit ribosomal protein L7/L12	1	1	1	1	1	1	1	1	1	0
K02939	RP-L9, MRPL9, rplI; large subunit ribosomal protein L9	1	1	1	1	1	1	1	1	1	1
K02946	RP-S10, MRPS10, rpsJ; small subunit ribosomal protein S10	1	1	1	0	1	1	1	1	1	1
K02948	RP-S11, MRPS11, rpsK; small subunit ribosomal protein S11	1	1	1	1	1	1	1	1	1	1
K02950	RP-S12, MRPS12, rpsL; small subunit ribosomal protein S12	1	1	1	1	1	1	1	1	0	1
K02952	RP-S13, rpsM; small subunit ribosomal protein S13	1	1	1	1	1	1	1	1	1	1
K02954	RP-S14, MRPS14, rpsN; small subunit ribosomal protein S14	1	1	0	1	1	0	1	1	1	1
K02956	RP-S15, MRPS15, rpsO; small subunit ribosomal protein S15	1	1	1	1	1	1	2	1	1	1
K02959	RP-S16, MRPS16, rpsP; small subunit ribosomal protein S16	1	1	1	1	1	1	1	1	1	1
K02961	RP-S17, MRPS17, rpsQ; small subunit ribosomal protein S17	1	1	1	1	1	1	1	1	1	1
K02963	RP-S18, MRPS18, rpsR; small subunit ribosomal protein S18	0	0	0	1	1	0	0	1	0	0
K02965	RP-S19, rpsS; small subunit ribosomal protein S19	1	1	1	1	1	1	1	1	1	1
K02967	RP-S2, MRPS2, rpsB; small subunit ribosomal protein S2	1	1	1	1	1	1	1	1	1	1
K02968	RP-S20, rpsT; small subunit ribosomal protein S20	1	1	1	1	1	1	1	1	1	1
K02970	RP-S21, MRPS21, rpsU; small subunit ribosomal protein S21	1	1	1	0	0	1	1	1	1	0
K02982	RP-S3, rpsC; small subunit ribosomal protein S3	1	0	1	1	1	1	1	1	1	1
K02986	RP-S4, rpsD; small subunit ribosomal protein S4	1	1	1	1	1	1	1	1	1	1
K02988	RP-S5, MRPS5, rpsE; small subunit ribosomal protein S5	1	1	1	1	1	1	1	1	1	1
K02990	RP-S6, MRPS6, rpsF; small subunit ribosomal protein S6	1	1	1	1	1	1	1	1	1	1
K02992	RP-S7, MRPS7, rpsG; small subunit ribosomal protein S7	1	1	1	1	1	1	0	1	0	1
K02994	RP-S8, rpsH; small subunit ribosomal protein S8	1	1	1	1	1	1	1	1	1	1
K02996	RP-S9, MRPS9, rpsI; small subunit ribosomal protein S9	1	1	1	1	1	1	1	1	1	1
K03040	rpoA; DNA-directed RNA polymerase subunit alpha [EC:2.7.7.6]	1	1	1	1	1	1	1	1	1	1
K03043	rpoB; DNA-directed RNA polymerase subunit beta [EC:2.7.7.6]	1	1	1	1	1	1	0	1	1	0
K03046	rpoC; DNA-directed RNA polymerase subunit beta' [EC:2.7.7.6]	1	1	1	1	1	1	1	1	0	1
K03060	rpoZ; DNA-directed RNA polymerase subunit omega [EC:2.7.7.6]	1	1	1	1	1	1	1	1	1	1
K03070	secA; preprotein translocase subunit SecA [EC:7.4.2.8]	1	1	1	1	1	1	1	1	1	1
K03071	secB; preprotein translocase subunit SecB	0	0	0	0	0	0	0	0	1	0
K03072	secD; preprotein translocase subunit SecD	1	0	2	1	1	1	1	2	1	1
K03073	secE; preprotein translocase subunit SecE	1	1	1	0	0	0	1	1	1	0
K03074	secF; preprotein translocase subunit SecF	1	1	2	1	1	1	1	2	1	1
K03075	secG; preprotein translocase subunit SecG	1	1	1	1	1	1	0	1	1	1
K03076	secY; preprotein translocase subunit SecY	1	1	1	1	1	1	1	1	1	1
K03086	rpoD; RNA polymerase primary sigma factor	1	1	1	1	1	1	1	1	2	2
K03088	rpoE; RNA polymerase sigma-70 factor, ECF subfamily	14	4	11	2	3	3	5	5	7	5
K03090	sigB; RNA polymerase sigma-B factor	0	0	0	0	0	1	0	0	0	0
K03091	sigH; RNA polymerase sporulation-specific sigma factor	5	5	5	4	6	5	4	6	6	5
K03092	rpoN; RNA polymerase sigma-54 factor	1	1	1	1	1	0	0	0	0	0
K03093	sigI; RNA polymerase sigma factor	2	0	1	0	0	0	1	0	0	0
K03098	APOD; apolipoprotein D and lipocalin family protein	0	0	0	0	0	0	0	1	0	0
K03100	lepB; signal peptidase I [EC:3.4.21.89]	4	3	3	3	4	1	1	1	1	1
K03101	lspA; signal peptidase II [EC:3.4.23.36]	1	1	1	1	1	1	1	1	1	1
K03106	SRP54, ffh; signal recognition particle subunit SRP54 [EC:3.6.5.4]	1	1	1	1	1	1	1	1	1	1
K03110	ftsY; fused signal recognition particle receptor	1	1	1	1	1	1	1	1	1	1
K03111	ssb; single-strand DNA-binding protein	2	1	1	1	1	1	1	1	2	2
K03116	tatA; sec-independent protein translocase protein TatA	2	1	1	1	0	0	0	0	0	0

K03117	tatB; sec-independent protein translocase protein TatB	0	0	0	0	0	1	0	0	0	0
K03118	tatC; sec-independent protein translocase protein TatC	1	1	1	1	1	1	0	0	0	0
K03147	thiC; phosphomethylpyrimidine synthase [EC:4.1.99.17]	2	0	0	0	0	1	0	0	1	0
K03148	thiF; sulfur carrier protein ThiS adenylyltransferase [EC:2.7.7.73]	0	0	0	1	0	0	0	0	0	0
K03149	thiG; thiazole synthase [EC:2.8.1.10]	0	0	0	1	0	0	0	0	0	0
K03150	thiH; 2-iminoacetate synthase [EC:4.1.99.19]	0	1	2	2	1	0	0	1	1	1
K03151	thiI; tRNA uracil 4-sulfurtransferase [EC:2.8.1.4]	1	1	1	1	1	1	0	1	1	1
K03152	thiJ; protein deglycase [EC:3.5.1.124]	0	0	1	0	0	0	0	1	0	0
K03154	thiS; sulfur carrier protein	0	1	1	1	0	0	0	0	2	0
K03168	topA; DNA topoisomerase I [EC:5.6.2.1]	1	1	1	1	1	1	1	1	1	1
K03169	topB; DNA topoisomerase III [EC:5.6.2.1]	4	0	2	1	2	0	0	1	2	1
K03177	truB, PUS4, TRUB1; tRNA pseudouridine55 synthase [EC:5.4.99.25]	1	1	1	0	0	1	1	1	0	0
K03179	ubiA; 4-hydroxybenzoate polyprenyltransferase [EC:2.5.1.39]	0	0	1	0	1	1	0	0	0	0
K03182	ubiD; 4-hydroxy-3-polyprenylbenzoate decarboxylase [EC:4.1.1.98]	0	0	5	0	1	1	0	0	1	1
K03183	ubiE; demethylmenaquinone methyltransferase / 2-methoxy-6-polyprenyl-1,4-benzoquinol methylase [EC:2.1.1.163 2.1.1.201]	1	1	2	0	0	0	1	0	0	0
K03186	ubiX, bsdB, PAD1; flavin prenyltransferase [EC:2.5.1.129]	0	1	4	0	1	1	0	0	1	1
K03205	virD4, lvhD4; type IV secretion system protein VirD4 [EC:7.4.2.8]	5	0	0	0	1	0	0	0	0	0
K03210	yajC; preprotein translocase subunit YajC	1	0	1	1	1	1	1	1	1	1
K03215	rumA; 23S rRNA (uracil1939-C5)-methyltransferase [EC:2.1.1.190]	1	1	1	1	1	1	1	1	1	1
K03216	trmL, cspR; tRNA (cytidine/uridine-2'-O-)-methyltransferase [EC:2.1.1.207]	1	1	1	1	1	1	1	1	1	1
K03217	yidC, spoIIIJ, OXA1, ccfA; YidC/Oxa1 family membrane protein insertase	2	0	2	1	0	0	1	0	0	0
K03218	rlmB; 23S rRNA (guanosine2251-2'-O)-methyltransferase [EC:2.1.1.185]	1	1	1	1	1	1	1	1	1	1
K03224	yscN, sctN, hrcN, ssaN; ATP synthase in type III secretion protein N [EC:7.4.2.8]	0	0	1	0	0	0	0	0	0	0
K03276	waaR, waaT, rfaJ; UDP-glucose/galactose:(glucosyl)LPS alpha-1,2-glucosyl/galactosyltransferase [EC:2.4.1.-]	1	0	0	0	0	0	0	0	0	0
K03281	TC.CIC; chloride channel protein, CIC family	0	0	1	0	0	1	0	0	0	0
K03282	mscL; large conductance mechanosensitive channel	1	1	0	1	1	0	0	1	1	1
K03284	corA; magnesium transporter	2	0	3	0	0	1	1	1	2	2
K03293	TC.AAT; amino acid transporter, AAT family	0	0	4	0	0	1	0	1	1	1
K03294	TC.APA; basic amino acid/polyamine antiporter, APA family	0	2	0	0	1	0	0	0	0	0
K03296	TC.HAE1; hydrophobic/amphiphilic exporter-1 (mainly G- bacteria), HAE1 family	1	0	6	1	2	1	1	1	0	0
K03297	emrE, qac, mmr, smr; small multidrug resistance pump	1	0	0	0	0	0	0	0	0	0
K03298	TC.DME; drug/metabolite transporter, DME family	0	0	1	2	0	0	0	0	1	0
K03303	lctP; lactate permease	0	1	2	0	0	1	0	1	0	0
K03306	TC.PIT; inorganic phosphate transporter, PiT family	1	1	1	0	1	0	1	1	2	2
K03307	TC.SSS; solute:Na+ symporter, SSS family	0	1	6	2	1	0	1	1	2	1
K03308	TC.NSS; neurotransmitter:Na+ symporter, NSS family	1	1	1	1	1	0	0	0	0	0
K03310	TC.AGCS; alanine or glycine:cation symporter, AGCS family	0	0	1	1	1	0	1	1	1	1
K03312	gltS; glutamate:Na+ symporter, ESS family	0	0	1	0	0	1	0	0	0	0
K03313	nhaA; Na+:H+ antiporter, NhaA family	0	0	0	0	1	0	0	0	0	0
K03315	nhaC; Na+:H+ antiporter, NhaC family	0	1	2	0	0	0	0	2	0	0
K03319	TC.DASS; divalent anion:Na+ symporter, DASS family	0	0	1	0	0	0	0	0	0	0
K03320	amt, AMT, MEP; ammonium transporter, Amt family	0	0	3	2	3	0	0	2	1	1
K03322	mntH; manganese transport protein	0	0	1	0	0	0	0	0	1	0
K03324	yjbB; phosphate:Na+ symporter	1	1	1	2	0	1	0	0	0	0
K03325	ACR3, arsB; arsenite transporter	0	0	1	0	0	0	0	0	0	0
K03328	TC.PST; polysaccharide transporter, PST family	1	0	0	0	0	0	0	0	0	0
K03378	morA; morphine 6-dehydrogenase [EC:1.1.1.218]	0	0	0	0	0	0	0	1	0	0
K03382	atzB; hydroxydechloroatrazine ethylaminohydrolase [EC:3.5.4.43]	0	0	2	0	0	0	1	0	0	0
K03385	nrfA; nitrite reductase (cytochrome c-552) [EC:1.7.2.2]	1	1	0	0	0	0	0	0	0	0
K03386	PRDX2_4, ahpC; peroxiredoxin 04-Feb [EC:1.11.1.24]	1	0	0	0	2	0	0	1	0	0
K03388	hdrA2; heterodisulfide reductase subunit A2 [EC:1.8.7.3 1.8.98.4 1.8.98.5 1.8.98.6]	2	2	3	0	0	1	2	0	0	0
K03389	hdrB2; heterodisulfide reductase subunit B2 [EC:1.8.7.3 1.8.98.4 1.8.98.5 1.8.98.6]	1	1	1	1	0	1	1	0	0	0
K03390	hdrC2; heterodisulfide reductase subunit C2 [EC:1.8.7.3 1.8.98.4 1.8.98.5 1.8.98.6]	1	1	1	0	0	1	1	0	0	0
K03392	ACMSD; aminocarboxymuconate-semialdehyde decarboxylase [EC:4.1.1.45]	0	0	0	0	0	0	0	1	0	0
K03394	cobI-cbiL; precorrin-2/cobalt-factor-2 C20-methyltransferase [EC:2.1.1.130 2.1.1.151]	0	1	1	1	1	0	1	0	0	0
K03399	cbiE; cobalt-precorrin-7 (C5)-methyltransferase [EC:2.1.1.289]	1	1	1	0	0	0	0	0	0	0
K03402	argR, ahrC; transcriptional regulator of arginine metabolism	1	1	1	1	1	1	1	1	1	1
K03406	mcp; methyl-accepting chemotaxis protein	0	3	14	0	6	0	1	0	2	2
K03407	cheA; two-component system, chemotaxis family, sensor kinase CheA [EC:2.7.13.3]	0	2	3	0	1	0	0	0	1	1
K03408	cheW; purine-binding chemotaxis protein CheW	0	2	4	0	2	0	2	0	1	1
K03409	cheX; chemotaxis protein CheX	0	1	1	0	0	0	0	0	0	0
K03410	cheC; chemotaxis protein CheC	0	1	1	0	1	0	1	1	1	1
K03411	cheD; chemotaxis protein CheD [EC:3.5.1.44]	0	1	1	0	1	0	1	1	1	1
K03412	cheB; two-component system, chemotaxis family, protein-glutamate methylesterase/glutaminase [EC:3.1.1.61 3.5.1.44]	0	1	2	0	1	0	1	0	1	1
K03413	cheY; two-component system, chemotaxis family, chemotaxis protein CheY	0	2	4	0	1	0	1	0	1	1
K03424	tatD; TatD DNase family protein [EC:3.1.21.-]	1	1	1	1	1	1	1	1	1	1
K03426	E3.6.1.22, NUDT12, nudC; NAD+ diphosphatase [EC:3.6.1.22]	1	1	1	0	1	0	0	0	1	1
K03427	hsdM; type I restriction enzyme M protein [EC:2.1.1.72]	1	0	0	0	3	0	0	1	0	1
K03429	ugtP; processive 1,2-diacylglycerol beta-glucosyltransferase [EC:2.4.1.315]	0	1	2	0	0	0	0	1	1	0
K03430	phnW; 2-aminoethylphosphonate-pyruvate transaminase [EC:2.6.1.37]	0	0	1	0	0	0	0	0	0	0
K03431	glmM; phosphoglucosamine mutase [EC:5.4.2.10]	1	1	1	1	1	1	1	1	1	1
K03436	fruR2, fruR; DeoR family transcriptional regulator, fructose operon transcriptional repressor	0	0	1	0	0	0	0	0	0	0
K03437	spoU; RNA methyltransferase, TrmH family	1	1	1	1	1	1	0	1	1	1
K03438	mraW, rsmH; 16S rRNA (cytosine1402-N4)-methyltransferase [EC:2.1.1.199]	1	1	1	1	1	1	0	1	1	1
K03439	trmB, METTL1, TRM8; tRNA (guanine-N7-)-methyltransferase [EC:2.1.1.33]	1	0	1	1	1	1	0	0	1	1
K03446	emrB; MFS transporter, DHA2 family, multidrug resistance protein	0	0	3	0	0	0	0	1	0	0
K03449	MFS.CP; MFS transporter, CP family, cyanate transporter	0	0	0	0	0	0	0	1	0	0
K03451	TC.BCT; betaine/carnitine transporter, BCCT family	2	0	5	3	2	0	0	0	0	0
K03453	TC.BASS; bile acid:Na+ symporter, BASS family	0	0	0	0	0	0	1	0	0	0
K03455	TC.KEF; monovalent cation:H+ antiporter-2, CPA2 family	0	0	1	1	1	0	0	0	1	1
K03457	TC.NCS1; nucleobase:cation symporter-1, NCS1 family	0	1	6	1	1	0	0	2	0	0
K03458	TC.NCS2; nucleobase:cation symporter-2, NCS2 family	0	0	2	0	0	0	0	2	0	0
K03465	thyX, thy1; thymidylate synthase (FAD) [EC:2.1.1.148]	1	1	1	0	0	1	0	1	1	1
K03466	ftsK, spoIIIE; DNA segregation ATPase FtsK/SpoIIIE, S-DNA-T family	1	1	1	0	1	1	1	1	1	2
K03469	rnhA, RNASEH1; ribonuclease HI [EC:3.1.26.4]	1	1	1	1	1	1	1	1	1	1
K03470	rnhB; ribonuclease HII [EC:3.1.26.4]	1	1	1	1	1	1	1	1	1	1
K03495	gidA, mnmG, MTO1; tRNA uridine 5-carboxymethylaminomethyl modification enzyme	1	1	1	1	1	1	0	1	1	1
K03496	parA, soj; chromosome partitioning protein	5	1	1	1	3	1	2	1	1	1
K03497	parB, spo0J; ParB family transcriptional regulator, chromosome partitioning protein	5	1	1	1	2	1	1	1	1	1
K03498	trkH, trkG, ktrB, ktrD; trk/ktr system potassium uptake protein	2	1	2	1	2	2	0	1	1	1
K03499	trkA, ktrA, ktrC; trk/ktr system potassium uptake protein	3	1	4	1	2	2	1	1	1	1
K03500	rsmB, sun; 16S rRNA (cytosine967-C5)-methyltransferase [EC:2.1.1.176]	1	1	1	1	1	1	1	1	1	1
K03501	gidB, rsmG; 16S rRNA (guanine527-N7)-methyltransferase [EC:2.1.1.170]	1	1	1	1	1	1	1	1	1	1
K03502	umuC; DNA polymerase V	0	0	0	1	0	0	1	0	0	0
K03517	nadA; quinolinate synthase [EC:2.5.1.72]	0	0	1	0	1	2	1	1	1	2
K03518	coxS; aerobic carbon-monoxide dehydrogenase small subunit [EC:1.2.5.3]	4	5	21	0	0	2	1	6	3	1
K03519	coxM, cutM; aerobic carbon-monoxide dehydrogenase medium subunit [EC:1.2.5.3]	3	4	9	0	0	1	2	5	1	1
K03520	coxL, cutL; aerobic carbon-monoxide dehydrogenase large subunit [EC:1.2.5.3]	0	0	3	0	0	0	0	3	0	0
K03521	fixA, etfB; electron transfer flavoprotein beta subunit	3	1	4	0	0	0	0	4	1	1
K03522	fixB, etfA; electron transfer flavoprotein alpha subunit	3	1	4	0	0	0	2	4	1	1
K03523	bioY; biotin transport system substrate-specific component	1	1	1	0	2	1	1	1	1	1
K03524	birA; BirA family transcriptional regulator, biotin operon repressor / biotin---[acetyl-CoA-carboxylase] ligase [EC:6.3.4.15]	1	1	1	2	2	1	1	1	1	1
K03525	coaX; type III pantothenate kinase [EC:2.7.1.33]	1	1	1	1	1	1	0	1	1	1
K03526	gcpE, ispG; (E)-4-hydroxy-3-methylbut-2-enyl-diphosphate synthase [EC:1.17.7.1 1.17.7.3]	1	0	1	1	1	1	1	1	1	1
K03529	smc; chromosome segregation protein	1	1	1	1	1	1	0	1	1	1
K03530	hupB; DNA-binding protein HU-beta	1	1	1	1	1	1	1	0	0	0
K03531	ftsZ; cell division protein FtsZ	1	1	1	1	1	1	0	1	1	1
K03535	gudP; MFS transporter, ACS family, glucarate transporter	2	0	0	0	0	0	0	0	0	0
K03536	rnpA; ribonuclease P protein component [EC:3.1.26.5]	0	0	1	1	1	1	1	1	1	1
K03544	clpX, CLPX; ATP-dependent Clp protease ATP-binding subunit ClpX	1	0	1	1	1	1	1	1	1	1
K03545	tig; trigger factor	1	1	1	1	1	1	1	1	1	1
K03546	sbcC, rad50; DNA repair protein SbcC/Rad50	1	0	1	1	0	0	0	0	0	0
K03547	sbcD, mre11; DNA repair protein SbcD/Mre11	1	1	1	1	0	1	0	1	0	0
K03548	perM; putative permease	0	1	1	0	0	0	0	1	1	1
K03550	ruvA; holliday junction DNA helicase RuvA [EC:5.6.2.4]	1	1	1	1	1	1	1	1	1	1
K03551	ruvB; holliday junction DNA helicase RuvB [EC:5.6.2.4]	1	1	1	1	1	1	1	1	1	1
K03553	recA; recombination protein RecA	1	1	1	1	1	1	1	1	1	1
K03555	mutS; DNA mismatch repair protein MutS	0	1	2	1	1	1	1	1	1	1
K03556	malT; LuxR family transcriptional regulator, maltose regulon positive regulatory protein	0	0	1	0	1	0	0	0	0	0
K03563	csrA; carbon storage regulator	0	1	1	0	1	0	1	0	1	1
K03564	BCP, PRXQ, DOT5; thioredoxin-dependent peroxiredoxin [EC:1.11.1.24]	0	0	0	1	1	0	0	0	0	0
K03565	recX; regulatory protein	0	1	0	0	0	0	0	0	1	0
K03568	tldD; TldD protein	1	1	1	1	1	0	0	0	0	0
K03569	mreB; rod shape-determining protein MreB and related proteins	2	2	2	0	0	2	1	2	2	2
K03570	mreC; rod shape-determining protein MreC	1	1	1	1	1	1	1	1	1	1
K03571	mreD; rod shape-determining protein MreD	1	1	1	0	0	1	1	1	1	1

K03572	mutL; DNA mismatch repair protein MutL	1	1	1	1	1	1	1	1	1	1
K03574	mutT, NUDT15, MTH2; 8-oxo-dGTP diphosphatase [EC:3.6.1.55]	1	0	2	0	0	0	0	0	1	0
K03575	mutY; A/G-specific adenine glycosylase [EC:3.2.2.31]	0	0	1	1	1	0	0	1	0	0
K03577	acrR, smeT; TetR/AcrR family transcriptional regulator, acrAB operon repressor	0	0	1	0	0	0	0	0	0	0
K03581	recD; exodeoxyribonuclease V alpha subunit [EC:3.1.11.5]	1	1	1	0	0	0	0	0	0	0
K03584	recO; DNA repair protein RecO (recombination protein O)	1	1	1	1	1	1	1	1	1	1
K03585	acrA, mexA, adeI, smeD, mtrC, cmeA; membrane fusion protein, multidrug efflux system	0	0	0	0	0	0	1	0	0	0
K03588	ftsW, spoVE; cell division protein FtsW	2	2	2	1	1	1	1	1	1	1
K03589	ftsQ; cell division protein FtsQ	1	1	1	0	0	0	1	0	1	1
K03590	ftsA; cell division protein FtsA	1	1	1	0	0	0	0	0	0	0
K03592	pmbA; PmbA protein	1	1	1	1	1	0	0	0	0	0
K03593	mrp, NUBPL; ATP-binding protein involved in chromosome partitioning	1	2	2	1	1	1	0	0	2	2
K03594	bfr; bacterioferritin [EC:1.16.3.1]	1	1	2	1	1	0	0	0	1	1
K03595	era, ERAL1; GTPase	1	1	1	1	1	1	0	1	1	1
K03596	lepA; GTP-binding protein LepA	1	0	1	1	1	1	0	1	1	1
K03601	xseA; exodeoxyribonuclease VII large subunit [EC:3.1.11.6]	1	1	1	1	1	0	0	1	1	1
K03602	xseB; exodeoxyribonuclease VII small subunit [EC:3.1.11.6]	0	1	1	1	1	0	0	0	0	0
K03605	hyaD, hybD; hydrogenase maturation protease [EC:3.4.23.-]	1	0	1	0	0	0	1	0	1	1
K03606	wcaJ; undecaprenyl-phosphate glucose phosphotransferase [EC:2.7.8.31]	0	1	1	0	0	0	0	0	0	0
K03608	minE; cell division topological specificity factor	1	1	1	1	1	1	1	0	1	1
K03609	minD; septum site-determining protein MinD	1	1	1	1	1	1	1	0	1	1
K03610	minC; septum site-determining protein MinC	2	2	2	1	1	1	2	0	2	2
K03612	rnfG; H+/Na+-translocating ferredoxin:NAD+ oxidoreductase subunit G	0	0	1	0	1	0	0	1	0	1
K03613	rnfE; H+/Na+-translocating ferredoxin:NAD+ oxidoreductase subunit E	0	0	1	0	1	0	0	1	0	0
K03614	rnfD; H+/Na+-translocating ferredoxin:NAD+ oxidoreductase subunit D [EC:7.1.1.11 7.2.1.2]	0	0	1	0	1	0	0	1	0	0
K03615	rnfC; H+/Na+-translocating ferredoxin:NAD+ oxidoreductase subunit C [EC:7.1.1.11 7.2.1.2]	0	0	1	0	1	0	0	1	0	0
K03616	rnfB; H+/Na+-translocating ferredoxin:NAD+ oxidoreductase subunit B [EC:7.1.1.11 7.2.1.2]	0	0	1	0	1	0	0	1	0	0
K03617	rnfA; H+/Na+-translocating ferredoxin:NAD+ oxidoreductase subunit A	0	0	1	0	1	0	0	1	0	0
K03620	hyaC; Ni/Fe-hydrogenase 1 B-type cytochrome subunit	0	0	1	0	0	0	0	0	0	0
K03621	plsX; phosphate acyltransferase [EC:2.3.1.274]	1	1	1	1	1	1	1	1	1	1
K03624	greA; transcription elongation factor GreA	1	1	1	1	1	1	1	1	1	1
K03625	nusB; transcription antitermination protein NusB	1	1	1	1	1	1	1	1	1	1
K03628	rho; transcription termination factor Rho	1	1	1	0	0	0	0	0	0	0
K03629	recF; DNA replication and repair protein RecF	1	1	1	1	1	1	1	1	1	1
K03630	radC; DNA repair protein RadC	4	1	2	1	1	1	1	1	1	1
K03631	recN; DNA repair protein RecN (Recombination protein N)	1	1	1	1	1	1	1	1	1	1
K03635	MOCS2B, moaE; molybdopterin synthase catalytic subunit [EC:2.8.1.12]	1	0	1	0	0	0	0	0	0	0
K03636	moaD, cysO; sulfur-carrier protein	0	0	0	0	0	0	0	0	1	1
K03637	moaC, CNX3; cyclic pyranopterin monophosphate synthase [EC:4.6.1.17]	1	1	1	0	0	1	1	1	1	1
K03639	moaA, CNX2; GTP 3',8-cyclase [EC:4.1.99.22]	1	1	2	0	0	1	1	1	1	1
K03641	tolB; TolB protein	0	0	1	0	0	0	0	0	0	0
K03644	lipA, LIAS, LIP1, LIP5; lipoyl synthase [EC:2.8.1.8]	0	0	1	0	0	0	0	0	0	0
K03648	UNG, UDG; uracil-DNA glycosylase [EC:3.2.2.27]	1	1	0	0	0	0	0	0	0	0
K03649	mug; double-stranded uracil-DNA glycosylase [EC:3.2.2.28]	0	0	0	1	1	0	1	1	1	1
K03650	mnmE, trmE, MSS1; tRNA modification GTPase [EC:3.6.-.-]	1	1	1	1	1	1	0	1	1	1
K03652	MPG; DNA-3-methyladenine glycosylase [EC:3.2.2.21]	1	1	0	0	0	1	0	0	0	0
K03654	recQ; ATP-dependent DNA helicase RecQ [EC:5.6.2.4]	2	0	0	1	1	0	0	0	0	0
K03655	recG; ATP-dependent DNA helicase RecG [EC:5.6.2.4]	0	0	1	1	1	1	0	1	1	1
K03657	uvrD, pcrA; ATP-dependent DNA helicase UvrD/PcrA [EC:5.6.2.4]	2	1	4	1	1	1	1	1	1	1
K03664	smpB; SsrA-binding protein	1	1	1	1	1	1	0	1	1	1
K03665	hflX; GTPase	1	1	1	0	0	1	1	1	1	1
K03666	hfq; host factor-I protein	1	1	1	1	1	1	0	1	1	1
K03667	hslU; ATP-dependent HslUV protease ATP-binding subunit HslU	1	1	1	1	1	1	1	1	1	1
K03671	trxA; thioredoxin 1	4	3	6	1	2	2	1	3	3	3
K03676	grxC, GLRX, GLRX2; glutaredoxin 3	0	0	1	0	0	0	0	0	0	0
K03685	rnc, DROSHA, RNT1; ribonuclease III [EC:3.1.26.3]	1	1	1	1	1	1	0	1	1	1
K03686	dnaJ; molecular chaperone DnaJ	2	2	2	2	2	1	1	1	1	1
K03687	GRPE; molecular chaperone GrpE	1	1	1	0	1	1	1	1	1	1
K03688	ubiB, aarF; ubiquinone biosynthesis protein	1	0	1	0	0	0	0	1	0	0
K03695	clpB; ATP-dependent Clp protease ATP-binding subunit ClpB	1	1	1	0	0	0	0	1	0	0
K03696	clpC; ATP-dependent Clp protease ATP-binding subunit ClpC	1	1	1	1	1	1	1	1	1	1
K03698	cbf, cbf1; 3'-5' exoribonuclease [EC:3.1.-.-]	1	1	1	0	0	0	1	0	0	0
K03699	tlyC; magnesium and cobalt exporter, CNNM family	1	0	1	1	1	0	0	0	1	1
K03700	recU; recombination protein U	0	0	1	0	0	0	0	0	1	0
K03701	uvrA; excinuclease ABC subunit A	1	1	1	1	1	1	1	1	1	1
K03702	uvrB; excinuclease ABC subunit B	1	1	1	1	1	1	1	1	1	1
K03703	uvrC; excinuclease ABC subunit C	2	0	1	1	1	1	0	1	1	1
K03704	cspA; cold shock protein	2	2	3	0	1	2	1	1	2	2
K03705	hrcA; heat-inducible transcriptional repressor	0	1	1	1	1	1	1	1	1	1
K03706	codY; transcriptional pleiotropic repressor	1	1	1	0	0	0	1	1	1	1
K03707	tenA; thiaminase (transcriptional activator TenA) [EC:3.5.99.2]	1	0	0	0	0	0	0	2	0	0
K03708	ctsR; transcriptional regulator of stress and heat shock response	1	1	1	1	1	1	1	1	1	1
K03709	troR; DtxR family transcriptional regulator, Mn-dependent transcriptional regulator	1	1	2	0	2	2	1	2	1	1
K03710	K03710; GntR family transcriptional regulator	0	0	13	0	1	0	0	0	0	0
K03711	fur, zur, furB; Fur family transcriptional regulator, ferric uptake regulator	1	1	1	1	1	0	0	1	3	2
K03716	splB; spore photoproduct lyase [EC:4.1.99.14]	0	1	1	0	0	0	0	0	1	1
K03718	asnC; Lrp/AsnC family transcriptional regulator, regulator for asnA, asnC and gidA	1	1	1	1	1	0	0	0	0	0
K03719	lrp; Lrp/AsnC family transcriptional regulator, leucine-responsive regulatory protein	1	0	0	0	0	0	0	0	0	0
K03721	tyrR; transcriptional regulator of aroF, aroG, tyrA and aromatic amino acid transport	6	6	18	6	5	0	0	0	0	0
K03722	dinG; ATP-dependent DNA helicase DinG [EC:5.6.2.3]	1	1	1	1	1	1	1	1	1	1
K03723	mfd; transcription-repair coupling factor (superfamily II helicase) [EC:5.6.2.4]	1	1	1	1	1	1	1	1	1	1
K03733	xerC; integrase/recombinase XerC	1	1	1	1	1	1	1	1	1	1
K03734	apbE; FAD:protein FMN transferase [EC:2.7.1.180]	1	1	1	0	2	0	0	1	0	1
K03735	eutB; ethanolamine ammonia-lyase large subunit [EC:4.3.1.7]	1	2	2	2	2	0	0	0	0	0
K03736	eutC; ethanolamine ammonia-lyase small subunit [EC:4.3.1.7]	1	2	2	2	2	0	0	0	0	0
K03737	por, nifJ; pyruvate-ferredoxin/flavodoxin oxidoreductase [EC:1.2.7.1 1.2.7.-]	1	1	1	0	0	1	1	1	1	1
K03738	aor; aldehyde:ferredoxin oxidoreductase [EC:1.2.7.5]	1	4	1	0	0	0	0	0	5	4
K03741	arsC; arsenate reductase (thioredoxin) [EC:1.20.4.4]	0	0	0	0	0	0	0	0	1	1
K03742	pncC; nicotinamide-nucleotide amidase [EC:3.5.1.42]	0	0	0	0	0	0	0	1	0	1
K03743	pncC; nicotinamide-nucleotide amidase [EC:3.5.1.42]	1	1	1	1	1	1	1	1	1	0
K03744	lemA; LemA protein	2	2	1	0	1	1	1	1	1	1
K03748	sanA; SanA protein	0	0	1	0	0	0	0	0	0	0
K03750	moeA; molybdopterin molybdotransferase [EC:2.10.1.1]	2	1	3	0	0	1	2	0	1	1
K03752	mobA; molybdenum cofactor guanylyltransferase [EC:2.7.7.77]	1	1	2	1	0	0	1	0	0	0
K03753	mobB; molybdopterin-guanine dinucleotide biosynthesis adapter protein	1	1	1	0	0	0	1	0	3	3
K03762	proP; MFS transporter, MHS family, proline/betaine transporter	0	0	2	0	0	0	0	0	0	0
K03763	polC; DNA polymerase III subunit alpha, Gram-positive type [EC:2.7.7.7]	1	1	1	0	0	0	0	0	0	0
K03767	PPIA; peptidyl-prolyl cis-trans isomerase A (cyclophilin A) [EC:5.2.1.8]	0	0	1	0	0	0	0	0	0	0
K03768	PPIB, ppiB; peptidyl-prolyl cis-trans isomerase B (cyclophilin B) [EC:5.2.1.8]	1	0	1	2	2	1	0	1	1	1
K03769	ppiC; peptidyl-prolyl cis-trans isomerase C [EC:5.2.1.8]	1	1	1	1	1	0	1	0	1	0
K03781	katE, CAT, catB, srpA; catalase [EC:1.11.1.6]	2	1	1	0	2	0	0	0	0	0
K03782	katG; catalase-peroxidase [EC:1.11.1.21]	0	0	1	0	0	0	0	0	0	0
K03783	punA, PNP; purine-nucleoside phosphorylase [EC:2.4.2.1]	0	0	0	0	0	1	1	1	0	0
K03785	aroD; 3-dehydroquinate dehydratase I [EC:4.2.1.10]	0	0	1	0	0	0	0	0	0	0
K03786	aroQ, qutE; 3-dehydroquinate dehydratase II [EC:4.2.1.10]	1	1	1	1	2	2	0	1	1	1
K03787	surE; 5'/3'-nucleotidase [EC:3.1.3.5 3.1.3.6]	1	1	1	1	1	1	1	1	1	1
K03789	rimI; [ribosomal protein S18]-alanine N-acetyltransferase [EC:2.3.1.266]	1	1	2	0	0	1	1	1	1	1
K03790	rimJ; [ribosomal protein S5]-alanine N-acetyltransferase [EC:2.3.1.267]	0	0	1	0	1	0	0	0	0	0
K03795	cbiX; sirohydrochlorin cobaltochelatase [EC:4.99.1.3]	1	1	1	0	0	0	1	0	0	0
K03797	E3.4.21.102, prc, ctpA; carboxyl-terminal processing protease [EC:3.4.21.102]	2	2	2	1	1	2	0	2	2	2
K03798	ftsH, hflB; cell division protease FtsH [EC:3.4.24.-]	2	1	1	2	1	1	1	1	1	1
K03800	lplA, lplJ, lipL1; lipoate---protein ligase [EC:6.3.1.20]	2	1	2	1	0	0	1	1	1	2
K03801	lipB; lipoyl(octanoyl) transferase [EC:2.3.1.181]	1	1	2	0	0	0	0	0	0	0
K03802	cphA; cyanophycin synthetase [EC:6.3.2.29 6.3.2.30]	1	1	1	0	0	0	0	0	0	0
K03803	rseC; sigma-E factor negative regulatory protein RseC	0	0	1	0	1	0	0	1	0	0
K03813	modD; molybdenum transport protein [EC:2.4.2.-]	0	0	1	0	0	0	0	0	0	0
K03816	xpt; xanthine phosphoribosyltransferase [EC:2.4.2.22]	1	1	1	1	1	0	1	0	0	0
K03820	lnt; apolipoprotein N-acyltransferase [EC:2.3.1.269]	0	0	0	0	0	1	1	0	0	0
K03823	pat, bar; phosphinothricin acetyltransferase [EC:2.3.1.183]	0	0	1	1	0	0	0	0	2	1
K03824	yhbS; putative acetyltransferase [EC:2.3.1.-]	0	0	0	0	0	0	0	0	0	1
K03827	yjaB; putative acetyltransferase [EC:2.3.1.-]	0	0	0	0	0	0	0	0	0	1
K03830	yafP; putative acetyltransferase [EC:2.3.1.-]	0	0	1	0	0	0	0	0	0	0
K03831	mogA; molybdopterin adenylyltransferase [EC:2.7.7.75]	1	1	1	0	0	0	0	0	0	0
K03833	selB, EEFSEC; selenocysteine-specific elongation factor	1	1	1	0	0	1	1	1	1	1
K03839	fldA, nifF, isiB; flavodoxin I	0	0	1	1	1	0	0	0	0	0
K03851	tpa; taurine-pyruvate aminotransferase [EC:2.6.1.77]	1	1	0	0	0	0	0	0	0	0

K03852	xsc; sulfoacetaldehyde acetyltransferase [EC:2.3.3.15]	1	0	0	0	0	0	0	0	0	0
K03855	fixX; ferredoxin like protein	3	1	2	0	0	0	2	1	0	0
K03856	AROA2, aroA; 3-deoxy-7-phosphoheptulonate synthase [EC:2.5.1.54]	2	2	3	1	1	2	1	2	1	1
K03892	arsR; ArsR family transcriptional regulator, arsenate/arsenite/antimonite-responsive transcriptional repressor	1	1	3	1	2	2	2	1	2	3
K03924	moxR; MoxR-like ATPase [EC:3.6.3.-]	1	0	3	0	1	0	0	0	0	0
K03925	mraZ; transcriptional regulator MraZ	1	1	1	1	1	1	0	1	1	1
K03969	pspA; phage shock protein A	1	0	0	0	0	0	0	0	0	0
K03972	pspE; phage shock protein E	0	0	0	0	1	0	0	0	0	0
K03973	pspC; phage shock protein C	0	0	0	1	0	1	1	1	1	1
K03975	dedA; membrane-associated protein	1	1	0	0	1	0	1	1	0	0
K03976	ybaK, ebsC; Cys-tRNA(Pro)/Cys-tRNA(Cys) deacylase [EC:3.1.1.-]	1	1	2	1	1	1	1	0	0	0
K03977	engA, der; GTPase	1	1	1	1	1	1	1	1	1	1
K03978	engB; GTP-binding protein	1	1	1	1	1	1	1	1	1	1
K03979	obgE, cgtA, MTG2; GTPase [EC:3.6.5.-]	1	1	1	1	1	1	1	1	1	1
K03980	murJ, mviN; putative peptidoglycan lipid II flippase	2	2	2	2	3	2	1	2	2	2
K04019	eutA; ethanolamine utilization protein EutA	1	1	1	1	1	0	0	0	0	0
K04023	eutH; ethanolamine transporter	0	1	2	3	4	0	0	0	0	0
K04024	eutJ; ethanolamine utilization protein EutJ	1	2	2	1	1	0	0	0	0	0
K04026	eutL; ethanolamine utilization protein EutL	1	1	1	1	1	0	0	0	0	0
K04027	eutM; ethanolamine utilization protein EutM	4	3	4	1	1	0	0	0	0	0
K04028	eutN; ethanolamine utilization protein EutN	1	2	2	0	1	0	0	0	0	0
K04029	eutP; ethanolamine utilization protein EutP	1	2	2	2	1	0	0	0	0	0
K04030	eutQ; ethanolamine utilization protein EutQ	1	1	1	0	0	0	0	0	0	0
K04031	eutS; ethanolamine utilization protein EutS	1	2	2	1	1	0	0	0	0	0
K04032	eutT; ethanolamine utilization cobalamin adenosyltransferase [EC:2.5.1.154]	1	1	1	1	1	0	0	0	0	0
K04034	bchE; anaerobic magnesium-protoporphyrin IX monomethyl ester cyclase [EC:1.21.98.3]	1	1	1	0	1	0	1	1	1	1
K04041	fbp3; fructose-1,6-bisphosphatase III [EC:3.1.3.11]	1	0	0	0	0	0	0	0	0	0
K04042	glmU; bifunctional UDP-N-acetylglucosamine pyrophosphorylase / glucosamine-1-phosphate N-acetyltransferase [EC:2.7.7.23 2.3.1.157]	1	1	1	1	1	1	1	1	1	1
K04043	dnaK, HSPA9; molecular chaperone DnaK	3	1	2	1	1	1	1	1	1	1
K04061	flhB2; flagellar biosynthesis protein	0	1	1	0	1	0	1	0	0	0
K04066	priA; primosomal protein N' (replication factor Y) (superfamily II helicase) [EC:5.6.2.4]	1	1	1	1	1	1	1	1	1	1
K04068	nrdG; anaerobic ribonucleoside-triphosphate reductase activating protein [EC:1.97.1.4]	1	1	1	1	0	1	1	0	1	1
K04069	pflA, pflC, pflE; pyruvate formate lyase activating enzyme [EC:1.97.1.4]	6	3	5	1	2	1	2	1	1	1
K04070	pflX; putative pyruvate formate lyase activating enzyme [EC:1.97.1.4]	0	1	0	0	0	0	0	1	0	0
K04072	adhE; acetaldehyde dehydrogenase / alcohol dehydrogenase [EC:1.2.1.10 1.1.1.1]	1	1	1	0	0	0	0	0	0	0
K04075	tilS, mesJ; tRNA(Ile)-lysidine synthase [EC:6.3.4.19]	0	1	1	1	1	1	1	1	1	1
K04076	lonB; ATP-dependent Lon protease [EC:3.4.21.53]	1	0	1	0	0	0	0	0	0	0
K04077	groEL, HSPD1; chaperonin GroEL [EC:5.6.1.7]	1	1	1	1	1	1	1	1	1	1
K04078	groES, HSPE1; chaperonin GroES	1	1	1	1	1	1	0	1	1	1
K04079	HSP90A, htpG; molecular chaperone HtpG	1	1	1	1	1	0	0	0	1	1
K04083	hslO; molecular chaperone Hsp33	1	1	1	0	0	1	1	1	0	0
K04085	tusA, sirA; tRNA 2-thiouridine synthesizing protein A [EC:2.8.1.-]	3	0	5	0	1	0	3	2	2	2
K04094	trmFO, gid; methylenetetrahydrofolate--tRNA-(uracil-5-)-methyltransferase [EC:2.1.1.74]	1	1	1	1	1	1	1	1	1	1
K04096	smf; DNA processing protein	1	1	1	1	1	1	1	1	1	1
K04102	pht5; 4,5-dihydroxyphthalate decarboxylase [EC:4.1.1.55]	0	0	1	0	0	0	0	0	0	0
K04108	hcrA, hbaC; 4-hydroxybenzoyl-CoA reductase subunit alpha [EC:1.1.7.1]	0	0	0	0	0	0	0	0	1	0
K04114	bcrA, badF; benzoyl-CoA reductase subunit A [EC:1.3.7.8]	2	2	2	0	0	0	0	1	1	1
K04477	ycdX; putative hydrolase	1	1	2	0	0	0	0	0	0	0
K04480	mtaB; methanol---5-hydroxybenzimidazolylcobamide Co-methyltransferase [EC:2.1.1.90]	1	0	1	1	1	0	0	0	0	0
K04485	radA, sms; DNA repair protein RadA/Sms	1	1	1	1	1	1	1	1	1	1
K04486	E3.1.3.15B; histidinol-phosphatase (PHP family) [EC:3.1.3.15]	2	2	4	1	1	1	1	2	2	2
K04487	iscS, NFS1; cysteine desulfurase [EC:2.8.1.7]	2	2	3	4	2	1	1	2	2	3
K04488	iscU, nifU; nitrogen fixation protein NifU and related proteins	2	2	2	1	1	0	1	2	2	3
K04516	AROA1, aroA; chorismate mutase [EC:5.4.99.5]	0	0	0	0	1	0	0	0	0	0
K04517	tyrA2; prephenate dehydrogenase [EC:1.3.1.12]	1	0	1	1	1	1	1	1	1	1
K04518	pheA2; prephenate dehydratase [EC:4.2.1.51]	1	1	1	0	0	0	0	0	0	0
K04562	flhG, fleN; flagellar biosynthesis protein FlhG	0	0	1	0	0	0	0	0	0	0
K04564	SOD2; superoxide dismutase, Fe-Mn family [EC:1.15.1.1]	0	0	1	0	1	1	0	1	1	1
K04565	SOD1; superoxide dismutase, Cu-Zn family [EC:1.15.1.1]	1	1	1	0	0	0	1	0	1	1
K04567	KARS, lysS; lysyl-tRNA synthetase, class II [EC:6.1.1.6]	1	1	1	1	1	1	1	1	1	1
K04651	hypA, hybF; hydrogenase nickel incorporation protein HypA/HybF	0	0	1	1	1	0	1	0	1	1
K04652	hypB; hydrogenase nickel incorporation protein HypB	0	0	1	1	1	0	1	1	1	1
K04653	hypC; hydrogenase expression/formation protein HypC	2	0	1	1	1	0	2	0	1	1
K04654	hypD; hydrogenase expression/formation protein HypD	1	0	1	1	1	0	1	0	1	1
K04655	hypE; hydrogenase expression/formation protein HypE	1	0	1	1	1	0	0	0	1	1
K04656	hypF; hydrogenase maturation protein HypF	1	0	1	1	1	0	1	0	1	1
K04719	bluB; 5,6-dimethylbenzimidazole synthase [EC:1.13.11.79]	0	1	1	0	0	0	0	0	0	0
K04720	cobD; threonine-phosphate decarboxylase [EC:4.1.1.81]	1	1	1	1	1	0	1	0	0	0
K04749	rsbV; anti-sigma B factor antagonist	0	0	4	0	0	0	0	0	0	1
K04750	phnB; PhnB protein	1	0	3	0	0	0	0	0	0	0
K04751	glnB; nitrogen regulatory protein P-II 1	0	0	0	0	1	0	0	2	0	0
K04752	glnK; nitrogen regulatory protein P-II 2	0	0	1	0	0	0	0	0	0	0
K04757	rsbW; serine/threonine-protein kinase RsbW [EC:2.7.11.1]	0	0	2	0	0	0	0	0	0	1
K04758	feoA; ferrous iron transport protein A	2	1	4	0	4	4	1	3	4	4
K04759	feoB; ferrous iron transport protein B	2	1	3	1	3	3	0	3	2	3
K04761	oxyR; LysR family transcriptional regulator, hydrogen peroxide-inducible genes activator	0	0	1	0	0	0	0	0	0	0
K04763	xerD; integrase/recombinase XerD	2	1	4	1	1	1	0	1	2	1
K04766	acuA; acetoin utilization protein AcuA [EC:2.3.1.-]	1	0	0	0	0	0	0	0	0	0
K04767	acuB; acetoin utilization protein AcuB	2	1	1	0	0	0	0	0	0	0
K04768	acuC; acetoin utilization protein AcuC	1	0	0	0	0	0	0	1	0	0
K04769	spoVT; AbrB family transcriptional regulator, stage V sporulation protein T	1	1	1	1	1	1	1	1	1	1
K04771	degP, htrA; serine protease Do [EC:3.4.21.107]	2	3	3	2	2	1	1	1	1	2
K04773	sppA; protease IV [EC:3.4.21.-]	0	0	0	0	0	0	0	0	1	1
K04780	dhbF; glyine---[glycyl-carrier protein] ligase [EC:6.2.1.66]	0	0	0	1	0	0	0	0	0	0
K04835	mal; methylaspartate ammonia-lyase [EC:4.3.1.2]	0	0	1	0	0	0	0	0	0	0
K04940	odh; opine dehydrogenase [EC:1.5.1.28]	0	0	3	0	0	0	0	0	0	0
K05020	opuD, betL; glycine betaine transporter	2	0	8	2	4	0	0	0	0	0
K05245	caiT; L-carnitine/gamma-butyrobetaine antiporter	1	0	1	1	0	0	0	0	0	0
K05297	rubB, alkT; rubredoxin---NAD+ reductase [EC:1.18.1.1]	0	0	0	0	0	0	1	0	1	0
K05299	fdhA; formate dehydrogenase (NADP+) alpha subunit [EC:1.17.1.10]	0	0	1	0	0	0	0	0	0	0
K05306	phnX; phosphonoacetaldehyde hydrolase [EC:3.11.1.1]	1	0	1	0	0	0	0	0	0	0
K05337	fer; ferredoxin	0	0	0	0	1	0	0	1	0	0
K05339	lrgB; holin-like protein LrgB	0	0	0	0	0	0	0	1	0	0
K05346	deoR; deoxyribonucleoside regulator	0	0	2	0	0	0	0	0	0	0
K05364	pbpA; penicillin-binding protein A	1	1	1	1	1	0	0	0	0	0
K05366	mrcA; penicillin-binding protein 1A [EC:2.4.1.129 3.4.16.4]	1	1	1	0	0	1	0	1	1	1
K05396	dcyD; D-cysteine desulfhydrase [EC:4.4.1.15]	0	0	1	0	0	0	0	1	0	0
K05515	mrdA; penicillin-binding protein 2 [EC:3.4.16.4]	2	2	2	2	2	2	1	2	2	2
K05516	cbpA; curved DNA-binding protein	1	0	1	0	0	0	0	1	0	0
K05521	draG; ADP-ribosyl-[dinitrogen reductase] hydrolase [EC:3.2.2.24]	0	0	1	0	0	0	0	0	0	0
K05540	dusB; tRNA-dihydrouridine synthase B [EC:1.-.-.-]	1	1	1	1	1	1	1	1	1	1
K05548	benK; MFS transporter, AAHS family, benzoate transport protein	0	0	1	0	0	0	0	0	0	0
K05565	mnhA, mrpA; multicomponent Na+:H+ antiporter subunit A	0	0	0	0	0	0	1	0	1	1
K05566	mnhB, mrpB; multicomponent Na+:H+ antiporter subunit B	1	0	0	0	0	0	0	0	0	0
K05567	mnhC, mrpC; multicomponent Na+:H+ antiporter subunit C	1	0	0	0	0	0	0	0	0	0
K05568	mnhD, mrpD; multicomponent Na+:H+ antiporter subunit D	1	0	0	0	0	0	2	0	1	1
K05569	mnhE, mrpE; multicomponent Na+:H+ antiporter subunit E	1	0	0	0	0	0	0	0	0	0
K05570	mnhF, mrpF; multicomponent Na+:H+ antiporter subunit F	1	0	0	0	0	0	0	0	0	0
K05571	mnhG, mrpG; multicomponent Na+:H+ antiporter subunit G	1	0	0	0	0	0	0	0	0	0
K05592	deaD, cshA; ATP-dependent RNA helicase DeaD [EC:3.6.4.13]	2	0	1	0	1	2	1	1	0	1
K05601	hcp; hydroxylamine reductase [EC:1.7.99.1]	2	1	1	1	2	0	1	1	1	1
K05606	MCEE, epi; methylmalonyl-CoA/ethylmalonyl-CoA epimerase [EC:5.1.99.1]	0	1	2	0	0	0	1	0	1	1
K05770	TSPO, BZRP; translocator protein	0	0	0	1	0	1	1	0	0	0
K05772	tupA, vupA; tungstate transport system substrate-binding protein	1	1	0	0	0	0	1	0	2	1
K05773	tupB, vupB; tungstate transport system permease protein	1	1	0	0	0	0	1	0	1	1
K05780	phnL; alpha-D-ribose 1-methylphosphonate 5-triphosphate synthase subunit PhnL [EC:2.7.8.37]	0	0	1	0	0	0	0	0	0	0
K05781	phnK; putative phosphonate transport system ATP-binding protein	0	0	1	0	0	0	0	0	0	0
K05786	rarD; chloramphenicol-sensitive protein RarD	0	0	1	0	0	0	0	0	0	0
K05794	terC; tellurite resistance protein TerC	0	0	0	0	0	0	1	0	0	1
K05799	pdhR; GntR family transcriptional regulator, transcriptional repressor for pyruvate dehydrogenase complex	2	1	7	0	0	0	2	0	0	0
K05808	yhbH; putative sigma-54 modulation protein	1	1	1	1	1	1	1	1	1	1
K05810	LACC1, yfiH; purine-nucleoside/S-methyl-5'-thioadenosine phosphorylase / adenosine deaminase [EC:2.4.2.1 2.4.2.28 3.5.4.4]	1	1	1	1	1	0	0	1	0	0
K05817	hcaR; LysR family transcriptional regulator, hca operon transcriptional activator	0	0	1	0	0	0	0	0	0	0
K05825	LYSN; 2-aminoadipate transaminase [EC:2.6.1.-]	0	0	1	0	0	0	0	0	0	0
K05832	K05832; putative tryptophan/tyrosine transport system permease protein	1	1	1	1	1	0	0	0	0	0
K05833	K05833; putative tryptophan/tyrosine transport system ATP-binding protein	1	1	1	1	1	0	0	0	0	0

K05837	rodA, mrdB; rod shape determining protein RodA	2	1	1	1	1	1	0	1	1	1
K05844	rimK; ribosomal protein S6--L-glutamate ligase [EC:6.3.2.-]	0	0	1	0	0	0	0	0	0	0
K05845	opuC; osmoprotectant transport system substrate-binding protein	0	2	2	0	0	0	0	0	0	0
K05846	opuBD; osmoprotectant transport system permease protein	0	3	4	0	1	0	0	0	0	0
K05847	opuA; osmoprotectant transport system ATP-binding protein [EC:7.6.2.9]	0	2	2	0	1	0	0	0	0	0
K05882	E1.1.1.91; aryl-alcohol dehydrogenase (NADP+) [EC:1.1.1.91]	0	0	0	1	0	0	0	0	0	0
K05884	comC; L-2-hydroxycarboxylate dehydrogenase (NAD+) [EC:1.1.1.337]	0	0	2	1	1	0	1	0	0	0
K05895	cobK-cbiJ; precorrin-6A/cobalt-precorrin-6A reductase [EC:1.3.1.54 1.3.1.106]	1	1	1	1	1	0	1	0	0	0
K05896	scpA; segregation and condensation protein A	1	0	1	1	1	1	1	1	1	1
K05898	kstD; 3-oxosteroid 1-dehydrogenase [EC:1.3.99.4]	1	0	0	0	0	0	0	0	0	0
K05919	dfx; superoxide reductase [EC:1.15.1.2]	1	1	1	1	1	1	0	1	0	0
K05921	hpaG; 5-oxopent-3-ene-1,2,5-tricarboxylate decarboxylase / 2-hydroxyhepta-2,4-diene-1,7-dioate isomerase [EC:4.1.1.68 5.3.3.-]	0	0	1	1	1	0	1	0	0	0
K05934	cobJ, cbiH; precorrin-3B C17-methyltransferase / cobalt-factor III methyltransferase [EC:2.1.1.131 2.1.1.272]	1	1	1	1	1	0	1	0	0	0
K05936	cobM, cbiF; precorrin-4/cobalt-precorrin-4 C11-methyltransferase [EC:2.1.1.133 2.1.1.271]	2	1	1	1	1	0	1	0	0	0
K05937	K05937; uncharacterized protein	2	0	0	0	0	0	0	0	0	0
K05946	tagA, tarA; N-acetylglucosaminyldiphosphoundecaprenol N-acetyl-beta-D-mannosaminyltransferase [EC:2.4.1.187]	2	0	1	0	1	1	1	1	1	1
K05964	citX; holo-ACP synthase [EC:2.7.7.61]	0	0	1	0	0	0	0	0	0	0
K05966	citG; triphosphoribosyl-dephospho-CoA synthase [EC:2.4.2.52]	0	0	2	0	0	0	0	0	0	0
K05967	K05967; uncharacterized protein	0	0	0	0	0	0	0	1	0	0
K05979	comB; 2-phosphosulfolactate phosphatase [EC:3.1.3.71]	0	0	1	0	0	0	1	1	1	1
K06001	trpB; tryptophan synthase beta chain [EC:4.2.1.20]	1	1	1	0	1	1	1	2	1	1
K06012	gpr; spore protease [EC:3.4.24.78]	1	1	1	1	1	1	0	1	1	1
K06015	E3.5.1.81; N-acyl-D-amino-acid deacylase [EC:3.5.1.81]	1	0	2	0	0	0	0	1	0	0
K06016	pydC; beta-ureidopropionase / N-carbamoyl-L-amino-acid hydrolase [EC:3.5.1.6 3.5.1.87]	0	1	0	0	0	0	0	0	0	0
K06019	ppaX; pyrophosphatase PpaX [EC:3.6.1.1]	1	1	1	1	1	1	1	1	1	1
K06020	ettA; energy-dependent translational throttle protein EttA	0	1	0	0	0	0	0	0	0	0
K06024	scpB; segregation and condensation protein B	1	1	1	1	1	1	1	1	1	1
K06027	NSF, SEC18; vesicle-fusing ATPase [EC:3.6.4.6]	1	1	1	0	0	0	0	0	0	0
K06034	comD; sulfopyruvate decarboxylase subunit alpha [EC:4.1.1.79]	0	0	1	0	0	0	0	0	0	0
K06042	cobH-cbiC; precorrin-8X/cobalt-precorrin-8 methylmutase [EC:5.4.99.61 5.4.99.60]	1	1	1	1	1	0	0	0	0	0
K06075	slyA; MarR family transcriptional regulator, transcriptional regulator for hemolysin	0	0	1	0	0	0	0	0	0	0
K06121	dhbC; glycerol dehydratase medium subunit [EC:4.2.1.30]	0	1	0	0	0	0	0	0	0	0
K06131	clsA_B; cardiolipin synthase A/B [EC:2.7.8.-]	2	1	2	1	1	1	0	1	1	1
K06133	LYS5, acpT; 4'-phosphopantetheinyl transferase [EC:2.7.8.-]	1	0	0	0	0	0	0	1	1	0
K06140	rnk; regulator of nucleoside diphosphate kinase	1	1	0	0	0	0	0	0	0	0
K06147	ABCB-BAC; ATP-binding cassette, subfamily B, bacterial	8	1	6	4	4	2	0	2	2	2
K06148	ABCC-BAC; ATP-binding cassette, subfamily C, bacterial	0	1	5	0	0	0	0	0	0	0
K06149	uspA; universal stress protein A	5	0	1	0	0	0	0	2	1	1
K06153	bacA; undecaprenyl-diphosphatase [EC:3.6.1.27]	1	1	1	1	3	1	1	1	1	1
K06158	ABCF3; ATP-binding cassette, subfamily F, member 3	2	1	3	1	1	0	2	1	1	1
K06162	phnM; alpha-D-ribose 1-methylphosphonate 5-triphosphate diphosphatase [EC:3.6.1.63]	0	0	1	0	0	0	0	0	0	0
K06163	phnJ; alpha-D-ribose 1-methylphosphonate 5-phosphate C-P lyase [EC:4.7.1.1]	0	0	1	0	0	0	0	0	0	0
K06164	phnI; alpha-D-ribose 1-methylphosphonate 5-triphosphate synthase subunit PhnI [EC:2.7.8.37]	0	0	1	0	0	0	0	0	0	0
K06165	phnH; alpha-D-ribose 1-methylphosphonate 5-triphosphate synthase subunit PhnH [EC:2.7.8.37]	0	0	1	0	0	0	0	0	0	0
K06166	phnG; alpha-D-ribose 1-methylphosphonate 5-triphosphate synthase subunit PhnG [EC:2.7.8.37]	0	0	1	0	0	0	0	0	0	0
K06167	phnP; phosphoribosyl 1,2-cyclic phosphate phosphodiesterase [EC:3.1.4.55]	0	0	1	0	0	0	0	0	0	0
K06168	miaB; tRNA-2-methylthio-N6-dimethylallyladenosine synthase [EC:2.8.4.3]	1	1	1	0	1	1	1	1	1	1
K06173	truA, PUS1; tRNA pseudouridine38-40 synthase [EC:5.4.99.12]	1	1	1	1	1	1	1	1	1	1
K06178	rluB; 23S rRNA pseudouridine2605 synthase [EC:5.4.99.22]	1	1	1	1	1	1	1	1	1	1
K06180	rluD; 23S rRNA pseudouridine1911/1915/1917 synthase [EC:5.4.99.23]	2	1	2	2	2	1	1	2	2	1
K06187	recR; recombination protein RecR	1	1	1	1	1	1	1	1	1	1
K06188	aqpZ; aquaporin Z	0	0	0	1	0	0	0	0	0	0
K06191	nrdH; glutaredoxin-like protein NrdH	1	0	1	0	0	0	0	0	0	0
K06194	nlpD; lipoprotein NlpD	0	0	0	0	0	0	0	0	1	0
K06196	ccdA; cytochrome c-type biogenesis protein	1	1	1	1	0	1	1	1	1	1
K06199	crcB, FEX; fluoride exporter	1	0	1	0	0	0	1	0	2	2
K06200	cstA; carbon starvation protein	0	0	2	0	0	0	0	0	0	0
K06206	sfsA; sugar fermentation stimulation protein A	1	1	1	2	1	0	0	0	0	0
K06207	typA, bipA; GTP-binding protein	1	1	1	1	1	1	1	1	2	1
K06208	aroH; chorismate mutase [EC:5.4.99.5]	1	1	1	0	0	0	0	0	0	0
K06209	pheB; chorismate mutase [EC:5.4.99.5]	1	1	1	0	0	0	0	0	0	0
K06213	mgtE; magnesium transporter	1	0	0	1	1	0	0	0	0	0
K06215	pdxS, pdx1; pyridoxal 5'-phosphate synthase pdxS subunit [EC:4.3.3.6]	0	0	2	0	1	1	1	1	0	0
K06217	phoH, phoL; phosphate starvation-inducible protein PhoH and related proteins	1	1	1	1	1	1	1	1	1	1
K06218	relE, stbE; mRNA interferase RelE/StbE	2	0	2	1	0	0	0	0	0	0
K06221	dkgA; 2,5-diketo-D-gluconate reductase A [EC:1.1.1.346]	1	0	1	3	0	0	0	0	0	0
K06223	dam; DNA adenine methylase [EC:2.1.1.72]	1	0	0	0	0	0	0	0	0	0
K06281	hyaB, hybC; hydrogenase large subunit [EC:1.12.99.6]	2	0	2	0	0	0	0	0	1	1
K06282	hyaA, hybO; hydrogenase small subunit [EC:1.12.99.6]	1	0	2	0	0	0	1	0	1	1
K06283	spoIIID; putative DeoR family transcriptional regulator, stage III sporulation protein D	1	1	1	1	1	1	0	1	1	1
K06284	abrB; AbrB family transcriptional regulator, transcriptional pleiotropic regulator of transition state genes	1	1	1	0	0	0	0	0	0	0
K06285	mtrB; transcription attenuation protein (tryptophan RNA-binding attenuator protein)	1	1	1	0	0	1	1	0	0	0
K06287	yhdE; nucleoside triphosphate pyrophosphatase [EC:3.6.1.-]	1	1	1	1	1	1	0	1	1	1
K06295	gerKA; spore germination protein KA	3	2	3	1	4	1	0	1	1	2
K06296	gerKB; spore germination protein KB	3	2	4	0	4	0	0	0	0	2
K06297	gerKC; spore germination protein KC	4	2	3	0	3	0	0	0	0	1
K06298	gerM; germination protein M	1	1	1	0	0	1	1	1	1	1
K06306	yaaH; spore germination protein	1	0	1	0	0	2	1	1	2	2
K06310	yndD; spore germination protein	0	0	1	0	0	0	0	0	0	0
K06311	yndE; spore germination protein	0	0	1	0	0	0	0	0	0	0
K06313	ypeB; spore germination protein	1	1	1	0	0	1	0	0	0	0
K06317	bofA; inhibitor of the pro-sigma K processing machinery	1	1	1	0	0	0	0	0	0	0
K06329	cotF; spore coat protein F	0	1	0	0	0	0	0	0	0	0
K06331	cotI; spore coat protein I	1	3	1	1	0	1	1	0	0	0
K06333	cotJB; spore coat protein JB	0	1	2	1	1	1	1	0	1	0
K06334	cotJC; spore coat protein JC	1	1	2	1	1	1	1	1	1	1
K06337	cotS; spore coat-associated protein S	2	0	1	0	0	0	0	0	0	0
K06346	jag; spoIIIJ-associated protein	1	1	1	1	1	1	1	1	1	1
K06373	spmA; spore maturation protein A	1	1	1	1	1	1	0	1	1	1
K06374	spmB; spore maturation protein B	1	1	1	1	1	1	1	1	1	1
K06378	spoIIAA; stage II sporulation protein AA (anti-sigma F factor antagonist)	1	1	1	1	1	1	1	1	1	1
K06379	spoIIAB; stage II sporulation protein AB (anti-sigma F factor) [EC:2.7.11.1]	1	1	1	1	1	1	1	1	1	1
K06381	spoIID; stage II sporulation protein D	3	3	3	1	2	2	2	2	1	1
K06382	spoIIE; stage II sporulation protein E [EC:3.1.3.16]	1	1	1	0	1	1	1	1	1	1
K06383	spoIIGA; stage II sporulation protein GA (sporulation sigma-E factor processing peptidase) [EC:3.4.23.-]	1	1	1	1	1	1	1	1	1	1
K06384	spoIIM; stage II sporulation protein M	1	1	1	0	0	1	0	1	0	0
K06385	spoIIP; stage II sporulation protein P	2	2	2	0	0	1	0	0	1	0
K06386	spoIIQ; stage II sporulation protein Q	0	0	1	0	0	0	0	0	0	0
K06387	spoIIR; stage II sporulation protein R	1	1	1	0	1	1	1	1	2	1
K06390	spoIIIAA; stage III sporulation protein AA	1	1	1	1	1	1	1	1	1	1
K06391	spoIIIAB; stage III sporulation protein AB	1	1	1	0	0	1	1	1	1	1
K06392	spoIIIAC; stage III sporulation protein AC	1	0	1	1	1	0	1	1	1	1
K06393	spoIIIAD; stage III sporulation protein AD	1	1	1	1	1	1	1	1	1	1
K06394	spoIIIAE; stage III sporulation protein AE	1	1	1	1	1	1	1	1	1	1
K06395	spoIIIAF; stage III sporulation protein AF	0	1	1	0	0	0	1	0	0	0
K06396	spoIIIAG; stage III sporulation protein AG	1	1	1	0	1	0	1	1	1	1
K06397	spoIIIAH; stage III sporulation protein AH	0	0	1	0	0	0	0	0	0	0
K06398	spoIVA; stage IV sporulation protein A	1	1	1	1	1	1	1	1	1	1
K06399	spoIVB; stage IV sporulation protein B [EC:3.4.21.116]	1	1	1	1	1	1	1	1	1	1
K06400	spoIVCA; site-specific DNA recombinase	3	0	0	0	1	0	0	1	0	2
K06402	spoIVFB; stage IV sporulation protein FB [EC:3.4.24.-]	1	1	1	0	0	0	0	0	0	0
K06405	spoVAC; stage V sporulation protein AC	2	2	2	1	1	1	1	1	1	1
K06406	spoVAD; stage V sporulation protein AD	2	3	2	1	1	1	1	1	1	1
K06407	spoVAE; stage V sporulation protein AE	3	3	3	1	1	1	2	1	1	1
K06408	spoVAF; stage V sporulation protein AF	1	1	1	0	0	0	0	0	0	0
K06409	spoVB; stage V sporulation protein B	2	1	1	1	1	1	1	1	1	1
K06410	spoVFA; dipicolinate synthase subunit A	1	1	1	1	1	1	1	1	1	2
K06411	spoVFB; dipicolinate synthase subunit B	1	1	1	1	1	1	0	1	1	2
K06412	spoVG; stage V sporulation protein G	2	1	1	1	1	0	0	1	1	1
K06413	spoVK; stage V sporulation protein K	1	1	1	0	0	1	1	1	1	1
K06415	spoVR; stage V sporulation protein R	1	1	1	0	0	0	0	0	0	0
K06416	spoVS; stage V sporulation protein S	2	2	2	0	0	0	0	1	0	0
K06418	SASP-A, sspA; small acid-soluble spore protein A (major alpha-type SASP)	0	0	0	1	0	0	0	0	0	0
K06419	SASP-B, sspB; small acid-soluble spore protein B (major beta-type SASP)	1	1	2	0	0	0	0	0	0	0
K06423	sspF; small acid-soluble spore protein F (minor alpha/beta-type SASP)	1	1	1	0	1	1	0	1	1	0
K06434	tlp; small acid-soluble spore protein (thioredoxin-like protein)	1	0	0	0	0	1	0	1	1	1

K06436	yabG; spore coat assemly protein	1	1	1	1	1	0	0	0	0	0
K06438	yqfD; similar to stage IV sporulation protein	1	1	1	0	1	1	1	1	1	1
K06442	tlyA; 23S rRNA (cytidine1920-2'-O)/16S rRNA (cytidine1409-2'-O)-methyltransferase [EC:2.1.1.226 2.1.1.227]	1	1	1	1	1	1	1	1	1	1
K06446	DCAA; acyl-CoA dehydrogenase [EC:1.3.99.-]	1	0	0	0	0	0	0	0	0	0
K06518	cidA; holin-like protein	0	0	0	0	0	0	1	0	0	0
K06580	SLC42A, RHAG, RHBG, RHCG, CD241; ammonium transporter Rh	0	0	0	0	0	0	0	0	1	0
K06599	chpD; AraC family transcriptional regulator, chemosensory pili system protein ChpD	1	0	0	0	0	0	0	0	0	0
K06714	rocR; arginine utilization regulatory protein	7	3	23	5	16	0	0	0	0	0
K06857	tupC, vupC; tungstate transport system ATP-binding protein [EC:7.3.2.6]	1	1	0	0	0	0	1	0	1	1
K06864	larE; pyridinium-3,5-biscarboxylic acid mononucleotide sulfurtransferase [EC:4.4.1.37]	1	1	1	1	2	0	1	0	1	1
K06871	K06871; uncharacterized protein	1	1	5	1	1	1	0	1	1	1
K06872	K06872; uncharacterized protein	0	1	1	0	0	0	0	1	0	0
K06881	nrnA; bifunctional oligoribonuclease and PAP phosphatase NrnA [EC:3.1.3.7 3.1.13.3]	1	1	1	1	1	1	1	1	1	1
K06888	K06888; uncharacterized protein	1	1	4	1	1	1	0	0	1	1
K06889	K06889; uncharacterized protein	1	0	2	1	1	0	0	1	2	1
K06894	yfhM; alpha-2-macroglobulin	1	1	1	0	0	1	0	1	1	1
K06897	K06897; 7,8-dihydropterin-6-yl-methyl-4-(beta-D-ribofuranosyl)aminobenzene 5'-phosphate synthase [EC:2.5.1.105]	1	0	1	0	0	1	0	1	1	1
K06898	larB; pyridinium-3,5-biscarboxylic acid mononucleotide synthase [EC:2.5.1.143]	1	1	1	1	2	0	0	0	1	1
K06900	capV; cGAMP-activated phospholipase [EC:3.1.1.32 3.1.1.-]	0	0	0	0	0	0	1	0	0	0
K06902	UMF1; MFS transporter, UMF1 family	0	0	1	0	0	0	0	0	0	0
K06904	K06904; uncharacterized protein	1	0	0	0	0	0	0	0	1	1
K06909	xtmB; phage terminase large subunit	0	0	0	1	1	1	0	1	0	0
K06910	PEBP, TFS1; phosphatidylethanolamine-binding protein	0	0	0	0	1	0	0	1	0	0
K06911	PIR; quercetin 2,3-dioxygenase [EC:1.13.11.24]	1	0	0	0	0	0	0	0	0	0
K06914	mfnD; tyramine---L-glutamate ligase [EC:6.3.4.24]	0	0	1	0	0	0	0	0	0	0
K06915	herA; DNA double-strand break repair helicase HerA and related ATPase	0	0	0	1	0	0	0	0	0	0
K06917	selU, mnmH; tRNA 2-selenouridine synthase [EC:2.9.1.3]	0	1	1	0	0	1	1	1	0	0
K06919	K06919; putative DNA primase/helicase	1	0	0	0	0	0	0	0	0	1
K06920	queC; 7-cyano-7-deazaguanine synthase [EC:6.3.4.20]	1	1	1	0	0	0	0	0	0	0
K06923	K06923; uncharacterized protein	1	1	1	0	1	1	1	1	1	1
K06924	K06924; uncharacterized protein	0	1	1	0	0	0	0	0	0	0
K06925	tsaE; tRNA threonylcarbamoyladenosine biosynthesis protein TsaE	1	1	1	1	1	1	1	1	1	1
K06926	K06926; uncharacterized protein	1	0	0	0	0	0	0	0	0	0
K06927	DPH6; diphthine-ammonia ligase [EC:6.3.1.14]	0	0	0	0	0	0	0	0	1	0
K06928	NTPCR; nucleoside-triphosphatase [EC:3.6.1.15]	0	0	0	0	0	1	1	0	1	1
K06929	K06929; uncharacterized protein	1	0	1	0	0	0	0	0	0	0
K06933	K06933; uncharacterized protein	2	1	1	0	1	0	0	1	0	0
K06934	K06934; uncharacterized protein	1	1	1	1	0	0	0	0	0	0
K06937	K06937; glycerol dibiphytanyl glycerol tetraether/macrocyclic archaeol synthase	0	1	1	0	0	0	0	0	1	1
K06941	rlmN; 23S rRNA (adenine2503-C2)-methyltransferase [EC:2.1.1.192]	1	0	1	1	1	1	1	1	0	0
K06942	ychF; ribosome-binding ATPase	1	1	1	1	1	1	1	1	1	1
K06949	rsgA, engC; ribosome biogenesis GTPase / thiamine phosphate phosphatase [EC:3.6.1.- 3.1.3.100]	1	1	1	1	2	1	1	1	1	1
K06950	K06950; uncharacterized protein	3	2	4	2	2	0	1	1	1	1
K06951	K06951; uncharacterized protein	0	1	0	0	0	0	0	0	0	0
K06952	yfdR; 5'-nucleotidase [EC:3.1.3.89]	0	0	0	0	1	0	0	0	0	0
K06958	rapZ; RNase adapter protein RapZ	1	1	1	1	1	1	1	1	1	1
K06959	tex; protein Tex	1	0	1	0	0	0	1	0	0	0
K06960	K06960; uncharacterized protein	1	1	1	1	1	1	1	1	1	1
K06962	K06962; uncharacterized protein	1	1	1	0	0	0	0	0	0	0
K06967	trmK; tRNA (adenine22-N1)-methyltransferase [EC:2.1.1.217]	1	1	1	0	1	1	1	1	1	1
K06971	K06971; uncharacterized protein	0	0	1	0	0	0	0	0	0	0
K06973	K06973; uncharacterized protein	1	1	1	1	1	1	0	1	1	1
K06975	K06975; uncharacterized protein	0	0	0	0	1	0	0	0	0	0
K06984	K06984; beta-ribofuranosylaminobenzene 5'-phosphate synthase [EC:2.4.2.54]	0	0	1	0	0	0	0	0	0	0
K06990	MEMO1; MEMO1 family protein	2	2	2	1	1	1	0	1	2	2
K06997	yggS, PROSC; PLP dependent protein	1	1	1	1	1	1	1	1	1	1
K06998	phzF; trans-2,3-dihydro-3-hydroxyanthranilate isomerase [EC:5.3.3.17]	0	0	1	0	0	0	0	0	0	0
K07001	K07001; NTE family protein	1	0	2	0	1	1	1	1	1	1
K07003	K07003; uncharacterized protein	0	0	0	0	0	1	0	0	0	0
K07005	K07005; uncharacterized protein	2	1	2	1	1	0	0	2	2	1
K07006	K07006; uncharacterized protein	3	0	2	1	1	0	0	0	0	0
K07007	baiN; 3-dehydro-bile acid Delta4,6-reductase [EC:1.3.1.114]	2	2	1	2	2	1	1	1	1	1
K07010	K07010; putative glutamine amidotransferase	1	1	1	1	0	0	0	0	1	1
K07012	cas3; CRISPR-associated endonuclease/helicase Cas3 [EC:3.1.-.- 5.6.2.4]	2	1	1	0	0	0	1	0	0	0
K07013	K07013; uncharacterized protein	0	1	1	0	0	0	0	0	0	0
K07015	yqeG; putative phosphatase [EC:3.1.3.-]	1	1	1	1	1	1	1	1	1	1
K07016	csm1, cas10; CRISPR-associated protein Csm1	0	1	0	0	0	0	0	0	0	0
K07017	K07017; uncharacterized protein	0	0	0	1	0	0	0	0	0	0
K07023	YGK1, HDDC2; 5'-deoxynucleotidase [EC:3.1.3.89]	0	0	2	0	0	0	0	0	0	0
K07024	SPP; sucrose-6-phosphatase [EC:3.1.3.24]	0	0	1	0	0	0	0	0	0	0
K07025	K07025; putative hydrolase of the HAD superfamily	1	1	4	0	0	1	1	1	0	0
K07027	K07027; glycosyltransferase 2 family protein	0	0	2	0	0	0	0	0	0	0
K07029	dagK; diacylglycerol kinase (ATP) [EC:2.7.1.107]	0	0	2	2	2	1	0	1	1	1
K07030	fakA; fatty acid kinase [EC:2.7.2.18]	1	1	1	1	1	1	1	1	1	1
K07033	K07033; uncharacterized protein	1	1	1	1	1	1	0	1	0	0
K07034	K07034; uncharacterized protein	1	0	0	0	1	1	0	1	0	0
K07035	K07035; uncharacterized protein	0	0	0	1	1	0	0	0	0	0
K07037	pgpH; cyclic-di-AMP phosphodiesterase PgpH [EC:3.1.4.-]	1	1	1	1	1	1	1	1	1	1
K07040	yceD, ylbN; DUF177 domain-containing protein	1	1	1	1	1	1	1	1	0	0
K07042	ybeY, yqfG; probable rRNA maturation factor	1	1	1	1	1	1	1	1	1	1
K07043	upp; UTP pyrophosphatase [EC:3.6.1.-]	1	1	1	0	1	0	0	1	0	0
K07045	K07045; uncharacterized protein	1	1	3	2	2	0	0	1	4	1
K07048	PTER, php; phosphotriesterase-related protein	0	0	0	0	0	0	0	1	0	0
K07052	K07052; CAAX protease family protein	3	1	2	0	1	1	0	1	3	2
K07053	E3.1.3.97; 3',5'-nucleoside bisphosphate phosphatase [EC:3.1.3.97]	2	3	4	1	1	1	1	1	1	1
K07056	rsmI; 16S rRNA (cytidine1402-2'-O)-methyltransferase [EC:2.1.1.198]	1	1	1	1	1	1	1	1	1	1
K07061	cmr1; CRISPR-associated protein Cmr1	1	0	0	0	0	0	0	0	0	0
K07067	disA; diadenylate cyclase [EC:2.7.7.85]	0	0	1	1	1	0	0	0	0	0
K07068	K07068; uncharacterized protein	0	0	1	0	0	0	0	0	0	0
K07069	K07069; uncharacterized protein	0	1	1	0	0	0	0	0	0	0
K07072	mfnF; (4-(4-[2-(gamma-L-glutamylamino)ethyl]phenoxymethyl)furan-2-yl)methanamine synthase [EC:2.5.1.131]	0	0	1	0	0	0	0	0	0	0
K07074	K07074; uncharacterized protein	0	0	1	0	0	0	0	0	0	0
K07075	K07075; uncharacterized protein	2	0	0	1	0	0	0	0	0	0
K07076	K07076; uncharacterized protein	2	2	0	0	0	0	0	0	1	1
K07079	K07079; uncharacterized protein	1	1	2	4	4	0	0	0	2	2
K07080	K07080; uncharacterized protein	0	0	4	0	0	2	0	0	0	0
K07082	K07082; UPF0755 protein	1	1	1	2	2	1	1	1	1	1
K07089	K07089; uncharacterized protein	1	1	3	0	0	2	1	1	1	1
K07090	K07090; uncharacterized protein	2	0	3	0	1	0	0	0	0	1
K07094	pcrB; heptaprenylglyceryl phosphate synthase [EC:2.5.1.-]	1	1	0	0	0	0	0	0	0	0
K07095	K07095; uncharacterized protein	1	3	3	1	2	0	1	0	0	0
K07096	K07096; uncharacterized protein	0	0	1	0	0	0	0	0	0	0
K07098	K07098; uncharacterized protein	0	1	3	0	1	0	0	0	2	1
K07099	K07099; uncharacterized protein	1	1	1	1	1	1	1	1	1	1
K07100	K07100; putative phosphoribosyl transferase	0	0	0	0	0	0	0	0	0	1
K07105	K07105; uncharacterized protein	0	1	0	0	1	0	0	0	0	0
K07107	ybgC; acyl-CoA thioester hydrolase [EC:3.1.2.-]	1	0	0	0	1	0	0	0	0	0
K07112	K07112; uncharacterized protein	3	3	0	0	0	1	1	3	0	0
K07113	fxsA; UPF0716 protein FxsA	0	0	1	0	0	0	0	0	0	0
K07120	K07120; uncharacterized protein	0	0	7	0	0	0	0	0	0	0
K07124	K07124; uncharacterized protein	0	0	0	0	0	1	0	1	0	0
K07130	kynB; arylformamidase [EC:3.5.1.9]	0	0	1	0	0	0	0	0	1	1
K07133	K07133; uncharacterized protein	2	0	1	1	0	0	0	0	0	0
K07137	K07137; uncharacterized protein	2	2	2	2	2	0	0	2	1	1
K07138	K07138; uncharacterized protein	1	1	1	1	0	0	0	0	0	0
K07141	mocA; molybdenum cofactor cytidylyltransferase [EC:2.7.7.76]	1	1	1	0	0	1	1	2	1	1
K07144	mfnE; 5-(aminomethyl)-3-furanmethanol phosphate kinase [EC:2.7.4.31]	0	0	1	0	0	0	0	0	0	0
K07149	K07149; uncharacterized protein	0	0	0	0	0	0	1	0	1	0
K07150	K07150; uncharacterized protein	1	1	1	1	1	0	0	0	0	0
K07161	K07161; uncharacterized protein	2	2	2	2	2	0	0	0	0	0
K07162	K07162; uncharacterized protein	1	0	0	0	0	0	0	0	0	0
K07166	K07166; ACT domain-containing protein	1	1	1	1	1	1	1	1	0	0
K07171	mazF, ndoA, chpA; mRNA interferase MazF [EC:3.1.-.-]	2	1	1	0	1	1	1	1	1	1
K07173	luxS; S-ribosylhomocysteine lyase [EC:4.4.1.21]	0	0	0	0	0	1	1	1	1	1
K07175	phoH2; PhoH-like ATPase	1	0	1	0	0	0	0	1	0	0
K07180	prkA; serine protein kinase	1	1	1	0	0	0	0	0	0	0

K07181	cdgJ; c-di-GMP phosphodiesterase [EC:3.1.4.52]	0	0	2	0	0	0	0	0	0	0
K07182	K07182; CBS domain-containing protein	0	0	1	0	0	0	0	1	0	0
K07213	ATOX1, ATX1, copZ, golB; copper chaperone	2	1	2	0	0	1	0	0	0	0
K07216	hr; hemerythrin	0	2	1	0	0	0	0	0	0	0
K07217	K07217; Mn-containing catalase	0	1	1	1	0	0	0	1	1	1
K07219	K07219; putative molybdopterin biosynthesis protein	0	0	1	0	0	0	1	0	0	1
K07220	K07220; uncharacterized protein	1	1	1	0	1	0	0	1	2	2
K07228	K07228; TrkA domain protein	0	0	1	0	0	0	0	0	0	0
K07230	p19, ftrA; periplasmic iron binding protein	0	0	1	0	0	0	0	0	0	0
K07238	TC.ZIP, zupT, ZRT3, ZIP2; zinc transporter, ZIP family	2	3	2	3	3	0	0	0	0	0
K07240	chrA; chromate transporter	2	2	2	2	2	2	2	2	2	2
K07243	FTR, FTH1, efeU; high-affinity iron transporter	0	0	1	0	0	0	0	0	0	0
K07250	gabT; 4-aminobutyrate aminotransferase / (S)-3-amino-2-methylpropionate transaminase / 5-aminovalerate transaminase [EC:2.6.1.19 2.6.1.22 2.6.1.48]	0	0	2	0	0	0	0	0	0	0
K07258	dacC, dacA, dacD; serine-type D-Ala-D-Ala carboxypeptidase (penicillin-binding protein 5/6) [EC:3.4.16.4]	4	3	6	2	2	2	2	3	3	3
K07263	pqqL; zinc protease [EC:3.4.24.-]	0	0	0	0	0	1	0	0	0	0
K07282	capA, pgsA; gamma-polyglutamate biosynthesis protein CapA	1	1	1	0	1	1	1	1	1	1
K07284	srtA; sortase A [EC:3.4.22.70]	1	0	0	1	3	0	0	2	0	1
K07300	chaA, CAX; Ca2+:H+ antiporter	0	0	0	0	1	0	0	0	0	0
K07301	yrbG; cation:H+ antiporter	0	1	0	1	1	0	0	1	0	0
K07306	dmsA; anaerobic dimethyl sulfoxide reductase subunit A [EC:1.8.5.3]	0	0	1	0	0	1	0	0	0	0
K07307	dmsB; anaerobic dimethyl sulfoxide reductase subunit B	1	0	1	1	0	1	0	0	0	0
K07310	ynfF; Tat-targeted selenate reductase subunit YnfF [EC:1.97.1.9]	0	0	0	1	0	0	0	0	0	0
K07313	pphA; serine/threonine protein phosphatase 1 [EC:3.1.3.16]	0	0	0	1	1	0	0	0	0	0
K07315	rsbU_P; phosphoserine phosphatase RsbU/P [EC:3.1.3.3]	0	0	2	0	0	0	0	0	0	0
K07316	mod; adenine-specific DNA-methyltransferase [EC:2.1.1.72]	3	0	0	0	0	0	1	0	0	0
K07317	K07317; adenine-specific DNA-methyltransferase [EC:2.1.1.72]	1	0	0	0	0	0	0	0	0	0
K07321	cooC; CO dehydrogenase maturation factor	3	2	2	3	3	0	0	0	0	0
K07322	ytfE, scdA; regulator of cell morphogenesis and NO signaling	1	1	0	0	0	0	0	0	0	0
K07335	bmpA, bmpB, tmpC; basic membrane protein A and related proteins	1	1	5	1	1	0	0	1	0	0
K07343	tfoX; DNA transformation protein and related proteins	0	0	0	0	1	0	0	1	0	0
K07391	comM; magnesium chelatase family protein	1	1	1	1	1	1	1	1	1	1
K07397	yhfA; putative redox protein	1	0	0	0	0	0	0	0	1	1
K07399	resB, ccs1; cytochrome c biogenesis protein	0	0	1	0	0	0	0	0	0	0
K07402	xdhC; xanthine dehydrogenase accessory factor	0	0	1	0	0	3	3	3	2	2
K07403	nfeD; membrane-bound serine protease (ClpP class)	1	1	1	0	0	0	1	1	0	1
K07406	melA; alpha-galactosidase [EC:3.2.1.22]	0	0	0	0	0	0	1	0	0	0
K07443	ybaZ; methylated-DNA-protein-cysteine methyltransferase related protein	0	0	1	0	0	0	0	0	0	0
K07444	ypsC; putative N6-adenine-specific DNA methylase [EC:2.1.1.-]	1	0	1	0	0	0	1	1	1	1
K07447	ruvX; putative pre-16S rRNA nuclease [EC:3.1.-.-]	1	1	1	1	1	1	1	1	1	1
K07451	mcrA; 5-methylcytosine-specific restriction enzyme A [EC:3.1.21.-]	0	0	0	0	0	0	0	0	0	1
K07452	mcrB; 5-methylcytosine-specific restriction enzyme B [EC:3.1.21.-]	1	0	0	0	0	0	0	0	0	0
K07456	mutS2; DNA mismatch repair protein MutS2	1	2	2	1	1	1	0	1	1	2
K07459	ybjD; putative ATP-dependent endonuclease of the OLD family	1	0	0	0	0	0	0	0	0	0
K07460	yraN; putative endonuclease	1	1	0	1	1	1	1	1	1	1
K07461	K07461; putative endonuclease	2	0	1	1	0	1	0	0	1	1
K07462	recJ; single-stranded-DNA-specific exonuclease [EC:3.1.-.-]	1	1	1	0	0	1	1	1	1	1
K07464	cas4; CRISPR-associated exonuclease Cas4 [EC:3.1.12.1]	2	1	1	0	1	0	1	0	0	0
K07469	mop; aldehyde oxidoreductase [EC:1.2.99.7]	0	1	6	0	0	0	1	0	2	2
K07473	dinJ; DNA-damage-inducible protein J	3	0	1	0	0	0	0	0	0	0
K07474	xtmA; phage terminase small subunit	1	0	0	0	0	1	0	0	0	0
K07475	cas3; CRISPR-associated endonuclease Cas3-HD [EC:3.1.-.-]	0	0	0	0	1	0	0	0	0	0
K07478	ycaJ; putative ATPase	1	1	1	1	1	1	0	1	1	1
K07481	K07481; transposase, IS5 family	0	0	1	0	0	0	0	0	0	0
K07482	K07482; transposase, IS30 family	0	1	1	0	0	0	0	0	0	0
K07483	K07483; transposase	6	0	8	0	1	0	0	2	0	0
K07484	K07484; transposase	14	0	3	0	0	0	0	0	0	0
K07486	K07486; transposase	1	0	2	0	0	0	0	0	0	0
K07487	K07487; transposase	1	1	0	0	0	0	0	0	0	0
K07491	rayT; REP-associated tyrosine transposase	0	0	3	0	0	0	0	0	0	0
K07493	K07493; putative transposase	4	0	7	0	0	0	0	0	0	0
K07494	K07494; putative transposase	1	0	0	0	0	0	0	0	0	0
K07495	K07495; putative transposase	0	1	0	0	0	0	0	0	0	0
K07497	K07497; putative transposase	7	0	10	0	4	0	0	2	0	0
K07504	K07504; predicted type IV restriction endonuclease	0	0	1	0	0	0	0	0	0	0
K07506	K07506; AraC family transcriptional regulator	0	1	0	0	0	0	0	0	0	0
K07507	mgtC; putative Mg2+ transporter-C (MgtC) family protein	1	1	1	1	1	1	1	1	1	1
K07533	prsA; foldase protein PrsA [EC:5.2.1.8]	0	0	1	0	0	0	0	0	0	1
K07540	E4.1.99.11; benzylsuccinate synthase [EC:4.1.99.11]	0	0	1	0	0	0	0	0	0	0
K07550	bbsB; benzoylsuccinyl-CoA thiolase BbsB subunit [EC:2.3.1.310]	0	0	1	0	0	0	0	0	0	0
K07552	bcr, tcaB; MFS transporter, DHA1 family, multidrug resistance protein	0	0	1	1	1	0	0	0	0	0
K07559	kptA; putative RNA 2'-phosphotransferase [EC:2.7.1.-]	0	0	1	0	0	0	0	0	0	0
K07560	dtd, DTD; D-aminoacyl-tRNA deacylase [EC:3.1.1.96]	1	1	1	1	1	1	1	1	1	1
K07566	tsaC, rimN, SUA5, YRDC; L-threonylcarbamoyladenylate synthase [EC:2.7.7.87]	1	1	1	1	1	1	0	1	1	1
K07568	queA; S-adenosylmethionine:tRNA ribosyltransferase-isomerase [EC:2.4.99.17]	1	1	1	1	1	1	1	1	1	1
K07571	K07571; S1 RNA binding domain protein	1	0	1	0	0	1	0	1	1	1
K07574	yhbY; RNA-binding protein	1	1	1	1	1	1	1	0	0	0
K07576	K07576; metallo-beta-lactamase family protein	1	1	1	1	1	1	1	1	1	1
K07588	MMAA, argK; GTPase [EC:3.6.5.-]	0	1	2	0	0	0	0	0	1	1
K07590	RP-L7A, rplGB; large subunit ribosomal protein L7A	1	1	1	1	1	1	1	1	0	0
K07636	phoR; two-component system, OmpR family, phosphate regulon sensor histidine kinase PhoR [EC:2.7.13.3]	1	0	1	0	1	1	1	1	1	1
K07642	baeS, smeS; two-component system, OmpR family, sensor histidine kinase BaeS [EC:2.7.13.3]	0	0	1	0	0	0	0	0	0	0
K07644	cusS, copS, silS; two-component system, OmpR family, heavy metal sensor histidine kinase CusS [EC:2.7.13.3]	0	0	0	0	1	0	0	0	0	0
K07646	kdpD; two-component system, OmpR family, sensor histidine kinase KdpD [EC:2.7.13.3]	2	0	1	0	0	1	0	0	0	0
K07651	resE; two-component system, OmpR family, sensor histidine kinase ResE [EC:2.7.13.3]	2	0	0	0	0	0	0	0	0	0
K07658	phoB1, phoP; two-component system, OmpR family, alkaline phosphatase synthesis response regulator PhoP	1	0	6	0	1	1	1	1	2	1
K07665	cusR, copR, silR; two-component system, OmpR family, copper resistance phosphate regulon response regulator CusR	0	0	1	0	0	0	0	0	0	0
K07666	qseB; two-component system, OmpR family, response regulator QseB	0	0	1	0	0	0	0	0	0	0
K07667	kdpE; two-component system, OmpR family, KDP operon response regulator KdpE	2	0	1	1	2	1	0	0	0	0
K07668	vicR; two-component system, OmpR family, response regulator VicR	0	0	2	1	1	0	0	0	0	0
K07669	mprA; two-component system, OmpR family, response regulator MprA	0	0	0	0	1	0	0	0	0	0
K07692	degU; two-component system, NarL family, response regulator DegU	1	0	0	0	0	0	0	0	1	1
K07694	vraR; two-component system, NarL family, vancomycin resistance associated response regulator VraR	0	0	0	0	0	0	0	0	1	1
K07696	nreC; two-component system, NarL family, response regulator NreC	0	0	1	0	0	0	0	0	0	0
K07699	spo0A; two-component system, response regulator, stage 0 sporulation protein A	1	1	1	1	1	1	0	2	1	1
K07705	lytT, lytR; two-component system, LytTR family, response regulator LytT	0	0	2	0	0	0	0	0	1	0
K07707	agrA, blpR, fsrA; two-component system, LytTR family, response regulator AgrA	0	0	0	0	0	0	0	0	1	1
K07708	glnL, ntrB; two-component system, NtrC family, nitrogen regulation sensor histidine kinase GlnL [EC:2.7.13.3]	0	0	1	0	0	0	0	0	0	0
K07710	atoS; two-component system, NtrC family, sensor histidine kinase AtoS [EC:2.7.13.3]	1	2	1	0	0	0	0	0	0	0
K07713	zraR, hydG; two-component system, NtrC family, response regulator HydG	1	0	0	0	0	0	0	0	0	0
K07717	glnK; two-component system, sensor histidine kinase GlnK [EC:2.7.13.3]	0	0	1	0	0	0	0	0	0	0
K07718	yesM; two-component system, sensor histidine kinase YesM [EC:2.7.13.3]	0	0	1	0	0	0	0	0	0	0
K07719	glnL; two-component system, response regulator GlnL	0	0	1	0	0	0	0	0	0	0
K07720	yesN; two-component system, response regulator YesN	4	2	10	2	2	0	0	0	0	0
K07723	ndoAI; CopG family transcriptional regulator / antitoxin EndoAI	1	1	1	0	0	0	0	0	0	0
K07727	K07727; putative transcriptional regulator	2	0	3	0	1	0	0	0	1	1
K07729	K07729; putative transcriptional regulator	0	0	3	2	4	0	0	3	4	2
K07736	carD; CarD family transcriptional regulator, regulator of rRNA transcription	1	1	2	1	1	1	1	1	1	2
K07738	nrdR; transcriptional repressor NrdR	1	1	1	0	0	1	1	1	2	1
K07741	antB; anti-repressor protein	0	0	0	0	0	0	0	0	1	1
K07742	ylxR; uncharacterized protein	1	1	1	1	1	1	1	1	1	1
K07749	frc; formyl-CoA transferase [EC:2.8.3.16]	0	1	2	0	0	0	0	0	0	0
K07774	tctD; two-component system, OmpR family, response regulator TctD	0	0	1	0	0	0	0	0	0	0
K07775	resD; two-component system, OmpR family, response regulator ResD	6	1	5	1	2	0	1	1	1	1
K07776	regX3; two-component system, OmpR family, response regulator RegX3	0	0	1	0	0	0	0	0	0	0
K07783	uhpC; MFS transporter, OPA family, sugar phosphate sensor protein UhpC	0	0	0	0	1	0	0	0	0	0
K07793	tctA; putative tricarboxylic transport membrane protein	1	0	20	0	0	0	0	0	0	0
K07794	tctB; putative tricarboxylic transport membrane protein	1	0	2	0	0	0	0	0	0	0
K07795	tctC; putative tricarboxylic transport membrane protein	0	0	8	0	0	0	0	0	0	0
K07800	agrD; AgrD protein	1	0	0	0	0	0	0	0	0	0
K07812	torZ; trimethylamine-N-oxide reductase (cytochrome c) [EC:1.7.2.3]	0	0	1	0	0	0	0	0	0	0
K07813	agrB; accessory gene regulator B	4	1	2	0	0	0	0	0	1	1
K07814	K07814; cyclic di-GMP phosphodiesterase [EC:3.1.4.-]	0	0	0	0	0	0	1	0	0	0
K07862	sstT; serine/threonine transporter	0	0	0	1	0	0	0	0	0	0
K07979	ytrA; GntR family transcriptional regulator	0	0	7	0	2	1	3	4	2	2
K08084	fimT; type IV fimbrial biogenesis protein FimT	0	0	1	0	0	0	0	0	0	0
K08097	comA; phosphosulfolactate synthase [EC:4.4.1.19]	0	0	5	0	0	0	0	0	0	0

K08151	tetA; MFS transporter, DHA1 family, tetracycline resistance protein	0	0	1	0	0	0	0	0	0	0
K08161	mdtG; MFS transporter, DHA1 family, multidrug resistance protein	0	0	5	0	0	0	0	0	0	0
K08169	yebQ; MFS transporter, DHA2 family, multidrug resistance protein	1	1	2	0	1	0	0	0	1	0
K08177	oxlT; MFS transporter, OFA family, oxalate/formate antiporter	1	0	0	0	0	1	0	0	0	0
K08191	exuT; MFS transporter, ACS family, hexuronate transporter	1	0	0	0	0	0	0	0	0	0
K08217	mef; MFS transporter, DHA3 family, macrolide efflux protein	1	1	2	0	1	0	1	0	0	0
K08223	fsr; MFS transporter, FSR family, fosmidomycin resistance protein	1	1	2	1	0	0	0	1	0	0
K08255	cdr; CoA-disulfide reductase [EC:1.8.1.14]	0	0	0	0	0	0	0	1	0	0
K08260	cbiZ; adenosylcobinamide hydrolase [EC:3.5.1.90]	0	0	2	0	0	0	0	0	0	0
K08264	hdrD; heterodisulfide reductase subunit D [EC:1.8.98.1]	0	0	2	1	0	0	0	0	0	0
K08279	caiE; carnitine operon protein CaiE	0	0	1	0	0	0	0	0	0	0
K08281	pncA; nicotinamidase/pyrazinamidase [EC:3.5.1.19 3.5.1.-]	0	1	0	1	1	0	0	0	0	0
K08282	E2.7.11.1; non-specific serine/threonine protein kinase [EC:2.7.11.1]	1	0	0	0	0	0	0	0	0	0
K08296	sixA; phosphohistidine phosphatase [EC:3.1.3.-]	1	0	0	0	0	0	0	0	0	0
K08297	caiA; crotonobetainyl-CoA dehydrogenase [EC:1.3.8.13]	1	0	0	0	0	0	0	0	0	0
K08301	rng, cafA; ribonuclease G [EC:3.1.26.-]	1	1	1	1	1	1	0	1	1	1
K08303	prtC, trhP; U32 family peptidase [EC:3.4.-.-]	2	2	2	2	3	3	1	2	1	1
K08309	slt; soluble lytic murein transglycosylase [EC:4.2.2.-]	1	1	1	0	0	1	1	1	0	0
K08315	hycI; hydrogenase 3 maturation protease [EC:3.4.23.51]	0	0	0	0	0	0	1	0	1	1
K08316	rsmD; 16S rRNA (guanine966-N2)-methyltransferase [EC:2.1.1.171]	1	1	1	1	1	1	0	1	1	1
K08352	phsA, psrA; thiosulfate reductase / polysulfide reductase chain A [EC:1.8.5.5]	1	0	2	0	0	0	0	0	0	0
K08368	yaaU; MFS transporter, putative metabolite transport protein	0	0	0	0	4	0	0	0	0	0
K08369	ydjE; MFS transporter, putative metabolite:H+ symporter	2	1	2	0	0	0	0	1	0	0
K08384	spoVD; stage V sporulation protein D (sporulation-specific penicillin-binding protein)	0	1	1	1	1	1	1	1	1	1
K08590	nit; 2-oxoglutaramate amidase [EC:3.5.1.111]	0	0	0	0	1	0	0	0	0	0
K08591	plsY; acyl phosphate:glycerol-3-phosphate acyltransferase [EC:2.3.1.275]	2	1	1	1	1	2	1	1	1	1
K08600	srtB; sortase B [EC:3.4.22.71]	0	0	0	1	3	0	0	0	0	0
K08602	pepF, pepB; oligoendopeptidase F [EC:3.4.24.-]	1	1	2	1	1	1	1	1	1	1
K08641	vanX; zinc D-Ala-D-Ala dipeptidase [EC:3.4.13.22]	0	1	2	0	1	0	0	0	1	1
K08659	pepDA, pepDB; dipeptidase [EC:3.4.-.-]	1	0	0	0	0	0	0	0	0	0
K08678	UXS1, uxs; UDP-glucuronate decarboxylase [EC:4.1.1.35]	0	1	0	0	0	0	0	0	0	0
K08679	GAE, cap1J; UDP-glucuronate 4-epimerase [EC:5.1.3.6]	1	0	0	0	0	0	0	0	0	0
K08681	pdxT, pdx2; pyridoxal 5'-phosphate synthase pdxT subunit [EC:4.3.3.6]	1	1	2	0	1	1	1	1	0	0
K08687	E3.5.1.59; N-carbamoylsarcosine amidase [EC:3.5.1.59]	1	0	0	0	0	0	0	0	0	0
K08722	yfbR; 5'-deoxynucleotidase [EC:3.1.3.89]	1	1	1	1	1	1	1	1	1	1
K08764	SCP2, SCPX; sterol carrier protein 2 [EC:2.3.1.176]	0	0	1	0	0	0	0	0	0	0
K08963	mtnA; methylthioribose-1-phosphate isomerase [EC:5.3.1.23]	1	1	1	1	1	1	0	1	1	1
K08965	mtnW; 2,3-diketo-5-methylthiopentyl-1-phosphate enolase [EC:5.3.2.5]	1	0	1	0	0	0	0	0	0	0
K08966	mtnX; 2-hydroxy-3-keto-5-methylthiopentenyl-1-phosphate phosphatase [EC:3.1.3.87]	0	0	1	0	0	0	0	0	0	0
K08968	msrC; L-methionine (R)-S-oxide reductase [EC:1.8.4.14]	0	0	0	1	0	0	0	0	0	0
K08972	K08972; putative membrane protein	2	2	3	0	0	1	0	0	0	0
K08974	K08974; putative membrane protein	0	0	0	0	0	1	0	0	0	0
K08978	TC.BAT1; bacterial/archaeal transporter family protein	0	0	2	0	0	0	0	0	0	0
K08984	yjdF; putative membrane protein	0	0	1	0	0	0	0	0	0	0
K08986	ycgQ; putative membrane protein	0	0	2	0	0	0	0	0	0	0
K08997	SELENOO, selO; protein adenylyltransferase [EC:2.7.7.108]	1	0	0	0	0	0	0	0	0	0
K08998	K08998; uncharacterized protein	0	0	1	1	0	1	1	0	1	1
K09000	cmr4; CRISPR-associated protein Cmr4	1	0	0	0	0	0	0	0	0	0
K09002	csm3; CRISPR-associated protein Csm3	0	1	0	0	0	0	0	0	0	0
K09005	K09005; uncharacterized protein	0	1	1	0	0	0	0	1	1	1
K09013	sufC; Fe-S cluster assembly ATP-binding protein	1	1	1	1	1	1	0	1	0	0
K09017	rutR; TetR/AcrR family transcriptional regulator	0	0	4	0	0	0	0	0	0	0
K09020	rutB; ureidoacrylate peracid hydrolase [EC:3.5.1.110]	0	0	1	0	0	0	0	0	0	0
K09022	ridA, tdcF, RIDA; 2-iminobutanoate/2-iminopropanoate deaminase [EC:3.5.99.10]	0	1	3	0	1	1	1	3	0	0
K09065	argF; N-acetylornithine carbamoyltransferase [EC:2.1.3.9]	0	0	2	0	0	0	0	0	0	0
K09116	K09116; damage-control phosphatase, subfamily I [EC:3.1.3.-]	0	0	0	0	0	1	0	0	0	0
K09117	K09117; uncharacterized protein	1	0	1	1	1	0	0	0	0	1
K09118	K09118; uncharacterized protein	1	1	1	0	0	0	0	0	0	0
K09121	larC; pyridinium-3,5-bisthiocarboxylic acid mononucleotide nickel chelatase [EC:4.99.1.12]	1	1	1	1	2	0	0	0	1	1
K09123	lhpI; cis-L-3-hydroxyproline dehydratase [EC:4.2.1.171]	0	2	4	0	0	1	0	0	0	0
K09124	K09124; uncharacterized protein	0	0	1	0	0	0	0	0	0	0
K09125	yhhQ; queuosine precursor transporter	0	0	0	1	1	1	1	1	1	1
K09126	K09126; uncharacterized protein	1	1	1	0	1	0	0	0	0	0
K09128	acnX2; mevalonate 5-phosphate dehydratase small subunit [EC:4.2.1.-]	0	2	4	0	0	1	0	0	0	0
K09131	K09131; uncharacterized protein	1	1	1	0	0	0	0	0	0	0
K09133	K09133; uncharacterized protein	0	0	3	0	0	1	1	0	1	1
K09137	K09137; uncharacterized protein	0	0	1	0	0	0	0	0	0	1
K09138	K09138; uncharacterized protein	0	0	1	0	0	1	1	1	2	2
K09153	K09153; small membrane protein	0	1	1	1	0	1	1	1	1	1
K09154	K09154; uncharacterized protein	0	0	1	0	0	0	0	0	0	0
K09157	K09157; uncharacterized protein	1	0	1	1	1	1	1	1	0	0
K09165	K09165; dodecin	1	0	1	0	0	0	0	0	0	0
K09251	patA; putrescine aminotransferase [EC:2.6.1.82]	1	1	1	0	0	0	0	0	0	0
K09384	K09384; uncharacterized protein	0	0	1	0	0	0	0	0	0	0
K09457	queF; 7-cyano-7-deazaguanine reductase [EC:1.7.1.13]	1	0	0	0	0	1	0	0	0	0
K09458	fabF, OXSM, CEM1; 3-oxoacyl-[acyl-carrier-protein] synthase II [EC:2.3.1.179]	2	1	1	0	2	1	1	1	1	1
K09493	CCT1, TCP1; T-complex protein 1 subunit alpha	1	0	1	0	0	0	0	0	0	0
K09497	CCT5; T-complex protein 1 subunit epsilon	0	1	0	0	0	0	0	0	0	0
K09681	gltC; LysR family transcriptional regulator, transcription activator of glutamate synthase operon	1	0	1	2	0	0	0	0	0	0
K09683	hutP; hut operon positive regulatory protein	0	1	1	0	0	0	0	0	0	0
K09684	pucR; PucR family transcriptional regulator, purine catabolism regulatory protein	1	1	2	0	0	0	0	1	0	0
K09685	purR; purine operon repressor	1	1	1	0	0	0	0	0	0	0
K09690	wzm, rfbA; lipopolysaccharide transport system permease protein	0	0	1	0	0	0	0	0	0	0
K09691	wzt, rbfB; lipopolysaccharide transport system ATP-binding protein	0	0	1	0	0	0	0	1	0	0
K09694	nodJ; lipooligosaccharide transport system permease protein	0	0	1	0	0	0	0	0	0	0
K09695	nodI; lipooligosaccharide transport system ATP-binding protein	0	0	1	0	0	0	0	0	0	0
K09698	gltX; nondiscriminating glutamyl-tRNA synthetase [EC:6.1.1.24]	0	1	1	1	1	1	1	1	0	0
K09705	K09705; uncharacterized protein	0	0	0	1	1	0	0	0	0	0
K09707	K09707; uncharacterized protein	0	0	0	1	0	0	0	0	0	0
K09710	ybeB; ribosome-associated protein	1	1	1	1	0	1	1	1	1	1
K09711	lipS1; lipoyl synthase	0	0	1	1	0	0	0	0	0	0
K09729	K09729; uncharacterized protein	1	1	1	0	0	0	0	1	0	0
K09733	mfnB; (5-formylfuran-3-yl)methyl phosphate synthase [EC:4.2.3.153]	0	0	1	0	0	0	0	0	0	0
K09740	K09740; uncharacterized protein	1	1	2	0	0	1	0	0	1	1
K09747	ebfC; nucleoid-associated protein EbfC	1	1	1	1	1	1	1	1	1	1
K09748	rimP; ribosome maturation factor RimP	1	1	1	1	1	1	1	1	1	1
K09749	K09749; uncharacterized protein	0	1	0	0	0	0	0	0	0	0
K09760	rmuC; DNA recombination protein RmuC	1	0	1	1	1	0	0	1	1	1
K09761	rsmE; 16S rRNA (uracil1498-N3)-methyltransferase [EC:2.1.1.193]	1	1	1	1	1	1	1	0	0	0
K09762	whiA; cell division protein WhiA	1	1	1	1	1	1	1	1	1	1
K09764	K09764; uncharacterized protein	1	1	1	0	0	0	0	1	0	0
K09765	queH; epoxyqueuosine reductase [EC:1.17.99.6]	1	1	1	1	1	1	1	1	1	1
K09767	yajQ; cyclic-di-GMP-binding protein	1	1	1	1	1	0	0	0	0	0
K09768	K09768; uncharacterized protein	1	1	1	0	0	0	1	1	1	1
K09769	ymdB; 2',3'-cyclic-nucleotide 2'-phosphodiesterase [EC:3.1.4.16]	1	1	1	1	1	1	1	1	1	1
K09770	K09770; uncharacterized protein	0	1	0	0	0	0	0	0	0	0
K09771	TC.SMR3; small multidrug resistance family-3 protein	1	0	1	0	0	0	0	0	0	0
K09772	sepF; cell division inhibitor SepF	1	1	1	1	0	1	1	1	1	1
K09773	ppsR; [pyruvate, water dikinase]-phosphate phosphotransferase / [pyruvate, water dikinase] kinase [EC:2.7.4.28 2.7.11.33]	1	0	1	1	1	0	0	0	0	0
K09776	K09776; uncharacterized protein	0	0	1	0	0	0	0	0	0	0
K09777	remA; extracellular matrix regulatory protein A	1	1	1	0	0	1	1	1	1	1
K09779	K09779; uncharacterized protein	0	1	1	1	1	0	1	1	1	1
K09786	K09786; uncharacterized protein	1	1	1	0	0	0	0	0	0	0
K09787	K09787; uncharacterized protein	1	1	1	1	1	1	1	1	1	1
K09790	K09790; uncharacterized protein	0	0	0	0	1	0	0	0	0	0
K09793	K09793; uncharacterized protein	1	0	0	0	0	0	0	1	1	1
K09798	K09798; uncharacterized protein	1	0	1	0	0	0	0	0	0	0
K09803	K09803; uncharacterized protein	0	0	0	1	0	0	0	0	0	0
K09807	K09807; uncharacterized protein	0	0	1	0	0	0	0	0	0	0
K09808	lolC_E; lipoprotein-releasing system permease protein	1	0	0	0	0	0	0	0	0	0
K09810	lolD; lipoprotein-releasing system ATP-binding protein [EC:7.6.2.-]	1	0	0	0	0	0	0	0	0	0
K09811	ftsX; cell division transport system permease protein	1	2	2	1	1	1	0	1	1	1
K09812	ftsE; cell division transport system ATP-binding protein	1	1	2	1	1	1	0	1	1	1
K09815	znuA; zinc transport system substrate-binding protein	1	1	2	0	1	0	0	1	1	1
K09816	znuB; zinc transport system permease protein	1	1	2	1	1	0	0	1	1	1
K09817	znuC; zinc transport system ATP-binding protein [EC:7.2.2.20]	1	1	2	1	1	0	0	1	1	1

K09820	ABC.MN.A; manganese/iron transport system ATP-binding protein	0	0	0	0	0	0	1	0	0	0
K09825	perR; Fur family transcriptional regulator, peroxide stress response regulator	1	0	3	0	1	1	1	1	1	1
K09861	K09861; uncharacterized protein	0	0	0	1	0	0	0	0	0	0
K09888	zapA; cell division protein ZapA	1	0	1	0	0	1	0	0	1	1
K09903	pyrH; uridylate kinase [EC:2.7.4.22]	1	1	1	1	1	1	0	1	1	1
K09913	ppnP; purine/pyrimidine-nucleoside phosphorylase [EC:2.4.2.1 2.4.2.2]	0	0	1	0	0	0	0	0	0	0
K09928	K09928; uncharacterized protein	0	0	1	0	0	0	0	0	0	0
K09936	TC.BAT2; bacterial/archaeal transporter family-2 protein	2	1	2	2	2	1	1	1	1	1
K09940	K09940; uncharacterized protein	1	1	1	0	0	0	0	0	0	0
K09942	K09942; uncharacterized protein	0	0	1	0	0	0	0	0	0	0
K09951	cas2; CRISPR-associated protein Cas2	3	2	1	1	1	0	1	0	0	0
K09952	csn1, cas9; CRISPR-associated endonuclease Csn1 [EC:3.1.-.-]	1	0	0	1	0	0	0	0	0	0
K09974	K09974; uncharacterized protein	0	0	0	1	1	0	0	0	1	1
K10026	queE; 7-carboxy-7-deazaguanine synthase [EC:4.3.99.3]	1	1	1	0	0	0	0	0	0	0
K10112	msmX, msmK, malK, sugC, ggtA, msiK; multiple sugar transport system ATP-binding protein [EC:7.5.2.-]	0	0	1	0	0	0	0	0	0	0
K10117	msmE; raffinose/stachyose/melibiose transport system substrate-binding protein	0	0	0	0	0	0	0	1	0	0
K10118	msmF; raffinose/stachyose/melibiose transport system permease protein	0	0	0	0	0	0	0	1	0	0
K10206	E2.6.1.83; LL-diaminopimelate aminotransferase [EC:2.6.1.83]	1	1	2	1	2	1	1	1	1	1
K10216	dmpD, xylF; 2-hydroxymuconate-semialdehyde hydrolase [EC:3.7.1.9]	0	0	0	1	0	0	0	0	0	0
K10218	ligK, galC; 4-hydroxy-4-methyl-2-oxoglutarate aldolase [EC:4.1.3.17]	0	0	1	0	0	0	0	0	0	0
K10439	rbsB; ribose transport system substrate-binding protein	0	0	1	0	0	0	0	0	0	0
K10440	rbsC; ribose transport system permease protein	0	1	1	0	0	0	1	0	0	0
K10441	rbsA; ribose transport system ATP-binding protein [EC:7.5.2.7]	0	0	1	0	0	0	0	0	0	0
K10549	alsB; D-allose transport system substrate-binding protein	0	1	1	0	0	0	1	0	0	0
K10554	frcA; fructose transport system ATP-binding protein	0	1	1	0	0	0	0	0	0	0
K10563	mutM, fpg; formamidopyrimidine-DNA glycosylase [EC:3.2.2.23 4.2.99.18]	1	1	2	1	1	1	1	1	1	1
K10670	grdA; glycine/sarcosine/betaine reductase complex component A [EC:1.21.4.2 1.21.4.3 1.21.4.4]	7	4	9	0	0	0	0	0	0	0
K10672	grdB; glycine reductase complex component B subunit gamma [EC:1.21.4.2]	6	4	4	0	0	0	0	0	0	0
K10679	nfnB, nfsB; nitroreductase / dihydropteridine reductase [EC:1.-.-.- 1.5.1.34]	1	0	0	0	0	0	0	0	0	0
K10680	nemA; N-ethylmaleimide reductase [EC:1.-.-.-]	1	0	0	0	0	0	0	0	0	0
K10681	saeS; two-component system, OmpR family, sensor histidine kinase SaeS [EC:2.7.13.3]	0	0	0	0	1	0	0	0	0	0
K10714	mtdB; methylene-tetrahydromethanopterin dehydrogenase [EC:1.5.1.-]	0	0	1	0	0	0	0	0	0	0
K10716	kch, trkA, mthK, pch; voltage-gated potassium channel	0	0	0	1	0	0	0	0	0	0
K10773	NTH; endonuclease III [EC:4.2.99.18]	1	1	1	1	1	1	0	1	1	1
K10797	enr; 2-enoate reductase [EC:1.3.1.31]	0	0	4	0	0	0	0	0	0	0
K10819	E2.7.13.3; histidine kinase [EC:2.7.13.3]	1	0	2	0	0	0	0	0	0	0
K10823	oppF; oligopeptide transport system ATP-binding protein	1	0	6	0	0	0	0	0	0	0
K10907	K10907; aminotransferase [EC:2.6.1.-]	1	1	2	1	1	1	1	2	2	2
K10913	hapR, luxR, litR; TetR/AcrR family transcriptional regulator, hemagglutinin/protease regulatory protein	0	0	0	0	0	0	0	0	1	1
K10914	crp; CRP/FNR family transcriptional regulator, cyclic AMP receptor protein	4	1	2	0	0	0	0	0	0	0
K10941	flrA, fleQ, flaK; sigma-54 dependent transcriptional regulator, flagellar regulatory protein	0	1	0	1	0	0	0	0	0	0
K10943	flrC, fleR, flaM; two-component system, response regulator FlrC	0	0	1	0	0	0	0	0	0	0
K10947	padR; PadR family transcriptional regulator, regulatory protein PadR	2	1	5	1	3	0	1	0	3	3
K10977	aksA; methanogen homocitrate synthase [EC:2.3.3.14 2.3.3.-]	0	0	1	0	0	1	1	0	0	0
K10979	ku; DNA end-binding protein Ku	1	1	1	0	0	0	0	0	1	1
K11041	eta; exfoliative toxin A/B	1	0	0	1	0	0	0	0	0	0
K11050	cylA; multidrug/hemolysin transport system ATP-binding protein	0	0	0	0	1	0	0	1	0	1
K11051	cylB; multidrug/hemolysin transport system permease protein	0	0	0	0	1	0	0	1	0	1
K11068	hlyIII; hemolysin III	0	0	1	1	2	0	1	0	0	0
K11069	potD; spermidine/putrescine transport system substrate-binding protein	0	1	1	0	0	0	0	0	0	0
K11070	potC; spermidine/putrescine transport system permease protein	0	1	1	0	0	0	0	0	0	0
K11071	potB; spermidine/putrescine transport system permease protein	0	1	1	0	0	0	0	0	0	0
K11072	potA; spermidine/putrescine transport system ATP-binding protein [EC:7.6.2.11]	0	1	1	0	0	0	0	0	0	0
K11105	cvrA, nhaP2; potassium/hydrogen antiporter	0	0	0	0	0	0	0	1	0	0
K11145	mrnC; mini-ribonuclease III [EC:3.1.26.-]	1	1	1	1	1	1	1	1	1	1
K11175	purN; phosphoribosylglycinamide formyltransferase 1 [EC:2.1.2.2]	1	1	1	1	1	1	1	1	1	1
K11178	yagS; xanthine dehydrogenase YagS FAD-binding subunit [EC:1.17.1.4]	0	0	1	0	0	0	0	0	0	0
K11179	tusE, dsrC; tRNA 2-thiouridine synthesizing protein E [EC:2.8.1.-]	1	1	2	0	0	0	0	0	0	0
K11180	dsrA; dissimilatory sulfite reductase alpha subunit [EC:1.8.99.5]	1	1	1	0	0	0	0	0	0	0
K11181	dsrB; dissimilatory sulfite reductase beta subunit [EC:1.8.99.5]	1	1	1	0	0	0	0	0	0	0
K11261	fwdE, fmdE; formylmethanofuran dehydrogenase subunit E [EC:1.2.7.12]	1	1	3	0	2	0	1	0	1	1
K11358	yhdR; aspartate aminotransferase [EC:2.6.1.1]	0	0	0	0	0	0	0	1	0	0
K11383	kinB; two-component system, NtrC family, sensor histidine kinase KinB [EC:2.7.13.3]	0	0	1	0	0	0	0	0	0	0
K11392	rsmF; 16S rRNA (cytosine1407-C5)-methyltransferase [EC:2.1.1.178]	0	0	1	0	0	0	0	0	0	0
K11444	wspR; two-component system, chemotaxis family, response regulator WspR [EC:2.7.7.65]	0	0	1	0	0	0	0	0	0	0
K11473	glcF; glycolate dehydrogenase iron-sulfur subunit [EC:1.1.99.14]	1	0	7	0	0	0	0	0	0	0
K11475	vanR; GntR family transcriptional regulator, vanillate catabolism transcriptional regulator	0	0	4	0	0	0	1	1	0	0
K11527	K11527; two-component system, sensor histidine kinase and response regulator [EC:2.7.13.3]	0	1	0	0	0	0	0	0	0	0
K11616	maeN; malate:Na+ symporter	0	0	0	0	0	0	1	0	0	0
K11688	dctP; C4-dicarboxylate-binding protein DctP	1	0	0	0	0	0	0	0	0	0
K11690	dctM; C4-dicarboxylate transporter, DctM subunit	1	0	1	0	0	1	0	2	1	0
K11693	femX, fmhB; peptidoglycan pentaglycine glycine transferase (the first glycine) [EC:2.3.2.16]	1	1	1	0	0	1	1	1	1	1
K11717	sufS; cysteine desulfurase / selenocysteine lyase [EC:2.8.1.7 4.4.1.16]	2	2	1	1	1	1	1	2	2	2
K11733	lysP; lysine-specific permease	0	0	1	0	0	0	0	0	0	0
K11737	cycA; D-serine/D-alanine/glycine transporter	1	0	0	0	0	0	0	0	0	0
K11741	sugE; quaternary ammonium compound-resistance protein SugE	0	0	1	1	0	0	0	0	1	0
K11749	rseP; regulator of sigma E protease [EC:3.4.24.-]	1	1	1	1	1	1	1	1	1	1
K11752	ribD; diaminohydroxyphosphoribosylaminopyrimidine deaminase / 5-amino-6-(5-phosphoribosylamino)uracil reductase [EC:3.5.4.26 1.1.1.193]	1	0	1	1	1	1	1	0	1	1
K11753	ribF; riboflavin kinase / FMN adenylyltransferase [EC:2.7.1.26 2.7.7.2]	1	1	2	0	1	1	1	1	1	0
K11754	folC; dihydrofolate synthase / folylpolyglutamate synthase [EC:6.3.2.12 6.3.2.17]	1	1	1	1	1	1	0	1	1	1
K11755	hisIE; phosphoribosyl-AMP cyclohydrolase / phosphoribosyl-ATP pyrophosphohydrolase [EC:3.5.4.19 3.6.1.31]	0	0	0	1	1	0	0	1	1	1
K11921	cynR; LysR family transcriptional regulator, cyn operon transcriptional activator	0	0	0	1	0	0	0	0	0	0
K11924	mntR; DtxR family transcriptional regulator, manganese transport regulator	0	0	1	1	1	0	0	0	1	0
K11927	rhlE; ATP-dependent RNA helicase RhlE [EC:3.6.4.13]	0	0	0	0	0	1	0	1	1	1
K11928	putP; sodium/proline symporter	0	0	1	1	1	1	0	0	0	0
K11936	pgaC, icaA; poly-beta-1,6-N-acetyl-D-glucosamine synthase [EC:2.4.1.-]	0	0	1	0	0	0	0	0	0	0
K11956	natD; neutral amino acid transport system permease protein	0	0	1	0	0	0	0	1	0	0
K11957	natA; neutral amino acid transport system ATP-binding protein	0	0	1	0	0	0	0	1	0	0
K11991	tadA; tRNA(adenine34) deaminase [EC:3.5.4.33]	1	1	1	1	1	1	0	1	1	1
K12132	prkC, stkP; eukaryotic-like serine/threonine-protein kinase [EC:2.7.11.1]	1	1	1	1	2	1	0	1	2	1
K12137	hyfB; hydrogenase-4 component B [EC:1.-.-.-]	2	0	0	0	0	0	0	0	0	0
K12138	hyfC; hydrogenase-4 component C [EC:1.-.-.-]	0	0	0	0	0	0	1	0	0	0
K12139	hyfD; hydrogenase-4 component D [EC:1.-.-.-]	0	0	0	0	0	0	0	0	0	1
K12140	hyfE; hydrogenase-4 component E [EC:1.-.-.-]	2	0	0	0	0	0	1	0	0	0
K12141	hyfF; hydrogenase-4 component F [EC:1.-.-.-]	2	0	0	0	0	0	1	0	0	0
K12251	aguB; N-carbamoylputrescine amidase [EC:3.5.1.53]	1	1	2	0	0	0	0	0	0	0
K12256	spuC; putrescine---pyruvate transaminase [EC:2.6.1.113]	0	1	0	0	0	0	0	0	0	0
K12264	norV; anaerobic nitric oxide reductase flavorubredoxin	2	1	2	1	1	0	0	1	1	1
K12267	msrAB; peptide methionine sulfoxide reductase msrA/msrB [EC:1.8.4.11 1.8.4.12]	1	1	1	0	1	0	0	1	1	1
K12339	cysM; S-sulfo-L-cysteine synthase (O-acetyl-L-serine-dependent) [EC:2.5.1.144]	1	0	0	0	0	0	0	0	0	0
K12410	npdA; NAD-dependent deacetylase [EC:2.3.1.286]	1	1	1	1	1	0	0	1	1	1
K12452	ascC, ddhC, rfbH; CDP-4-dehydro-6-deoxyglucose reductase, E1 [EC:1.17.1.1]	0	0	1	0	0	0	0	1	0	0
K12507	fadK; acyl-CoA synthetase [EC:6.2.1.-]	1	0	0	0	0	0	0	0	0	0
K12508	fcs; feruloyl-CoA synthase [EC:6.2.1.34]	0	0	0	0	0	0	0	0	1	0
K12510	tadB; tight adherence protein B	1	1	1	0	0	0	0	1	1	1
K12511	tadC; tight adherence protein C	1	1	1	0	0	0	0	1	1	1
K12527	ygfK; putative selenate reductase [EC:1.97.1.9]	0	0	1	0	0	0	0	0	0	0
K12528	xdhD; putative selenate reductase molybdopterin-binding subunit	1	0	0	0	0	0	0	0	0	0
K12573	rnr, vacB; ribonuclease R [EC:3.1.13.1]	1	1	1	1	1	0	0	1	0	1
K12574	rnj; ribonuclease J [EC:3.1.-.-]	1	1	1	1	1	1	1	1	1	1
K12583	mgtA; phosphatidylinositol alpha 1,6-mannosyltransferase [EC:2.4.1.-]	0	0	1	0	0	0	0	0	0	0
K12700	rihC; non-specific ribonucleoside hydrolase [EC:3.2.2.-]	1	0	0	0	0	0	0	0	0	0
K12910	pmmS; 2-phosphinomethylmalate synthase [EC:2.3.3.18]	1	1	0	0	0	1	0	1	0	0
K12940	abgA; aminobenzoyl-glutamate utilization protein A	0	1	1	0	0	0	0	0	0	0
K12950	ctpC; manganese-transporting P-type ATPase C [EC:7.2.2.22]	0	0	1	2	0	0	0	0	0	0
K12960	mtaD; 5-methylthioadenosine/S-adenosylhomocysteine deaminase [EC:3.5.4.31 3.5.4.28]	1	1	5	1	2	1	1	4	1	1
K12994	wbdB, mtfB; O-antigen biosynthesis alpha-1,3-mannosyltransferase [EC:2.4.1.349 2.4.1.-]	2	2	4	2	2	2	2	0	0	0
K12999	rgpI; glucosyltransferase [EC:2.4.1.-]	0	0	0	0	0	0	0	0	2	1
K13010	per, rfbE; perosamine synthetase [EC:2.6.1.102]	0	2	2	0	0	0	0	0	0	0
K13012	wbqP; O-antigen biosynthesis protein WbqP	0	0	0	0	0	1	0	0	0	0
K13015	wbpA; UDP-N-acetyl-D-glucosamine dehydrogenase [EC:1.1.1.136]	0	1	0	0	0	0	0	0	0	0
K13017	wbpE, wlbC; UDP-2-acetamido-2-deoxy-ribo-hexuluronate aminotransferase [EC:2.6.1.98]	0	1	0	0	0	0	0	0	0	0
K13020	wlbA, bplA; UDP-N-acetyl-2-amino-2-deoxyglucuronate dehydrogenase [EC:1.1.1.335]	0	1	0	0	0	0	0	0	0	0
K13038	coaBC, dfp; phosphopantothenoylcysteine decarboxylase / phosphopantothenate---cysteine ligase [EC:4.1.1.36 6.3.2.5]	1	1	1	1	1	1	0	1	1	1
K13039	comE; sulfopyruvate decarboxylase subunit beta [EC:4.1.1.79]	0	0	1	0	0	0	0	0	0	0
K13052	divIC, divA; cell division protein DivIC	0	0	1	0	0	0	0	0	0	0

K13280	SEC11, sipW; signal peptidase I [EC:3.4.21.89]	1	2	1	1	0	0	0	0	0	0
K13281	uvsE, UVE1; UV DNA damage endonuclease [EC:3.-.-.-]	0	0	1	0	0	0	0	0	0	0
K13282	cphB; cyanophycinase [EC:3.4.15.6]	1	1	1	0	0	0	0	0	0	0
K13283	fieF; ferrous-iron efflux pump FieF	0	1	0	0	0	0	0	0	0	0
K13292	lgt, umpA; phosphatidylglycerol---prolipoprotein diacylglyceryl transferase [EC:2.5.1.145]	2	1	1	1	2	1	0	1	1	1
K13479	ygeT, xdhB; xanthine dehydrogenase FAD-binding subunit [EC:1.17.1.4]	1	0	5	0	0	0	0	0	0	0
K13525	VCP, CDC48; transitional endoplasmic reticulum ATPase	0	0	1	0	0	0	0	0	0	0
K13529	ada-alkA; AraC family transcriptional regulator, regulatory protein of adaptative response / DNA-3-methyladenine glycosylase II [EC:3.2.2.21]	0	0	1	0	1	0	0	0	0	0
K13542	cobA-hemD; uroporphyrinogen III methyltransferase / synthase [EC:2.1.1.107 4.2.1.75]	1	1	1	1	1	0	0	0	0	0
K13566	NIT2, yafV; omega-amidase [EC:3.5.1.3]	1	1	2	0	0	1	0	1	1	1
K13601	bchQ; bacteriochlorophyllide d C-8(2)-methyltransferase [EC:2.1.1.332]	1	0	2	0	0	0	0	0	0	0
K13602	bchR; bacteriochlorophyllide d C-12(1)-methyltransferase [EC:2.1.1.331]	0	0	0	0	0	0	0	0	0	1
K13626	fliW; flagellar assembly factor FliW	0	0	1	0	1	0	1	0	1	1
K13641	iclR; IclR family transcriptional regulator, acetate operon repressor	0	1	4	0	0	1	1	0	1	1
K13643	iscR; Rrf2 family transcriptional regulator, iron-sulfur cluster assembly transcription factor	0	0	1	1	2	0	0	1	1	1
K13653	K13653; AraC family transcriptional regulator	0	1	2	1	2	0	0	0	1	1
K13677	dgs, bgsA; 1,2-diacylglycerol-3-alpha-glucose alpha-1,2-glucosyltransferase [EC:2.4.1.208]	1	0	0	0	0	0	0	0	0	0
K13688	chvB, cgs, ndvB; cyclic beta-1,2-glucan synthetase [EC:2.4.1.-]	1	1	1	0	0	0	0	0	0	0
K13745	ddc; L-2,4-diaminobutyrate decarboxylase [EC:4.1.1.86]	0	0	0	0	0	0	0	1	0	0
K13747	nspC; carboxynorspermidine decarboxylase [EC:4.1.1.96]	1	0	1	0	0	1	0	1	1	1
K13767	fadB; enoyl-CoA hydratase [EC:4.2.1.17]	1	0	0	0	0	0	0	0	0	0
K13770	ysiA, fadR; TetR/AcrR family transcriptional regulator, fatty acid metabolism regulator protein	1	1	1	0	0	0	0	1	0	0
K13772	rirA; Rrf2 family transcriptional regulator, iron-responsive regulator	0	0	1	0	1	0	0	0	0	0
K13789	GGPS; geranylgeranyl diphosphate synthase, type II [EC:2.5.1.1 2.5.1.10 2.5.1.29]	1	1	1	1	1	1	1	1	1	1
K13919	pduD; propanediol dehydratase medium subunit [EC:4.2.1.28]	0	0	1	0	0	0	0	0	0	0
K13920	pduE; propanediol dehydratase small subunit [EC:4.2.1.28]	0	1	1	0	0	0	0	0	0	0
K13922	pduP; propionaldehyde dehydrogenase [EC:1.2.1.87]	0	1	1	0	0	0	0	0	0	0
K13924	cheBR; two-component system, chemotaxis family, CheB/CheR fusion protein [EC:2.1.1.80 3.1.1.61]	0	0	1	0	0	0	0	0	0	0
K13953	adhP; alcohol dehydrogenase, propanol-preferring [EC:1.1.1.1]	0	0	0	0	0	0	0	1	0	0
K13954	yiaY; alcohol dehydrogenase [EC:1.1.1.1]	0	0	2	1	1	0	0	1	0	0
K13963	SERPINB; serpin B	0	1	1	0	0	0	0	0	0	0
K13993	HSP20; HSP20 family protein	1	2	3	1	1	1	0	1	2	2
K13995	nicF; maleamate amidohydrolase [EC:3.5.1.107]	0	0	3	0	0	0	0	1	0	0
K14052	puuP; putrescine importer	0	0	0	0	0	0	0	1	0	0
K14056	puuR; HTH-type transcriptional regulator, repressor for puuD	0	1	2	0	0	0	0	0	0	0
K14058	ttcA; tRNA 2-thiocytidine biosynthesis protein TtcA	1	1	2	1	1	1	1	1	1	1
K14059	int; integrase	2	0	1	2	1	0	0	0	0	0
K14080	mtaA; [methyl-Co(III) methanol/glycine betaine-specific corrinoid protein]:coenzyme M methyltransferase [EC:2.1.1.246 2.1.1.377]	1	0	8	0	1	0	1	0	0	0
K14081	mtaC; methanol corrinoid protein	0	0	1	0	0	0	0	0	0	0
K14083	mttB; trimethylamine---corrinoid protein Co-methyltransferase [EC:2.1.1.250]	22	26	88	25	57	0	0	0	0	0
K14084	mttC; trimethylamine corrinoid protein	6	1	13	6	5	0	0	0	0	0
K14086	echA; ech hydrogenase subunit A	0	0	0	0	1	0	0	0	0	0
K14087	echB; ech hydrogenase subunit B	0	0	0	0	1	0	0	0	0	0
K14088	echC; ech hydrogenase subunit C	0	0	0	1	1	0	1	0	1	1
K14089	echD; ech hydrogenase subunit D	0	0	0	1	1	0	0	0	0	0
K14090	echE; ech hydrogenase subunit E	0	0	0	1	1	0	0	0	0	0
K14091	echF; ech hydrogenase subunit F	0	0	0	1	1	0	0	0	0	0
K14127	mvhD, vhuD, vhcD; F420-non-reducing hydrogenase iron-sulfur subunit [EC:1.12.99.- 1.8.98.5 1.8.98.6]	1	1	2	0	0	1	1	0	0	0
K14138	acsB; acetyl-CoA synthase [EC:2.3.1.169]	1	1	1	1	1	0	0	0	0	0
K14155	patB, malY; cysteine-S-conjugate beta-lyase [EC:4.4.1.13]	0	0	0	1	1	0	0	1	1	0
K14170	pheA; chorismate mutase / prephenate dehydratase [EC:5.4.99.5 4.2.1.51]	0	0	0	1	1	1	1	1	1	1
K14189	dltE; uncharacterized oxidoreductase [EC:1.-.-.-]	0	0	0	0	0	0	0	0	0	1
K14250	oxyN, snoaM, dpsY, aknW, mtmY; cyclase	0	0	0	0	0	0	0	1	0	0
K14392	panF; sodium/pantothenate symporter	1	0	1	0	0	0	0	0	0	0
K14393	actP; cation/acetate symporter	0	0	0	0	0	0	0	1	0	0
K14407	CSTF2, RNA15; cleavage stimulation factor subunit 2	1	1	1	0	0	0	0	0	0	0
K14415	RTCB, rtcB; tRNA-splicing ligase RtcB (3'-phosphate/5'-hydroxy nucleic acid ligase) [EC:6.5.1.8]	0	0	1	0	0	0	0	0	0	0
K14441	rimO; ribosomal protein S12 methylthiotransferase [EC:2.8.4.4]	1	1	1	1	1	1	1	1	1	1
K14445	SLC13A2_3_5; solute carrier family 13 (sodium-dependent dicarboxylate transporter), member 2002-03-05	1	0	0	0	0	0	1	0	1	0
K14534	abfD; 4-hydroxybutyryl-CoA dehydratase / vinylacetyl-CoA-Delta-isomerase [EC:4.2.1.120 5.3.3.3]	0	1	4	0	0	0	0	0	0	0
K14540	rbgA; ribosome biogenesis GTPase A	1	1	1	1	1	1	1	1	1	1
K14623	dinD; DNA-damage-inducible protein D	1	0	0	0	0	0	0	0	0	0
K14652	ribBA; 3,4-dihydroxy 2-butanone 4-phosphate synthase / GTP cyclohydrolase II [EC:4.1.99.12 3.5.4.25]	1	0	1	1	1	1	1	0	1	1
K14665	amhX; amidohydrolase [EC:3.5.1.-]	0	0	1	0	0	0	0	0	1	1
K14730	E1.1.1.243; (-)-trans-carveol dehydrogenase [EC:1.1.1.243]	0	0	1	0	0	0	0	0	0	0
K14742	tsaB; tRNA threonylcarbamoyladenosine biosynthesis protein TsaB	1	1	1	1	1	1	1	1	1	1
K14761	ybcJ; ribosome-associated protein	0	0	0	0	0	1	1	0	0	0
K15019	K15019; 3-hydroxypropionyl-coenzyme A dehydratase [EC:4.2.1.116]	1	0	0	0	0	0	0	0	0	0
K15023	acsE; 5-methyltetrahydrofolate corrinoid/iron sulfur protein methyltransferase [EC:2.1.1.258]	1	1	1	1	1	0	0	0	0	0
K15024	K15024; putative phosphotransacetylase [EC:2.3.1.8]	2	3	3	2	2	1	0	0	1	1
K15039	K15039; 3-hydroxypropionate dehydrogenase (NADP+) [EC:1.1.1.298]	0	0	0	0	0	0	0	1	0	0
K15064	desA; syringate O-demethylase [EC:2.1.1.-]	0	0	1	0	0	0	0	0	0	0
K15268	eamA; O-acetylserine/cysteine efflux transporter	1	0	0	0	0	0	0	0	0	0
K15270	sam; S-adenosylmethionine uptake transporter	0	0	1	0	0	0	0	0	0	0
K15342	cas1; CRISP-associated protein Cas1	3	2	1	1	1	0	1	0	0	0
K15372	toa; taurine---2-oxoglutarate transaminase [EC:2.6.1.55]	0	1	1	0	0	0	1	0	0	0
K15460	yfiC, trmX; tRNA1Val (adenine37-N6)-methyltransferase [EC:2.1.1.223]	1	1	1	1	1	1	1	1	1	1
K15495	wtpA; molybdate/tungstate transport system substrate-binding protein	0	0	0	0	0	0	0	0	1	0
K15515	sauS; sulfoacetaldehyde dehydrogenase [EC:1.2.1.81]	0	0	1	0	0	0	0	1	0	0
K15521	mshA; D-inositol-3-phosphate glycosyltransferase [EC:2.4.1.250]	0	0	1	0	0	0	0	0	0	0
K15553	ssuA; sulfonate transport system substrate-binding protein	1	0	0	0	0	0	0	0	0	0
K15554	ssuC; sulfonate transport system permease protein	1	0	0	0	0	0	0	0	0	0
K15555	ssuB; sulfonate transport system ATP-binding protein [EC:7.6.2.14]	1	0	0	0	0	0	0	0	0	0
K15580	oppA, mppA; oligopeptide transport system substrate-binding protein	1	0	1	0	0	0	0	0	0	0
K15581	oppB; oligopeptide transport system permease protein	1	0	1	0	0	0	0	0	0	0
K15582	oppC; oligopeptide transport system permease protein	1	0	1	0	0	0	0	0	0	0
K15583	oppD; oligopeptide transport system ATP-binding protein	1	0	2	0	0	0	0	0	0	0
K15586	nikC, cntC; nickel transport system permease protein	0	0	0	2	0	0	0	0	0	0
K15598	thiY; putative hydroxymethylpyrimidine transport system substrate-binding protein	1	1	0	0	0	0	0	1	1	1
K15599	thiX; putative hydroxymethylpyrimidine transport system permease protein	1	1	0	0	0	0	0	1	1	1
K15600	thiZ; putative hydroxymethylpyrimidine transport system ATP-binding protein	1	0	1	0	0	0	0	1	0	0
K15633	gpmI; 2,3-bisphosphoglycerate-independent phosphoglycerate mutase [EC:5.4.2.12]	1	1	1	1	1	1	0	1	1	1
K15635	apgM; 2,3-bisphosphoglycerate-independent phosphoglycerate mutase [EC:5.4.2.12]	1	1	1	1	1	1	1	1	1	1
K15664	ppsA, fenC; plipastatin/fengycin lipopeptide synthetase A	0	0	0	0	0	1	0	1	0	0
K15727	czcB, cusB, cnrB; membrane fusion protein, heavy metal efflux system	1	1	1	0	0	0	0	0	0	0
K15738	uup; ABC transport system ATP-binding/permease protein	1	0	1	0	0	0	1	0	1	1
K15739	vanB, vanA, vanD; D-alanine---(R)-lactate ligase [EC:6.1.2.1]	0	0	1	0	1	0	0	0	1	1
K15770	cycB, ganO, mdxE; arabinogalactan oligomer / maltooligosaccharide transport system substrate-binding protein	1	0	0	1	0	0	0	1	1	0
K15778	pmm-pgm; phosphomannomutase / phosphoglucomutase [EC:5.4.2.8 5.4.2.2]	1	1	1	0	0	1	1	0	0	0
K15783	doeA; ectoine hydrolase [EC:3.5.4.44]	0	0	0	0	0	0	0	1	0	0
K15829	hycD; formate hydrogenlyase subunit 4	2	0	0	0	0	0	0	0	0	0
K15830	hycE; formate hydrogenlyase subunit 5	1	0	0	0	0	0	0	0	0	0
K15831	hycF; formate hydrogenlyase subunit 6	0	0	0	0	0	0	0	0	0	1
K15836	fhlA; formate hydrogenlyase transcriptional activator	0	0	1	0	1	0	0	0	0	0
K15858	ascF; CDP-3, 6-dideoxy-D-glycero-L-glycero-4-hexulose-4-reductase [EC:1.1.1.-]	0	0	1	0	0	0	0	0	0	0
K15866	paaG; 2-(1,2-epoxy-1,2-dihydrophenyl)acetyl-CoA isomerase [EC:5.3.3.18]	0	0	1	0	0	0	1	0	0	0
K15876	nrfH; cytochrome c nitrite reductase small subunit	1	1	0	0	0	0	0	0	0	0
K15913	pglD; UDP-N-acetylbacillosamine N-acetyltransferase [EC:2.3.1.203]	0	0	1	0	1	0	0	0	0	0
K15915	pglC; undecaprenyl phosphate N,N'-diacetylbacillosamine 1-phosphate transferase [EC:2.7.8.36]	1	0	0	0	0	0	0	0	0	0
K15916	pgi-pmi; glucose/mannose-6-phosphate isomerase [EC:5.3.1.9 5.3.1.8]	1	1	1	0	0	1	1	0	0	0
K15962	mtmC; D-mycarose 3-C-methyltransferase	0	1	0	0	0	0	0	0	0	0
K15971	tcmP, elmP; O-methyltransferase [EC:2.1.1.-]	0	0	1	0	0	0	0	0	0	0
K15973	mhqR; MarR family transcriptional regulator, 2-MHQ and catechol-resistance regulon repressor	1	0	2	0	0	0	0	0	0	0
K15986	ppaC; manganese-dependent inorganic pyrophosphatase [EC:3.6.1.1]	1	0	0	0	0	0	0	0	0	0
K15987	hppA; K(+)-stimulated pyrophosphate-energized sodium pump [EC:7.1.3.2]	2	1	2	1	2	1	0	1	1	1
K16012	cydC; ATP-binding cassette, subfamily C, bacterial CydC	0	0	1	0	0	0	0	0	0	0
K16013	cydD; ATP-binding cassette, subfamily C, bacterial CydD	0	0	1	0	0	0	0	0	0	0
K16027	asm16; methoxymalonate biosynthesis protein	0	0	0	0	1	0	0	0	0	0
K16052	ynaI, mscMJ; MscS family membrane protein	0	1	0	0	0	0	0	0	0	0
K16053	ybdG, mscM; miniconductance mechanosensitive channel	1	0	0	0	0	0	0	0	0	0
K16149	K16149; 1,4-alpha-glucan branching enzyme [EC:2.4.1.18]	1	1	1	0	0	0	0	0	0	0
K16150	K16150; glycogen synthase [EC:2.4.1.11]	1	1	1	0	0	0	0	0	0	0
K16164	K16164; acylpyruvate hydrolase [EC:3.7.1.5]	1	0	0	0	0	0	0	0	1	1
K16169	pbuX; xanthine permease	0	0	3	0	0	0	0	0	0	0
K16176	mtmB; methylamine---corrinoid protein Co-methyltransferase [EC:2.1.1.248]	6	0	6	1	2	0	0	0	0	0
K16178	mtbB; dimethylamine---corrinoid protein Co-methyltransferase [EC:2.1.1.249]	3	4	14	10	6	0	0	0	0	0

K16179	mtbC; dimethylamine corrinoid protein	1	0	7	0	0	0	1	0	0	0
K16180	pylB; methylornithine synthase [EC:5.4.99.58]	1	1	4	1	1	0	0	0	0	0
K16181	pylC; 3-methylornithine--L-lysine ligase [EC:6.3.2.59]	1	1	2	1	1	0	0	0	0	0
K16182	pylD; 3-methylornithyl-N6-L-lysine dehydrogenase [EC:1.4.1.-]	1	0	1	1	1	0	0	0	0	0
K16188	ytpB; tetraprenyl-beta-curcumene synthase [EC:4.2.3.130]	1	1	1	0	0	0	0	0	0	0
K16238	eat, eutP; ethanolamine permease	1	0	3	0	0	0	0	1	0	0
K16306	K16306; fructose-bisphosphate aldolase / 2-amino-3,7-dideoxy-D-threo-hept-6-ulosonate synthase [EC:4.1.2.13 2.2.1.10]	0	0	1	0	0	0	0	0	0	0
K16327	K16327; putative LysE/RhtB family amino acid efflux pump	0	0	1	0	0	0	0	0	0	0
K16416	mxaA; myxalamid-type nonribosomal peptide synthetase MxaA	0	0	0	0	0	0	0	0	1	0
K16783	bioN; biotin transport system permease protein	0	0	0	0	0	1	0	0	0	0
K16784	bioM; biotin transport system ATP-binding protein [EC:7.6.2.-]	0	0	0	0	0	1	0	0	0	0
K16785	ecfT; energy-coupling factor transport system permease protein	3	4	6	1	4	1	2	3	5	7
K16786	ecfA1; energy-coupling factor transport system ATP-binding protein [EC:7.-.-.-]	1	1	3	0	1	0	1	2	3	4
K16787	ecfA2; energy-coupling factor transport system ATP-binding protein [EC:7.-.-.-]	1	1	3	0	1	0	1	2	3	4
K16788	niaX; niacin transporter	0	0	0	0	0	0	1	0	1	0
K16841	hpxA; allantoin racemase [EC:5.1.99.3]	0	1	3	0	0	0	0	0	0	0
K16845	suyA; (2R)-sulfolactate sulfo-lyase subunit alpha [EC:4.4.1.24]	2	0	0	0	0	0	1	0	0	0
K16846	suyB; (2R)-sulfolactate sulfo-lyase subunit beta [EC:4.4.1.24]	2	0	0	0	0	0	0	0	0	0
K16849	uxaA1; altronate dehydratase small subunit [EC:4.2.1.7]	0	0	2	0	0	0	0	0	0	0
K16850	uxaA2; altronate dehydratase large subunit [EC:4.2.1.7]	0	0	2	0	0	0	1	0	0	0
K16874	hmfF; 2,5-furandicarboxylate decarboxylase 1	0	0	3	0	1	1	0	0	0	0
K16881	K16881; mannose-1-phosphate guanylyltransferase / phosphomannomutase [EC:2.7.7.13 5.4.2.8]	1	1	1	0	0	0	0	0	0	0
K16898	addA; ATP-dependent helicase/nuclease subunit A [EC:5.6.2.4 3.1.-.-]	1	1	1	1	1	1	1	1	1	1
K16899	addB; ATP-dependent helicase/nuclease subunit B [EC:5.6.2.4 3.1.-.-]	1	1	1	0	0	1	0	1	1	1
K16904	DCTPP1; dCTP diphosphatase [EC:3.6.1.12]	0	0	1	1	0	0	0	0	0	0
K16905	K16905; fluoroquinolone transport system permease protein	1	0	0	0	0	0	0	0	0	0
K16906	K16906; fluoroquinolone transport system permease protein	1	0	0	0	0	0	0	0	0	0
K16907	K16907; fluoroquinolone transport system ATP-binding protein [EC:7.6.2.-]	1	0	0	0	0	0	0	0	0	0
K16923	qrtT; energy-coupling factor transport system substrate-specific component	0	0	1	0	0	0	1	0	0	0
K16925	ykoE; energy-coupling factor transport system permease protein	0	0	2	0	0	0	0	0	2	3
K16926	htsT; energy-coupling factor transport system substrate-specific component	0	0	0	0	1	0	0	0	0	0
K16927	cbrT; energy-coupling factor transport system substrate-specific component	1	2	1	0	0	0	1	1	0	1
K16937	doxD; thiosulfate dehydrogenase (quinone) large subunit [EC:1.8.5.2]	0	0	2	0	0	0	0	0	0	0
K16950	asrA; anaerobic sulfite reductase subunit A	1	1	2	0	0	1	1	0	0	0
K16951	asrB; anaerobic sulfite reductase subunit B	1	0	2	0	0	1	1	0	0	0
K16957	tcyK; L-cystine transport system substrate-binding protein	0	0	2	0	0	0	0	0	0	0
K16959	tcyM; L-cystine transport system permease protein	0	0	2	0	0	0	0	0	0	0
K16960	tcyN; L-cystine transport system ATP-binding protein [EC:7.4.2.1]	0	0	2	0	0	0	0	0	0	0
K17073	lysX1; putative lysine transport system substrate-binding protein	0	0	0	1	2	0	0	0	0	0
K17074	lysX2; putative lysine transport system permease protein	0	0	0	1	1	0	0	0	0	0
K17076	lysY; putative lysine transport system ATP-binding protein	0	0	0	1	1	0	0	0	0	0
K17103	CHO1, pssA; CDP-diacylglycerol---serine O-phosphatidyltransferase [EC:2.7.8.8]	1	1	1	0	0	0	0	0	0	0
K17218	sqr; sulfide:quinone oxidoreductase [EC:1.8.5.4]	1	1	1	0	0	0	0	0	0	0
K17248	pglJ; N-acetylgalactosamine-N,N'-diacetylbacillosaminyl-diphospho-undecaprenol 4-alpha-N-acetylgalactosaminyltransferase [EC:2.4.1.291]	0	0	0	0	1	0	0	0	0	0
K17472	cymR; Rrf2 family transcriptional regulator, cysteine metabolism repressor	1	1	2	0	0	0	1	2	1	1
K17675	SUPV3L1, SUV3; ATP-dependent RNA helicase SUPV3L1/SUV3 [EC:3.6.4.13]	0	0	0	0	0	0	0	0	1	0
K17686	copA, ctpA, ATP7; P-type Cu+ transporter [EC:7.2.2.8]	1	1	2	0	1	1	2	1	1	1
K17723	preA; dihydropyrimidine dehydrogenase (NAD+) subunit PreA [EC:1.3.1.1]	0	0	6	0	0	0	0	0	1	1
K17732	PMPCB, MAS1; mitochondrial-processing peptidase subunit beta [EC:3.4.24.64]	1	1	1	1	1	0	1	1	1	1
K17733	cwlK; peptidoglycan LD-endopeptidase CwlK [EC:3.4.-.-]	0	0	1	0	0	0	0	0	0	0
K17754	cpnA; cyclopentanol dehydrogenase [EC:1.1.1.163]	0	0	1	0	0	0	0	0	0	0
K17828	pyrDI; dihydroorotate dehydrogenase (NAD+) catalytic subunit [EC:1.3.1.14]	1	0	2	1	1	1	1	1	1	1
K17830	GGR; digeranylgeranylglycerophospholipid reductase [EC:1.3.1.101 1.3.7.11]	0	0	2	1	0	0	0	0	0	0
K17865	croR; 3-hydroxybutyryl-CoA dehydratase [EC:4.2.1.55]	0	0	1	0	0	0	0	0	0	0
K17992	hndB; NADP-reducing hydrogenase subunit HndB [EC:1.12.1.3]	1	1	2	0	0	0	0	0	0	0
K17995	hydG; sulfhydrogenase subunit gamma (sulfur reductase) [EC:1.12.98.4]	0	0	1	0	0	0	0	0	0	0
K18009	budC; meso-butanediol dehydrogenase / (S,S)-butanediol dehydrogenase / diacetyl reductase [EC:1.1.1.- 1.1.1.76 1.1.1.304]	0	0	0	0	0	0	0	1	0	0
K18011	kamE; beta-lysine 5,6-aminomutase beta subunit [EC:5.4.3.3]	0	0	1	0	0	0	0	0	0	0
K18012	kdd; L-erythro-3,5-diaminohexanoate dehydrogenase [EC:1.4.1.11]	0	0	1	0	0	0	0	0	0	0
K18013	kce; 3-keto-5-aminohexanoate cleavage enzyme [EC:2.3.1.247]	0	0	2	0	0	0	0	2	0	0
K18118	aarC, cat1; succinyl-CoA:acetate CoA-transferase [EC:2.8.3.18]	0	0	1	1	1	1	0	0	1	1
K18122	cat2, abfT; 4-hydroxybutyrate CoA-transferase [EC:2.8.3.-]	1	1	2	0	0	0	0	1	0	0
K18148	rtcB; release factor H-coupled RctB family protein	0	0	1	0	0	0	0	0	0	0
K18164	NDUFAF7; NADH dehydrogenase [ubiquinone] 1 alpha subcomplex assembly factor 7	1	1	0	0	0	0	0	0	0	0
K18166	FOXRED1; FAD-dependent oxidoreductase domain-containing protein 1	0	0	1	0	0	0	0	0	0	0
K18189	TACO1; translational activator of cytochrome c oxidase 1	0	0	0	1	1	0	0	1	0	0
K18199	inhA; cyclohexyl-isocyanide hydratase [EC:4.2.1.103]	0	0	2	0	0	0	0	1	1	0
K18209	tfrA; fumarate reductase (CoM/CoB) subunit A [EC:1.3.4.1]	0	0	1	0	0	0	0	0	0	0
K18231	msr, vmlR; macrolide transport system ATP-binding/permease protein	1	0	0	0	0	0	0	0	0	0
K18234	vat; virginiamycin A acetyltransferase [EC:2.3.1.-]	0	0	0	0	0	0	0	0	1	1
K18290	ich-Y; itaconyl-CoA hydratase [EC:4.2.1.56]	0	0	2	0	0	0	0	0	0	0
K18302	mexJ; membrane fusion protein, multidrug efflux system	0	0	1	0	0	0	0	0	0	0
K18303	mexK; multidrug efflux pump	0	0	1	0	0	0	0	0	0	0
K18325	ramA; AraC family of transcriptional regulator, multidrug resistance transcriptional activator	1	0	0	0	0	0	0	0	0	0
K18330	hndA; NADP-reducing hydrogenase subunit HndA [EC:1.12.1.3]	2	1	1	0	0	1	0	0	0	0
K18331	hndC; NADP-reducing hydrogenase subunit HndC [EC:1.12.1.3]	0	0	0	0	0	0	1	0	0	0
K18332	hndD; NADP-reducing hydrogenase subunit HndD [EC:1.12.1.3]	0	0	0	0	0	0	1	0	1	2
K18345	vanSB, vanS, vanSD; two-component system, OmpR family, sensor histidine kinase VanS [EC:2.7.13.3]	1	0	1	0	0	0	0	0	0	0
K18346	vanW; vancomycin resistance protein VanW	1	0	2	0	1	0	0	0	1	1
K18347	vanH; D-specific alpha-keto acid dehydrogenase [EC:1.1.1.-]	0	0	1	0	1	0	0	0	1	1
K18349	vanRC, vanRE, vanRG; two-component system, OmpR family, response regulator VanR	1	1	2	0	2	0	0	0	1	1
K18350	vanSC, vanSE, vanSG; two-component system, OmpR family, sensor histidine kinase VanS [EC:2.7.13.3]	0	0	1	0	1	0	0	0	1	1
K18359	padH; phenylglyoxylate dehydrogenase epsilon subunit [EC:1.2.1.58]	0	0	2	0	0	0	0	0	0	0
K18367	CoADR; CoA-dependent NAD(P)H sulfur oxidoreductase [EC:1.8.1.18]	1	1	1	1	0	0	0	0	0	0
K18369	adh2; alcohol dehydrogenase [EC:1.1.1.-]	0	0	0	0	0	1	0	0	0	0
K18429	legG, neuC2; GDP/UDP-N,N'-diacetylbacillosamine 2-epimerase (hydrolysing) [EC:3.2.1.184]	0	0	1	0	0	0	0	0	0	0
K18430	legI, neuB2; N,N'-diacetyllegionaminate synthase [EC:2.5.1.101]	0	0	1	0	0	0	0	0	0	0
K18431	legF, ptmB; CMP-N,N'-diacetyllegionaminic acid synthase [EC:2.7.7.82]	0	0	1	0	0	0	0	0	0	0
K18445	ndx1; diadenosine hexaphosphate hydrolase (ATP-forming) [EC:3.6.1.61]	1	0	1	1	1	0	0	0	0	0
K18456	E3.5.4.32; 8-oxoguanine deaminase [EC:3.5.4.32]	0	0	1	0	0	0	0	0	0	0
K18471	ydjG; methylglyoxal reductase [EC:1.1.1.-]	0	0	1	0	0	0	0	0	0	0
K18501	hmeD; heterodisulfide reductase iron-sulfur subunit	1	1	1	0	0	0	0	0	0	0
K18563	thnL; methyltransferase [EC:2.1.1.-]	0	0	1	0	0	0	0	0	0	0
K18608	ppaT; pyridoxamine---pyruvate transaminase [EC:2.6.1.30]	0	0	0	0	0	0	0	0	2	3
K18672	dacA; diadenylate cyclase [EC:2.7.7.85]	1	1	1	1	1	1	1	1	1	1
K18682	rny; ribonucrease Y [EC:3.1.-.-]	1	1	1	0	0	0	1	1	1	1
K18700	flK; fluoroacetyl-CoA thioesterase [EC:3.1.2.29]	0	0	1	0	0	0	0	0	0	0
K18702	uctC; CoA:oxalate CoA-transferase [EC:2.8.3.19]	0	0	3	0	0	0	0	0	0	0
K18707	mtaB; threonylcarbamoyladenosine tRNA methylthiotransferase MtaB [EC:2.8.4.5]	1	1	1	1	1	1	1	1	1	1
K18785	mp2; beta-1,4-mannooligosaccharide/beta-1,4-mannosyl-N-acetylglucosamine phosphorylase [EC:2.4.1.319 2.4.1.320]	0	0	0	0	0	1	0	0	0	0
K18814	ictB; putative inorganic carbon (hco3(-)) transporter	0	0	0	1	1	0	0	0	0	0
K18889	mdlA, smdA; ATP-binding cassette, subfamily B, multidrug efflux pump	1	0	0	0	0	0	0	0	0	0
K18906	mgrA; MarR family transcriptional regulator, multiple gene regulator MgrA	0	0	0	1	0	0	0	0	0	0
K18908	mepA; multidrug efflux pump	0	0	0	0	0	0	0	0	1	1
K18924	ykkC; paired small multidrug resistance pump	1	0	0	0	0	0	0	0	0	0
K18925	ykkD; paired small multidrug resistance pump	1	0	0	0	0	0	0	0	0	0
K18934	lmrS; MFS transporter, DHA2 family, multidrug resistance protein	0	0	0	0	0	1	0	0	0	0
K18935	sdrM; MFS transporter, DHA2 family, multidrug resistance protein	1	0	1	0	0	0	0	0	0	0
K18940	arlS; two-component system, OmpR family, sensor histidine kinase ArlS [EC:2.7.13.3]	1	0	0	0	0	0	0	0	0	0
K18941	arlR; two-component system, OmpR family, response regulator ArlR	1	0	1	0	1	1	0	0	0	0
K18979	queG; epoxyqueuosine reductase [EC:1.17.99.6]	1	0	2	0	0	0	0	0	0	0
K18997	cbpM; chaperone modulatory protein CbpM	1	0	0	0	0	0	0	0	0	0
K19002	mgs, bgsB; 1,2-diacylglycerol 3-alpha-glucosyltransferase [EC:2.4.1.337]	1	0	0	0	1	0	0	0	0	0
K19005	ltaS; lipoteichoic acid synthase [EC:2.7.8.20]	0	0	0	0	0	0	0	0	1	1
K19055	prdX, proX; Ala-tRNA(Pro) deacylase [EC:3.1.1.-]	1	0	1	0	0	0	0	0	0	0
K19068	wbjC; UDP-2-acetamido-2,6-beta-L-arabino-hexul-4-ose reductase [EC:1.1.1.367]	1	0	0	0	0	0	0	0	0	0
K19075	cst2, cas7; CRISPR-associated protein Cst2	0	0	1	0	0	0	1	0	0	0
K19081	braS, bceS; two-component system, OmpR family, sensor histidine kinase BraS/BceS [EC:2.7.13.3]	0	0	0	0	1	0	0	0	1	1
K19082	braR, bceR; two-component system, OmpR family, response regulator protein BraR/BceR	0	0	0	0	0	0	0	0	1	1
K19090	cas5t; CRISPR-associated protein Cas5t	0	0	1	0	0	0	1	0	0	0
K19091	cas6; CRISPR-associated endoribonuclease Cas6 [EC:3.1.-.-]	2	2	1	0	0	0	1	0	0	0
K19092	parE1_3_4; toxin ParE1/3/4	1	0	2	0	0	0	0	0	0	0
K19114	csh1; CRISPR-associated protein Csh1	1	1	0	0	0	0	0	0	0	0
K19115	csh2; CRISPR-associated protein Csh2	2	1	0	0	0	0	0	0	0	0
K19116	cas5h; CRISPR-associated protein Cas5h	2	1	0	0	0	0	0	0	0	0
K19117	csd1, cas8c; CRISPR-associated protein Csd1	0	1	0	0	1	0	0	1	0	0

K19118	csd2, cas7; CRISPR-associated protein Csd2	0	1	0	0	1	0	0	2	0	0
K19119	cas5d; CRISPR-associated protein Cas5d	0	0	0	0	1	0	0	1	0	0
K19134	csx10; CRISPR-associated protein Csx10	1	0	0	0	0	0	0	0	0	0
K19137	csn2; CRISPR-associated protein Csn2	1	0	0	0	0	0	0	0	0	0
K19138	csm2; CRISPR-associated protein Csm2	0	1	0	0	0	0	0	0	0	0
K19139	csm4; CRISPR-associated protein Csm4	0	1	0	0	0	0	0	0	0	0
K19140	csm5; CRISPR-associated protein Csm5	0	1	0	0	0	0	0	0	0	0
K19141	cmr5; CRISPR-associated protein Cmr5	1	0	0	0	0	0	0	0	0	0
K19147	mcrC; 5-methylcytosine-specific restriction enzyme subunit McrC	1	0	0	0	0	0	0	1	0	1
K19157	yafQ; mRNA interferase YafQ [EC:3.1.-.-]	2	0	1	0	0	0	0	0	0	0
K19158	yoeB; toxin YoeB [EC:3.1.-.-]	1	0	1	0	0	0	0	0	0	0
K19159	yefM; antitoxin YefM	1	0	0	0	0	0	0	0	0	0
K19171	dndD; DNA sulfur modification protein DndD	0	0	1	0	0	0	0	0	0	0
K19200	IAL; isopenicillin-N N-acyltransferase like protein	0	0	1	0	0	0	0	0	0	0
K19221	cobA, btuR; cob(I)alamin adenosyltransferase [EC:2.5.1.17]	1	0	4	1	1	1	1	1	1	1
K19222	menI, DHNAT; 1,4-dihydroxy-2-naphthoyl-CoA hydrolase [EC:3.1.2.28]	0	0	0	0	0	0	0	1	0	0
K19224	lytE, cwlF; peptidoglycan DL-endopeptidase LytE [EC:3.4.-.-]	1	1	1	0	0	0	0	0	0	0
K19239	sapZ; predicted membrane protein	1	1	1	0	0	0	0	0	0	0
K19244	ala; alanine dehydrogenase [EC:1.4.1.1]	0	0	1	0	0	0	0	0	0	0
K19270	hxpA; mannitol-1-/sugar-/sorbitol-6-phosphatase [EC:3.1.3.22 3.1.3.23 3.1.3.50]	1	0	0	0	0	0	0	1	0	0
K19294	algI; alginate O-acetyltransferase complex protein AlgI	1	0	1	0	1	0	0	1	1	1
K19302	bcrC; undecaprenyl-diphosphatase [EC:3.6.1.27]	2	1	3	1	1	1	0	1	1	1
K19333	kdgR; IclR family transcriptional regulator, KDG regulon repressor	0	0	2	0	1	0	0	0	0	0
K19350	lsa; lincosamide and streptogramin A transport system ATP-binding/permease protein	0	0	1	0	1	0	0	0	0	1
K19405	mcsB; protein arginine kinase [EC:2.7.14.1]	1	1	1	1	1	1	1	1	0	0
K19411	mcsA; protein arginine kinase activator	1	1	1	1	1	1	0	1	0	0
K19421	epsC; polysaccharide biosynthesis protein EpsC	1	1	2	0	1	1	0	0	0	0
K19422	epsD; glycosyltransferase EpsD [EC:2.4.-.-]	1	0	0	0	1	1	0	0	0	0
K19423	epsE; glycosyltransferase EpsE [EC:2.4.-.-]	1	0	0	0	0	0	0	0	0	0
K19424	epsF; glycosyltransferase EpsF [EC:2.4.-.-]	0	0	0	0	0	1	0	0	0	0
K19425	epsH; glycosyltransferase EpsH [EC:2.4.-.-]	1	0	0	0	0	0	0	0	0	0
K19427	epsJ; glycosyltransferase EpsJ [EC:2.4.-.-]	1	0	0	0	1	0	0	0	0	0
K19428	epsL; sugar transferase EpsL [EC:2.-.-.-]	0	0	1	0	1	0	0	0	0	0
K19430	epsN; pyridoxal phosphate-dependent aminotransferase EpsN [EC:2.6.1.-]	1	0	1	0	1	1	0	0	0	0
K19545	lnuA_C_D_E, lin; lincosamide nucleotidyltransferase A/C/D/E	0	0	0	0	1	0	0	0	0	0
K19689	ampS, pepS, ampT; aminopeptidase [EC:3.4.11.-]	1	1	1	1	1	1	1	1	1	0
K19709	ydiF; acetate CoA-transferase [EC:2.8.3.8]	0	0	1	0	0	0	0	0	0	0
K19736	aefR; TetR/AcrR family transcriptional regulator, regulator of autoinduction and epiphytic fitness	0	0	0	0	0	0	0	1	0	0
K19784	chrR, NQR; chromate reductase, NAD(P)H dehydrogenase (quinone)	1	0	1	0	1	0	0	0	0	0
K19802	ycjG, ykfB; L-Ala-D/L-Glu epimerase [EC:5.1.1.20]	0	0	1	0	0	0	0	0	0	0
K19814	eam; glutamate 2,3-aminomutase [EC:5.4.3.9]	2	1	0	0	0	0	0	0	0	0
K19955	adh2; alcohol dehydrogenase [EC:1.1.1.-]	1	0	0	0	0	1	1	1	0	0
K19960	chnA; cyclohexanol dehydrogenase [EC:1.1.1.245]	1	0	0	0	0	0	0	0	0	0
K20038	cutC; choline trimethylamine-lyase [EC:4.3.99.4]	1	1	1	0	0	0	0	0	0	0
K20074	prpC, phpP; PPM family protein phosphatase [EC:3.1.3.16]	1	1	1	1	2	1	1	1	1	1
K20156	sgcG; 2-amino-4-deoxychorismate dehydrogenase [EC:1.3.8.16]	2	0	0	0	0	0	0	0	0	0
K20265	gadC; glutamate:GABA antiporter	0	0	0	2	2	0	0	0	0	0
K20429	vioA; dTDP-4-amino-4,6-dideoxy-D-glucose transaminase [EC:2.6.1.33]	1	0	1	0	0	0	0	0	0	0
K20445	ndhF; nicotinate dehydrogenase FAD-subunit [EC:1.17.1.5]	0	1	4	0	0	1	0	0	0	0
K20446	ndhS; nicotinate dehydrogenase small FeS subunit [EC:1.17.1.5]	0	0	1	0	0	0	0	0	0	0
K20447	ndhL; nicotinate dehydrogenase large molybdopterin subunit [EC:1.17.1.5]	2	3	9	0	0	1	1	3	0	0
K20448	ndhM; nicotinate dehydrogenase medium molybdopterin subunit [EC:1.17.1.5]	0	0	1	0	0	0	0	1	0	0
K20449	hnr; 6-hydroxynicotinate reductase [EC:1.3.7.1]	0	0	1	0	0	0	0	0	0	0
K20451	mii; methylitaconate Delta-isomerase [EC:5.3.3.6]	0	0	4	0	0	0	0	0	0	0
K20454	dml; 2,3-dimethylmalate lyase [EC:4.1.3.32]	0	0	0	0	0	0	0	1	0	0
K20459	nukF, mcdF, sboF; lantibiotic transport system ATP-binding protein	0	0	0	0	0	0	0	0	1	0
K20460	nukE, mcdE, sboE; lantibiotic transport system permease protein	0	0	0	0	0	0	0	0	1	0
K20487	nisK, spaK; two-component system, OmpR family, lantibiotic biosynthesis sensor histidine kinase NisK/SpaK [EC:2.7.13.3]	1	0	0	0	0	0	0	0	1	1
K20488	nisR, spaR; two-component system, OmpR family, lantibiotic biosynthesis response regulator NisR/SpaR	1	0	0	0	1	0	0	0	1	1
K20609	sgcX; putative aminopeptidase [EC:3.4.11.-]	0	0	1	0	0	0	0	0	0	0
K20625	ahy; acetylene hydratase [EC:4.2.1.112]	0	0	3	0	0	0	0	0	0	0
K20680	fdtB; dTDP-3-amino-3,6-dideoxy-alpha-D-galactopyranose transaminase [EC:2.6.1.90]	0	0	1	0	0	0	0	0	0	0
K20763	naaA; 5-nitroanthranilic acid aminohydrolase [EC:3.5.99.8]	0	0	1	0	0	0	0	0	0	0
K20859	phnPP; phosphoribosyl 1,2-cyclic phosphate 1,2-diphosphodiesterase [EC:3.1.4.57]	0	0	1	0	0	0	0	0	0	0
K20882	hadA; (R)-2-hydroxy-4-methylpentanoate CoA-transferase [EC:2.8.3.24]	1	0	0	0	0	0	0	0	0	0
K20922	vpsI; polysaccharide biosynthesis protein VpsI	1	0	0	0	0	0	0	0	0	0
K21020	siaD; diguanylate cyclase [EC:2.7.7.65]	0	0	1	0	0	0	0	0	0	0
K21022	roeA; diguanylate cyclase [EC:2.7.7.65]	0	0	0	0	0	0	0	1	0	0
K21028	ynjE; molybdopterin synthase sulfurtransferase [EC:2.8.1.11]	0	0	1	0	0	0	0	0	0	0
K21029	moeB; molybdopterin-synthase adenylyltransferase [EC:2.7.7.80]	0	1	1	0	0	0	0	0	1	2
K21030	tarI; D-ribitol-5-phosphate cytidylyltransferase [EC:2.7.7.40]	0	0	0	1	1	0	0	0	0	0

Table S5. Recipe for trace element solution A, 500 mL solution

Description	Volume (mg, unless stated)
HCl	10 mL
FeCl2.4H_2O	750
$CoCl_2.6H_2O$	95
$MnCl_2.4H_2O$	50
$ZnCl_2$	35
H_3BO_3	3
$Na_2MoO_4.H_2O$	18
$NiCl_2.6H_2O$	12
$CuCl_2.2H_2O$	1
Water (MilliQ)	500 mL

Table S6. Recipe for trace element solution B, 500 mL solution

Description	Volume (mg, unless stated)
$Na_2SeO_3.5H_2O$	3
$NaWO_4.2H_2O$	4
NaOH	250
Water (MilliQ)	500 mL

Table S7. Recipe for 1000X vitamin, 1L solution. After dissolving all the reagents in the water, the solution water was filter-sterilized three times into serum bottles and capped with sterile septa. The solution was then purged with sterile N_2 for 30 minutes.

Description	Volume (mg, unless stated)
p-aminobenzoate	20
Biotin	5
Nicotinic acid	50
Pantothenic acid	25
Pyridoxine	75
Thiamine	50
Cyanocobalamin	50
Water (MilliQ)	1 L

FASP Protocol

All centrifugation steps were done at 14,000 rcf for 10 minutes unless indicated otherwise.

1. Each extract sample was diluted to a final concentration of 0.8 μg/μl using ammonium bicarcbonate buffer (ABC, 0.05 M stock, pH 9.0). The proteins in the diluted sample were denatured by adding dithioreitol to a final concentration of 5 mM and incubating the mixture at 30° C for 30 minutes.

2. The samples were mixed with 200 μl of urea solution (UA, 8 M stock) and the filter units were centrifugated twice. The flow-through from each filter unit was discarded from the collection tube after each centrifugation step.

3. 100 μl of iodoacetamide solution (IAA, 0.05 M) was added to the filter units and was mixed using a thermomixer temperature at 600 rpm. The filter unites were incubated for 20 minutes in the dark before being centrifugated.

4. 100 μl of UA was added to the filter units which were then centrifugated twice.

5. 100 μl of ABC was added to the filter units which were then centrifugated twice.

6. 40 μl of ABC and 0.9 μl of trypsin stock solution (0.4 ng/μl) were added to the filter units and were mixed at 600 rpm in thermomixer for 1 minute. The flow-through from each filter unit was then discarded from the collection tube.

7. 500 μl of MilliQ H_2O was added to the filter units, which were incubated at 37° C in dark for 12 hours.

8. The filter component from each unit was relocated to a new container tube and was centrifugated.

9. 20 μl of ABC was added to the filter units, which were then centrifugated twice. The flow-through containing peptides were transferred to 1.5 mL tubes and were stored at -20° C until proteomics analysis.

Table S8. List of proteins identified in all three replicates of *D. formicoaceticum* grown in DCM

The protein sequence coverage (95%) refers to the percentage of matching amino acids from the identified peptides having confidence greater than or equal to 95% divided by the total number of amino acids in the sequence

				Protein Scores			Number of peptides			Protein sequence coverage (95%)		
**NCBI Protein ID**	**Protein Description**	**Protein Name**	**Pathway**	**Average**	**STDEV**	**% STDEV**	**Average**	**STDEV**	**% STDEV**	**Average**	**STDEV**	**% STDEV**
WP_089610968.1	(R)-phenyllactyl-CoA dehydratase beta subunit		Amino Acid fermentation - L Phenyl -Lactate	31.1	2.8	0.1	19.3	3.3	0.2	49.4	5.6	0.1
WP_089610974.1	Methionine synthase	MetH	Amino acid synthesis	9.0	0.9	0.1	5.0	1.4	0.3	25.4	8.2	0.3
WP_242965479.1	Branched-chain-amino-acid aminotransferase 2		Amino Acids	29.5	4.5	0.2	20.3	2.1	0.1	50.6	6.5	0.1
WP_089612575.1	S-adenosylmethionine synthase		Amino Acids - Enzyme cofactors	30.1	4.5	0.1	17.0	2.2	0.1	46.1	5.8	0.1
WP_089610166.1	Sulfoacetaldehyde acetyltransferase	Xsc	Amino Acids	37.0	2.0	0.1	20.7	1.7	0.1	32.0	1.9	0.1
WP_089611605.1	Hypotaurine/taurine--pyruvate aminotransferase		Amino Acids - fermentation, pyruvate producting	31.9	4.7	0.1	22.7	2.5	0.1	46.0	4.7	0.1
WP_089609398.1	NADP-specific glutamate dehydrogenase		Amino Acids - Glutamate	56.1	4.4	0.1	72.3	5.2	0.1	67.7	3.7	0.1
WP_089609238.1	LL-diaminopimelate aminotransferase	DAP	Amino Acids - Lysine	33.1	2.9	0.1	22.7	2.1	0.1	55.8	8.5	0.2
WP_089609252.1	Diaminopimelate decarboxylase		Amino Acids - Lysine	35.2	3.9	0.1	27.7	0.5	0.0	47.0	1.6	0.0
WP_198306497.1	Malonate-semialdehyde dehydrogenase	IolA	Amino Acids - beta-alanine, alanine, aspartate	38.7	4.5	0.1	24.0	3.6	0.1	44.5	3.4	0.1
WP_089609063.1	Methionine synthase	MetH	Amino Acids - Methionine	42.9	4.2	0.1	102.3	11.3	0.1	84.4	6.7	0.1
WP_089612390.1	Methionine synthase	MetH	Amino Acids - Methionine	30.2	0.8	0.0	46.7	0.9	0.0	78.8	6.1	0.1
WP_089611707.1	Tryptophan synthase beta chain		Amino Acids - Tryptophan	49.0	2.2	0.0	39.0	2.2	0.1	57.1	4.2	0.1
WP_089612137.1	Carbamoyl-phosphate synthase large chain		Amio Acids - Arginine and pyrimidine precursor	52.1	5.7	0.1	29.7	2.5	0.1	27.8	3.1	0.1
WP_242965392.1	Methenyltetrahydromethanopterin cyclohydrolase	Mch	C1 pathway	2.3	0.1	0.0	1.0	0.0	0.0	4.6	0.0	0.0
WP_089608723.1	methanol--corrinoid methyltransferase	MtaB	C1 pathway	49.7	1.7	0.0	49.3	3.1	0.1	61.4	4.1	0.1
WP_157677303.1	Methylcorrinoid:tetrahydrofolate methyltransferase	MtrH	C1 pathway	52.1	2.5	0.0	65.0	1.6	0.0	73.0	2.7	0.0
WP_089608939.1	Glycine betaine methyltransferase	MttB	C1 pathway	12.5	1.3	0.1	7.7	0.5	0.1	18.9	1.7	0.1
WP_089609966.1	Glycine betaine methyltransferase	MttB	C1 pathway	1.9	0.2	0.1	1.0	0.0	0.0	1.9	0.0	0.0
WP_198306644.1	Glycine betaine methyltransferase	MttB	C1 pathway	8.9	3.6	0.4	6.7	2.5	0.4	49.6	14.5	0.3
WP_198306644.1	Glycine betaine methyltransferase	MttB	C1 pathway	13.5	1.4	0.1	7.7	0.5	0.1	33.5	2.8	0.1
WP_089612392.1	Glycine betaine methyltransferase	MttB	C1 pathway	15.6	3.3	0.2	8.7	1.2	0.1	23.4	4.1	0.2
WP_089608568.1	Chaperone protein DnaK		Chaperone	80.7	5.5	0.1	49.0	3.3	0.1	52.6	7.9	0.2
WP_089608728.1	60 kDa chaperonin		Chaperone	70.9	4.1	0.1	77.0	7.3	0.1	65.0	4.7	0.1
WP_089608882.1	Chaperone protein ClpB		Chaperone	48.6	1.9	0.0	30.0	1.4	0.0	36.0	0.9	0.0
WP_089608885.1	Chaperone protein dnaK2		Chaperone	54.4	2.4	0.0	32.7	3.3	0.1	49.5	3.4	0.1
WP_089609257.1	Chaperone protein HtpG		Chaperone	57.6	9.9	0.2	33.0	3.6	0.1	50.8	4.0	0.1
WP_089612357.1	Siroheme synthase	CysG	Cofactor biosynthesis	31.5	3.6	0.1	16.7	1.2	0.1	37.3	4.7	0.1
WP_089609442.1	Negative regulator of genetic competence ClpC/MecB		Competence	70.0	7.4	0.1	39.0	3.6	0.1	47.4	4.3	0.1
WP_089611628.1	NAD(P)H-quinone oxidoreductase subunit J, chloroplastic	NuoC	Complex I	4.3	2.4	0.6	2.7	0.9	0.4	31.1	12.3	0.4
WP_089611627.1	NADH-quinone oxidoreductase subunit 4	NuoD	Complex I	15.6	4.4	0.3	8.3	1.7	0.2	27.6	4.9	0.2
WP_089611863.1	Na(+)-translocating NADH-quinone reductase subunit F	NuoF	Complex I	10.5	1.0	0.1	5.0	0.0	0.0	12.3	1.0	0.1
WP_089611623.1	NAD(P)H-quinone oxidoreductase subunit I, chloroplastic	NuoI	Complex I	5.7	0.2	0.0	3.0	0.0	0.0	30.0	0.6	0.0
WP_198306733.1	NADH-quinone oxidoreductase subunit K	NuoK	Complex I	2.0	0.0	0.0	1.0	0.0	0.0	12.7	0.0	0.0
WP_089611617.1	NADH-quinone oxidoreductase subunit L	NuoL	Complex I	4.2	1.4	0.3	3.3	0.5	0.1	5.8	0.7	0.1
WP_089611615.1	NAD(P)H-quinone oxidoreductase chain 4 1	NuoM	Complex I	4.7	0.7	0.1	3.0	0.8	0.3	6.1	0.8	0.1
WP_089611614.1	NAD(P)H-quinone oxidoreductase subunit 2	NuoN	Complex I	3.2	0.3	0.1	2.3	0.5	0.2	6.9	0.8	0.1
WP_089612431.1	Sulfite reductase, dissimilatory-type subunit alpha	DsrA	Dissimilatory sulfite reduction	40.5	4.0	0.1	25.0	0.8	0.0	51.0	2.6	0.1
WP_089612430.1	Sulfite reductase, dissimilatory-type subunit beta	DsrB	Dissimilatory sulfite reduction	24.5	0.4	0.0	12.7	0.9	0.1	32.5	2.8	0.1
WP_089612427.1	Sulfite reductase, dissimilatory-type subunit gamma	DsrC	Dissimilatory sulfite reduction	7.0	2.1	0.3	5.3	0.5	0.1	51.7	10.3	0.2
WP_242965458.1	FAD-dependent oxidoreductase	GltD/StnC	Electron bifurcation	62.9	3.0	0.0	39.0	0.0	0.0	49.9	0.7	0.0
WP_089609814.1	NADP-reducing hydrogenase subunit HndA	HndA	Electron bifurcation	9.3	3.2	0.3	7.3	1.2	0.2	37.3	3.0	0.1
WP_089609810.1	NADP-reducing hydrogenase subunit HndA	HndA/StnA	Electron bifurcation	6.3	0.2	0.0	3.3	0.5	0.1	33.3	1.0	0.0
WP_157677371.1	NADP-reducing hydrogenase subunit HndB	HndB	Electron bifurcation	7.6	3.1	0.4	7.0	1.4	0.2	44.9	7.0	0.2
WP_242965405.1	Ferredoxin--NADP reductase	HndC	Electron bifurcation	80.8	6.8	0.1	56.7	2.9	0.1	45.4	7.0	0.2
WP_242965405.1	Ion-translocating oxidoreductase complex subunit C	HndC/StnB	Electron bifurcation	29.1	1.5	0.1	17.3	1.2	0.1	32.3	0.7	0.0
WP_089609815.1	NADP-reducing hydrogenase subunit HndD	HndD	Electron bifurcation	61.8	10.6	0.2	46.0	6.5	0.1	56.2	4.7	0.1
WP_089611730.1	Aldehyde-alcohol dehydrogenase		Fermentation producing ethanol	39.6	4.2	0.1	32.0	0.0	0.0	63.0	6.9	0.1
WP_089612456.1	Valine--tRNA ligase		General cellular functions	30.3	2.1	0.1	18.0	2.2	0.1	24.6	1.4	0.1
WP_089608704.1	Trigger factor		General cellular functions	35.5	0.9	0.0	19.0	0.8	0.0	45.1	0.6	0.0
WP_089608919.1	Methionine--tRNA ligase		General cellular functions	29.5	3.5	0.1	14.7	1.2	0.1	28.1	2.6	0.1
WP_089612510.1	Lysine--tRNA ligase		General cellular functions	34.7	2.1	0.1	18.7	1.2	0.1	47.1	2.8	0.1
WP_089609461.1	DNA-directed RNA polymerase subunit beta		General cellular functions	53.6	6.1	0.1	27.0	3.6	0.1	29.2	4.3	0.1
WP_198306698.1	DNA-directed RNA polymerase subunit beta'		General cellular functions	50.2	1.9	0.0	23.7	0.5	0.0	25.8	0.0	0.0
WP_089609466.1	Elongation factor G		General cellular functions	39.0	1.3	0.0	25.0	1.4	0.1	39.5	2.5	0.1
WP_089609453.1	Elongation factor Tu		General cellular functions	64.1	3.9	0.1	74.3	6.6	0.1	80.8	4.6	0.1
WP_089609560.1	Glycine--tRNA ligase beta subunit		General cellular functions	42.5	4.5	0.1	23.0	1.6	0.1	37.5	4.6	0.1
WP_089609646.1	Glutamate--tRNA ligase		General cellular functions	29.6	4.2	0.1	16.3	2.5	0.2	34.1	7.6	0.2
WP_089610227.1	30S ribosomal protein S2		General cellular functions	30.6	2.2	0.1	21.0	1.4	0.1	69.2	5.0	0.1
WP_089611234.1	Translation initiation factor IF-2		General cellular functions	45.1	4.6	0.1	28.7	2.9	0.1	33.8	3.4	0.1
WP_089611512.1	Phenylalanine--tRNA ligase beta subunit		General cellular functions	40.7	2.1	0.1	21.3	0.5	0.0	26.4	1.3	0.0
WP_089611525.1	Threonine--tRNA ligase 2		General cellular functions	41.6	5.6	0.1	23.0	2.2	0.1	36.1	3.1	0.1
WP_089611245.1	Polyribonucleotide nucleotidyltransferase		General cellular functions	41.2	1.3	0.0	22.3	0.9	0.0	32.7	2.6	0.1
WP_089610191.1	Glycine cleavage system H protein	GcvH	Glycine cleavage system	5.8	0.7	0.1	3.0	0.0	0.0	16.1	0.0	0.0
WP_089612298.1	Glycine cleavage system H protein	GcvH	Glycine cleavage system	6.4	0.5	0.1	6.0	1.6	0.3	24.9	1.9	0.1
WP_089612299.1	Aminomethyltransferase	GcvT	Glycine cleavage system	36.3	1.8	0.1	24.3	1.7	0.1	52.5	3.7	0.1
WP_089608827.1	Glyceraldehyde-3-phosphate dehydrogenase	GapA	Glycolysis	26.6	1.7	0.1	17.0	1.6	0.1	42.5	4.4	0.1
WP_089609613.1	2,3-bisphosphoglycerate-independent phosphoglycerate mutase	GpmI	Glycolysis	24.8	6.0	0.2	12.7	1.9	0.1	42.5	10.6	0.2
WP_089609361.1	Phosphoheptose isomerase	Pgi	Glycolysis	14.2	1.2	0.1	9.0	0.8	0.1	31.1	5.1	0.2
WP_089612675.1	Pyruvate kinase	Pyk	Glycolysis	2.2	0.2	0.1	1.0	0.0	0.0	2.9	0.0	0.0
WP_089608823.1	Enolase	Eno	Glycolysis/Gluconeogenesis	34.5	3.2	0.1	23.7	1.7	0.1	51.7	5.3	0.1
WP_089611592.1	Fructose-bisphosphate aldolase	Fba	Glycolysis/Gluconeogenesis	36.9	2.1	0.1	21.3	0.9	0.0	46.6	3.7	0.1
WP_089609329.1	Phosphoglucomutase	Pgm	Glycolysis/Gluconeogenesis	19.0	5.1	0.3	11.3	3.4	0.3	26.9	5.3	0.2
WP_089609562.1	Pyruvate, phosphate dikinase	ppdK	Glycolysis/Pyruvate metabolism - forms PEP	38.9	5.7	0.1	21.7	1.2	0.1	27.2	3.5	0.1
WP_089609061.1	Uroporphyrinogen decarboxylase		heme biosynthesis	31.2	3.0	0.1	34.7	4.8	0.1	57.4	6.7	0.1
WP_089609062.1	Uroporphyrinogen-III decarboxylase		heme biosynthesis	63.2	2.7	0.0	88.7	1.9	0.0	74.7	2.1	0.0
WP_089609129.1	1-deoxy-D-xylulose-5-phosphate synthase	DXPS	Isoprenoid synthesis	34.0	4.0	0.1	21.7	2.1	0.1	30.4	2.6	0.1
WP_242965475.1	4-hydroxy-3-methylbut-2-enyl diphosphate reductase		Isoprenoid synthesis	51.1	0.5	0.0	35.7	0.5	0.0	39.9	2.2	0.1
WP_089610162.1	NAD(P) transhydrogenase subunit alpha part 1		Metabolism - interconversion NADH-NADPH	29.5	3.0	0.1	17.7	1.9	0.1	50.5	1.5	0.0
WP_089609848.1	2,3-dimethylmalate dehydratase large subunit	DmdA	Nicotinate degradation	37.3	0.9	0.0	26.3	2.1	0.1	54.2	3.0	0.1
WP_242965416.1	Catalase		Oxidative stress response	30.5	4.0	0.1	20.7	2.5	0.1	40.4	5.2	0.1
WP_089612325.1	Rubrerythrin		Oxidative stress response	32.4	1.1	0.0	38.3	4.1	0.1	80.3	3.3	0.0
WP_089609284.1	Putative sugar phosphate isomerase YwlF	RpiB	Pentose phosphate pathway	10.0	0.3	0.0	5.0	0.0	0.0	37.9	0.0	0.0
WP_089609274.1	Transaldolase	Tal	Pentose phosphate pathway	7.6	1.5	0.2	5.0	0.0	0.0	29.0	2.2	0.1
WP_089609338.1	Apulose-4-phosphate transketolase subunit A	TktA	Pentose phosphate pathway	15.3	0.8	0.1	11.3	0.5	0.0	38.6	2.0	0.1
WP_089609339.1	Apulose-4-phosphate transketolase subunit B	TktB	Pentose phosphate pathway	5.5	1.6	0.3	3.7	1.2	0.3	16.8	5.1	0.3
WP_089611302.1	Inosine-5'-monophosphate dehydrogenase	guaA (?)	Purine biosynthesis	36.9	1.8	0.0	22.7	1.7	0.1	49.7	3.0	0.1
WP_089609737.1	GMP synthase [glutamine-hydrolyzing]		Purine biosynthesis	33.1	2.0	0.1	18.3	1.2	0.1	42.8	4.3	0.1
WP_089612687.1	Phosphoribosylformylglycinamidine synthase		Purine biosynthesis	31.2	3.0	0.1	17.7	1.7	0.1	19.7	1.3	0.1
WP_089611765.1	Acetate kinase	AckA	Pyruvate metabolism	29.1	5.6	0.2	32.0	3.7	0.1	43.2	4.6	0.1
WP_089608750.1	Phosphate propanoyltransferase	PduL	Pyruvate metabolism	27.6	0.1	0.0	20.0	1.4	0.1	75.5	3.7	0.0
WP_089608943.1	Pyruvate:ferredoxin oxidoreductase	Por	Pyruvate metabolism	113.9	8.7	0.1	94.7	7.0	0.1	51.7	2.0	0.0
WP_198306742.1	Pyruvate carboxylase	Pyc	Pyruvate metabolism	87.8	3.6	0.0	59.3	0.9	0.0	49.4	2.7	0.1
WP_089609648.1	2-succinylbenzoate--CoA ligase	MenE	Quinone biosynthesis - menaquinone	32.4	5.9	0.2	20.0	3.6	0.2	28.5	4.9	0.2
WP_242965500.1	putative glycine dehydrogenase (decarboxylating) subunit 1	GcvPA	Reductive glycine pathway	26.6	5.4	0.2	20.7	5.3	0.3	28.9	6.3	0.2
WP_089612296.1	putative glycine dehydrogenase (decarboxylating) subunit 2	GcvPB	Reductive glycine pathway	51.9	4.0	0.1	44.0	3.6	0.1	65.3	5.6	0.1
WP_089609580.1	Glycine/sarcosine/betaine reductase complex component A	GrdA	Reductive glycine pathway	6.0	0.0	0.0	3.3	0.5	0.1	51.2	0.0	0.0
WP_089609585.1	Glycine reductase complex component B subunit gamma	GrdB	Reductive glycine pathway	49.8	3.4	0.1	48.0	3.6	0.1	75.6	3.3	0.0
WP_089609585.1	Glycine reductase complex component B subunit gamma	GrdB	Reductive glycine pathway	6.7	1.1	0.2	4.0	0.8	0.2	62.3	9.0	0.1
WP_089612053.1	Glycine reductase complex component B subunit gamma	GrdB	Reductive glycine pathway	6.7	1.2	0.2	3.3	0.5	0.1	68.3	0.8	0.0
WP_089612053.1	Glycine reductase complex component B subunit gamma	GrdB	Reductive glycine pathway	33.8	2.4	0.1	21.7	1.2	0.1	69.4	1.0	0.0
WP_089609581.1	Glycine/sarcosine/betaine reductase complex component C subunit beta	GrdC	Reductive glycine pathway	48.6	6.3	0.1	33.7	2.5	0.1	64.3	5.5	0.1
WP_089608591.1	Glycine/sarcosine/betaine reductase complex component C subunit beta	GrdD	Reductive glycine pathway	3.3	1.2	0.3	1.7	0.5	0.3	6.7	2.2	0.3
WP_089609582.1	Glycine/sarcosine/betaine reductase complex component C subunit alpha	GrdD	Reductive glycine pathway	20.5	1.1	0.1	14.0	0.8	0.1	36.9	3.0	0.1
WP_089609584.1	Glycine reductase complex component B subunits alpha and beta	GrdE	Reductive glycine pathway	39.7	3.9	0.1	30.3	2.6	0.1	50.3	3.0	0.1
WP_089612054.1	Glycine reductase complex component B subunits alpha and beta	GrdE	Reductive glycine pathway	21.1	2.7	0.1	15.3	2.1	0.1	41.3	5.9	0.1
WP_089609285.1	Serine hydroxymethyltransferase	GlyA	Serine pathway	34.7	1.4	0.0	22.3	1.7	0.1	45.4	3.4	0.1
WP_198306669.1	4Fe-4S dicluster domain-containing protein	FloxA	Sulfur redox reaction	28.1	1.8	0.1	19.0	1.4	0.1	42.3	4.3	0.1
WP_089609932.1	FAD/NAD(P)-binding protein	FloxB	Sulfur redox reaction	21.0	2.9	0.1	14.0	1.6	0.1	46.7	2.5	0.1
WP_089609931.1	4Fe-4S binding protein	FloxC	Sulfur redox reaction	37.4	2.5	0.1	22.3	2.4	0.1	60.1	4.6	0.1
WP_157677380.1	Hydrogenase iron-sulfur subunit	FloxD	Sulfur redox reaction	3.1	0.3	0.1	2.7	0.5	0.2	32.4	5.5	0.2
WP_089609930.1	CoB--CoM heterodisulfide reductase iron-sulfur subunit A family protein	HdrA	Sulfur redox reaction	15.2	1.1	0.1	9.0	0.0	0.0	46.6	0.7	0.0
WP_089609930.1	CoB--CoM heterodisulfide reductase iron-sulfur subunit A family protein	HdrA	Sulfur redox reaction	33.0	0.8	0.0	19.3	0.5	0.0	45.9	1.4	0.0
WP_089609929.1	CoB--CoM heterodisulfide reductase iron-sulfur subunit B family protein	HdrB	Sulfur redox reaction	17.9	1.7	0.1	11.7	1.2	0.1	42.0	2.3	0.1
WP_198306668.1	4Fe-4S dicluster domain-containing protein	HdrC	Sulfur redox reaction	11.0	0.7	0.1	6.7	0.5	0.1	35.8	0.0	0.0
WP_089611538.1	Aconitate hydratase	AcnA	TCA cycle	32.2	7.8	0.2	18.3	2.9	0.2	32.7	5.0	0.2
WP_089609882.1	Fumarate hydratase class I, anaerobic	FumA	TCA cycle	3.3	1.2	0.3	1.7	0.5	0.3	14.4	4.1	0.3
WP_089611970.1	Citrate (Re)-synthase	GltA	TCA cycle	29.4	6.0	0.2	16.7	1.2	0.1	40.5	10.9	0.3
WP_089609924.1	Isocitrate dehydrogenase [NADP]	Icd	TCA cycle	19.7	2.2	0.1	10.0	0.8	0.1	28.6	0.2	0.0
WP_089609958.1	2-oxoglutarate oxidoreductase subunit KorA	KorA	TCA cycle	20.7	3.7	0.2	11.3	1.7	0.1	24.6	2.7	0.1
WP_089609883.1	NAD-dependent malic enzyme	MaeA	TCA cycle	14.8	4.7	0.3	8.3	1.9	0.2	29.1	4.9	0.2
WP_242965367.1	Leucine-, isoleucine-, valine-, threonine-, and alanine-binding protein		Transporter - amino acids	33.6	0.6	0.0	29.0	4.3	0.1	47.4	4.0	0.1
WP_089612406.1	Protein translocase subunit SecDF	SecDF	Transporter - protein export	36.5	2.9	0.1	25.7	2.1	0.1	43.0	1.1	0.0
WP_089609335.1	Protein translocase subunit SecA		Transporter - protein export	44.2	7.8	0.2	22.3	3.4	0.2	34.3	5.7	0.2
WP_198306667.1	DUF4127 family protein		Unknown	34.4	3.5	0.1	18.0	2.2	0.1	27.2	1.6	0.1
WP_089612401.1	DUF4430 domain-containing protein		Unknown	45.0	5.0	0.1	26.7	2.1	0.1	54.2	2.5	0.0
WP_089609411.1	carboxypeptidase-like regulatory domain-containing protein		Unknown	34.3	2.5	0.1	28.7	2.5	0.1	32.2	1.7	0.1
WP_089612339.1	Threonine-phosphate decarboxylase		Vitamin Biosynthesis - B12	30.5	1.1	0.0	18.3	0.9	0.1	37.4	2.0	0.1
WP_089612341.1	Cobyric acid synthase		Vitamin Biosynthesis - B12	35.8	2.6	0.1	28.0	1.4	0.1	44.6	3.3	0.1
WP_242965292.1	pyridoxamine 5'-phosphate oxidase family protein	PdxH	Vitamin Biosynthesis - B6	36.6	4.4	0.1	71.7	7.3	0.1	88.7	5.8	0.1
WP_089611869.1	Carbon monoxide dehydrogenase/acetyl-CoA synthase subunit beta	AcsB	Wood-Ljungdahl Pathway	41.1	3.4	0.1	27.3	0.9	0.0	39.2	3.7	0.1
WP_089612391.1	5-methyltetrahydrofolate:corrinoid/iron-sulfur protein co-methyltransferase	AcsE	Wood-Ljungdahl Pathway	23.5	1.9	0.1	23.0	2.8	0.1	42.5	6.5	0.2
WP_089611865.1	Corrinoid/iron-sulfur protein large subunit	CFeSP	Wood-Ljungdahl Pathway	39.7	7.7	0.2	24.3	2.6	0.1	50.9	4.5	0.1
WP_089611867.1	Carbon monoxide dehydrogenase/acetyl-CoA synthase subunit alpha	CooS/AcsA	Wood-Ljungdahl Pathway	41.9	1.2	0.0	24.0	0.8	0.0	33.7	0.5	0.0
WP_242965458.1	Formate dehydrogenase subunit alpha	FdhF	Wood-Ljungdahl Pathway	24.0	2.6	0.1	15.3	0.5	0.0	36.4	5.6	0.2
WP_089609513.1	Formate--tetrahydrofolate ligase	Fhs	Wood-Ljungdahl Pathway	74.6	6.4	0.1	72.0	4.1	0.1	64.3	2.1	0.0
WP_089612071.1	Methenyltetrahydrofolate cyclohydrolase	FolD	Wood-Ljungdahl Pathway	15.9	1.5	0.1	13.3	1.2	0.1	49.5	2.0	0.0
WP_089608526.1	Methylenetetrahydrofolate reductase	MetF	Wood-Ljungdahl Pathway	15.7	1.0	0.1	10.3	0.5	0.0	40.4	4.2	0.1
WP_089611856.1	Methylenetetrahydrofolate reductase	MetF	Wood-Ljungdahl Pathway	39.9	3.9	0.1	26.0	0.8	0.0	51.0	4.9	0.1
WP_089611285.1	Ribonuclease Y	CobQ		36.3	0.7	0.0	21.0	1.6	0.1	42.6	6.5	0.2

WP_089608521.1	secondary thiamine-phosphate synthase enzyme YjbQ	2.0	0.0	0.0	1.0	0.0	0.0	8.5	0.0	0.0
WP_089608530.1	Ig-like domain-containing protein	25.4	2.8	0.1	11.7	2.1	0.2	10.4	1.5	0.1
WP_089608538.1	Ethanolamine utilization protein EutL	1.8	0.2	0.1	1.0	0.0	0.0	7.3	0.0	0.0
WP_198306594.1	aryl-sulfate sulfotransferase	3.0	1.2	0.4	2.0	0.8	0.4	5.5	2.2	0.4
WP_089608553.1	Ferredoxin--NADP reductase	11.2	3.1	0.3	7.3	1.7	0.2	23.2	7.3	0.3
WP_089608554.1	Thioredoxin 1	6.8	0.0	0.0	5.0	0.0	0.0	46.3	0.0	0.0
WP_089608561.1	DUF4163 domain-containing protein	9.8	1.2	0.1	5.7	1.2	0.2	19.6	4.1	0.2
WP_198306679.1	flotillin-like protein FloA	27.0	2.0	0.1	16.3	0.9	0.1	53.8	3.0	0.1
WP_089608564.1	nodulation protein NfeD	6.9	1.1	0.2	3.7	0.5	0.1	10.0	1.4	0.1
WP_089608565.1	30S ribosomal protein S21	5.5	1.2	0.2	3.7	1.2	0.3	44.3	6.5	0.1
WP_089612441.1	Purine nucleoside phosphoramidase	7.7	1.6	0.2	4.3	0.9	0.2	39.5	0.0	0.0
WP_089608566.1	Ribosomal RNA small subunit methyltransferase E	4.4	1.4	0.3	2.7	0.5	0.2	18.4	4.3	0.2
WP_089612443.1	Ribosomal protein L11 methyltransferase	9.0	1.6	0.2	6.0	0.0	0.0	18.5	3.3	0.2
WP_089608567.1	Chaperone protein DnaJ	25.4	1.3	0.1	14.7	1.2	0.1	44.4	5.0	0.1
WP_089608569.1	Protein GrpE	15.0	2.5	0.2	10.0	1.6	0.2	48.7	2.2	0.0
WP_089608570.1	60 kDa chaperonin	6.0	1.7	0.3	3.7	0.9	0.3	10.2	3.4	0.3
WP_089612444.1	Elongation factor 4	9.1	3.9	0.4	5.0	1.4	0.3	13.6	5.1	0.4
WP_089608578.1	30S ribosomal protein S20	2.6	1.2	0.5	1.7	0.5	0.3	25.7	0.5	0.0
WP_089612456.1	Leucine--tRNA ligase	8.1	2.1	0.3	4.0	0.8	0.2	15.7	2.3	0.1
WP_089610002.1	Leucine--tRNA ligase subunit alpha	15.2	3.1	0.2	9.3	2.5	0.3	21.6	6.5	0.3
WP_089608583.1	hemolysin family protein	4.8	2.2	0.5	3.0	0.8	0.3	7.8	4.4	0.6
WP_157677240.1	Sodium/pantothenate symporter	2.6	1.2	0.5	1.3	0.5	0.4	3.6	1.6	0.5
WP_089608593.1	ATP-binding protein	7.9	2.7	0.3	4.7	1.2	0.3	9.5	2.6	0.3
WP_089608594.1	hypothetical protein	7.7	1.7	0.2	4.0	0.8	0.2	15.6	4.3	0.3
WP_089608595.1	Phage shock protein A	39.5	2.1	0.1	28.7	3.1	0.1	73.6	4.5	0.1
WP_089608599.1	cupin domain-containing protein	2.8	0.1	0.0	4.0	0.0	0.0	19.7	0.0	0.0
WP_089608600.1	hypothetical protein	2.0	0.0	0.0	1.0	0.0	0.0	16.1	0.0	0.0
WP_089608606.1	Release factor glutamine methyltransferase	4.1	0.2	0.0	2.0	0.0	0.0	6.2	0.3	0.0
WP_089608619.1	Sensor protein KdpD	3.1	1.3	0.4	2.3	0.9	0.4	3.5	1.7	0.5
WP_089608622.1	Potassium-transporting ATPase potassium-binding subunit	5.6	2.1	0.4	3.0	0.8	0.3	5.1	1.6	0.3
WP_089608623.1	Potassium-transporting ATPase ATP-binding subunit	19.3	0.7	0.0	10.3	0.9	0.1	21.7	1.7	0.1
WP_089608624.1	hypothetical protein	4.1	0.1	0.0	2.0	0.0	0.0	17.2	0.0	0.0
WP_089608625.1	Potassium-transporting ATPase KdpC subunit	4.6	1.1	0.2	2.7	0.9	0.4	23.5	3.8	0.2
WP_198306599.1	Ribonuclease Y	2.0	0.0	0.0	1.0	0.0	0.0	7.5	0.0	0.0
WP_089608681.1	RNA-binding protein	3.2	0.3	0.1	2.3	0.5	0.2	27.1	4.4	0.2
WP_089608684.1	50S ribosomal protein L27	7.5	0.3	0.0	4.3	0.5	0.1	50.0	0.0	0.0
WP_089608685.1	50S ribosomal protein L21	15.3	1.2	0.1	9.0	0.8	0.1	53.4	0.0	0.0
WP_089608686.1	Ribonuclease G	4.1	1.5	0.4	2.3	0.9	0.4	5.2	2.0	0.4
WP_089608693.1	Septum site-determining protein MinD	14.3	0.3	0.0	8.7	0.5	0.1	36.4	1.6	0.0
WP_242965345.1	Peptidoglycan D,D-transpeptidase MrdA	9.3	2.3	0.2	5.3	1.2	0.2	10.9	3.5	0.3
WP_089608697.1	Cell shape-determining protein MreB	13.9	3.0	0.2	7.0	0.8	0.1	25.6	4.8	0.2
WP_089608699.1	Redox-sensing transcriptional repressor Rex	6.0	2.5	0.4	3.3	1.2	0.4	16.1	4.5	0.3
WP_089608700.1	Folylpolyglutamate synthase	5.9	1.1	0.2	3.3	0.5	0.1	10.2	1.2	0.1
WP_089612458.1	Lon protease 1	39.6	3.0	0.1	23.0	1.4	0.1	32.9	1.4	0.0
WP_089608702.1	ATP-dependent Clp protease ATP-binding subunit ClpX	23.5	1.8	0.1	13.3	0.9	0.1	32.0	2.4	0.1
WP_089608703.1	ATP-dependent Clp protease proteolytic subunit	10.4	1.1	0.1	7.3	0.5	0.1	22.8	1.4	0.1
WP_089608707.1	2-methoxy-6-polyprenyl-1,4-benzoquinol methylase, mitochondrial	5.9	0.5	0.1	3.7	0.5	0.1	18.0	5.1	0.3
WP_089608711.1	Bifunctional uridylyltransferase/uridylyl-removing enzyme	8.8	2.3	0.3	6.0	1.6	0.3	40.1	12.5	0.3
WP_089608712.1	Phenylacetate-coenzyme A ligase	22.1	1.7	0.1	13.7	1.2	0.1	28.9	2.5	0.1
WP_089608716.1	FMN-binding protein	29.2	2.3	0.1	27.3	5.0	0.2	49.0	0.0	0.0
WP_089608717.1	Formate dehydrogenase, cytochrome b556(fdo) subunit	2.1	0.1	0.0	1.3	0.5	0.4	5.4	1.6	0.3
WP_242965346.1	Oligopeptide-binding protein OppA	7.5	2.2	0.3	4.3	0.9	0.2	11.0	3.2	0.3
WP_089608721.1	Methionine synthase	2.0	0.0	0.0	2.0	0.0	0.0	11.7	0.0	0.0
WP_089608724.1	Zinc-binding GTPase YeiR	4.1	2.0	0.5	2.3	1.2	0.5	11.4	7.3	0.6
WP_089608726.1	Hypoxanthine-guanine phosphoribosyltransferase	5.5	1.5	0.3	3.0	0.8	0.3	16.7	4.6	0.3
WP_089608729.1	10 kDa chaperonin	14.0	0.9	0.1	10.7	0.5	0.0	76.6	3.9	0.1
WP_157677251.1	hypothetical protein	2.9	0.9	0.3	1.7	0.5	0.3	39.3	12.3	0.3
WP_089608735.1	Imidazolonepropionase	8.1	2.0	0.3	4.7	0.9	0.2	15.7	3.3	0.2
WP_198306687.1	Alanine racemase 1	10.6	3.2	0.3	6.3	1.2	0.2	23.7	5.1	0.2
WP_089608737.1	Bifunctional NAD(P)H-hydrate repair enzyme Nnr	13.5	2.5	0.2	8.3	2.1	0.2	15.3	3.4	0.2
WP_089608746.1	Protoporphyrinogen oxidase	2.0	0.1	0.0	1.0	0.0	0.0	1.9	0.0	0.0
WP_089608748.1	Uroporphyrinogen decarboxylase	2.0	0.0	0.0	1.0	0.0	0.0	4.9	1.0	0.2
WP_089608757.1	Pyruvate synthase subunit PorC	6.9	0.3	0.0	3.7	0.5	0.1	25.9	1.8	0.1
WP_089608758.1	Pyruvate synthase subunit PorD	5.7	0.7	0.1	3.7	0.5	0.1	46.7	5.5	0.1
WP_089608759.1	Pyruvate synthase subunit PorA	15.6	1.7	0.1	8.3	0.5	0.1	36.8	3.0	0.1
WP_089608760.1	Oxalate oxidoreductase subunit beta	16.1	2.4	0.1	9.0	0.8	0.1	39.7	6.1	0.2
WP_089608761.1	DUF86 domain-containing protein	4.7	1.3	0.3	2.3	0.5	0.2	23.6	4.9	0.2
WP_089608768.1	Chromosome partition protein Smc	7.4	1.8	0.2	4.3	1.2	0.3	4.1	1.1	0.3
WP_089608769.1	TIGR02678 family protein	2.0	0.1	0.0	1.0	0.0	0.0	3.8	0.0	0.0
WP_089608774.1	DUF2680 domain-containing protein	11.5	2.1	0.2	7.7	1.2	0.2	54.5	6.2	0.1
WP_089608791.1	Pyridinium-3,5-bisthiocarboxylic acid mononucleotide nickel insertion protein	5.0	0.9	0.2	2.7	0.5	0.2	9.2	1.4	0.2
WP_089608800.1	Macrolide export ATP-binding/permease protein MacB	6.4	1.4	0.2	3.3	0.5	0.1	19.2	3.5	0.2
WP_089608817.1	Ribonuclease R	24.4	1.3	0.1	12.7	1.2	0.1	20.1	2.7	0.1
WP_089608821.1	Putative K(+)-stimulated pyrophosphate-energized sodium pump	21.0	0.9	0.0	12.3	1.2	0.1	16.1	1.0	0.1
WP_089608822.1	putative protein-export membrane protein SecG	2.0	0.0	0.0	1.7	0.5	0.3	24.0	0.0	0.0
WP_242965438.1	2,3-bisphosphoglycerate-independent phosphoglycerate mutase	8.2	1.6	0.2	5.0	0.8	0.2	15.7	2.3	0.1
WP_089608825.1	Triosephosphate isomerase	7.8	1.2	0.2	4.7	0.9	0.2	21.8	2.2	0.1
WP_089608826.1	Phosphoglycerate kinase	18.1	1.2	0.1	9.7	1.2	0.1	37.0	3.1	0.1
WP_089608830.1	RNA polymerase sigma-54 factor	3.0	1.0	0.3	1.7	0.5	0.3	4.6	1.3	0.3
WP_089608836.1	Reverse rubrerythrin-2	17.9	3.0	0.2	14.7	2.4	0.2	76.6	4.4	0.1
WP_089608852.1	Large-conductance mechanosensitive channel	6.1	0.9	0.1	4.3	0.5	0.1	17.4	1.0	0.1
WP_089608854.1	Aspartate aminotransferase	26.2	3.4	0.1	13.3	2.1	0.2	40.1	6.4	0.2
WP_089608855.1	Phosphoserine aminotransferase	28.2	0.9	0.0	20.3	1.2	0.1	45.5	2.9	0.1
WP_089608858.1	2-hydroxyisocaproyl-CoA dehydratase activator	4.1	1.9	0.5	2.7	0.5	0.2	16.0	3.9	0.2
WP_089608859.1	Cysteine desulfurase SufS	8.6	4.1	0.5	5.0	2.4	0.5	15.7	6.9	0.4
WP_089608860.1	Cytochrome c nitrite reductase subunit NrfA	6.4	1.6	0.2	4.3	0.5	0.1	11.5	1.0	0.1
WP_089608863.1	S-layer homology domain-containing protein	41.6	2.1	0.0	28.0	2.2	0.1	43.5	1.2	0.0
WP_242965350.1	putative protein/GBAA_0901/BAS0853	16.2	0.8	0.0	10.0	0.8	0.1	30.2	1.9	0.1
WP_089608869.1	K(+)-stimulated pyrophosphate-energized proton pump	24.9	2.5	0.1	17.0	0.8	0.0	17.6	1.7	0.1
WP_089608870.1	DUF169 domain-containing protein	7.8	0.9	0.1	4.7	0.5	0.1	28.3	2.4	0.1
WP_089608873.1	Molybdate-binding protein ModA	20.8	1.2	0.1	17.7	0.9	0.1	50.9	0.0	0.0
WP_089608875.1	1-pyrroline-5-carboxylate dehydrogenase 1	19.6	1.7	0.1	13.0	1.6	0.1	26.1	1.9	0.1
WP_089608877.1	Hydroxyacylglutathione hydrolase	2.6	1.1	0.4	1.3	0.5	0.4	7.1	2.0	0.3
WP_089608884.1	Curved DNA-binding protein	8.2	2.9	0.4	4.3	0.9	0.2	20.5	4.8	0.2
WP_089608886.1	hydantoinase/oxoprolinase family protein	20.2	2.0	0.1	10.3	0.5	0.0	16.8	2.0	0.1
WP_089608894.1	N-acetylglucosaminyldiphosphoundecaprenol N-acetyl-beta-D-mannosaminyltransferase	2.4	0.6	0.2	1.3	0.5	0.4	5.8	1.6	0.3
WP_089608908.1	polysaccharide lyase	29.4	4.6	0.2	21.7	1.2	0.1	37.0	3.6	0.1
WP_089611195.1	MULTISPECIES: DUF5320 domain-containing protein	3.5	0.4	0.1	2.7	0.5	0.2	26.1	0.0	0.0
WP_089611882.1	MULTISPECIES: NifB/NifX family molybdenum-iron cluster-binding protein	11.6	1.6	0.1	7.7	0.5	0.1	68.9	3.5	0.1
WP_089609235.1	Ribonuclease BN	5.0	2.3	0.5	2.7	0.9	0.4	15.8	7.1	0.4
WP_089611870.1	Iron-sulfur cluster carrier protein	19.9	1.3	0.1	12.3	2.1	0.2	39.8	1.3	0.0
WP_242965352.1	putative protein	2.0	0.0	0.0	1.0	0.0	0.0	6.6	0.0	0.0
WP_242965353.1	D-aminoacyl-tRNA deacylase	6.0	2.0	0.3	3.3	0.5	0.1	19.9	2.7	0.1
WP_089608920.1	AbrB/MazE/SpoVT family DNA-binding domain-containing protein	7.0	1.8	0.3	4.7	1.2	0.3	57.0	14.8	0.3
WP_089608924.1	stage 0 sporulation family protein	10.0	2.3	0.2	5.3	1.2	0.2	23.2	2.6	0.1
WP_157677287.1	Sodium/pantothenate symporter	2.4	0.8	0.3	2.0	0.0	0.0	2.8	0.0	0.0
WP_089608940.1	Anaerobic nitric oxide reductase transcription regulator NorR	4.4	0.3	0.1	2.3	0.5	0.2	5.7	0.9	0.2
WP_089608941.1	Putative niacin/nicotinamide transporter NaiP	3.0	1.0	0.3	2.0	0.8	0.4	5.1	1.1	0.2
WP_198306613.1	putative xanthine dehydrogenase subunit D	9.2	0.6	0.1	5.0	0.0	0.0	9.0	0.4	0.0
WP_089608946.1	Nucleoid-associated protein	4.4	0.1	0.0	8.0	1.4	0.2	30.5	11.8	0.4
WP_157677289.1	DNA polymerase III subunit gamma/tau	2.1	0.8	0.4	1.7	0.9	0.6	3.0	1.7	0.6
WP_089608956.1	flavodoxin family protein	7.2	0.7	0.1	4.0	0.0	0.0	23.8	0.9	0.0
WP_157677293.1	ferritin family protein	15.7	0.4	0.0	8.7	0.5	0.1	52.1	2.1	0.0
WP_089610940.1	Acid shock protein	4.3	0.7	0.2	3.7	0.5	0.1	27.5	5.4	0.2
WP_089608992.1	TIR domain-containing protein	7.0	1.7	0.2	4.3	0.5	0.1	30.9	7.4	0.2
WP_089608993.1	TIR domain-containing protein	4.4	0.6	0.1	2.3	0.5	0.2	19.5	1.9	0.1
WP_089608998.1	site-specific DNA-methyltransferase	1.4	0.1	0.1	1.0	0.0	0.0	1.2	0.0	0.0
WP_089609004.1	helix-turn-helix domain-containing protein	1.8	0.3	0.2	1.0	0.0	0.0	9.0	0.0	0.0
WP_089609025.1	ATP-binding protein	5.2	1.1	0.2	3.3	0.5	0.1	8.6	1.3	0.2
WP_089609054.1	pyridoxamine 5'-phosphate oxidase family protein	16.4	1.9	0.1	28.0	1.6	0.1	79.4	1.4	0.0
WP_089609055.1	DUF1638 domain-containing protein	13.4	2.6	0.2	8.0	0.8	0.1	31.5	4.7	0.1
WP_089609056.1	Na(+)/H(+)-K(+) antiporter GerN	3.2	1.0	0.3	2.3	0.5	0.2	6.0	1.4	0.2
WP_089609057.1	Uroporphyrinogen decarboxylase	17.3	0.1	0.0	12.7	0.5	0.0	40.5	3.3	0.1
WP_089609058.1	Na(+)-translocating NADH-quinone reductase subunit F	41.7	4.1	0.1	25.0	1.6	0.1	38.0	4.3	0.1
WP_089609059.1	DUF1638 domain-containing protein	4.0	1.6	0.4	3.0	0.8	0.3	19.0	4.6	0.2
WP_089608722.1	putative response regulatory protein	8.6	0.8	0.1	5.7	1.2	0.2	22.0	4.6	0.2
WP_242965363.1	PocR ligand-binding domain-containing protein	7.4	2.4	0.3	4.0	0.8	0.2	12.4	2.2	0.2
WP_157677299.1	IS66 family transposase ISSwo2	3.9	2.2	0.6	2.3	1.2	0.5	5.9	3.3	0.6
WP_089609071.1	hypothetical protein	1.7	0.4	0.2	1.0	0.0	0.0	8.1	0.0	0.0
WP_089609086.1	N-6 DNA methylase	3.2	0.6	0.2	2.3	0.5	0.2	3.2	0.8	0.3
WP_089609087.1	hypothetical protein	5.4	1.5	0.3	3.7	0.5	0.1	4.0	0.7	0.2
WP_089609088.1	hypothetical protein	2.0	0.0	0.0	1.0	0.0	0.0	7.3	0.0	0.0
WP_089609094.1	Serine--tRNA ligase	28.1	2.5	0.1	15.3	0.5	0.0	41.4	2.1	0.1
WP_242965445.1	DNA gyrase subunit A	27.5	2.2	0.1	15.7	0.9	0.1	22.3	1.7	0.1
WP_089609097.1	DNA gyrase subunit B	3.2	1.2	0.4	1.7	0.5	0.3	4.7	1.6	0.3
WP_157677307.1	DUF370 domain-containing protein	2.7	1.2	0.4	1.3	0.5	0.4	22.9	10.2	0.4
WP_089609100.1	Beta sliding clamp	8.3	3.5	0.4	4.7	1.9	0.4	20.4	8.6	0.4
WP_089609101.1	Chromosomal replication initiator protein DnaA	7.7	0.4	0.1	4.0	0.0	0.0	13.6	0.0	0.0
WP_242965365.1	Membrane protein insertase YidC 2	4.5	1.9	0.4	3.0	0.8	0.3	10.5	2.1	0.2
WP_089609104.1	Transcription termination/antitermination protein NusA	18.1	3.0	0.2	10.3	0.9	0.1	38.4	4.7	0.1
WP_157677309.1	Stage 0 sporulation protein J	3.9	1.9	0.5	2.3	0.5	0.2	9.4	2.4	0.3
WP_198306619.1	Lipoprotein LpqB	7.3	1.2	0.2	4.0	0.8	0.2	17.3	2.3	0.1
WP_089609112.1	L-seryl-tRNA(Sec) selenium transferase	6.7	0.4	0.1	4.0	0.0	0.0	11.0	1.3	0.1
WP_089609113.1	Selenocysteine-specific elongation factor	5.4	3.4	0.6	3.0	1.4	0.5	5.9	3.2	0.5
WP_089609116.1	Lipopolysaccharide export system ATP-binding protein LptB	2.0	0.1	0.0	1.0	0.0	0.0	7.1	1.8	0.3

WP_089609118.1	hypothetical protein	25.2	2.6	0.1	16.7	2.5	0.1	40.7	3.3	0.1
WP_089609120.1	hypothetical protein	21.9	3.3	0.2	13.0	1.4	0.1	32.4	3.7	0.1
WP_089609123.1	GTP-binding protein TypA/BipA	7.3	1.8	0.2	4.7	1.7	0.4	8.8	2.3	0.3
WP_089609130.1	indolepyruvate oxidoreductase subunit beta	11.4	2.3	0.2	7.3	0.9	0.1	38.2	4.3	0.1
WP_089609132.1	Oligoendopeptidase F, plasmid	14.2	4.0	0.3	8.0	1.4	0.2	17.9	4.4	0.2
WP_089609133.1	TraR/DksA C4-type zinc finger protein	3.4	0.8	0.2	2.3	0.5	0.2	15.6	0.0	0.0
WP_089609134.1	hypothetical protein	5.3	1.1	0.2	2.7	0.5	0.2	5.1	0.8	0.2
WP_089609137.1	Acetolactate synthase large subunit	20.8	5.4	0.3	11.7	3.3	0.3	25.6	5.6	0.2
WP_089609138.1	Acetolactate synthase small subunit	9.0	1.6	0.2	5.7	0.5	0.1	27.6	3.1	0.1
WP_089609140.1	Aminopeptidase T	4.4	1.7	0.4	2.7	0.5	0.2	11.2	1.7	0.2
WP_089609142.1	Ornithine carbamoyltransferase	15.9	3.3	0.2	9.3	2.1	0.2	31.3	7.4	0.2
WP_089609144.1	Thiol-disulfide oxidoreductase ResA	5.0	1.6	0.3	3.3	0.9	0.3	22.3	3.7	0.2
WP_089609147.1	ABC transporter permease	3.6	0.5	0.1	2.0	0.0	0.0	8.0	0.0	0.0
WP_089609148.1	Ribose import ATP-binding protein RbsA	3.2	0.5	0.2	2.3	0.5	0.2	7.2	1.0	0.1
WP_089612490.1	Purine-binding protein	26.0	3.3	0.1	15.7	2.1	0.1	51.3	10.3	0.2
WP_089609150.1	SAM-dependent methyltransferase	5.7	0.3	0.0	3.3	0.5	0.1	13.6	2.1	0.2
WP_198306622.1	peptidoglycan-binding protein	2.0	0.0	0.0	1.0	0.0	0.0	4.0	0.0	0.0
WP_089609153.1	Inosine-5'-monophosphate dehydrogenase	12.6	2.3	0.2	7.3	0.9	0.1	39.0	8.7	0.2
WP_089609154.1	lactate utilization protein	11.5	0.7	0.1	6.7	0.9	0.1	27.4	1.4	0.1
WP_198306623.1	Signal peptidase I P	6.9	1.1	0.2	5.3	0.9	0.2	41.2	9.9	0.2
WP_089609156.1	HTH-type transcriptional regulator CysL	4.8	2.8	0.6	2.7	0.9	0.4	9.3	2.1	0.2
WP_089609157.1	Ribosome-binding ATPase YchF	18.0	4.7	0.3	9.7	2.5	0.3	32.7	6.7	0.2
WP_089609158.1	Putative HMP/thiamine import ATP-binding protein YkoD	2.9	0.1	0.0	2.0	0.0	0.0	4.5	0.2	0.0
WP_089609160.1	5-hydroxybenzimidazole synthase BzaA	18.3	2.6	0.1	13.3	1.2	0.1	28.6	3.8	0.1
WP_089609161.1	5-hydroxybenzimidazole synthase BzaB	13.4	1.8	0.1	7.7	0.5	0.1	23.8	2.4	0.1
WP_089609163.1	Anaerobic magnesium-protoporphyrin IX monomethyl ester cyclase	7.7	1.4	0.2	4.3	0.5	0.1	12.1	1.1	0.1
WP_157677319.1	Epoxyqueuosine reductase	2.7	1.2	0.4	1.3	0.5	0.4	8.4	2.7	0.3
WP_089609174.1	Selenide, water dikinase	13.7	3.1	0.2	8.0	1.4	0.2	32.3	3.6	0.1
WP_089609180.1	30S ribosomal protein S6	9.6	1.4	0.1	7.0	0.8	0.1	54.4	6.0	0.1
WP_089609181.1	Single-stranded DNA-binding protein A	11.7	0.3	0.0	6.7	0.5	0.1	49.2	0.0	0.0
WP_089609182.1	30S ribosomal protein S18	7.2	1.4	0.2	4.7	0.5	0.1	40.9	0.0	0.0
WP_089609185.1	50S ribosomal protein L9	10.5	1.3	0.1	6.0	0.8	0.1	38.3	0.3	0.0
WP_089609193.1	Aklanonic acid methyl ester cyclase DnrD	4.0	0.0	0.0	2.0	0.0	0.0	13.4	0.0	0.0
WP_089609195.1	hypothetical protein	5.7	1.6	0.3	3.0	0.8	0.3	9.2	2.2	0.2
WP_089609198.1	Ketol-acid reductoisomerase (NAD(+))	23.6	0.3	0.0	21.3	0.5	0.0	47.0	2.3	0.0
WP_089609199.1	2-hydroxymuconate tautomerase	6.4	1.2	0.2	7.0	0.0	0.0	59.6	10.8	0.2
WP_089609200.1	Pyrophosphate--fructose 6-phosphate 1-phosphotransferase	10.8	1.1	0.1	6.3	0.5	0.1	20.0	0.0	0.0
WP_089609203.1	Putrescine aminotransferase	10.8	1.4	0.1	6.0	0.8	0.1	18.7	2.9	0.2
WP_089609205.1	Adenylosuccinate synthetase	14.7	3.1	0.2	9.0	2.2	0.2	24.2	5.3	0.2
WP_089609209.1	hypothetical protein	1.9	0.2	0.1	1.0	0.0	0.0	3.6	0.0	0.0
WP_157677327.1	copper amine oxidase N-terminal domain-containing protein	3.5	1.9	0.5	1.7	0.5	0.3	7.8	2.0	0.3
WP_198306630.1	M23 family metallopeptidase	21.7	4.8	0.2	15.0	2.2	0.1	27.7	2.6	0.1
WP_089609214.1	putative protein YccU	3.1	1.1	0.4	2.0	0.0	0.0	20.0	0.0	0.0
WP_089609215.1	Thioredoxin reductase	17.4	4.3	0.2	11.3	2.5	0.2	35.4	5.2	0.1
WP_089609216.1	Copper chaperone CopZ	3.8	1.2	0.3	3.0	0.8	0.3	36.7	4.8	0.1
WP_089609227.1	hypothetical protein	5.8	1.9	0.3	3.7	0.9	0.3	17.6	3.1	0.2
WP_089611957.1	Polyphosphate kinase	4.4	0.6	0.1	2.7	0.5	0.2	6.7	1.4	0.2
WP_198306631.1	Exopolyphosphatase	2.8	1.3	0.5	1.7	0.5	0.3	4.8	1.1	0.2
WP_198306632.1	CYTH and CHAD domain-containing protein	10.1	3.8	0.4	5.7	1.7	0.3	19.6	5.8	0.3
WP_089609234.1	UDP-N-acetylglucosamine 1-carboxyvinyltransferase 1	3.5	1.9	0.5	2.7	1.7	0.6	7.3	3.1	0.4
WP_198306634.1	Serine protease Do-like HtrA	11.0	2.0	0.2	6.0	0.8	0.1	20.8	2.8	0.1
WP_089609239.1	Hypoxanthine-guanine phosphoribosyltransferase	4.8	1.8	0.4	3.3	0.5	0.1	19.7	2.9	0.1
WP_089609240.1	Cytidylate kinase	11.4	0.6	0.1	7.3	1.2	0.2	25.5	3.9	0.2
WP_089609242.1	General stress protein CTC	22.3	0.1	0.0	16.0	0.8	0.1	54.9	4.0	0.1
WP_089609245.1	Cold shock-like protein CspLA	2.0	0.0	0.0	2.0	0.0	0.0	38.5	0.0	0.0
WP_089609246.1	DEAD-box ATP-dependent RNA helicase CshA	9.7	4.5	0.5	5.3	1.9	0.4	14.9	5.8	0.4
WP_089609247.1	SPFH domain-containing protein	3.3	0.6	0.2	2.0	0.0	0.0	10.1	1.9	0.2
WP_089609250.1	Hydroxylamine reductase	37.7	8.2	0.2	24.3	4.2	0.2	47.1	5.6	0.1
WP_198306637.1	hypothetical protein	6.5	2.2	0.3	3.3	0.9	0.3	11.0	2.3	0.2
WP_089612503.1	Inosine-5'-monophosphate dehydrogenase	5.4	1.7	0.3	3.3	0.5	0.1	25.3	1.0	0.0
WP_089609253.1	UPF0182 family protein	9.9	2.2	0.2	5.3	0.9	0.2	8.6	2.0	0.2
WP_089609320.1	hypothetical protein	7.9	1.2	0.2	4.3	0.5	0.1	30.6	2.4	0.1
WP_089609255.1	Vitamin B12 import ATP-binding protein BtuD	21.5	2.8	0.1	11.3	1.2	0.1	51.9	6.2	0.1
WP_089609256.1	D-alanine--D-alanine ligase	23.7	1.1	0.0	16.0	3.3	0.2	35.6	6.4	0.2
WP_089609258.1	hypothetical protein	24.6	0.8	0.0	28.3	3.3	0.1	51.6	4.1	0.1
WP_089609266.1	Arginine--tRNA ligase	14.7	3.1	0.2	7.7	0.9	0.1	17.3	3.1	0.2
WP_089609267.1	CTP synthase	3.6	1.3	0.4	3.7	0.5	0.1	7.4	2.2	0.3
WP_089610216.1	peptidoglycan bridge formation glycyltransferase FemA/FemB family protein	8.4	2.6	0.3	4.0	0.8	0.2	20.2	3.6	0.2
WP_089609272.1	Sporulation initiation phosphotransferase F	2.1	0.1	0.1	1.0	0.0	0.0	8.2	0.0	0.0
WP_089609275.1	Transcription termination factor Rho	5.9	0.4	0.1	3.3	0.5	0.1	10.8	1.1	0.1
WP_089609277.1	50S ribosomal protein L31	5.8	0.9	0.2	4.3	0.5	0.1	59.2	7.7	0.1
WP_089609278.1	Peptide chain release factor 1	19.9	3.0	0.2	12.7	0.9	0.1	34.6	3.5	0.1
WP_089609286.1	Uracil phosphoribosyltransferase	5.7	0.8	0.1	3.7	0.5	0.1	20.3	1.9	0.1
WP_089609293.1	ATP synthase subunit c	1.9	0.1	0.1	1.0	0.0	0.0	12.0	0.0	0.0
WP_089609295.1	ATP synthase subunit delta	4.7	1.3	0.3	2.3	0.5	0.2	15.1	3.3	0.2
WP_089609297.1	ATP synthase subunit alpha	3.5	0.7	0.2	2.0	0.0	0.0	8.4	0.2	0.0
WP_089609297.1	ATP synthase subunit beta	29.5	0.5	0.0	19.7	2.4	0.1	46.6	1.9	0.0
WP_089609298.1	ATP synthase epsilon chain	4.4	2.3	0.5	2.7	0.9	0.4	22.1	7.0	0.3
WP_198306639.1	UDP-N-acetylglucosamine 1-carboxyvinyltransferase 1	7.1	2.1	0.3	4.0	0.8	0.2	13.2	2.8	0.2
WP_089609306.1	Cell shape-determining protein Mbl	19.3	1.0	0.1	11.7	0.5	0.0	39.5	1.8	0.0
WP_089609307.1	copper amine oxidase N-terminal domain-containing protein	3.7	2.5	0.7	2.7	0.9	0.4	15.4	6.3	0.4
WP_089609308.1	Polyisoprenyl-teichoic acid--peptidoglycan teichoic acid transferase TagU	8.4	2.2	0.3	7.3	0.9	0.1	22.4	4.4	0.2
WP_089609310.1	S-layer homology domain-containing protein	176.8	19.7	0.1	343.0	26.7	0.1	82.8	3.5	0.0
WP_089609311.1	Membrane-bound lytic murein transglycosylase F	20.1	2.7	0.1	13.7	0.9	0.1	37.9	4.6	0.1
WP_089609313.1	Glutamine transport ATP-binding protein GlnQ	3.9	1.4	0.3	2.7	0.5	0.2	13.5	1.8	0.1
WP_089609315.1	Multidrug resistance protein MdtA	12.0	2.8	0.2	9.0	2.9	0.3	28.0	6.2	0.2
WP_089609316.1	Swarming motility protein SwrC	39.4	3.0	0.1	23.0	1.4	0.1	22.3	1.6	0.1
WP_089609317.1	TolC family protein	46.8	6.3	0.1	30.0	2.4	0.1	61.3	3.0	0.0
WP_089609319.1	S-layer homology domain-containing protein	34.8	1.0	0.0	22.3	1.2	0.1	47.2	2.0	0.0
WP_089609320.1	S-layer homology domain-containing protein	33.7	2.5	0.1	32.3	3.1	0.1	39.2	1.4	0.0
WP_089609321.1	hypothetical protein	4.8	0.8	0.2	2.7	0.5	0.2	5.0	0.1	0.0
WP_089609326.1	Cell division coordinator CpoB	20.4	3.3	0.2	14.0	1.4	0.1	60.6	5.7	0.1
WP_089609332.1	Ribosome hibernation promotion factor	12.4	2.4	0.2	7.7	1.2	0.2	34.9	0.8	0.0
WP_089609333.1	HD domain-containing protein	1.9	0.2	0.1	1.0	0.0	0.0	2.4	0.0	0.0
WP_242965367.1	Leu/Ile/Val-binding protein	12.4	1.5	0.1	8.0	2.2	0.3	32.6	1.6	0.0
WP_089609343.1	High-affinity branched-chain amino acid transport ATP-binding protein LivF	4.6	2.5	0.6	2.3	0.9	0.4	15.0	5.5	0.4
WP_242965448.1	Murein hydrolase activator EnvC	27.8	3.1	0.1	19.3	1.2	0.1	33.7	3.4	0.1
WP_089609346.1	Carboxy-terminal processing protease CtpB	10.5	2.1	0.2	5.7	0.9	0.2	19.8	3.1	0.2
WP_089609348.1	Ion-translocating oxidoreductase complex subunit B	4.2	1.3	0.3	2.3	0.5	0.2	8.1	1.5	0.2
WP_089609353.1	efflux RND transporter periplasmic adaptor subunit	6.4	2.1	0.3	3.3	0.9	0.3	14.2	4.6	0.3
WP_089609354.1	TolC family protein	9.4	1.5	0.2	5.3	0.5	0.1	17.3	0.4	0.0
WP_089609358.1	UvrABC system protein A	4.1	1.6	0.4	3.3	0.5	0.1	4.3	0.9	0.2
WP_089609362.1	1,4-alpha-glucan branching enzyme GlgB	5.5	3.3	0.6	3.3	1.2	0.4	6.3	2.8	0.4
WP_242965242.1	Glycogen phosphorylase	10.7	3.8	0.4	6.0	1.6	0.3	12.6	3.4	0.3
WP_089609363.1	Glycogen synthase	21.3	3.7	0.2	12.3	1.7	0.1	30.4	5.3	0.2
WP_089609364.1	Alpha-1,4-glucan:maltose-1-phosphate maltosyltransferase	9.9	6.6	0.7	5.3	2.6	0.5	7.4	3.2	0.4
WP_089609365.1	Glutamine synthetase	16.9	3.8	0.2	9.3	1.2	0.1	29.1	4.8	0.2
WP_157677534.1	Diaminopimelate epimerase	5.7	0.5	0.1	3.3	0.5	0.1	16.2	0.0	0.0
WP_089609369.1	HTH-type transcriptional repressor RspR	8.2	0.8	0.1	4.0	0.0	0.0	20.2	0.4	0.0
WP_089609371.1	Pur operon repressor	5.8	1.0	0.2	4.3	0.9	0.2	19.2	1.7	0.1
WP_089609372.1	Putative septation protein SpoVG	3.4	1.3	0.4	2.0	0.8	0.4	27.4	11.8	0.4
WP_157677339.1	Bifunctional protein GlmU	10.5	1.4	0.1	6.7	0.5	0.1	16.6	4.1	0.2
WP_089609374.1	Ribose-phosphate pyrophosphokinase	17.0	3.0	0.2	9.7	1.2	0.1	33.5	2.9	0.1
WP_089609378.1	Foldase protein PrsA 2	37.5	1.6	0.0	19.7	0.5	0.0	68.2	4.0	0.1
WP_242965378.1	Nucleoside triphosphate pyrophosphohydrolase	3.3	1.2	0.3	1.7	0.5	0.3	10.3	3.4	0.3
WP_089609380.1	DNA-binding protein HU	14.8	2.5	0.2	13.0	0.8	0.1	65.6	18.0	0.3
WP_089609383.1	putative sulfate exporter family transporter	9.5	3.2	0.3	5.0	1.6	0.3	9.0	2.3	0.3
WP_089609394.1	Peptidyl-prolyl cis-trans isomerase A	7.1	0.9	0.1	5.0	0.8	0.2	37.5	6.6	0.2
WP_089609406.1	ATP-dependent zinc metalloprotease FtsH	17.3	1.1	0.1	9.7	0.9	0.1	18.1	1.7	0.1
WP_089609407.1	Nucleoside diphosphate kinase	12.5	1.4	0.1	11.7	0.5	0.0	46.3	0.0	0.0
WP_089609410.1	Thiosulfate sulfurtransferase GlpE	21.5	0.5	0.0	17.3	1.7	0.1	51.7	4.3	0.1
WP_089609412.1	Ig-like domain-containing protein	22.0	2.1	0.1	22.7	3.9	0.2	23.0	0.6	0.0
WP_089609413.1	S-layer homology domain-containing protein	37.4	6.5	0.2	56.0	8.8	0.2	38.2	3.9	0.1
WP_089609414.1	dockerin type I repeat-containing protein	14.7	1.4	0.1	19.0	2.9	0.2	48.7	2.2	0.0
WP_089609415.1	S-layer homology domain-containing protein	45.3	1.2	0.0	43.3	5.2	0.1	56.9	2.8	0.1
WP_089609416.1	Bifunctional uridylyltransferase/uridylyl-removing enzyme	2.0	0.1	0.1	1.3	0.5	0.4	8.8	4.1	0.5
WP_089609417.1	dihydropteroate synthase	16.4	1.6	0.1	10.7	1.2	0.1	21.1	2.3	0.1
WP_089609418.1	Dihydroneopterin aldolase	5.8	1.8	0.3	3.3	1.2	0.4	42.4	17.5	0.4
WP_089609419.1	2-amino-4-hydroxy-6-hydroxymethyldihydropteridine pyrophosphokinase	2.0	0.0	0.0	1.0	0.0	0.0	9.0	0.0	0.0
WP_089609423.1	ferritin family protein	20.0	4.4	0.2	11.7	1.7	0.1	62.3	5.7	0.1
WP_089609428.1	Type III pantothenate kinase	3.4	0.8	0.2	2.0	0.0	0.0	8.9	0.0	0.0
WP_089609433.1	Transcription elongation factor GreA	4.8	1.0	0.2	2.7	0.9	0.4	19.4	6.9	0.4
WP_198306696.1	Phosphoribosylaminoimidazole-succinocarboxamide synthase	19.3	1.8	0.1	11.3	0.5	0.0	38.9	5.0	0.1
WP_089609434.1	Glutamine--fructose-6-phosphate aminotransferase [isomerizing]	26.3	1.9	0.1	15.0	2.2	0.1	31.7	2.1	0.1
WP_089609435.1	Glutamine synthetase	10.9	1.3	0.1	6.3	0.5	0.1	17.4	2.1	0.1
WP_242965381.1	Amidophosphoribosyltransferase	12.0	5.1	0.4	8.7	1.9	0.2	22.8	5.9	0.3
WP_089609437.1	GMP synthase [glutamine-hydrolyzing]	11.3	4.1	0.4	6.3	1.7	0.3	16.8	4.7	0.3
WP_089609440.1	Protein-arginine kinase activator protein	8.8	2.7	0.3	5.3	1.2	0.2	37.8	9.2	0.2
WP_089612513.1	DUF1573 domain-containing protein	8.3	0.3	0.0	4.0	0.0	0.0	40.6	0.0	0.0
WP_089609444.1	RNA polymerase-binding transcription factor CarD	4.4	1.8	0.4	2.7	0.5	0.2	20.3	4.0	0.2
WP_089612515.1	Cysteine--tRNA ligase	18.0	0.7	0.0	10.3	0.5	0.0	18.9	0.4	0.0
WP_089611518.1	Putative TrmH family tRNA/rRNA methyltransferase	3.2	0.8	0.3	1.7	0.5	0.3	11.4	3.2	0.3
WP_089612517.1	Glutamine--tRNA ligase	15.7	1.8	0.1	7.7	0.5	0.1	17.5	1.1	0.1
WP_089609456.1	Transcription termination/antitermination protein NusG	8.3	2.2	0.3	4.7	0.9	0.2	41.0	9.2	0.2
WP_089609457.1	50S ribosomal protein L11	18.0	1.5	0.1	12.3	1.2	0.1	47.8	0.3	0.0

WP_089609458.1	50S ribosomal protein L1	20.5	1.3	0.1	11.3	0.5	0.0	42.2	4.1	0.1
WP_242965451.1	50S ribosomal protein L10	14.2	3.5	0.2	8.3	2.1	0.2	37.9	13.4	0.4
WP_089609460.1	50S ribosomal protein L7/L12	19.3	2.4	0.1	19.7	2.1	0.1	77.0	7.1	0.1
WP_089609463.1	Ribosome-associated protein L7Ae-like protein	2.6	1.0	0.4	1.3	0.5	0.4	27.2	9.7	0.4
WP_089609464.1	30S ribosomal protein S12	5.0	0.8	0.2	3.7	0.5	0.1	22.9	2.9	0.1
WP_089609465.1	30S ribosomal protein S7	18.5	1.8	0.1	16.7	2.4	0.1	59.0	3.9	0.1
WP_089609467.1	30S ribosomal protein S10	11.7	1.3	0.1	7.3	1.7	0.2	50.7	6.7	0.1
WP_089609468.1	50S ribosomal protein L3	18.0	1.4	0.1	11.7	0.5	0.0	49.3	2.1	0.0
WP_089609469.1	50S ribosomal protein L4	9.2	1.3	0.1	10.3	1.7	0.2	26.4	1.4	0.1
WP_089609470.1	50S ribosomal protein L23	10.1	1.4	0.1	5.7	0.5	0.1	62.1	0.0	0.0
WP_089609471.1	50S ribosomal protein L2	18.7	2.5	0.1	14.3	1.7	0.1	41.4	4.2	0.1
WP_089609472.1	30S ribosomal protein S19	14.5	0.9	0.1	11.0	0.8	0.1	72.3	0.0	0.0
WP_089609473.1	50S ribosomal protein L22	8.4	2.2	0.3	5.3	2.1	0.4	41.0	2.9	0.1
WP_089609474.1	30S ribosomal protein S3	20.4	1.3	0.1	11.7	0.5	0.0	49.0	1.5	0.0
WP_089609475.1	50S ribosomal protein L16	8.0	2.0	0.2	4.0	0.8	0.2	41.7	9.3	0.2
WP_089609476.1	50S ribosomal protein L29	11.3	0.7	0.1	6.7	0.5	0.1	56.7	0.0	0.0
WP_242965452.1	30S ribosomal protein S17	14.7	1.4	0.1	14.7	0.5	0.0	51.0	6.0	0.1
WP_089609478.1	50S ribosomal protein L14	5.7	0.4	0.1	4.3	1.2	0.3	40.7	8.8	0.2
WP_089609479.1	50S ribosomal protein L24	13.3	0.7	0.1	9.7	0.9	0.1	54.1	3.9	0.1
WP_089609480.1	50S ribosomal protein L5	20.8	1.7	0.1	14.0	1.4	0.1	58.3	5.1	0.1
WP_089609482.1	30S ribosomal protein S8	9.1	1.8	0.2	7.0	1.4	0.2	45.0	9.3	0.2
WP_089609483.1	50S ribosomal protein L6	17.1	2.4	0.1	10.7	1.9	0.2	48.5	12.4	0.3
WP_089609484.1	50S ribosomal protein L18	11.4	0.9	0.1	6.7	0.9	0.1	51.4	5.5	0.1
WP_089609485.1	30S ribosomal protein S5	15.8	0.3	0.0	11.3	0.5	0.0	46.4	0.0	0.0
WP_089609486.1	50S ribosomal protein L30	4.7	0.6	0.1	3.3	0.5	0.1	37.1	9.1	0.2
WP_089609487.1	50S ribosomal protein L15	7.9	1.6	0.2	5.0	1.4	0.3	38.8	3.6	0.1
WP_089609488.1	Protein translocase subunit SecY	6.1	2.5	0.4	4.3	1.7	0.4	11.9	3.8	0.3
WP_089609489.1	Adenylate kinase	23.5	2.8	0.1	15.3	1.2	0.1	61.9	3.2	0.1
WP_089609490.1	Methionine aminopeptidase 1	3.8	2.0	0.5	2.0	0.8	0.4	15.6	7.0	0.5
WP_089609492.1	Translation initiation factor IF-1	1.8	0.3	0.2	1.0	0.0	0.0	16.7	0.0	0.0
WP_089609494.1	30S ribosomal protein S13	7.0	0.6	0.1	4.7	0.5	0.1	37.7	3.1	0.1
WP_089609495.1	30S ribosomal protein S11	6.7	1.0	0.1	8.3	0.5	0.1	39.8	0.0	0.0
WP_089609496.1	30S ribosomal protein S4	23.1	0.7	0.0	15.3	1.2	0.1	49.5	2.0	0.0
WP_089609497.1	DNA-directed RNA polymerase subunit alpha	18.6	0.1	0.0	10.7	0.5	0.0	29.8	0.9	0.0
WP_089609498.1	50S ribosomal protein L17	6.9	1.9	0.3	4.7	0.5	0.1	33.9	4.8	0.1
WP_242965453.1	50S ribosomal protein L13	8.1	1.1	0.1	6.7	0.5	0.1	46.1	4.4	0.1
WP_089609504.1	30S ribosomal protein S9	8.4	0.5	0.1	6.0	0.8	0.1	40.5	2.9	0.1
WP_089609507.1	2,3-dihydroxyphenylpropionate/2,3-dihydroxicinnamic acid 1,2-dioxygenase	10.5	2.2	0.2	6.0	0.8	0.1	19.1	1.6	0.1
WP_198306641.1	YajQ family cyclic di-GMP-binding protein	8.9	2.6	0.3	7.7	0.5	0.1	35.2	6.5	0.2
WP_089609510.1	N-acetyl-gamma-glutamyl-phosphate reductase	17.7	1.8	0.1	9.3	1.2	0.1	40.7	4.9	0.1
WP_089611532.1	Arginine biosynthesis bifunctional protein ArgJ	14.8	3.3	0.2	9.3	1.2	0.1	29.3	1.9	0.1
WP_089609511.1	Acetylglutamate kinase	6.0	0.0	0.0	3.0	0.0	0.0	16.0	0.0	0.0
WP_157677348.1	Acetylornithine aminotransferase	24.6	2.2	0.1	13.0	0.8	0.1	45.8	4.6	0.1
WP_089609512.1	Argininosuccinate synthase	15.0	4.2	0.3	8.0	1.6	0.2	23.6	4.6	0.2
WP_089609514.1	S-inosyl-L-homocysteine hydrolase	5.4	0.2	0.0	3.0	0.0	0.0	15.0	0.0	0.0
WP_089609515.1	Lactate racemase	9.9	0.8	0.1	5.7	0.5	0.1	17.7	1.0	0.1
WP_089609519.1	Na(+)-translocating NADH-quinone reductase subunit F	33.0	2.5	0.1	25.3	1.7	0.1	36.7	2.0	0.1
WP_089609535.1	Glutamine-dependent NAD(+) synthetase	3.7	1.4	0.4	2.0	0.8	0.4	3.9	1.4	0.4
WP_089609536.1	Argininosuccinate lyase	12.9	3.6	0.3	7.3	0.9	0.1	20.7	3.2	0.2
WP_089609539.1	Ion-translocating oxidoreductase complex subunit B	6.9	1.9	0.3	3.7	0.9	0.3	22.7	5.6	0.2
WP_089609540.1	hypothetical protein	9.6	1.8	0.2	5.0	0.8	0.2	27.1	4.4	0.2
WP_198306622.1	Cytidine deaminase	6.0	3.3	0.6	3.0	1.4	0.5	30.2	13.2	0.4
WP_089609549.1	GTPase Era	4.8	2.5	0.5	2.7	0.9	0.4	10.2	3.8	0.4
WP_089609555.1	ABC transporter substrate-binding protein	27.7	2.0	0.1	21.7	0.9	0.0	39.3	2.7	0.1
WP_089609557.1	Coenzyme A disulfide reductase	3.2	0.9	0.3	2.7	1.7	0.6	5.2	4.1	0.8
WP_089609559.1	Glycine--tRNA ligase alpha subunit	18.5	3.6	0.2	10.0	0.8	0.1	36.3	2.4	0.1
WP_089609563.1	type II toxin-antitoxin system RelB/DinJ family antitoxin	8.8	1.9	0.2	5.7	0.9	0.2	43.6	8.4	0.2
WP_089609566.1	hypothetical protein	2.0	0.0	0.0	1.0	0.0	0.0	14.8	0.0	0.0
WP_157677350.1	Arsenite methyltransferase	6.9	1.0	0.1	3.7	0.5	0.1	26.5	4.2	0.2
WP_198306648.1	HTH-type transcriptional repressor RspR	8.6	1.4	0.2	5.7	0.5	0.1	29.6	3.7	0.1
WP_089609578.1	Thioredoxin reductase	19.6	2.0	0.1	14.7	0.5	0.0	38.9	2.0	0.1
WP_089609579.1	Thioredoxin	10.7	1.8	0.2	8.0	0.8	0.1	51.0	6.8	0.1
WP_089609583.1	GrdX family protein	2.7	1.2	0.4	1.3	0.5	0.4	15.3	4.2	0.3
WP_089609586.1	hypothetical protein	7.2	2.0	0.3	5.7	1.7	0.3	25.3	7.4	0.3
WP_242965389.1	Vitamin B12-binding protein	10.4	1.5	0.1	6.3	0.5	0.1	22.6	4.4	0.2
WP_089609590.1	CooT family nickel-binding protein	3.0	1.4	0.5	1.7	0.5	0.3	40.9	12.9	0.3
WP_089609593.1	High-affinity zinc uptake system binding-protein ZnuA	5.4	2.4	0.4	3.0	0.8	0.3	15.3	4.9	0.3
WP_089609594.1	Zinc import ATP-binding protein ZnuC	2.0	0.0	0.0	1.0	0.0	0.0	6.8	0.0	0.0
WP_089609598.1	Formylaminopyrimidine-binding protein	3.1	1.2	0.4	2.0	0.8	0.4	6.1	1.1	0.2
WP_089609601.1	DNA primase	2.6	1.2	0.5	1.3	0.5	0.4	2.5	1.0	0.4
WP_089609602.1	RNA polymerase sigma factor SigA	15.8	2.3	0.1	9.0	0.8	0.1	20.0	2.7	0.1
WP_089609604.1	GTP cyclohydrolase 1 type 2	4.7	0.5	0.1	2.7	0.5	0.2	8.2	1.8	0.2
WP_157677352.1	Histidinol-phosphate aminotransferase	11.2	1.3	0.1	8.3	0.5	0.1	26.5	1.7	0.1
WP_198306701.1	Glutamine transport ATP-binding protein GlnQ	5.2	2.0	0.4	2.7	0.9	0.4	15.7	6.5	0.4
WP_089609618.1	Phosphate-binding protein PstS 1	16.1	0.6	0.0	10.0	0.0	0.0	37.0	2.1	0.1
WP_089609622.1	Phosphate-specific transport system accessory protein PhoU	8.7	1.5	0.2	5.3	0.5	0.1	31.2	3.9	0.1
WP_089609624.1	metal-dependent hydrolase	5.3	1.2	0.2	3.7	0.5	0.1	18.0	2.5	0.1
WP_089609631.1	PDZ domain-containing protein	29.1	1.8	0.1	17.3	0.5	0.0	35.1	1.5	0.0
WP_157677355.1	hypothetical protein	11.2	4.4	0.4	6.7	2.4	0.4	32.5	9.7	0.3
WP_089609636.1	BlaI/MecI/CopY family transcriptional regulator	3.0	0.8	0.3	1.7	0.5	0.3	25.8	5.3	0.2
WP_157677356.1	copper amine oxidase N-terminal domain-containing protein	7.6	1.5	0.2	4.0	0.8	0.2	9.3	1.8	0.2
WP_089609645.1	Nicotinate-nucleotide--dimethylbenzimidazole phosphoribosyltransferase	22.9	3.5	0.2	34.0	5.4	0.2	41.3	3.4	0.1
WP_089609647.1	Glutamate 2,3-aminomutase	5.6	1.9	0.3	3.7	1.2	0.3	9.6	2.4	0.2
WP_089609650.1	Nicotinate phosphoribosyltransferase pncB2	18.1	3.7	0.2	9.3	1.7	0.2	20.9	3.6	0.2
WP_089609686.1	Phosphate acyltransferase	10.2	1.8	0.2	6.0	0.0	0.0	23.7	4.0	0.2
WP_089609687.1	3-oxoacyl-[acyl-carrier-protein] synthase 3 protein 1	13.0	2.7	0.2	8.0	1.4	0.2	35.8	5.4	0.2
WP_089609688.1	NADH:quinone reductase	12.5	1.0	0.1	6.7	0.9	0.1	27.1	1.8	0.1
WP_089609689.1	Malonyl CoA-acyl carrier protein transacylase	5.1	2.1	0.4	4.7	2.1	0.4	22.1	8.2	0.4
WP_089609690.1	3-oxoacyl-[acyl-carrier-protein] reductase FabG	11.3	1.1	0.1	7.7	0.5	0.1	31.3	3.1	0.1
WP_089609691.1	Acyl carrier protein	3.2	1.2	0.4	1.7	0.5	0.3	34.2	13.3	0.4
WP_089609692.1	Inosine-5'-monophosphate dehydrogenase	5.5	2.2	0.4	3.0	0.8	0.3	13.0	4.5	0.4
WP_198306654.1	3-oxoacyl-[acyl-carrier-protein] synthase 2	9.6	4.7	0.5	7.0	1.6	0.2	25.6	8.9	0.3
WP_242965394.1	Ribonuclease 3	3.8	1.5	0.4	3.0	0.8	0.3	13.8	2.7	0.2
WP_089609696.1	DUF4445 domain-containing protein	4.5	1.4	0.3	2.3	0.5	0.2	6.1	1.8	0.3
WP_089609697.1	NYN domain-containing protein	6.1	1.6	0.3	3.7	0.5	0.1	21.3	4.9	0.2
WP_242965395.1	Signal recognition particle receptor FtsY	3.6	1.0	0.3	2.3	0.9	0.4	8.5	3.2	0.4
WP_089609701.1	S-methyl-5'-thioinosine phosphorylase	6.7	1.7	0.3	5.3	0.9	0.2	24.8	4.8	0.2
WP_089609702.1	Methylthioribose-1-phosphate isomerase	2.7	1.2	0.4	1.3	0.5	0.4	5.1	1.8	0.4
WP_242965396.1	5-deoxy-D-ribulose 1-phosphate aldolase	2.0	0.0	0.0	1.0	0.0	0.0	6.4	0.0	0.0
WP_089609703.1	5-methylthioadenosine/S-adenosylhomocysteine deaminase	12.5	2.4	0.2	8.0	1.4	0.2	21.3	4.9	0.2
WP_089609706.1	hypothetical protein	13.8	3.5	0.3	10.0	2.2	0.2	27.1	9.0	0.3
WP_089609708.1	Cobalt-dependent inorganic pyrophosphatase	6.4	4.3	0.7	4.0	1.6	0.4	10.2	3.6	0.4
WP_089609710.1	Signal recognition particle protein	19.1	1.7	0.1	12.0	0.8	0.1	29.0	2.7	0.1
WP_089609711.1	30S ribosomal protein S16	8.0	0.0	0.0	5.7	0.5	0.1	41.9	0.0	0.0
WP_089609712.1	KH domain-containing protein	2.6	0.9	0.3	2.0	0.8	0.4	32.4	12.8	0.4
WP_198306705.1	Calcium-transporting ATPase 1	22.5	2.6	0.1	12.0	0.0	0.0	19.1	2.3	0.1
WP_089609719.1	type II toxin-antitoxin system HicB family antitoxin	5.1	1.0	0.2	2.7	0.5	0.2	30.1	8.3	0.3
WP_089609725.1	Protein GrpE	2.8	1.4	0.5	1.3	0.5	0.4	8.1	3.5	0.4
WP_089609726.1	Chaperone protein DnaK	7.4	3.6	0.5	5.3	1.7	0.3	14.0	5.9	0.4
WP_089612539.1	4-hydroxy-tetrahydrodipicolinate synthase	12.5	1.4	0.1	8.0	0.8	0.1	28.9	3.3	0.1
WP_089609755.1	Endonuclease MutS2	2.0	0.0	0.0	1.0	0.0	0.0	14.0	0.0	0.0
WP_089609757.1	Phosphonoacetaldehyde hydrolase	11.5	0.8	0.1	6.7	0.5	0.1	35.6	1.4	0.0
WP_089609760.1	ATP-dependent zinc metalloprotease FtsH 4	21.7	3.1	0.1	12.0	1.6	0.1	23.2	3.2	0.1
WP_089609761.1	General stress protein A	3.0	1.7	0.6	1.7	0.9	0.6	9.2	5.2	0.6
WP_089609765.1	Septum site-determining protein MinD	3.8	1.0	0.3	3.0	0.8	0.3	9.2	0.4	0.0
WP_157677365.1	hypothetical protein	2.1	0.3	0.2	1.3	0.5	0.4	0.8	0.3	0.3
WP_198306657.1	Protein LemA	2.1	0.2	0.1	1.0	0.0	0.0	7.0	0.0	0.0
WP_089609791.1	Phosphorylated carbohydrates phosphatase	2.9	0.9	0.3	1.7	0.5	0.3	10.6	2.3	0.2
WP_089609802.1	Arylsulfate sulfotransferase AssT	4.7	2.9	0.6	2.7	1.2	0.5	6.0	3.2	0.5
WP_089609813.1	hypothetical protein	4.2	0.9	0.2	2.3	0.5	0.2	26.2	0.8	0.0
WP_089612547.1	Ribosomal RNA large subunit methyltransferase K/L	5.2	2.4	0.5	3.7	1.2	0.3	13.2	4.4	0.3
WP_089609817.1	Threonine synthase	14.6	5.5	0.4	8.0	2.4	0.3	19.3	4.8	0.3
WP_089609818.1	Hydrogenase maturation factor HypA	6.6	1.4	0.2	4.7	0.9	0.2	50.0	15.0	0.3
WP_089609819.1	Cold shock protein CspB	10.2	0.8	0.1	9.3	0.9	0.1	86.7	5.1	0.1
WP_089609836.1	putative protein	12.2	0.5	0.0	6.0	0.0	0.0	25.6	0.6	0.0
WP_089609838.1	Cobalt-precorrin-4 C(11)-methyltransferase	5.4	0.8	0.1	2.7	0.5	0.2	18.0	3.1	0.2
WP_089609841.1	nucleotidyltransferase substrate binding protein	7.8	0.8	0.1	5.0	0.0	0.0	40.2	11.2	0.3
WP_089609842.1	Thiamine thiazole synthase	22.3	1.7	0.1	13.7	0.5	0.0	24.7	0.5	0.0
WP_089609844.1	Dihydroxy-acid dehydratase	22.7	5.1	0.2	13.7	1.7	0.1	25.4	7.5	0.3
WP_089609846.1	Acetolactate synthase large subunit	11.5	1.0	0.1	6.7	1.7	0.3	17.0	2.3	0.1
WP_089609847.1	2-isopropylmalate synthase	26.2	4.9	0.2	17.3	2.4	0.1	33.4	5.8	0.2
WP_089609849.1	2,3-dimethylmalate dehydratase small subunit	19.3	0.6	0.0	12.7	0.5	0.0	80.2	0.0	0.0
WP_089609850.1	3-isopropylmalate dehydrogenase	15.4	1.0	0.1	10.3	1.2	0.1	28.4	0.9	0.0
WP_089609851.1	(R)-citramalate synthase	25.7	2.9	0.1	15.3	0.9	0.1	33.5	1.5	0.0
WP_089609858.1	DUF881 domain-containing protein	3.3	1.2	0.3	1.7	0.5	0.3	10.3	3.0	0.3
WP_089609864.1	Phosphoglucosamine mutase	25.2	5.4	0.2	14.0	2.2	0.2	39.2	9.0	0.2
WP_089609868.1	putative glucosamine-6-phosphate deaminase 2	10.8	1.2	0.1	7.0	0.8	0.1	17.9	1.4	0.1
WP_198306664.1	Aminopeptidase YpdF	3.4	1.8	0.5	2.7	0.5	0.2	12.2	1.9	0.2
WP_198306665.1	Putative NAD(P)H nitroreductase	14.8	1.1	0.1	13.0	1.4	0.1	38.2	0.0	0.0
WP_089609876.1	Bifunctional protein PaaZ	2.4	0.7	0.3	1.3	0.5	0.4	16.9	6.2	0.4
WP_089609878.1	Iron-sulfur protein	7.5	2.0	0.3	4.0	0.8	0.2	33.1	6.6	0.2
WP_257912036.1	NADP-reducing hydrogenase subunit HndC	2.2	0.3	0.1	1.3	0.5	0.4	2.3	0.0	0.0
WP_242965461.1	Carbon monoxide dehydrogenase 2	18.3	4.0	0.2	12.3	1.7	0.1	19.9	1.6	0.1
WP_089609887.1	GTP 3',8-cyclase	15.7	4.2	0.3	8.3	1.7	0.2	47.5	13.7	0.3
WP_242965411.1	peroxiredoxin	7.4	3.2	0.4	3.7	1.2	0.3	47.5	13.3	0.3

WP_242965411.1	Selenocysteine-containing peroxiredoxin PrxU	8.1	0.1	0.0	5.0	0.8	0.2	60.2	6.4	0.1
WP_089609897.1	hypothetical protein	6.4	3.0	0.5	3.3	0.9	0.3	34.4	9.4	0.3
WP_089609911.1	Phospho-2-dehydro-3-deoxyheptonate aldolase	9.1	1.9	0.2	4.3	0.5	0.1	16.7	2.1	0.1
WP_089609914.1	Potassium transporter KimA	2.5	0.8	0.3	1.3	0.5	0.4	2.4	0.9	0.4
WP_089609918.1	Chromosomal replication initiator protein DnaA	4.8	1.2	0.3	2.7	0.9	0.4	6.9	2.0	0.3
WP_089609920.1	2-amino-4-deoxychorismate dehydrogenase	10.3	1.7	0.2	5.3	1.2	0.2	29.7	5.8	0.2
WP_089609921.1	33 kDa chaperonin	10.1	2.9	0.3	5.3	1.2	0.2	33.1	6.7	0.2
WP_089609934.1	hypothetical protein	6.1	1.4	0.2	5.0	0.8	0.2	44.7	6.9	0.2
WP_242965417.1	Peroxide-responsive repressor PerR	4.5	0.7	0.2	2.7	0.5	0.2	24.1	4.0	0.2
WP_089609937.1	Holliday junction ATP-dependent DNA helicase RuvB	20.4	1.1	0.1	14.3	0.5	0.0	37.2	1.6	0.0
WP_089609939.1	Hydrogenase maturation factor HypA	2.0	0.0	0.0	1.0	0.0	0.0	26.9	0.0	0.0
WP_089609940.1	Photosystem I assembly protein Ycf3	20.2	1.0	0.0	11.0	1.4	0.1	33.9	3.9	0.1
WP_242965419.1	Stage V sporulation protein D	5.7	2.9	0.5	4.0	1.4	0.4	11.3	4.1	0.4
WP_089609945.1	UDP-N-acetylmuramoyl-L-alanyl-D-glutamate--2,6-diaminopimelate ligase	14.3	4.1	0.3	8.0	1.4	0.2	19.1	4.1	0.2
WP_089609947.1	UDP-N-acetylmuramoylalanine--D-glutamate ligase	6.8	1.1	0.2	3.7	0.9	0.3	12.0	2.1	0.2
WP_089609949.1	UDP-N-acetylmuramate--L-alanine ligase	15.5	2.2	0.1	8.3	0.5	0.1	25.3	2.4	0.1
WP_157677382.1	Cell division protein FtsA	5.5	1.8	0.3	3.0	0.8	0.3	13.1	3.6	0.3
WP_089609952.1	Cell division protein FtsZ	25.9	0.7	0.0	15.3	0.5	0.0	40.5	1.7	0.0
WP_089609957.1	2-oxoacid:ferredoxin oxidoreductase subunit beta	8.3	3.4	0.4	4.3	1.2	0.3	23.8	5.7	0.2
WP_089609971.1	Anaerobic ribonucleoside-triphosphate reductase	6.0	0.7	0.1	3.3	0.5	0.1	6.3	2.0	0.3
WP_089609994.1	Polyphenol oxidase	5.8	1.6	0.3	3.0	0.8	0.3	15.6	2.1	0.1
WP_242965424.1	Pyridoxal phosphate homeostasis protein	5.0	1.6	0.3	2.7	0.9	0.4	16.7	3.9	0.2
WP_089609998.1	Pyrroline-5-carboxylate reductase	8.1	3.4	0.4	4.0	1.6	0.4	20.4	7.3	0.4
WP_089610002.1	Isoleucine--tRNA ligase	22.2	7.2	0.3	12.3	2.6	0.2	18.0	5.0	0.3
WP_089610004.1	PFL family protein	8.8	4.9	0.6	4.7	2.1	0.4	13.6	6.6	0.5
WP_089610005.1	sulfite reductase subunit beta (hemoprotein)	26.4	0.6	0.0	20.0	0.0	0.0	59.5	2.4	0.0
WP_089610008.1	Molybdenum cofactor cytidylyltransferase	2.9	1.0	0.4	1.7	0.5	0.3	10.9	3.1	0.3
WP_089610009.1	EF2563 family selenium-dependent molybdenum hydroxylase system protein	25.1	1.2	0.0	16.3	0.5	0.0	30.6	1.0	0.0
WP_089610011.1	Putative competence-damage inducible protein	17.8	4.1	0.2	9.7	1.2	0.1	39.0	3.4	0.1
WP_089610783.1	Monomethylamine methyltransferase MtmB	3.3	1.3	0.4	2.3	0.9	0.4	12.9	4.9	0.4
WP_089610015.1	HTH-type transcriptional repressor GlaR	2.3	0.4	0.2	1.3	0.5	0.4	7.7	2.1	0.3
WP_089610033.1	hypothetical protein	9.6	2.1	0.2	6.3	0.9	0.1	16.5	3.2	0.2
WP_089610034.1	beta-propeller domain-containing protein	9.6	1.0	0.1	4.7	0.5	0.1	12.2	1.5	0.1
WP_089610047.1	Dihydroorotate dehydrogenase B (NAD(+)), electron transfer subunit	8.8	1.6	0.2	5.3	0.9	0.2	22.8	2.6	0.1
WP_089610049.1	Orotidine 5'-phosphate decarboxylase	12.1	1.3	0.1	6.7	0.9	0.1	35.4	3.0	0.1
WP_089610050.1	Orotate phosphoribosyltransferase	10.7	1.5	0.1	6.3	0.9	0.1	35.1	0.2	0.0
WP_089610051.1	Na/Pi cotransporter family protein	5.2	1.4	0.3	3.0	0.8	0.3	7.3	1.7	0.2
WP_089610052.1	Carbon disulfide hydrolase	2.9	1.5	0.5	1.7	0.9	0.6	9.7	5.1	0.5
WP_089610053.1	Rqc2 RqcH	5.4	4.3	0.8	3.3	1.9	0.6	8.5	4.7	0.6
WP_089612573.1	YicC family protein	2.0	0.0	0.0	1.3	0.5	0.4	5.1	0.0	0.0
WP_089610055.1	Extracellular matrix regulatory protein A	7.9	1.7	0.2	4.3	0.9	0.2	62.7	11.7	0.2
WP_089610058.1	zinc ribbon domain-containing protein	12.3	1.6	0.1	7.7	0.5	0.1	50.5	9.5	0.2
WP_242965466.1	TIGR00266 family protein	2.7	1.2	0.4	1.3	0.5	0.4	6.4	0.5	0.1
WP_198306677.1	Chromosome partition protein Smc	9.1	2.4	0.3	5.3	0.5	0.1	31.8	3.1	0.1
WP_089610071.1	hypothetical protein	3.2	1.2	0.4	2.0	0.0	0.0	9.0	0.6	0.1
WP_089610104.1	Uroporphyrinogen decarboxylase	13.0	0.6	0.0	9.0	0.0	0.0	19.3	0.8	0.0
WP_089610106.1	Uroporphyrinogen decarboxylase	16.5	1.3	0.1	9.7	1.2	0.1	32.4	1.7	0.1
WP_089610120.1	Trans-4-hydroxy-L-proline dehydratase	6.2	0.3	0.1	3.3	0.5	0.1	4.6	0.6	0.1
WP_089610130.1	Peptide deformylase 1	4.0	0.0	0.0	2.0	0.0	0.0	27.8	0.0	0.0
WP_089610133.1	zinc metallopeptidase	3.0	1.0	0.3	2.0	0.0	0.0	11.4	2.3	0.2
WP_089610139.1	Serine/threonine phosphatase stp	5.7	0.3	0.1	3.3	0.5	0.1	18.5	3.7	0.2
WP_089610141.1	Serine/threonine-protein kinase PrkC	9.1	2.2	0.2	5.7	0.5	0.1	12.1	1.4	0.1
WP_089610149.1	Riboflavin biosynthesis protein RibBA	18.7	3.1	0.2	9.0	1.4	0.2	28.2	5.3	0.2
WP_089610151.1	6,7-dimethyl-8-ribityllumazine synthase	7.5	2.2	0.3	4.3	0.5	0.1	39.7	8.9	0.2
WP_242965082.1	NAD-dependent protein deacylase sirtuin-5, mitochondrial	2.0	0.4	0.2	2.0	0.0	0.0	3.4	0.0	0.0
WP_157677401.1	Putative ABC transporter periplasmic binding protein	26.5	2.7	0.1	17.0	1.6	0.1	47.1	3.4	0.1
WP_089610177.1	sodium:proton antiporter	7.3	0.4	0.1	6.7	0.9	0.1	8.2	0.0	0.0
WP_089610182.1	C-GCAxxG-C-C family protein	7.3	1.8	0.2	3.7	0.9	0.3	24.4	3.3	0.1
WP_089610184.1	hypothetical protein	4.5	1.2	0.3	2.3	0.5	0.2	38.2	8.3	0.2
WP_089610188.1	hypothetical protein	5.1	1.9	0.4	4.0	0.8	0.2	30.6	6.6	0.2
WP_089610193.1	putative zinc-binding protein	6.1	0.2	0.0	3.3	0.5	0.1	34.6	0.0	0.0
WP_089610195.1	putative zinc-binding protein	4.8	1.1	0.2	2.7	0.5	0.2	27.3	8.1	0.3
WP_089610210.1	D-alanine--D-alanine ligase A	24.7	3.8	0.2	14.3	2.9	0.2	42.5	3.5	0.1
WP_089610216.1	DNA topoisomerase 1	4.5	1.9	0.4	3.0	1.4	0.5	4.9	2.1	0.4
WP_089610218.1	Methylenetetrahydrofolate--tRNA-(uracil-5-)-methyltransferase TrmFO	1.8	0.3	0.1	1.0	0.0	0.0	2.2	0.0	0.0
WP_089610219.1	ATP-dependent protease subunit HslV	2.0	0.0	0.0	1.0	0.0	0.0	6.3	0.0	0.0
WP_089610221.1	ATP-dependent protease ATPase subunit ClpY	21.8	0.5	0.0	12.0	0.0	0.0	22.5	0.4	0.0
WP_089610229.1	Elongation factor Ts	28.2	1.1	0.0	21.3	1.2	0.1	58.8	2.7	0.0
WP_089610231.1	Uridylate kinase	5.8	1.3	0.2	3.0	0.8	0.3	13.0	4.1	0.3
WP_089610233.1	Ribosome-recycling factor	26.4	1.0	0.0	15.0	1.6	0.1	59.3	7.0	0.1
WP_089610237.1	Ferredoxin	2.0	0.0	0.0	1.7	0.5	0.3	38.9	0.0	0.0
WP_089610248.1	4-hydroxy-3-methylbut-2-en-1-yl diphosphate synthase (flavodoxin)	3.7	0.7	0.2	2.3	0.5	0.2	8.1	2.4	0.3
WP_089610250.1	Proline--tRNA ligase	21.2	1.2	0.1	10.3	0.5	0.0	24.6	2.6	0.1
WP_089612588.1	(R)-phenyllactyl-CoA dehydratase beta subunit	11.3	0.8	0.1	6.7	0.9	0.1	20.8	0.4	0.0
WP_089610273.1	sulfur reduction protein DsrE	7.0	0.2	0.0	12.7	1.2	0.1	62.7	9.7	0.2
WP_198306719.1	(R)-phenyllactyl-CoA dehydratase beta subunit	23.6	3.0	0.1	14.3	0.5	0.0	41.7	3.2	0.1
WP_089610280.1	ABC transporter substrate-binding protein	16.6	4.3	0.3	10.3	1.2	0.1	27.5	6.9	0.3
WP_089610284.1	Alkylmercury lyase	4.8	0.3	0.1	3.0	0.0	0.0	24.1	0.0	0.0
WP_089610286.1	(R)-2-hydroxyglutaryl-CoA dehydratase activating ATPase	13.9	2.0	0.1	7.0	0.8	0.1	32.8	4.8	0.1
WP_089610329.1	S-adenosylmethionine decarboxylase proenzyme	2.7	1.2	0.4	1.3	0.5	0.4	12.8	1.1	0.1
WP_089610338.1	Uroporphyrinogen decarboxylase	24.6	2.4	0.1	26.7	1.2	0.0	30.9	3.3	0.1
WP_089610351.1	Pantothenate synthetase	2.1	1.0	0.5	1.3	0.5	0.4	5.1	2.3	0.5
WP_089610353.1	Aspartate 1-decarboxylase	3.2	0.4	0.1	2.0	0.0	0.0	8.7	0.0	0.0
WP_089610355.1	hypothetical protein	3.2	0.8	0.3	2.0	0.0	0.0	4.0	0.4	0.1
WP_089610358.1	Uroporphyrinogen decarboxylase	7.3	4.1	0.6	3.7	1.7	0.5	12.9	4.4	0.3
WP_089610378.1	hypothetical protein	2.5	0.4	0.2	2.0	0.0	0.0	11.8	0.0	0.0
WP_089610395.1	Aklanonic acid methyl ester cyclase DnrD	5.0	2.3	0.5	3.7	1.2	0.3	17.9	3.1	0.2
WP_089612593.1	hypothetical protein	7.7	0.9	0.1	5.0	0.0	0.0	15.6	1.8	0.1
WP_242965103.1	Adaptive-response sensory-kinase SasA	2.0	0.0	0.0	1.0	0.0	0.0	2.1	0.0	0.0
WP_089610410.1	Elongation factor Ts, mitochondrial	10.6	2.0	0.2	18.0	2.8	0.2	41.7	4.8	0.1
WP_089610412.1	DUF1659 domain-containing protein	6.7	0.7	0.1	3.3	0.5	0.1	39.1	6.3	0.2
WP_089610414.1	DUF2922 domain-containing protein	6.6	1.4	0.2	3.7	0.5	0.1	59.2	0.0	0.0
WP_089610416.1	N-acetylmuramoyl-L-alanine amidase	5.7	2.1	0.4	3.7	1.7	0.5	15.4	4.4	0.3
WP_089610418.1	YvrJ family protein	1.9	0.2	0.1	1.0	0.0	0.0	29.2	0.0	0.0
WP_089610422.1	UTP--glucose-1-phosphate uridylyltransferase	8.9	1.3	0.1	4.7	0.5	0.1	22.2	2.4	0.1
WP_089610425.1	hypothetical protein	6.0	0.5	0.1	3.0	0.0	0.0	41.9	0.0	0.0
WP_157677415.1	DUF3841 domain-containing protein	4.0	0.0	0.0	2.0	0.0	0.0	19.1	0.0	0.0
WP_198306515.1	Nitrogenase iron protein 1	3.1	1.0	0.3	2.0	0.8	0.4	10.3	3.8	0.4
WP_089610496.1	putative protein	14.7	2.8	0.2	8.0	1.4	0.2	31.0	7.6	0.2
WP_089610504.1	YedE-related selenium metabolism membrane protein	3.1	0.6	0.2	2.7	0.9	0.4	7.2	3.1	0.4
WP_089610506.1	Putative sulfur carrier protein YedF	7.0	1.8	0.3	7.0	0.8	0.1	70.8	10.3	0.1
WP_089610507.1	DUF3343 domain-containing protein	5.3	1.4	0.3	2.7	0.5	0.2	20.6	6.0	0.3
WP_089610509.1	ATP-dependent (S)-NAD(P)H-hydrate dehydratase	9.7	3.3	0.3	5.3	1.2	0.2	22.6	4.8	0.2
WP_089610511.1	molybdenum cofactor biosynthesis protein MoaE	14.4	1.2	0.1	13.0	0.8	0.1	58.1	4.8	0.1
WP_089610514.1	DUF4179 domain-containing protein	2.4	0.4	0.2	1.3	0.5	0.4	4.8	2.5	0.5
WP_089610518.1	C4-dicarboxylate ABC transporter	1.6	0.3	0.2	1.0	0.0	0.0	14.5	0.0	0.0
WP_089610557.1	flavodoxin	3.6	1.8	0.5	2.3	0.9	0.4	16.1	4.9	0.3
WP_089610567.1	Na(+)-translocating NADH-quinone reductase subunit F	19.8	0.4	0.0	10.7	1.2	0.1	23.1	1.7	0.1
WP_242965131.1	Cell division suppressor protein YneA	24.4	1.0	0.0	16.0	0.8	0.1	62.9	5.1	0.1
WP_089610581.1	Peptidoglycan-associated lipoprotein	7.3	0.5	0.1	4.7	0.5	0.1	25.3	1.3	0.1
WP_089610608.1	hypothetical protein	2.6	1.0	0.4	1.7	0.5	0.3	19.8	0.8	0.0
WP_089610629.1	hemerythrin domain-containing protein	9.9	0.5	0.1	8.0	1.6	0.2	34.6	1.6	0.0
WP_089610630.1	cupin domain-containing protein	9.6	3.3	0.3	7.3	1.7	0.2	69.5	14.9	0.2
WP_089610635.1	NADP-dependent glyceraldehyde-3-phosphate dehydrogenase	18.5	2.0	0.1	10.7	0.9	0.1	28.7	1.6	0.1
WP_089610645.1	flavodoxin	4.0	1.4	0.4	2.3	0.5	0.2	20.5	3.4	0.2
WP_089610647.1	putative oxidoreductase/MSMEI_2347	6.8	2.5	0.4	3.7	1.2	0.3	19.1	5.2	0.3
WP_089610663.1	copper amine oxidase N-terminal domain-containing protein	7.7	0.8	0.1	6.7	0.5	0.1	51.1	2.1	0.0
WP_089610666.1	hypothetical protein	12.1	1.4	0.1	7.3	0.9	0.1	21.6	1.9	0.1
WP_089610738.1	Intracellular iron chaperone frataxin	2.0	0.0	0.0	1.0	0.0	0.0	10.0	0.0	0.0
WP_089610740.1	(2Fe-2S)-binding protein	11.1	0.7	0.1	6.0	0.8	0.1	53.5	2.5	0.0
WP_089610744.1	Transcriptional regulator SlyA	1.5	0.3	0.2	1.3	0.5	0.4	11.1	4.3	0.4
WP_089610746.1	OsmC family protein	13.6	1.7	0.1	10.0	1.4	0.1	54.2	5.0	0.1
WP_089610748.1	Dihydrolipoyl dehydrogenase	14.0	0.3	0.0	7.0	0.0	0.0	17.4	0.4	0.0
WP_089610756.1	Protein-ADP-ribose hydrolase	8.2	0.3	0.0	4.0	0.0	0.0	23.2	0.0	0.0
WP_242965141.1	Protein ADP-ribosyltransferase	2.7	1.2	0.4	1.3	0.5	0.4	13.6	4.8	0.4
WP_089610758.1	HTH-type transcriptional activator HxlR	3.0	0.9	0.3	2.0	0.0	0.0	19.6	1.5	0.1
WP_089610770.1	putative ABC transporter ATP-binding protein	2.0	0.0	0.0	1.0	0.0	0.0	3.4	0.0	0.0
WP_089610794.1	Pyridoxal kinase PdxY	3.8	1.7	0.4	2.7	0.5	0.2	13.5	1.2	0.1
WP_089610829.1	ATP-dependent zinc metalloprotease FtsH	8.6	1.5	0.2	5.0	0.8	0.2	18.2	3.4	0.2
WP_089610857.1	Anaerobic nitric oxide reductase flavorubredoxin	12.4	2.2	0.2	6.3	1.2	0.2	21.1	3.5	0.2
WP_089610859.1	Manganese efflux system protein MneP	2.6	0.1	0.0	2.0	0.0	0.0	7.8	0.0	0.0
WP_089610873.1	Ion-translocating oxidoreductase complex subunit B	11.8	0.3	0.0	6.7	0.5	0.1	23.6	1.5	0.1
WP_089610898.1	Caffeyl-CoA reductase-Etf complex subunit CarD	11.7	1.0	0.1	7.0	0.0	0.0	29.0	2.9	0.1
WP_089610900.1	Caffeyl-CoA reductase-Etf complex subunit CarE	4.7	2.2	0.5	3.3	1.2	0.4	11.7	4.2	0.4
WP_089610902.1	Electron transfer flavoprotein-ubiquinone oxidoreductase	14.1	4.7	0.3	8.0	1.6	0.2	22.6	6.2	0.3
WP_089610907.1	Nitrobenzene nitroreductase	13.6	0.9	0.1	8.3	0.5	0.1	34.5	0.0	0.0
WP_089610909.1	Benzylsuccinate synthase activating enzyme	3.0	0.9	0.3	1.7	0.5	0.3	7.1	1.5	0.2
WP_089612616.1	Trans-4-hydroxy-L-proline dehydratase	13.4	3.2	0.2	7.7	1.2	0.2	11.7	2.3	0.2
WP_089610911.1	Dihydrofolate reductase	8.2	1.7	0.2	4.7	0.5	0.1	30.7	5.2	0.2
WP_089610913.1	CDGSH iron-sulfur domain-containing protein	11.5	1.4	0.1	7.0	0.8	0.1	36.2	5.6	0.2
WP_242965156.1	CRISPR-associated protein Cas6	9.1	1.9	0.2	6.0	1.4	0.2	29.3	1.6	0.1
WP_089610948.1	Glutamyl-tRNA(Gln) amidotransferase subunit A	9.4	1.2	0.1	5.0	0.8	0.2	19.0	2.5	0.1
WP_089610950.1	Aspartyl/glutamyl-tRNA(Asn/Gln) amidotransferase subunit B	9.3	3.2	0.3	6.3	1.7	0.3	14.2	4.4	0.3
WP_242965158.1	Protein	2.4	0.6	0.3	1.3	0.5	0.4	27.7	6.5	0.2
WP_089610972.1	Uroporphyrinogen decarboxylase	19.1	3.9	0.2	15.7	0.9	0.1	38.2	9.9	0.3

WP_089608798.1	Uroporphyrinogen decarboxylase	2.1	0.5	0.2	1.3	0.5	0.4	9.2	2.8	0.3
WP_157677447.1	hypothetical protein	10.7	2.0	0.2	6.3	0.9	0.1	30.7	3.7	0.1
WP_198306530.1	DUF5050 domain-containing protein	6.5	2.6	0.4	3.7	0.9	0.3	11.6	2.9	0.2
WP_242965474.1	VOC family protein	2.0	0.0	0.0	1.3	0.5	0.4	8.5	1.6	0.2
WP_089610998.1	Tryptophan synthase alpha chain	7.2	2.9	0.4	4.7	1.2	0.3	18.8	5.8	0.3
WP_089611707.1	Tryptophan synthase beta chain	6.7	1.1	0.2	3.3	0.5	0.1	27.7	5.3	0.2
WP_089611707.1	Tryptophan synthase beta chain	10.6	1.7	0.2	7.3	0.5	0.1	22.2	3.8	0.2
WP_089610999.1	ABC transporter substrate-binding protein	1.9	0.2	0.1	1.0	0.0	0.0	4.5	0.0	0.0
WP_089611004.1	Zinc-binding GTPase YeiR	2.0	0.0	0.0	1.0	0.0	0.0	3.5	0.0	0.0
WP_089611006.1	Phospho-2-dehydro-3-deoxyheptonate aldolase, Tyr-sensitive	23.0	0.9	0.0	14.3	1.2	0.1	50.5	3.0	0.1
WP_089611018.1	Glucose 1-dehydrogenase 4	5.1	1.5	0.3	2.7	0.5	0.2	13.8	3.2	0.2
WP_089611031.1	hypothetical protein	2.6	1.3	0.5	1.7	0.5	0.3	21.2	6.8	0.3
WP_089611044.1	D-3-phosphoglycerate dehydrogenase	22.0	1.5	0.1	12.0	0.8	0.1	26.8	2.6	0.1
WP_089611048.1	phosphodiester glycosidase family protein	25.2	2.1	0.1	15.3	0.9	0.1	22.7	0.6	0.0
WP_089611052.1	sulfurtransferase-like selenium metabolism protein YedF	19.7	1.8	0.1	17.0	0.0	0.0	52.4	2.2	0.0
WP_089611054.1	DUF3343 domain-containing protein	5.8	1.7	0.3	3.0	0.8	0.3	41.3	9.9	0.2
WP_089611057.1	DNA mismatch repair protein MutS	4.7	0.6	0.1	2.7	0.5	0.2	6.5	0.9	0.1
WP_089611059.1	Exodeoxyribonuclease 7 small subunit	3.5	0.6	0.2	2.0	0.0	0.0	34.5	0.0	0.0
WP_089611064.1	1-deoxy-D-xylulose-5-phosphate synthase	1.9	0.5	0.2	1.7	0.5	0.3	3.0	0.5	0.2
WP_089611068.1	NAD kinase	2.0	0.1	0.0	1.0	0.0	0.0	3.8	0.0	0.0
WP_089611073.1	DNA repair protein RecN	1.8	0.3	0.2	1.3	0.5	0.4	4.2	1.5	0.4
WP_089612621.1	Methanol dehydrogenase activator	4.7	1.1	0.2	3.0	1.4	0.5	20.0	3.5	0.2
WP_089611093.1	Putative universal stress protein	4.7	0.5	0.1	4.3	0.5	0.1	22.3	0.0	0.0
WP_089611096.1	Phosphopentomutase	15.7	2.7	0.2	8.0	1.4	0.2	27.5	4.8	0.2
WP_089611098.1	Pyrimidine-nucleoside phosphorylase	4.1	0.3	0.1	2.7	0.5	0.2	11.5	1.3	0.1
WP_089611109.1	hypothetical protein	9.3	1.1	0.1	5.3	0.5	0.1	32.8	0.0	0.0
WP_089611131.1	A-adding tRNA nucleotidyltransferase	2.5	0.5	0.2	2.0	0.0	0.0	3.2	0.7	0.2
WP_089611136.1	Tryptophan--tRNA ligase	14.3	2.7	0.2	7.3	0.9	0.1	33.3	5.3	0.2
WP_089611147.1	D-alanyl-D-alanine carboxypeptidase DacF	12.0	1.5	0.1	6.3	0.9	0.1	22.5	2.3	0.1
WP_089611156.1	Corrinoid adenosyltransferase	12.8	2.4	0.2	7.3	0.5	0.1	35.9	7.0	0.2
WP_089611158.1	putative protein YisK	3.9	1.3	0.3	3.0	1.4	0.5	15.6	5.7	0.4
WP_089611166.1	Chorismate mutase AroH	7.7	1.7	0.2	4.3	0.5	0.1	50.1	10.9	0.2
WP_089611169.1	N-(5'-phosphoribosyl)anthranilate isomerase	5.2	2.9	0.6	2.7	0.9	0.4	16.2	6.5	0.4
WP_242965180.1	Phospho-2-dehydro-3-deoxyheptonate aldolase	5.4	1.2	0.2	3.0	0.8	0.3	15.6	4.2	0.3
WP_089611173.1	3-sulfolactaldehyde reductase	4.5	1.6	0.4	3.0	0.8	0.3	9.5	2.5	0.3
WP_089611175.1	3-phosphoshikimate 1-carboxyvinyltransferase 1	3.4	0.7	0.2	2.3	0.5	0.2	7.8	1.1	0.1
WP_089611207.1	GTPase Der	15.3	2.5	0.2	8.3	0.9	0.1	24.9	2.6	0.1
WP_089611211.1	Glycerol-3-phosphate dehydrogenase [NAD(P)+]	8.0	0.1	0.0	4.3	0.5	0.1	16.8	1.2	0.1
WP_242965182.1	Homoserine dehydrogenase	15.8	1.8	0.1	8.3	0.9	0.1	17.9	1.2	0.1
WP_089611224.1	Aspartokinase	15.2	2.3	0.1	8.7	1.2	0.1	23.0	3.0	0.1
WP_089611228.1	Transcription termination/antitermination protein NusA	12.3	1.9	0.2	7.0	1.6	0.2	25.5	4.2	0.2
WP_089611236.1	Ribosome-binding factor A	2.5	0.7	0.3	2.3	0.5	0.2	22.8	3.8	0.2
WP_157677463.1	30S ribosomal protein S15	4.4	1.9	0.4	3.0	1.4	0.5	35.2	17.0	0.5
WP_089611255.1	4-hydroxy-tetrahydrodipicolinate reductase	14.1	4.0	0.3	10.0	2.2	0.2	23.8	5.8	0.2
WP_089611262.1	Ribonuclease J1	19.0	2.4	0.1	11.7	0.5	0.0	27.8	2.4	0.1
WP_198306540.1	Protein RecA	18.7	0.7	0.0	11.7	0.5	0.0	32.2	3.2	0.1
WP_089611304.1	GTP-sensing transcriptional pleiotropic repressor CodY	11.7	1.9	0.2	6.3	0.5	0.1	24.5	2.0	0.1
WP_089611306.1	Polyisoprenyl-teichoic acid--peptidoglycan teichoic acid transferase TagU	4.2	2.0	0.5	3.3	1.2	0.4	8.9	4.5	0.5
WP_089611308.1	Diadenosine hexaphosphate hydrolase	14.1	1.0	0.1	12.0	1.4	0.1	61.8	0.0	0.0
WP_089611313.1	YlbF family regulator	11.7	2.3	0.2	10.7	1.7	0.2	76.1	6.5	0.1
WP_089611336.1	Lipopolysaccharide assembly protein B	7.5	1.3	0.2	5.3	0.5	0.1	18.3	2.3	0.1
WP_089611347.1	Alkaline shock protein 23	2.2	0.1	0.1	2.0	0.0	0.0	12.2	0.0	0.0
WP_089611365.1	hypothetical protein	4.9	1.0	0.2	5.7	0.5	0.1	14.3	0.0	0.0
WP_089611367.1	Elongation factor P	4.6	0.2	0.0	3.0	0.8	0.3	14.6	4.2	0.3
WP_089611370.1	Metalloprotease PmbA	4.7	1.1	0.2	2.3	0.5	0.2	7.7	1.7	0.2
WP_089612639.1	Metalloprotease TldD	10.8	1.1	0.1	5.7	0.5	0.1	17.5	2.0	0.1
WP_089611372.1	3-dehydroquinate synthase	2.9	0.7	0.3	1.7	0.5	0.3	5.8	1.3	0.2
WP_089611376.1	Chorismate synthase	6.0	1.9	0.3	3.7	1.2	0.3	14.8	4.6	0.3
WP_198306543.1	Type II secretion system protein G	11.6	0.9	0.1	7.0	0.0	0.0	60.2	3.3	0.1
WP_089611411.1	Carboxynorspermidine synthase	18.1	5.0	0.3	12.7	1.7	0.1	33.4	5.4	0.2
WP_089611416.1	Polyamine aminopropyltransferase	7.1	2.6	0.4	4.0	1.4	0.4	21.2	5.7	0.3
WP_242965202.1	Arginine decarboxylase	2.3	0.3	0.1	1.7	0.5	0.3	4.1	1.5	0.4
WP_089611434.1	DUF1292 domain-containing protein	5.4	0.8	0.1	4.7	0.5	0.1	42.8	3.6	0.1
WP_198306560.1	Putative pre-16S rRNA nuclease	1.9	0.3	0.2	1.0	0.0	0.0	14.9	0.0	0.0
WP_089612644.1	IreB family regulatory phosphoprotein	8.6	0.1	0.0	4.7	0.5	0.1	44.0	0.0	0.0
WP_089611525.1	Alanine--tRNA ligase	22.5	1.3	0.1	13.3	1.2	0.1	27.7	1.7	0.1
WP_089611876.1	Alanine--tRNA ligase	13.9	1.6	0.1	7.7	0.5	0.1	36.9	2.8	0.1
WP_089611487.1	Alanine--tRNA ligase	5.1	1.8	0.4	2.7	0.9	0.4	22.1	4.5	0.2
WP_089611447.1	tRNA-specific 2-thiouridylase MnmA	5.8	1.4	0.2	3.0	0.8	0.3	11.2	2.2	0.2
WP_089611452.1	Thioredoxin	7.9	1.1	0.1	10.0	0.0	0.0	37.6	0.0	0.0
WP_089611459.1	Aspartate--tRNA ligase	24.6	2.8	0.1	14.7	1.2	0.1	24.0	1.3	0.1
WP_089611461.1	Histidine--tRNA ligase	15.6	4.0	0.3	8.0	2.2	0.3	22.2	6.0	0.3
WP_089611470.1	Lipopolysaccharide assembly protein A	3.9	0.2	0.0	2.0	0.0	0.0	31.2	0.0	0.0
WP_242965207.1	Sec translocon accessory complex subunit YajC	7.6	1.2	0.2	4.3	0.5	0.1	39.9	9.3	0.2
WP_089611472.1	Queuine tRNA-ribosyltransferase	7.8	0.8	0.1	4.3	0.5	0.1	18.2	1.0	0.1
WP_089611479.1	anti-sigma factor domain-containing protein	8.0	4.3	0.5	4.7	1.7	0.4	10.0	3.5	0.4
WP_089611492.1	putative transcriptional regulatory protein	3.0	0.3	0.1	3.0	0.0	0.0	8.8	0.0	0.0
WP_089611502.1	Biosynthetic peptidoglycan transglycosylase	8.9	2.1	0.2	5.0	0.8	0.2	9.2	2.3	0.3
WP_089611507.1	NGG1p interacting factor NIF3	6.8	1.7	0.3	3.7	0.9	0.3	17.1	4.3	0.3
WP_089611514.1	Phenylalanine--tRNA ligase alpha subunit	9.4	1.2	0.1	5.3	0.5	0.1	19.4	1.2	0.1
WP_089611518.1	23S rRNA (guanosine-2'-O-)-methyltransferase RlmB	2.0	0.0	0.0	1.3	0.5	0.4	6.9	0.0	0.0
WP_089611523.1	Translation initiation factor IF-3	11.9	0.1	0.0	6.0	0.0	0.0	44.5	0.0	0.0
WP_089611534.1	Pyrophosphate--fructose 6-phosphate 1-phosphotransferase	3.3	0.8	0.2	2.0	0.0	0.0	6.1	0.7	0.1
WP_089611536.1	NAD(P)H-dependent FMN reductase	4.6	1.9	0.4	2.7	1.2	0.5	18.6	10.2	0.6
WP_089611540.1	Putative universal stress protein	4.0	1.1	0.3	3.0	0.8	0.3	23.2	6.4	0.3
WP_089611542.1	Putative universal stress protein	14.0	1.4	0.1	9.0	0.0	0.0	54.2	4.2	0.1
WP_089611544.1	Putative universal stress protein	22.1	2.3	0.1	14.0	0.8	0.1	76.8	4.3	0.1
WP_089611548.1	N-substituted formamide deformylase	11.4	6.6	0.6	8.7	4.2	0.5	16.0	7.4	0.5
WP_089612649.1	Ferredoxin--NADP reductase	16.7	1.0	0.1	9.7	1.2	0.1	9.3	1.5	0.2
WP_089611555.1	UDP-2-acetamido-2,6-beta-L-arabino-hexul-4-ose reductase	23.2	1.6	0.1	14.3	1.2	0.1	32.2	2.1	0.1
WP_089611560.1	hypothetical protein	2.0	0.0	0.0	1.0	0.0	0.0	9.6	0.0	0.0
WP_089611562.1	GatB/YqeY domain-containing protein	10.1	1.8	0.2	6.0	0.8	0.1	47.8	3.3	0.1
WP_089611573.1	Tyrosine-protein kinase YwqD	3.5	1.3	0.4	2.3	0.5	0.2	13.4	2.7	0.2
WP_089611575.1	putative capsular polysaccharide biosynthesis protein YwqC	10.0	0.1	0.0	5.0	0.0	0.0	29.2	0.0	0.0
WP_089611580.1	Ribonuclease BN	2.0	0.1	0.0	1.0	0.0	0.0	3.6	0.0	0.0
WP_089611582.1	NADPH-dependent 7-cyano-7-deazaguanine reductase	3.2	2.4	0.8	1.7	0.9	0.6	12.7	9.7	0.8
WP_089611585.1	MBL fold metallo-hydrolase	2.7	1.2	0.4	1.3	0.5	0.4	9.1	6.2	0.7
WP_089611594.1	Lactate utilization protein A	3.6	1.0	0.3	2.3	0.5	0.2	6.7	1.9	0.3
WP_089611596.1	putative FAD-linked oxidoreductase	5.9	2.2	0.4	4.0	0.8	0.2	9.7	3.8	0.4
WP_089611600.1	Fructose-1,6-bisphosphatase class 3	13.7	5.8	0.4	7.3	2.6	0.4	16.2	5.7	0.4
WP_198306549.1	Peptide methionine sulfoxide reductase MsrA/MsrB	6.5	1.9	0.3	3.7	1.2	0.3	19.2	5.7	0.3
WP_089611698.1	Long-chain-fatty-acid--CoA ligase FadD13	25.4	3.3	0.1	15.3	0.9	0.1	29.2	3.2	0.1
WP_089611700.1	Gamma-glutamyl phosphate reductase	17.7	2.8	0.2	12.3	1.2	0.1	33.9	5.1	0.2
WP_089611702.1	Glutamate 5-kinase	12.6	2.7	0.2	7.0	0.8	0.1	21.3	1.4	0.1
WP_089611703.1	Immunoglobin (Ig)-like domain-containing protein	61.5	5.7	0.1	43.3	5.6	0.1	48.2	2.9	0.1
WP_089611709.1	Ion-translocating oxidoreductase complex subunit B	21.0	2.4	0.1	12.0	1.4	0.1	53.0	2.3	0.0
WP_089611726.1	D-inositol-3-phosphate glycosyltransferase	1.9	0.5	0.3	1.3	0.5	0.4	5.6	1.6	0.3
WP_089611728.1	N-acetyl-alpha-D-glucosaminyl L-malate deacetylase 1	8.7	4.0	0.5	6.0	1.4	0.2	31.4	6.1	0.2
WP_089611754.1	DNA polymerase I	5.2	2.4	0.5	3.0	0.8	0.3	5.6	1.2	0.2
WP_089611763.1	DUF177 domain-containing protein	13.4	0.4	0.0	8.0	0.0	0.0	47.9	5.3	0.1
WP_089611769.1	hypothetical protein	21.1	0.8	0.0	15.0	0.8	0.1	67.3	3.2	0.0
WP_089611788.1	putative protein	6.9	2.3	0.3	4.7	1.7	0.4	12.5	5.8	0.5
WP_089612657.1	putative protein	2.6	0.5	0.2	1.7	0.5	0.3	19.6	4.4	0.2
WP_089611812.1	Elongation factor G	58.9	1.8	0.0	35.7	1.7	0.0	48.1	1.3	0.0
WP_089611816.1	5'-nucleotidase SurE	3.9	1.8	0.5	2.0	0.8	0.4	13.6	5.7	0.4
WP_089611818.1	YpmA family protein	2.7	1.2	0.4	1.3	0.5	0.4	19.0	2.9	0.2
WP_089611820.1	GTP cyclohydrolase 1	14.4	1.5	0.1	8.0	0.0	0.0	46.3	1.0	0.0
WP_089611822.1	7-carboxy-7-deazaguanine synthase	2.1	0.1	0.0	1.0	0.0	0.0	6.9	0.0	0.0
WP_089611830.1	Phosphatidylserine decarboxylase proenzyme	1.9	0.2	0.1	1.0	0.0	0.0	4.7	0.0	0.0
WP_089611833.1	2-oxoglutarate carboxylase large subunit	6.3	0.5	0.1	7.0	1.4	0.2	50.8	0.0	0.0
WP_089611837.1	Propionyl-CoA carboxylase beta chain	14.5	3.2	0.2	7.7	1.7	0.2	22.3	4.1	0.2
WP_089611839.1	Adenosylhomocysteinase	14.9	3.8	0.3	9.3	1.9	0.2	24.5	6.1	0.2
WP_157677486.1	Ribonuclease	9.2	2.8	0.3	4.7	1.2	0.3	12.3	3.8	0.3
WP_242965235.1	DNA-binding transcriptional activator DecR	3.7	0.6	0.2	2.3	0.5	0.2	16.3	2.7	0.2
WP_089611857.1	5-methyltetrahydrofolate:corrinoid/iron-sulfur protein co-methyltransferase	20.8	0.5	0.0	20.3	1.7	0.1	38.4	0.9	0.0
WP_089611859.1	Corrinoid/iron-sulfur protein small subunit	22.1	0.6	0.0	12.0	0.8	0.1	39.5	3.4	0.1
WP_089611872.1	hypothetical protein	8.0	2.0	0.2	4.3	0.9	0.2	24.4	5.7	0.2
WP_089611874.1	hypothetical protein	16.8	1.1	0.1	10.0	0.8	0.1	58.0	2.5	0.0
WP_089611876.1	Iron-sulfur cluster repair protein ScdA	3.2	1.2	0.4	1.7	0.5	0.3	30.6	11.1	0.4
WP_089611877.1	hypothetical protein	12.0	2.1	0.2	8.7	1.2	0.1	47.9	4.4	0.1
WP_089611879.1	Protein-L-isoaspartate O-methyltransferase	3.7	1.4	0.4	2.0	0.8	0.4	12.6	0.5	0.0
WP_089611880.1	Tyrosine--tRNA ligase	12.6	3.9	0.3	6.7	1.2	0.2	20.3	3.8	0.2
WP_089611882.1	NifB/NifX family molybdenum-iron cluster-binding protein	12.4	4.5	0.4	16.0	2.8	0.2	61.2	4.2	0.1
WP_089612660.1	DUF4153 domain-containing protein	5.7	2.0	0.3	3.3	0.5	0.1	9.2	1.7	0.2
WP_089611883.1	Copper chaperone CopZ	3.5	0.8	0.2	2.0	0.0	0.0	37.3	0.0	0.0
WP_089611893.1	Cysteine synthase	15.8	1.4	0.1	9.3	0.9	0.1	40.8	4.2	0.1
WP_242965240.1	Tol-Pal system protein TolB	11.3	3.6	0.3	6.3	1.2	0.2	25.9	6.9	0.3
WP_242965242.1	Glycogen phosphorylase	8.8	2.0	0.2	5.0	0.8	0.2	8.2	1.2	0.1
WP_089611895.1	Putative niacin/nicotinamide transporter NaiP	13.8	1.2	0.1	10.3	0.9	0.1	19.7	2.8	0.1
WP_242965244.1	Indole-3-glycerol phosphate synthase	6.0	2.0	0.3	3.0	0.8	0.3	14.5	2.6	0.2
WP_089611897.1	Anthranilate phosphoribosyltransferase	8.0	2.0	0.2	4.0	0.8	0.2	13.6	2.4	0.2
WP_089611898.1	Anthranilate synthase component 2	3.1	0.1	0.0	2.3	0.5	0.2	12.2	2.7	0.2
WP_089612664.1	Anthranilate synthase component 1	16.3	1.8	0.1	8.7	0.9	0.1	26.2	2.3	0.1
WP_157677490.1	primosomal protein N'	3.2	1.2	0.4	2.3	0.5	0.2	6.5	1.5	0.2
WP_089611904.1	Hydroxylamine reductase	26.0	5.6	0.2	17.7	3.3	0.2	41.6	7.5	0.2

WP_089611908.1	AbrB/MazE/SpoVT family DNA-binding domain-containing protein	1.7	0.3	0.2	1.0	0.0	0.0	15.4	0.0	0.0
WP_242965256.1	virulence RhuM family protein	8.6	1.7	0.2	4.3	0.5	0.1	18.4	1.7	0.1
WP_089611953.1	DUF1232 domain-containing protein	3.0	1.5	0.5	2.3	0.5	0.2	15.8	1.7	0.1
WP_089611954.1	UDP-N-acetylglucosamine 4-epimerase	13.4	0.7	0.1	6.7	0.5	0.1	23.6	1.5	0.1
WP_089611955.1	UDP-glucose 6-dehydrogenase YwqF	6.9	2.8	0.4	4.0	1.4	0.4	14.8	7.7	0.5
WP_089611956.1	DNA replication and repair protein RecF	4.1	2.5	0.6	3.0	1.6	0.5	5.1	2.5	0.5
WP_089611965.1	3-oxoacyl-[acyl-carrier-protein] synthase 3	3.4	1.4	0.4	2.7	0.9	0.4	8.3	3.5	0.4
WP_089611966.1	3-oxoacyl-[acyl-carrier-protein] synthase 3	6.5	0.8	0.1	4.0	0.0	0.0	17.0	0.6	0.0
WP_089611969.1	Putative pyridoxal phosphate-dependent aminotransferase EpsN	15.3	0.4	0.0	8.0	0.8	0.1	24.8	2.2	0.1
WP_089611971.1	HTH-type transcriptional repressor RspR	7.0	1.2	0.2	3.7	0.5	0.1	21.3	2.7	0.1
WP_089611973.1	Glutamate-1-semialdehyde 2,1-aminomutase	26.0	9.1	0.4	19.3	6.0	0.3	41.9	13.5	0.3
WP_089611975.1	Delta-aminolevulinic acid dehydratase	13.1	6.3	0.5	7.3	2.6	0.4	27.4	11.5	0.4
WP_089611976.1	Porphobilinogen deaminase	9.2	1.0	0.1	4.7	0.5	0.1	19.8	2.3	0.1
WP_089611978.1	dTDP-4-dehydrorhamnose 3,5-epimerase family protein	2.8	1.0	0.4	1.7	0.5	0.3	12.8	2.4	0.2
WP_089611981.1	UDP-glucose 4-epimerase	5.2	2.7	0.5	2.7	1.2	0.5	12.6	5.0	0.4
WP_089611982.1	vitamin B12 dependent methionine synthase	20.5	1.3	0.1	10.7	0.5	0.0	42.2	3.2	0.1
WP_089611984.1	GerMN domain-containing protein	10.6	1.2	0.1	5.3	0.5	0.1	23.5	3.3	0.1
WP_089611985.1	N-acetylmuramoyl-L-alanine amidase	15.2	1.9	0.1	7.7	0.5	0.1	19.5	1.8	0.1
WP_242965258.1	Aspartate-semialdehyde dehydrogenase 2	14.0	4.4	0.3	10.3	2.5	0.2	32.5	7.8	0.2
WP_089611988.1	Nigerythrin	16.2	1.6	0.1	9.3	0.9	0.1	51.2	0.0	0.0
WP_089612052.1	transposase	1.9	0.1	0.1	1.0	0.0	0.0	5.9	0.6	0.1
WP_089612055.1	GrdX family protein	4.8	1.3	0.3	3.0	0.0	0.0	32.0	4.4	0.1
WP_089612059.1	Transcriptional regulatory protein SrrA	3.5	2.9	0.8	2.7	0.9	0.4	8.8	4.6	0.5
WP_089612061.1	tripartite tricarboxylate transporter substrate binding protein	2.0	0.0	0.0	1.0	0.0	0.0	3.3	0.0	0.0
WP_089612069.1	hypothetical protein	2.5	1.4	0.6	1.3	0.5	0.4	13.3	4.4	0.3
WP_089612070.1	Bifunctional protein FolD protein	22.2	0.9	0.0	15.7	0.9	0.1	42.7	0.0	0.0
WP_198306571.1	hypothetical protein DCMF_01805	2.8	0.7	0.2	1.7	0.5	0.3	4.0	1.0	0.3
WP_242965274.1	Oxaloacetate decarboxylase alpha chain	14.1	1.7	0.1	8.3	1.7	0.2	19.3	4.5	0.2
WP_089612087.1	pirin family protein	10.6	1.9	0.2	7.0	0.8	0.1	25.9	3.6	0.1
WP_089612092.1	sporulation protein	14.3	2.7	0.2	10.3	0.9	0.1	57.1	3.2	0.1
WP_089612100.1	DUF4317 domain-containing protein	1.8	0.3	0.2	1.0	0.0	0.0	2.8	0.0	0.0
WP_089612101.1	Putative NAD(P)H nitroreductase YfkO	8.3	1.8	0.2	4.7	0.9	0.2	22.5	5.1	0.2
WP_089612103.1	2,3-diketo-5-methylthiopentyl-1-phosphate enolase	11.4	3.1	0.3	6.0	1.6	0.3	21.8	5.7	0.3
WP_089612106.1	Light-independent protochlorophyllide reductase subunit B	8.7	1.9	0.2	4.7	0.9	0.2	15.6	3.8	0.2
WP_089612107.1	Nitrogenase iron protein	14.2	3.1	0.2	6.7	1.2	0.2	14.2	3.4	0.2
WP_089612108.1	hypothetical protein	7.4	2.3	0.3	4.0	0.8	0.2	13.1	3.0	0.2
WP_198306574.1	Copper-exporting P-type ATPase B	3.6	2.0	0.6	3.3	1.2	0.4	6.3	2.1	0.3
WP_089612130.1	Flavoredoxin	5.6	0.6	0.1	3.3	0.5	0.1	23.3	0.0	0.0
WP_089612134.1	Uroporphyrinogen decarboxylase	23.8	1.2	0.0	15.3	1.2	0.1	46.6	0.0	0.0
WP_089612136.1	Choline trimethylamine-lyase	7.1	4.8	0.7	3.7	2.4	0.6	7.5	4.7	0.6
WP_089612141.1	Uridine kinase	5.0	2.5	0.5	3.7	2.1	0.6	10.6	5.3	0.5
WP_089612145.1	putative protein	3.6	0.2	0.1	2.0	0.0	0.0	22.5	0.0	0.0
WP_089612147.1	Lactoylglutathione lyase	4.3	0.3	0.1	2.7	0.5	0.2	22.4	6.2	0.3
WP_089612148.1	Putative universal stress protein	11.0	1.3	0.1	6.0	0.0	0.0	49.2	0.6	0.0
WP_089612151.1	Bifunctional F420 biosynthesis protein FbiB	4.7	1.2	0.2	3.7	0.5	0.1	19.0	2.7	0.1
WP_198306577.1	Lipoate-protein ligase A	6.5	1.2	0.2	4.0	0.8	0.2	34.2	7.3	0.2
WP_089612161.1	Long-chain-alcohol dehydrogenase 2	16.4	2.5	0.2	9.0	1.6	0.2	27.3	5.9	0.2
WP_089612164.1	CGGC domain-containing protein	2.0	0.0	0.0	1.3	0.5	0.4	13.6	0.0	0.0
WP_089612165.1	putative protein	2.6	1.0	0.4	1.3	0.5	0.4	7.4	2.3	0.3
WP_242965295.1	RibD family protein	3.0	1.2	0.4	1.7	0.5	0.3	13.4	3.9	0.3
WP_089612170.1	Flavoredoxin	6.6	2.4	0.4	3.3	0.9	0.3	24.5	5.5	0.2
WP_089612218.1	site-specific DNA-methyltransferase	10.4	3.6	0.3	6.3	1.2	0.2	10.9	3.2	0.3
WP_089609716.1	(S)-2-haloacid dehalogenase	17.0	1.1	0.1	12.7	2.4	0.2	41.6	4.3	0.1
WP_242965203.1	2-hydroxychromene-2-carboxylate isomerase	6.8	2.5	0.4	5.3	1.2	0.2	20.3	5.9	0.3
WP_089611960.1	MULTISPECIES: pyridoxamine 5'-phosphate oxidase family protein	14.5	2.2	0.2	13.3	0.9	0.1	73.4	14.0	0.2
WP_089610557.1	hypothetical protein C1O63_0543	3.8	0.3	0.1	2.0	0.0	0.0	15.3	0.0	0.0
WP_089608534.1	MULTISPECIES: metal-binding protein	3.6	0.4	0.1	2.0	0.0	0.0	31.9	0.0	0.0
WP_089612224.1	hypothetical protein	3.4	0.7	0.2	2.7	0.5	0.2	11.7	2.7	0.2
WP_089612226.1	helix-turn-helix transcriptional regulator	2.2	0.3	0.2	1.3	0.5	0.4	23.0	5.7	0.2
WP_198306586.1	Aspartyl/glutamyl-tRNA(Asn/Gln) amidotransferase subunit B	25.1	2.3	0.1	12.3	1.2	0.1	30.3	3.0	0.1
WP_089612238.1	Glutamyl-tRNA(Gln) amidotransferase subunit A	26.9	1.0	0.0	14.3	0.9	0.1	39.5	2.6	0.1
WP_089612239.1	Aspartyl/glutamyl-tRNA(Asn/Gln) amidotransferase subunit C	2.0	0.0	0.0	1.3	0.5	0.4	22.7	6.5	0.3
WP_089612240.1	D-gamma-glutamyl-meso-diaminopimelic acid endopeptidase CwlS	24.7	0.9	0.0	19.3	0.5	0.0	53.2	2.4	0.0
WP_089612242.1	3'-5' exoribonuclease YhaM	2.0	0.0	0.0	1.0	0.0	0.0	3.7	0.0	0.0
WP_089612248.1	universal stress protein	4.0	0.0	0.0	2.3	0.5	0.2	24.3	0.0	0.0
WP_089612249.1	DNA ligase	10.2	2.8	0.3	5.0	1.4	0.3	11.7	2.7	0.2
WP_089610042.1	Phosphoribosyl-ATP pyrophosphatase	6.0	0.0	0.0	3.0	0.0	0.0	30.9	0.0	0.0
WP_089612253.1	Protein YiiM	20.7	1.2	0.1	10.7	0.9	0.1	71.2	1.0	0.0
WP_089612254.1	Molybdopterin-guanine dinucleotide biosynthesis adapter protein	4.9	1.4	0.3	3.0	0.8	0.3	22.6	4.6	0.2
WP_089612255.1	GTP 3',8-cyclase	3.7	0.8	0.2	1.7	0.5	0.3	6.5	1.3	0.2
WP_089612256.1	Molybdopterin molybdenumtransferase	25.7	0.9	0.0	14.3	1.2	0.1	41.9	2.4	0.1
WP_089612257.1	hypothetical protein	3.7	1.3	0.4	2.0	0.8	0.4	36.8	14.1	0.4
WP_089612689.1	hypothetical protein	11.7	0.4	0.0	10.0	1.4	0.1	49.3	8.1	0.2
WP_242965496.1	Imidazole glycerol phosphate synthase subunit HisF	22.6	4.1	0.2	12.3	1.7	0.1	40.8	2.0	0.0
WP_089612262.1	1-(5-phosphoribosyl)-5-[(5-phosphoribosylamino)methylideneamino] imidazole-4-carboxamide isomerase	14.7	3.4	0.2	8.3	0.9	0.1	45.1	8.4	0.2
WP_089612263.1	Imidazole glycerol phosphate synthase subunit HisH	2.0	0.0	0.0	1.0	0.0	0.0	6.6	0.0	0.0
WP_242965498.1	Histidinol dehydrogenase	10.2	1.8	0.2	5.3	0.5	0.1	19.3	0.4	0.0
WP_089612691.1	ATP phosphoribosyltransferase	17.5	2.8	0.2	11.7	1.7	0.1	33.2	3.2	0.1
WP_089612265.1	ATP phosphoribosyltransferase regulatory subunit	8.5	2.4	0.3	4.7	1.2	0.3	15.6	3.0	0.2
WP_089608934.1	IS21 family transposase	2.6	1.1	0.4	1.3	0.5	0.4	3.9	1.7	0.4
WP_089608935.1	IS21 family transposase ISHahy12	9.6	1.9	0.2	5.0	0.8	0.2	27.3	4.8	0.2
WP_089612280.1	type I CRISPR-associated protein Cas7	3.1	1.8	0.6	1.7	0.9	0.6	7.0	3.4	0.5
WP_089612293.1	hypothetical protein	5.1	1.1	0.2	4.0	0.8	0.2	48.7	6.8	0.1
WP_089612692.1	TrpR-like protein YerC/YecD	2.5	1.3	0.5	1.3	0.5	0.4	14.4	5.1	0.4
WP_089612294.1	Lipoate-protein ligase A	13.5	1.8	0.1	6.7	0.9	0.1	24.7	0.7	0.0
WP_089612295.1	Dihydrolipoyl dehydrogenase	20.4	2.2	0.1	11.7	1.2	0.1	32.0	4.2	0.1
WP_089612693.1	Phosphoribosylamine--glycine ligase	7.5	3.1	0.4	5.0	1.4	0.3	16.1	6.9	0.4
WP_089612300.1	Bifunctional purine biosynthesis protein PurH	16.3	4.1	0.2	8.3	1.7	0.2	22.2	5.4	0.2
WP_089612302.1	Phosphoribosylformylglycinamidine cyclo-ligase	6.8	1.9	0.3	3.3	0.9	0.3	16.0	4.0	0.3
WP_089612303.1	Amidophosphoribosyltransferase	12.1	2.4	0.2	6.3	1.2	0.2	18.2	3.5	0.2
WP_089612304.1	Adenylosuccinate lyase	12.0	1.4	0.1	7.0	0.8	0.1	17.8	2.4	0.1
WP_089612305.1	N5-carboxyaminoimidazole ribonucleotide mutase	4.8	1.9	0.4	4.3	1.2	0.3	21.0	7.2	0.3
WP_089612313.1	hemerythrin domain-containing protein	7.1	2.3	0.3	4.0	0.8	0.2	28.5	9.7	0.3
WP_089612314.1	UDP-N-acetylenolpyruvoylglucosamine reductase	4.2	1.3	0.3	2.7	0.9	0.4	10.1	3.9	0.4
WP_198306589.1	tandem-95 repeat protein	4.6	1.9	0.4	5.3	2.5	0.5	15.2	4.7	0.3
WP_089612326.1	Desulfoferrodoxin	12.4	1.8	0.1	10.7	1.2	0.1	73.0	10.4	0.1
WP_089612327.1	Nitric oxide reductase	29.2	2.4	0.1	19.7	2.1	0.1	47.3	2.5	0.1
WP_089612328.1	High molecular weight rubredoxin	12.1	2.3	0.2	10.0	2.2	0.2	29.8	4.0	0.1
WP_089612329.1	Cobalt-precorrin-6A reductase	5.5	1.7	0.3	3.0	0.8	0.3	15.8	5.4	0.3
WP_089612330.1	Cobalt-factor III methyltransferase	17.4	2.7	0.2	13.0	0.8	0.1	41.3	9.5	0.2
WP_089612331.1	Cobalt-precorrin-5A hydrolase	3.5	2.3	0.7	2.0	0.8	0.4	13.3	5.8	0.4
WP_089612333.1	Cobalt-precorrin-4 C(11)-methyltransferase	12.5	3.6	0.3	12.0	1.4	0.1	36.8	9.3	0.3
WP_157677516.1	precorrin-2 C(20)-methyltransferase	4.0	0.0	0.0	2.0	0.0	0.0	42.9	0.0	0.0
WP_089612334.1	Cobalt-precorrin-2 C(20)-methyltransferase	18.5	1.2	0.1	12.3	1.2	0.1	61.4	0.8	0.0
WP_089612335.1	Cobalt-precorrin-6B C(15)-methyltransferase (decarboxylating)	9.5	4.5	0.5	5.0	1.6	0.3	36.6	14.6	0.4
WP_089612336.1	Cobalt-precorrin-7 C(5)-methyltransferase	11.4	1.1	0.1	7.0	0.8	0.1	22.0	4.7	0.2
WP_242965316.1	Cobalt-precorrin-5B C(1)-methyltransferase	20.1	2.5	0.1	10.3	1.2	0.1	32.7	3.6	0.1
WP_089612337.1	Cobalt-precorrin-8 methylmutase	17.5	3.0	0.2	17.0	3.6	0.2	52.9	4.7	0.1
WP_089612338.1	Cobyrinate a,c-diamide synthase	16.2	1.9	0.1	9.0	0.8	0.1	27.3	3.4	0.1
WP_089612340.1	Cobalamin biosynthesis protein CobD	2.4	0.3	0.1	2.3	0.5	0.2	7.6	0.4	0.1
WP_089612342.1	Adenosylcobalamin/alpha-ribazole phosphatase	8.8	1.3	0.1	4.7	0.5	0.1	27.5	1.4	0.1
WP_089612696.1	Bifunctional adenosylcobalamin biosynthesis protein CobU	4.4	0.3	0.1	2.3	0.5	0.2	20.1	1.8	0.1
WP_089612344.1	Succinyl-diaminopimelate desuccinylase	3.3	2.2	0.7	2.3	0.5	0.2	8.1	2.0	0.2
WP_089612353.1	Methionine synthase	3.9	0.2	0.1	36.0	3.6	0.1	68.8	4.8	0.1
WP_089612359.1	Glutamyl-tRNA reductase	1.7	0.2	0.1	1.3	0.5	0.4	2.9	1.0	0.4
WP_089612360.1	Sirohydrochlorin cobaltochelatase	5.0	1.7	0.3	4.0	1.4	0.4	39.7	7.0	0.2
WP_089612361.1	Phosphoribosylaminoimidazole-succinocarboxamide synthase	24.2	2.8	0.1	17.3	0.9	0.1	61.4	3.9	0.1
WP_089612364.1	Hydrogenase-4 component B	2.0	0.1	0.0	1.0	0.0	0.0	2.1	0.0	0.0
WP_089612370.1	pyridoxamine 5'-phosphate oxidase family protein	23.2	5.9	0.3	23.0	2.8	0.1	80.7	17.2	0.2
WP_198306574.1	Copper-exporting P-type ATPase	5.3	1.4	0.3	3.0	0.8	0.3	6.9	1.9	0.3
WP_089612380.1	Putative efflux system component YknX	3.2	0.8	0.2	1.7	0.5	0.3	5.8	1.4	0.2
WP_089612384.1	O-acetylserine sulfhydrylase	22.8	4.0	0.2	14.0	1.6	0.1	46.3	6.3	0.1
WP_089612385.1	O-acetyl-L-homoserine sulfhydrylase	18.9	5.8	0.3	11.3	2.1	0.2	31.7	7.0	0.2
WP_089612386.1	Iron-sulfur cluster assembly scaffold protein IscU	7.1	1.3	0.2	5.0	1.4	0.3	51.8	11.5	0.2
WP_089612387.1	Cysteine desulfurase IscS	13.1	1.7	0.1	7.3	1.2	0.2	30.2	4.1	0.1
WP_089612388.1	Homoserine O-acetyltransferase	6.9	1.0	0.1	3.3	0.5	0.1	13.7	2.4	0.2
WP_157677530.1	hypothetical protein	8.0	1.2	0.1	5.3	1.2	0.2	25.4	4.0	0.2
WP_089612395.1	Lactoylglutathione lyase	9.1	1.0	0.1	6.3	0.5	0.1	45.8	0.0	0.0
WP_089612396.1	Ubiquinone biosynthesis O-methyltransferase, mitochondrial	15.8	1.2	0.1	8.7	0.9	0.1	41.3	1.8	0.0
WP_089612398.1	S-layer homology domain-containing protein	25.1	2.0	0.1	18.7	2.1	0.1	36.1	6.3	0.2
WP_089612399.1	Putative HMP/thiamine import ATP-binding protein YkoD	10.5	2.6	0.2	5.7	0.5	0.1	14.7	1.7	0.1
WP_089612400.1	Energy-coupling factor transporter transmembrane protein EcfT	2.0	0.0	0.0	1.0	0.0	0.0	3.6	0.0	0.0
WP_198306590.1	S-layer homology domain-containing protein	78.6	7.7	0.1	62.7	6.2	0.1	50.4	4.7	0.1
WP_089612404.1	Cobalt transport protein CbiM	2.0	0.0	0.0	1.7	0.5	0.3	7.0	2.0	0.3
WP_089612407.1	Protein translocase subunit SecF	9.6	0.4	0.0	10.0	0.8	0.1	16.9	0.0	0.0
WP_089612411.1	Energy-coupling factor transporter ATP-binding protein EcfA3	2.0	0.0	0.0	1.0	0.0	0.0	4.2	0.0	0.0
WP_089612413.1	adenosylcobinamide amidohydrolase	13.1	3.1	0.2	7.7	1.7	0.2	40.6	0.9	0.0
WP_089612700.1	vitamin B12-dependent ribonucleotide reductase	13.2	1.2	0.1	7.3	0.9	0.1	11.8	1.5	0.1
WP_089612701.1	hypothetical protein	7.3	0.2	0.0	4.0	0.0	0.0	42.3	0.0	0.0
WP_242965323.1	DUF1847 domain-containing protein	16.5	2.7	0.2	10.0	1.4	0.1	51.4	9.6	0.2
WP_089612415.1	Phenylacetate-coenzyme A ligase	18.5	3.3	0.2	13.0	2.2	0.2	29.2	4.9	0.2
WP_089612419.1	Menaquinone reductase, iron-sulfur cluster-binding subunit	4.6	1.6	0.3	2.7	0.5	0.2	14.0	2.1	0.1
WP_089612421.1	Lactate utilization protein A	3.1	1.0	0.3	2.0	0.8	0.4	4.4	2.1	0.5
WP_089612704.1	(Fe-S)-binding protein	12.6	2.7	0.2	7.0	0.8	0.1	15.2	2.8	0.2
WP_089612426.1	Coenzyme A disulfide reductase	24.8	1.1	0.0	17.0	2.2	0.1	30.0	1.5	0.1

WP_089612429.1	hypothetical protein	3.9	1.2	0.3	3.3	0.9	0.3	27.2	7.3	0.3
WP_089612433.1	Protein LemA	7.0	1.4	0.2	5.0	0.8	0.2	26.0	7.6	0.3

Table S9. Protein assignments to 22 KEGG categories using BlastKOALA

KEGG Functional Category	Category Code	Raw number	% out of total annotated (702)	% out of proteins in proteome (1,012)
Genetic information processing	9120	115	16.4	11.4
Amino acid metabolism	9105	97	13.8	9.6
Energy metabolism	9102	90	12.8	8.9
Metabolism of cofactors and vitamins	9108	84	12.0	8.3
Carbohydrate metabolism	9101	80	11.4	7.9
Protein families: genetic information processing	9182	58	8.3	5.7
Environmental information processing	9130	49	7.0	4.8
Unclassified: metabolism	9191	38	5.4	3.8
Nucleotide metabolism	9104	36	5.1	3.6
Human diseases	9160	34	4.8	3.4
Protein families: signalling and cellular processing	9183	33	4.7	3.3
Cellular processes	9140	33	4.7	3.3
Metabolism of other amino acids	9106	23	3.3	2.3
Glycan biosynthesis and metabolism	9107	22	3.1	2.2
Xenobiotics degradation and metabolism	9111	22	3.1	2.2
Organismal systems	9150	20	2.8	2.0
Unclassified		19	2.7	1.9
Protein families: metabolism	9181	18	2.6	1.8
Unclassified: genetic information processing	9192	12	1.7	1.2
Unclassified: signalling and cellular processing	9193	11	1.6	1.1
Lipid metabolism	9103	10	1.4	1.0
Metabolism of terpenoids and polyketides	9109	4	0.6	0.4

Table S10. List of proteins identified from *D. formicoaceticum* proteome assigned to 22 KEGG functional categories using BlastKOALA

Query_GTDB	Qurey_Prokka	Best Hit	KEGG Functional Category	Category Code	Hit Description	Score
NZ_CP022121.1_2554	JBMCGNCK_02618	K00003	Amino acid metabolism	9105	hom; homoserine dehydrogenase [EC:1.1.1.3]	231
NZ_CP022121.1_3424	JBMCGNCK_03511	K00013	Amino acid metabolism	9105	hisD; histidinol dehydrogenase [EC:1.1.1.23]	306
NZ_CP022121.1_3067	JBMCGNCK_03149	K00133	Amino acid metabolism	9105	asd; aspartate-semialdehyde dehydrogenase [EC:1.2.1.11]	243
NZ_CP022121.1_1158	JBMCGNCK_01199	K00145	Amino acid metabolism	9105	argC; N-acetyl-gamma-glutamyl-phosphate reductase [EC:1.2.1.38]	201
NZ_CP022121.1_2826	JBMCGNCK_02898	K00147	Amino acid metabolism	9105	proA; glutamate-5-semialdehyde dehydrogenase [EC:1.2.1.41]	255
NZ_CP022121.1_2572	JBMCGNCK_02637	K00215	Amino acid metabolism	9105	dapB; 4-hydroxy-tetrahydrodipicolinate reductase [EC:1.17.1.8]	152
NZ_CP022121.1_1730	JBMCGNCK_01783	K00286	Amino acid metabolism	9105	proC; pyrroline-5-carboxylate reductase [EC:1.5.1.2]	116
NZ_CP022121.1_2663	JBMCGNCK_02728	K00290	Amino acid metabolism	9105	LYS1; saccharopine dehydrogenase (NAD+, L-lysine forming) [EC:1.5.1.7]	343
NZ_CP022121.1_415	JBMCGNCK_00425	K00294	Amino acid metabolism	9105	E1.2.1.88; 1-pyrroline-5-carboxylate dehydrogenase [EC:1.2.1.88]	346
NZ_CP022121.1_736	JBMCGNCK_00758	K00611	Amino acid metabolism	9105	OTC, argF, argI; ornithine carbamoyltransferase [EC:2.1.3.3]	252
NZ_CP022121.1_1160	JBMCGNCK_01200	K00620	Amino acid metabolism	9105	gJ; glutamate N-acetyltransferase / amino-acid N-acetyltransferase [EC:2.3.1.35 2.3.1.	251
NZ_CP022121.1_3425	JBMCGNCK_03512	K00765	Amino acid metabolism	9105	hisG; ATP phosphoribosyltransferase [EC:2.4.2.17]	136
NZ_CP022121.1_2963	JBMCGNCK_03043	K00766	Amino acid metabolism	9105	trpD; anthranilate phosphoribosyltransferase [EC:2.4.2.18]	230
NZ_CP022121.1_1374	JBMCGNCK_01425	K00772	Amino acid metabolism	9105	mtaP, MTAP; 5'-methylthioadenosine phosphorylase [EC:2.4.2.28]	218
NZ_CP022121.1_1802	JBMCGNCK_01856	K00789	Amino acid metabolism	9105	metK, MAT; S-adenosylmethionine synthetase [EC:2.5.1.6]	302
NZ_CP022121.1_2531	JBMCGNCK_02595	K00800	Amino acid metabolism	9105	aroA; 3-phosphoshikimate 1-carboxyvinyltransferase [EC:2.5.1.19]	330
NZ_CP022121.1_1268	JBMCGNCK_01311	K00817	Amino acid metabolism	9105	hisC; histidinol-phosphate aminotransferase [EC:2.6.1.9]	177
NZ_CP022121.1_1162	JBMCGNCK_01202	K00821	Amino acid metabolism	9105	gD; acetylornithine/N-succinyldiaminopimelate aminotransferase [EC:2.6.1.11 2.6.1.1	255
NZ_CP022121.1_390	JBMCGNCK_00400	K00832	Amino acid metabolism	9105	tyrB; aromatic-amino-acid transaminase [EC:2.6.1.57]	149
NZ_CP022121.1_2556	JBMCGNCK_02620	K00928	Amino acid metabolism	9105	lysC; aspartate kinase [EC:2.7.2.4]	244
NZ_CP022121.1_1161	JBMCGNCK_01201	K00930	Amino acid metabolism	9105	argB; acetylglutamate kinase [EC:2.7.2.8]	228
NZ_CP022121.1_2827	JBMCGNCK_02899	K00931	Amino acid metabolism	9105	proB; glutamate 5-kinase [EC:2.7.2.11]	274
NZ_CP022121.1_2907	JBMCGNCK_02983	K01251	Amino acid metabolism	9105	E3.3.1.1, ahcY; adenosylhomocysteinase [EC:3.3.1.1]	296
NZ_CP022121.1_2666	JBMCGNCK_02731	K01585	Amino acid metabolism	9105	speA; arginine decarboxylase [EC:4.1.1.19]	352
NZ_CP022121.1_2962	JBMCGNCK_03042	K01609	Amino acid metabolism	9105	trpC; indole-3-glycerol phosphate synthase [EC:4.1.1.48]	124
NZ_CP022121.1_2001	JBMCGNCK_02059	K01611	Amino acid metabolism	9105	speD, AMD1; S-adenosylmethionine decarboxylase [EC:4.1.1.50]	122
NZ_CP022121.1_2420	JBMCGNCK_02483	K01695	Amino acid metabolism	9105	trpA; tryptophan synthase alpha chain [EC:4.2.1.20]	159
NZ_CP022121.1_2422	JBMCGNCK_02485	K01696	Amino acid metabolism	9105	trpB; tryptophan synthase beta chain [EC:4.2.1.20]	158
NZ_CP022121.1_2421	JBMCGNCK_02484	K01696	Amino acid metabolism	9105	trpB; tryptophan synthase beta chain [EC:4.2.1.20]	67
NZ_CP022121.1_1414	JBMCGNCK_01468	K01714	Amino acid metabolism	9105	dapA; 4-hydroxy-tetrahydrodipicolinate synthase [EC:4.3.3.7]	169
NZ_CP022121.1_2640	JBMCGNCK_02705	K01735	Amino acid metabolism	9105	aroB; 3-dehydroquinate synthase [EC:4.2.3.4]	159
NZ_CP022121.1_2642	JBMCGNCK_02707	K01736	Amino acid metabolism	9105	aroC; chorismate synthase [EC:4.2.3.5]	319
NZ_CP022121.1_3563	JBMCGNCK_03651	K01740	Amino acid metabolism	9105	metY; O-acetylhomoserine (thiol)-lyase [EC:2.5.1.49]	340
NZ_CP022121.1_1189	JBMCGNCK_01229	K01755	Amino acid metabolism	9105	argH, ASL; argininosuccinate lyase [EC:4.3.2.1]	333
NZ_CP022121.1_995	JBMCGNCK_01023	K01778	Amino acid metabolism	9105	dapF; diaminopimelate epimerase [EC:5.1.1.7]	160
NZ_CP022121.1_3421	JBMCGNCK_03508	K01814	Amino acid metabolism	9105	phosphoribosylformimino-5-aminoimidazole carboxamide ribotide isomerase [EC:5.3.	134
NZ_CP022121.1_2528	JBMCGNCK_02592	K01817	Amino acid metabolism	9105	trpF; phosphoribosylanthranilate isomerase [EC:5.3.1.24]	21
NZ_CP022121.1_227	JBMCGNCK_00236	K01912	Amino acid metabolism	9105	paaK; phenylacetate-CoA ligase [EC:6.2.1.30]	307
NZ_CP022121.1_3599	JBMCGNCK_03687	K01912	Amino acid metabolism	9105	paaK; phenylacetate-CoA ligase [EC:6.2.1.30]	286
NZ_CP022121.1_3420	JBMCGNCK_03507	K02500	Amino acid metabolism	9105	hisF; imidazole glycerol-phosphate synthase subunit HisF [EC:4.3.2.10]	84
NZ_CP022121.1_3422	JBMCGNCK_03509	K02501	Amino acid metabolism	9105	hisH; imidazole glycerol-phosphate synthase subunit HisH [EC:4.3.2.10]	108
NZ_CP022121.1_3426	JBMCGNCK_03513	K02502	Amino acid metabolism	9105	hisZ; ATP phosphoribosyltransferase regulatory subunit	120
NZ_CP022121.1_1621	JBMCGNCK_01674	K03856	Amino acid metabolism	9105	AROA2, aroA; 3-deoxy-7-phosphoheptulonate synthase [EC:2.5.1.54]	222
NZ_CP022121.1_2529	JBMCGNCK_02593	K03856	Amino acid metabolism	9105	AROA2, aroA; 3-deoxy-7-phosphoheptulonate synthase [EC:2.5.1.54]	156
NZ_CP022121.1_2530	JBMCGNCK_02594	K04517	Amino acid metabolism	9105	tyrA2; prephenate dehydrogenase [EC:1.3.1.12]	146
NZ_CP022121.1_2830	JBMCGNCK_02903	K06001	Amino acid metabolism	9105	trpB; tryptophan synthase beta chain [EC:4.2.1.20]	343
NZ_CP022121.1_2527	JBMCGNCK_02591	K06208	Amino acid metabolism	9105	aroH; chorismate mutase [EC:5.4.99.5]	64
NZ_CP022121.1_1375	JBMCGNCK_01426	K08963	Amino acid metabolism	9105	mtnA; methylthioribose-1-phosphate isomerase [EC:5.3.1.23]	191
NZ_CP022121.1_808	JBMCGNCK_00829	K09251	Amino acid metabolism	9105	patA; putrescine aminotransferase [EC:2.6.1.82]	301
NZ_CP022121.1_847	JBMCGNCK_00873	K10206	Amino acid metabolism	9105	E2.6.1.83; LL-diaminopimelate aminotransferase [EC:2.6.1.83]	452
NZ_CP022121.1_3409	JBMCGNCK_03496	K11755	Amino acid metabolism	9105	horibosyl-AMP cyclohydrolase / phosphoribosyl-ATP pyrophosphohydrolase [EC:3.5.4.	18
NZ_CP022121.1_2955	JBMCGNCK_03035	K12339	Amino acid metabolism	9105	cysM; S-sulfo-L-cysteine synthase (O-acetyl-L-serine-dependent) [EC:2.5.1.144]	268
NZ_CP022121.1_1377	JBMCGNCK_01428	K12960	Amino acid metabolism	9105	taD; 5-methylthioadenosine/S-adenosylhomocysteine deaminase [EC:3.5.4.31 3.5.4.2	250
NZ_CP022121.1_1165	JBMCGNCK_01205	K16182	Amino acid metabolism	9105	pylD; 3-methylornithyl-N6-L-lysine dehydrogenase [EC:1.4.1.-]	66
NZ_CP022121.1_2363	JBMCGNCK_02432	K23121	Amino acid metabolism	9105	pflD; trans-4-hydroxy-L-proline dehydratase [EC:4.2.1.172]	538
NZ_CP022121.1_2429	JBMCGNCK_02492	K01626	Amino acid metabolism, Cellular processes	9140	E2.5.1.54, aroF, aroG, aroH; 3-deoxy-7-phosphoheptulonate synthase [EC:2.5.1.54]	208
NZ_CP022121.1_2965	JBMCGNCK_03045	K01657	Amino acid metabolism, Cellular processes	9140	trpE; anthranilate synthase component I [EC:4.1.3.27]	309
NZ_CP022121.1_2964	JBMCGNCK_03044	K01658	Amino acid metabolism, Cellular processes	9140	trpG; anthranilate synthase component II [EC:4.1.3.27]	132
NZ_CP022121.1_2452	JBMCGNCK_02516	K00058	Amino acid metabolism, Energy metabolism	9105, 9102	GDH; D-3-phosphoglycerate dehydrogenase / 2-oxoglutarate reductase [EC:1.1.1.95 1.	315
NZ_CP022121.1_1028	JBMCGNCK_01056	K00262	Amino acid metabolism, Energy metabolism	9105, 9102	E1.4.1.4, gdhA; glutamate dehydrogenase (NADP+) [EC:1.4.1.4]	299
NZ_CP022121.1_3566	JBMCGNCK_03654	K00651	Amino acid metabolism, Energy metabolism	9105, 9102	metA; homoserine O-succinyltransferase/O-acetyltransferase [EC:2.3.1.46 2.3.1.31]	202
NZ_CP022121.1_3562	JBMCGNCK_03650	K01738	Amino acid metabolism, Energy metabolism	9105, 9102	cysK; cysteine synthase [EC:2.5.1.47]	236
NZ_CP022121.1_1665	JBMCGNCK_01718	K01928	Amino acid metabolism, Glycan biosynthesis and metabolism	9105, 9107	E; UDP-N-acetylmuramoyl-L-alanyl-D-glutamate--2,6-diaminopimelate ligase [EC:6.3.2	233
NZ_CP022121.1_1163	JBMCGNCK_01203	K01940	Amino acid metabolism, Human diseases	9105, 9160	argG, ASS1; argininosuccinate synthase [EC:6.3.4.5]	426
NZ_CP022121.1_804	JBMCGNCK_00825	K00053	Amino acid metabolism, Metabolism of cofactors and vitamins	9105, 9108	ilvC; ketol-acid reductoisomerase [EC:1.1.1.86]	242
NZ_CP022121.1_2787	JBMCGNCK_02856	K00826	Amino acid metabolism, Metabolism of cofactors and vitamins	9105, 9108	E2.6.1.42, ilvE; branched-chain amino acid aminotransferase [EC:2.6.1.42]	355
NZ_CP022121.1_391	JBMCGNCK_00401	K00831	Amino acid metabolism, Metabolism of cofactors and vitamins, Energy metabolism	9105, 9108, 9102	serC, PSAT1; phosphoserine aminotransferase [EC:2.6.1.52]	249
NZ_CP022121.1_9	JBMCGNCK_00010	K00548	nino acid metabolism, Metabolism of cofactors and vitamins, Metabolism of other amino ac	9105, 9108, 9106	metH, MTR; 5-methyltetrahydrofolate--homocysteine methyltransferase [EC:2.1.1.13]	51
NZ_CP022121.1_2665	JBMCGNCK_02730	K00797	Amino acid metabolism, Metabolism of other amino acids	9105, 9106	speE, SRM, SPE3; spermidine synthase [EC:2.5.1.16]	218
NZ_CP022121.1_867	JBMCGNCK_00894	K01586	Amino acid metabolism, Metabolism of other amino acids	9105, 9106	lysA; diaminopimelate decarboxylase [EC:4.1.1.20]	313
NZ_CP022121.1_402	JBMCGNCK_00412	K01777	Amino acid metabolism, Metabolism of other amino acids	9105, 9106	prdF; proline racemase [EC:5.1.1.4]	181
NZ_CP022121.1_3468	JBMCGNCK_03554	K00764	Amino acid metabolism, Nucleotide metabolism	9105, 9104	purF, PPAT; amidophosphoribosyltransferase [EC:2.4.2.14]	298
NZ_CP022121.1_1071	JBMCGNCK_01101	K00764	Amino acid metabolism, Nucleotide metabolism	9105, 9104	purF, PPAT; amidophosphoribosyltransferase [EC:2.4.2.14]	222
NZ_CP022121.1_3469	JBMCGNCK_03555	K01756	Amino acid metabolism, Nucleotide metabolism	9105, 9104	purB, ADSL; adenylosuccinate lyase [EC:4.3.2.2]	312
NZ_CP022121.1_810	JBMCGNCK_00831	K01939	Amino acid metabolism, Nucleotide metabolism	9105, 9104	purA, ADSS; adenylosuccinate synthase [EC:6.3.4.4]	408
NZ_CP022121.1_3237	JBMCGNCK_03318	K01955	Amino acid metabolism, Nucleotide metabolism	9105, 9104	carB, CPA2; carbamoyl-phosphate synthase large subunit [EC:6.3.5.5]	807
NZ_CP022121.1_1726	JBMCGNCK_01779	K05810	Amino acid metabolism, Nucleotide metabolism	9105, 9104	-nucleoside/S-methyl-5'-thioadenosine phosphorylase / adenosine deaminase [EC:2.4.	167
NZ_CP022121.1_1540	JBMCGNCK_01593	K01687	Amino acid metabolism, Metabolism of cofactors and vitamins	9105, 9108	ilvD; dihydroxy-acid dehydratase [EC:4.2.1.9]	395
NZ_CP022121.1_1508	JBMCGNCK_01562	K01733	Amino acid metabolism, Metabolism of cofactors and vitamins	9105, 9108	thrC; threonine synthase [EC:4.2.3.1]	292
NZ_CP022121.1_2782	JBMCGNCK_02851	K00104	Carbohydrate metabolism	9101	glcD; glycolate oxidase [EC:1.1.3.15]	301
NZ_CP022121.1_3460	JBMCGNCK_03546	K00283	Carbohydrate metabolism	9101	gcvPB; glycine dehydrogenase subunit 2 [EC:1.4.4.2]	333
NZ_CP022121.1_891	JBMCGNCK_00918	K00616	Carbohydrate metabolism	9101	E2.2.1.2, talA, talB; transaldolase [EC:2.2.1.2]	179
NZ_CP022121.1_988	JBMCGNCK_01016	K00700	Carbohydrate metabolism	9101	GBE1, glgB; 1,4-alpha-glucan branching enzyme [EC:2.4.1.18]	346
NZ_CP022121.1_3170	JBMCGNCK_03250	K01571	Carbohydrate metabolism	9101	oadA; oxaloacetate decarboxylase (Na+ extruding) subunit alpha [EC:7.2.4.2]	164
NZ_CP022121.1_1376	JBMCGNCK_01427	K01628	Carbohydrate metabolism	9101	fucA; L-fuculose-phosphate aldolase [EC:4.1.2.17]	122
NZ_CP022121.1_3248	JBMCGNCK_03329	K01759	Carbohydrate metabolism	9101	GLO1, gloA; lactoylglutathione lyase [EC:4.4.1.5]	87
NZ_CP022121.1_3575	JBMCGNCK_03663	K01759	Carbohydrate metabolism	9101	GLO1, gloA; lactoylglutathione lyase [EC:4.4.1.5]	74
NZ_CP022121.1_1564	JBMCGNCK_01617	K02564	Carbohydrate metabolism	9101	nagB, GNPDA; glucosamine-6-phosphate deaminase [EC:3.5.99.6]	152
NZ_CP022121.1_1560	JBMCGNCK_01613	K03431	Carbohydrate metabolism	9101	glmM; phosphoglucosamine mutase [EC:5.4.2.10]	273
NZ_CP022121.1_3051	JBMCGNCK_03132	K05942	Carbohydrate metabolism	9101	E2.3.3.3; citrate (Re)-synthase [EC:2.3.3.3]	430
NZ_CP022121.1_2781	JBMCGNCK_02850	K11473	Carbohydrate metabolism	9101	glcF; glycolate oxidase iron-sulfur subunit	134
NZ_CP022121.1_987	JBMCGNCK_01015	K15916	Carbohydrate metabolism	9101	pgi-pmi; glucose/mannose-6-phosphate isomerase [EC:5.3.1.9 5.3.1.8]	180
NZ_CP022121.1_1166	JBMCGNCK_01206	K22373	Carbohydrate metabolism	9101	larA; lactate racemase [EC:5.1.2.1]	202
NZ_CP022121.1_1546	JBMCGNCK_01599	K00052	Carbohydrate metabolism, Amino acid metabolism	9101, 9105	leuB, IMDH; 3-isopropylmalate dehydrogenase [EC:1.1.1.85]	247
NZ_CP022121.1_3461	JBMCGNCK_03547	K00282	Carbohydrate metabolism, Amino acid metabolism	9101, 9105	gcvPA; glycine dehydrogenase subunit 1 [EC:1.4.4.2]	284
NZ_CP022121.1_2259	JBMCGNCK_02328	K00382	Carbohydrate metabolism, Amino acid metabolism	9101, 9105	DLD, lpd, pdhD; dihydrolipoamide dehydrogenase [EC:1.8.1.4]	261
NZ_CP022121.1_3459	JBMCGNCK_03545	K00382	Carbohydrate metabolism, Amino acid metabolism	9101, 9105	DLD, lpd, pdhD; dihydrolipoamide dehydrogenase [EC:1.8.1.4]	222
NZ_CP022121.1_1547	JBMCGNCK_01600	K01649	Carbohydrate metabolism, Amino acid metabolism	9101, 9105	leuA, IMS; 2-isopropylmalate synthase [EC:2.3.3.13]	383
NZ_CP022121.1_1543	JBMCGNCK_01596	K01649	Carbohydrate metabolism, Amino acid metabolism	9101, 9105	leuA, IMS; 2-isopropylmalate synthase [EC:2.3.3.13]	348
NZ_CP022121.1_1544	JBMCGNCK_01597	K01703	Carbohydrate metabolism, Amino acid metabolism	9101, 9105	MI-L; 3-isopropylmalate/(R)-2-methylmalate dehydratase large subunit [EC:4.2.1.33 4	327
NZ_CP022121.1_1545	JBMCGNCK_01598	K01704	Carbohydrate metabolism, Amino acid metabolism	9101, 9105	MI-S; 3-isopropylmalate/(R)-2-methylmalate dehydratase small subunit [EC:4.2.1.33 4	116
NZ_CP022121.1_1921	JBMCGNCK_01978	K02437	Carbohydrate metabolism, Amino acid metabolism	9101, 9105	gcvH, GCSH; glycine cleavage system H protein	124
NZ_CP022121.1_3462	JBMCGNCK_03548	K02437	Carbohydrate metabolism, Amino acid metabolism	9101, 9105	gcvH, GCSH; glycine cleavage system H protein	67
NZ_CP022121.1_357	JBMCGNCK_00365	K15633	Carbohydrate metabolism, Amino acid metabolism, Energy metabolism	9101, 9105, 9102	gpmI; 2,3-bisphosphoglycerate-independent phosphoglycerate mutase [EC:5.4.2.12]	320
NZ_CP022121.1_1279	JBMCGNCK_01324	K15635	Carbohydrate metabolism, Amino acid metabolism, Energy metabolism	9101, 9105, 9102	apgM; 2,3-bisphosphoglycerate-independent phosphoglycerate mutase [EC:5.4.2.12]	235
NZ_CP022121.1_993	JBMCGNCK_01021	K01915	rate metabolism, Amino acid metabolism, Energy metabolism, Cellular processes, Organism	)1, 9105, 9102, 9140, 9	glnA, GLUL; glutamine synthetase [EC:6.3.1.2]	352
NZ_CP022121.1_1069	JBMCGNCK_01099	K01915	rate metabolism, Amino acid metabolism, Energy metabolism, Cellular processes, Organism	)1, 9105, 9102, 9140, 9	glnA, GLUL; glutamine synthetase [EC:6.3.1.2]	240
NZ_CP022121.1_1068	JBMCGNCK_01098	K00820	Carbohydrate metabolism, Amino acid metabolism, Human diseases	9101, 9105, 9160	glmS, GFPT; glutamine---fructose-6-phosphate transaminase (isomerizing) [EC:2.6.1.16	341
NZ_CP022121.1_3463	JBMCGNCK_03549	K00605	Carbohydrate metabolism, Amino acid metabolism, Metabolism of cofactors and vitamins	9101, 9105, 9108	gcvT, AMT; aminomethyltransferase [EC:2.1.2.10]	268
NZ_CP022121.1_731	JBMCGNCK_00753	K01652	Carbohydrate metabolism, Amino acid metabolism, Metabolism of cofactors and vitamins	9101, 9105, 9108	E2.2.1.6L, ilvB, ilvG, ilvI; acetolactate synthase I/II/III large subunit [EC:2.2.1.6]	341
NZ_CP022121.1_1542	JBMCGNCK_01595	K01652	Carbohydrate metabolism, Amino acid metabolism, Metabolism of cofactors and vitamins	9101, 9105, 9108	E2.2.1.6L, ilvB, ilvG, ilvI; acetolactate synthase I/II/III large subunit [EC:2.2.1.6]	316
NZ_CP022121.1_732	JBMCGNCK_00754	K01653	Carbohydrate metabolism, Amino acid metabolism, Metabolism of cofactors and vitamins	9101, 9105, 9108	E2.2.1.6S, ilvH, ilvN; acetolactate synthase I/III small subunit [EC:2.2.1.6]	116
NZ_CP022121.1_905	JBMCGNCK_00932	K00600	acid metabolism, Metabolism of cofactors and vitamins, Energy metabolism, Metabolism of	9105, 9108, 9102, 9106	glyA, SHMT; glycine hydroxymethyltransferase [EC:2.1.2.1]	289
NZ_CP022121.1_1911	JBMCGNCK_01968	K00140	Carbohydrate metabolism, Amino acid metabolism, Metabolism of other amino acids	9101, 9105, 9106	te-semialdehyde dehydrogenase (acetylating) / methylmalonate-semialdehyde dehydı	301
NZ_CP022121.1_991	JBMCGNCK_01019	K00703	Carbohydrate metabolism, Cellular processes	9101, 9140	glgA; starch synthase [EC:2.4.1.21]	258
NZ_CP022121.1_2957	JBMCGNCK_03037	K00688	Carbohydrate metabolism, Cellular processes, Organismal system, Human diseases	9101, 9140, 9150, 9160	PYG, glgP; glycogen phosphorylase [EC:2.4.1.1]	442
NZ_CP022121.1_989	JBMCGNCK_01017	K00688	Carbohydrate metabolism, Cellular processes, Organismal system, Human diseases	9101, 9140, 9150, 9160	PYG, glgP; glycogen phosphorylase [EC:2.4.1.1]	432
NZ_CP022121.1_2751	JBMCGNCK_02819	K00123	Carbohydrate metabolism, Energy metabolism	9101, 9102	fdoG, fdhF, fdwA; formate dehydrogenase major subunit [EC:1.17.1.9]	322
NZ_CP022121.1_1497	JBMCGNCK_01551	K00123	Carbohydrate metabolism, Energy metabolism	9101, 9102	fdoG, fdhF, fdwA; formate dehydrogenase major subunit [EC:1.17.1.9]	260
NZ_CP022121.1_1681	JBMCGNCK_01734	K00174	Carbohydrate metabolism, Energy metabolism	9101, 9102	A, oforA; 2-oxoglutarate/2-oxoacid ferredoxin oxidoreductase subunit alpha [EC:1.2.7.3	305
NZ_CP022121.1_1680	JBMCGNCK_01733	K00175	Carbohydrate metabolism, Energy metabolism	9101, 9102	B, oforB; 2-oxoglutarate/2-oxoacid ferredoxin oxidoreductase subunit beta [EC:1.2.7.3	174
NZ_CP022121.1_961	JBMCGNCK_00988	K00615	Carbohydrate metabolism, Energy metabolism	9101, 9102	E2.2.1.1, tktA, tktB; transketolase [EC:2.2.1.1]	212
NZ_CP022121.1_1217	JBMCGNCK_01257	K01006	Carbohydrate metabolism, Energy metabolism	9101, 9102	ppdK; pyruvate, orthophosphate dikinase [EC:2.7.9.1]	632

NZ_CP022121.1_2780	JBMCGNCK_02849	K01624	Carbohydrate metabolism, Energy metabolism	9101, 9102	FBA, fbaA; fructose-bisphosphate aldolase, class II [EC:4.1.2.13]	251
NZ_CP022121.1_1588	JBMCGNCK_01641	K01678	Carbohydrate metabolism, Energy metabolism	9101, 9102	E4.2.1.2AB, fumB; fumarate hydratase subunit beta [EC:4.2.1.2]	161
NZ_CP022121.1_2741	JBMCGNCK_02808	K01681	Carbohydrate metabolism, Energy metabolism	9101, 9102	ACO, acnA; aconitate hydratase [EC:4.2.1.3]	400
NZ_CP022121.1_358	JBMCGNCK_00366	K01803	Carbohydrate metabolism, Energy metabolism	9101, 9102	TPI, tpiA; triosephosphate isomerase (TIM) [EC:5.3.1.1]	174
NZ_CP022121.1_904	JBMCGNCK_00931	K01808	Carbohydrate metabolism, Energy metabolism	9101, 9102	rpiB; ribose 5-phosphate isomerase B [EC:5.3.1.6]	117
NZ_CP022121.1_3600	JBMCGNCK_03688	K01958	Carbohydrate metabolism, Energy metabolism	9101, 9102	PC, pyc; pyruvate carboxylase [EC:6.4.1.1]	856
NZ_CP022121.1_495	JBMCGNCK_00507	K03737	Carbohydrate metabolism, Energy metabolism	9101, 9102	por, nifJ; pyruvate-ferredoxin/flavodoxin oxidoreductase [EC:1.2.7.1 1.2.7.-]	868
NZ_CP022121.1_2786	JBMCGNCK_02855	K04041	Carbohydrate metabolism, Energy metabolism	9101, 9102	fbp3; fructose-1,6-bisphosphatase III [EC:3.1.3.11]	510
NZ_CP022121.1_2738	JBMCGNCK_02805	K21071	Carbohydrate metabolism, Energy metabolism	9101, 9102	ependent phosphofructokinase / diphosphate-dependent phosphofructokinase [EC:2.7	278
NZ_CP022121.1_806	JBMCGNCK_00827	K21071	Carbohydrate metabolism, Energy metabolism	9101, 9102	ependent phosphofructokinase / diphosphate-dependent phosphofructokinase [EC:2.7	219
NZ_CP022121.1_1636	JBMCGNCK_01689	K00031	e metabolism, Energy metabolism, Cellular processes, Metabolism of other amino acids, Hu	)1, 9102, 9140, 9106, 9:	IDH1, IDH2, icd; isocitrate dehydrogenase [EC:1.1.1.42]	328
NZ_CP022121.1_359	JBMCGNCK_00367	K00927	Carbohydrate metabolism, Energy metabolism, Environmental information processing	9101, 9102, 9130	PGK, pgk; phosphoglycerate kinase [EC:2.7.2.3]	263
NZ_CP022121.1_360	JBMCGNCK_00368	K00134	hydrate metabolism, Energy metabolism, Environmental information processing, Human dis	9101, 9102, 9130, 9160	APDH, gapA; glyceraldehyde 3-phosphate dehydrogenase (phosphorylating) [EC:1.2.1.1	258
NZ_CP022121.1_2861	JBMCGNCK_02935	K00925	Carbohydrate metabolism, Energy metabolism, Metabolism of other amino acids	9101, 9102, 9106	ackA; acetate kinase [EC:2.7.2.1]	320
NZ_CP022121.1_275	JBMCGNCK_00283	K15024	Carbohydrate metabolism, Energy metabolism, Metabolism of other amino acids	9101, 9102, 9106	K15024; putative phosphotransacetylase [EC:2.3.1.8]	125
NZ_CP022121.1_285	JBMCGNCK_00293	K00169	Carbohydrate metabolism, Energy metabolism, Xenobiotics biodegradation and metabolism	9101, 9102, 9111	porA; pyruvate ferredoxin oxidoreductase alpha subunit [EC:1.2.7.1]	99
NZ_CP022121.1_286	JBMCGNCK_00294	K00170	Carbohydrate metabolism, Energy metabolism, Xenobiotics biodegradation and metabolism	9101, 9102, 9111	porB; pyruvate ferredoxin oxidoreductase beta subunit [EC:1.2.7.1]	176
NZ_CP022121.1_284	JBMCGNCK_00292	K00171	Carbohydrate metabolism, Energy metabolism, Xenobiotics biodegradation and metabolism	9101, 9102, 9111	porD; pyruvate ferredoxin oxidoreductase delta subunit [EC:1.2.7.1]	32
NZ_CP022121.1_283	JBMCGNCK_00291	K00172	Carbohydrate metabolism, Energy metabolism, Xenobiotics biodegradation and metabolism	9101, 9102, 9111	porC, porG; pyruvate ferredoxin oxidoreductase gamma subunit [EC:1.2.7.1]	57
NZ_CP022121.1_1589	JBMCGNCK_01642	K00027	Carbohydrate metabolism, Environmental information processing	9101, 9130	ME2, sfcA, maeA; malate dehydrogenase (oxaloacetate-decarboxylating) [EC:1.1.1.38]	248
NZ_CP022121.1_1379	JBMCGNCK_01430	K03781	hydrate metabolism, Environmental information processing, Organismal systems, Human di	9101, 9130, 9150, 9160	katE, CAT, catB, srpA; catalase [EC:1.11.1.6]	472
NZ_CP022121.1_356	JBMCGNCK_00364	K01689	lism, Genetic information processing, Energy metabolism, Environmntal information process	)1, 9120, 9102, 9130, 9:	ENO, eno; enolase [EC:4.2.1.11]	378
NZ_CP022121.1_3034	JBMCGNCK_03115	K00012	Carbohydrate metabolism, Glycan biosynthesis and metabolism	9101, 9107	UGDH, ugd; UDPglucose 6-dehydrogenase [EC:1.1.1.22]	333
NZ_CP022121.1_3482	JBMCGNCK_03568	K00075	Carbohydrate metabolism, Glycan biosynthesis and metabolism	9101, 9107	murB; UDP-N-acetylmuramate dehydrogenase [EC:1.3.1.98]	147
NZ_CP022121.1_923	JBMCGNCK_00950	K00790	Carbohydrate metabolism, Glycan biosynthesis and metabolism	9101, 9107	murA; UDP-N-acetylglucosamine 1-carboxyvinyltransferase [EC:2.5.1.7]	365
NZ_CP022121.1_843	JBMCGNCK_00869	K00790	Carbohydrate metabolism, Glycan biosynthesis and metabolism	9101, 9107	murA; UDP-N-acetylglucosamine 1-carboxyvinyltransferase [EC:2.5.1.7]	295
NZ_CP022121.1_2057	JBMCGNCK_02120	K00963	Carbohydrate metabolism, Glycan biosynthesis and metabolism	9101, 9107	UGP2, galU, galF; UTP--glucose-1-phosphate uridylyltransferase [EC:2.7.7.9]	190
NZ_CP022121.1_3062	JBMCGNCK_03143	K01784	Carbohydrate metabolism, Glycan biosynthesis and metabolism	9101, 9107	galE, GALE; UDP-glucose 4-epimerase [EC:5.1.3.2]	216
NZ_CP022121.1_1001	JBMCGNCK_01029	K04042	Carbohydrate metabolism, Glycan biosynthesis and metabolism	9101, 9107	I-acetylglucosamine pyrophosphorylase / glucosamine-1-phosphate N-acetyltransfera:	246
NZ_CP022121.1_3033	JBMCGNCK_03114	K08679	Carbohydrate metabolism, Glycan biosynthesis and metabolism	9101, 9107	GAE, cap1J; UDP-glucuronate 4-epimerase [EC:5.1.3.6]	168
NZ_CP022121.1_3059	JBMCGNCK_03140	K01790	ydrate metabolism, Glycan biosynthesis and metabolism, Metabolism of terpenoids and poly	9101, 9107, 9109	rfbC, rmlC; dTDP-4-dehydrorhamnose 3,5-epimerase [EC:5.1.3.13]	58
NZ_CP022121.1_960	JBMCGNCK_00987	K00615	Carbohydrate metabolism, Metabolism of terpenoids and polyketides	9101, 9109	E2.2.1.1, tktA, tktB; transketolase [EC:2.2.1.1]	180
NZ_CP022121.1_1002	JBMCGNCK_01030	K00948	Carbohydrate metabolism, Nucleotide metabolism	9101, 9104	PRPS, prsA; ribose-phosphate pyrophosphokinase [EC:2.7.6.1]	236
NZ_CP022121.1_2484	JBMCGNCK_02548	K01839	Carbohydrate metabolism, Nucleotide metabolism	9101, 9104	deoB; phosphopentomutase [EC:5.4.2.7]	248
NZ_CP022121.1_3171	JBMCGNCK_03251	K00873	Carbohydrate metabolism, Organismal system, Human diseases	9101, 9150, 9160	PK, pyk; pyruvate kinase [EC:2.7.1.40]	347
NZ_CP022121.1_2904	JBMCGNCK_02980	K23351	Carbohydrate metabolism, Xenobiotics biodegradation and metabolism	9101, 9111	gcdC, mmdC; glutaconyl-CoA/methylmalonyl-CoA decarboxylase subunit gamma	43
NZ_CP022121.1_215	JBMCGNCK_00217	K01338	Cellular processes	9140	lon; ATP-dependent Lon protease [EC:3.4.21.53]	548
NZ_CP022121.1_1264	JBMCGNCK_01305	K03086	Cellular processes	9140	rpoD; RNA polymerase primary sigma factor	276
NZ_CP022121.1_1674	JBMCGNCK_01727	K03531	Cellular processes	9140	ftsZ; cell division protein FtsZ	225
NZ_CP022121.1_217	JBMCGNCK_00219	K03544	Cellular processes	9140	clpX, CLPX; ATP-dependent Clp protease ATP-binding subunit ClpX	348
NZ_CP022121.1_1673	JBMCGNCK_01726	K03590	Cellular processes	9140	ftsA; cell division protein FtsA	182
NZ_CP022121.1_434	JBMCGNCK_00444	K05946	Cellular processes, Glycan biosynthesis and metabolism	9140, 9107	acetylglucosaminyldiphosphoundecaprenol N-acetyl-beta-D-mannosaminyltransferase	82
NZ_CP022121.1_218	JBMCGNCK_00220	K01358	Cellular processes, Organismal systems	9140, 9150	clpP, CLPP; ATP-dependent Clp protease, protease subunit [EC:3.4.21.92]	158
NZ_CP022121.1_1646	JBMCGNCK_01699	K00125	Energy metabolism	9102	fdhB; formate dehydrogenase (coenzyme F420) beta subunit [EC:1.17.98.3 1.8.98.6]	113
NZ_CP022121.1_2920	JBMCGNCK_02997	K00194	Energy metabolism	9102	acsD; acetyl-CoA decarbonylase/synthase, CODH/ACS complex subunit delta [EC:2.1.:	264
NZ_CP022121.1_2923	JBMCGNCK_03000	K00197	Energy metabolism	9102	acsC; acetyl-CoA decarbonylase/synthase, CODH/ACS complex subunit gamma [EC:2.1.	278
NZ_CP022121.1_2801	JBMCGNCK_02873	K00332	Energy metabolism	9102	nuoC; NADH-quinone oxidoreductase subunit C [EC:7.1.1.2]	44
NZ_CP022121.1_2800	JBMCGNCK_02872	K00333	Energy metabolism	9102	nuoD; NADH-quinone oxidoreductase subunit D [EC:7.1.1.2]	289
NZ_CP022121.1_1504	JBMCGNCK_01558	K00335	Energy metabolism	9102	nuoF; NADH-quinone oxidoreductase subunit F [EC:7.1.1.2]	484
NZ_CP022121.1_1583	JBMCGNCK_01636	K00335	Energy metabolism	9102	nuoF; NADH-quinone oxidoreductase subunit F [EC:7.1.1.2]	210
NZ_CP022121.1_1496	JBMCGNCK_01550	K00335	Energy metabolism	9102	nuoF; NADH-quinone oxidoreductase subunit F [EC:7.1.1.2]	183
NZ_CP022121.1_2798	JBMCGNCK_02870	K00338	Energy metabolism	9102	nuoI; NADH-quinone oxidoreductase subunit I [EC:7.1.1.2]	42
NZ_CP022121.1_2795	JBMCGNCK_02867	K00341	Energy metabolism	9102	nuoL; NADH-quinone oxidoreductase subunit L [EC:7.1.1.2]	299
NZ_CP022121.1_2794	JBMCGNCK_02866	K00342	Energy metabolism	9102	nuoM; NADH-quinone oxidoreductase subunit M [EC:7.1.1.2]	281
NZ_CP022121.1_2793	JBMCGNCK_02865	K00343	Energy metabolism	9102	nuoN; NADH-quinone oxidoreductase subunit N [EC:7.1.1.2]	304
NZ_CP022121.1_1364	JBMCGNCK_01415	K00459	Energy metabolism	9102	ncd2, npd; nitronate monooxygenase [EC:1.13.12.16]	106
NZ_CP022121.1_640	JBMCGNCK_00655	K00584	Energy metabolism	9102	mtrH; tetrahydromethanopterin S-methyltransferase subunit H [EC:2.1.1.86]	39
NZ_CP022121.1_1313	JBMCGNCK_01364	K01499	Energy metabolism	9102	mch; methenyltetrahydromethanopterin cyclohydrolase [EC:3.5.4.27]	189
NZ_CP022121.1_1793	JBMCGNCK_01847	K01673	Energy metabolism	9102	cynT, can; carbonic anhydrase [EC:4.2.1.1]	111
NZ_CP022121.1_913	JBMCGNCK_00940	K02110	Energy metabolism	9102	ATPF0C, atpE; F-type H+-transporting ATPase subunit c	39
NZ_CP022121.1_916	JBMCGNCK_00943	K02111	Energy metabolism	9102	ATPF1A, atpA; F-type H+/Na+-transporting ATPase subunit alpha [EC:7.1.2.2 7.2.2.1]	151
NZ_CP022121.1_919	JBMCGNCK_00946	K02112	Energy metabolism	9102	ATPF1B, atpD; F-type H+/Na+-transporting ATPase subunit beta [EC:7.1.2.2 7.2.2.1]	360
NZ_CP022121.1_915	JBMCGNCK_00942	K02113	Energy metabolism	9102	ATPF1D, atpH; F-type H+-transporting ATPase subunit delta	62
NZ_CP022121.1_920	JBMCGNCK_00947	K02114	Energy metabolism	9102	ATPF1E, atpC; F-type H+-transporting ATPase subunit epsilon	66
NZ_CP022121.1_396	JBMCGNCK_00406	K03385	Energy metabolism	9102	nrfA; nitrite reductase (cytochrome c-552) [EC:1.7.2.2]	180
NZ_CP022121.1_1644	JBMCGNCK_01697	K03388	Energy metabolism	9102	hdrA2; heterodisulfide reductase subunit A2 [EC:1.8.7.3 1.8.98.4 1.8.98.5 1.8.98.6]	285
NZ_CP022121.1_1643	JBMCGNCK_01696	K03388	Energy metabolism	9102	hdrA2; heterodisulfide reductase subunit A2 [EC:1.8.7.3 1.8.98.4 1.8.98.5 1.8.98.6]	34
NZ_CP022121.1_1642	JBMCGNCK_01695	K03389	Energy metabolism	9102	hdrB2; heterodisulfide reductase subunit B2 [EC:1.8.7.3 1.8.98.4 1.8.98.5 1.8.98.6]	142
NZ_CP022121.1_1641	JBMCGNCK_01694	K03390	Energy metabolism	9102	hdrC2; heterodisulfide reductase subunit C2 [EC:1.8.7.3 1.8.98.4 1.8.98.5 1.8.98.6]	84
NZ_CP022121.1_241	JBMCGNCK_00249	K04480	Energy metabolism	9102	ntaB; methanol---5-hydroxybenzimidazolylcobamide Co-methyltransferase [EC:2.1.1.9(	361
NZ_CP022121.1_2796	JBMCGNCK_02868	K05576	Energy metabolism	9102	ndhE; NAD(P)H-quinone oxidoreductase subunit 4L [EC:7.1.1.2]	38
NZ_CP022121.1_863	JBMCGNCK_00889	K05601	Energy metabolism	9102	hcp; hydroxylamine reductase [EC:1.7.99.1]	574
NZ_CP022121.1_2975	JBMCGNCK_03056	K05601	Energy metabolism	9102	hcp; hydroxylamine reductase [EC:1.7.99.1]	414
NZ_CP022121.1_726	JBMCGNCK_00748	K11261	Energy metabolism	9102	fwdE, fmdE; formylmethanofuran dehydrogenase subunit E [EC:1.2.7.12]	81
NZ_CP022121.1_3527	JBMCGNCK_03613	K14083	Energy metabolism	9102	mttB; trimethylamine---corrinoid protein Co-methyltransferase [EC:2.1.1.250]	206
NZ_CP022121.1_3572	JBMCGNCK_03660	K14083	Energy metabolism	9102	mttB; trimethylamine---corrinoid protein Co-methyltransferase [EC:2.1.1.250]	187
NZ_CP022121.1_1690	JBMCGNCK_01743	K14083	Energy metabolism	9102	mttB; trimethylamine---corrinoid protein Co-methyltransferase [EC:2.1.1.250]	159
NZ_CP022121.1_491	JBMCGNCK_00503	K14083	Energy metabolism	9102	mttB; trimethylamine---corrinoid protein Co-methyltransferase [EC:2.1.1.250]	121
NZ_CP022121.1_3526	JBMCGNCK_03612	K14083	Energy metabolism	9102	mttB; trimethylamine---corrinoid protein Co-methyltransferase [EC:2.1.1.250]	10
NZ_CP022121.1_1645	JBMCGNCK_01698	K14127	Energy metabolism	9102	huD, vhcD; F420-non-reducing hydrogenase iron-sulfur subunit [EC:1.12.99.- 1.8.98.5 :	34
NZ_CP022121.1_2924	JBMCGNCK_03001	K14138	Energy metabolism	9102	acsB; acetyl-CoA synthase [EC:2.3.1.169]	539
NZ_CP022121.1_2919	JBMCGNCK_02996	K15023	Energy metabolism	9102	E; 5-methyltetrahydrofolate corrinoid/iron sulfur protein methyltransferase [EC:2.1.1.2	189
NZ_CP022121.1_3571	JBMCGNCK_03659	K15023	Energy metabolism	9102	E; 5-methyltetrahydrofolate corrinoid/iron sulfur protein methyltransferase [EC:2.1.1.2	46
NZ_CP022121.1_1382	JBMCGNCK_01433	K15986	Energy metabolism	9102	ppaC; manganese-dependent inorganic pyrophosphatase [EC:3.6.1.1]	304
NZ_CP022121.1_1746	JBMCGNCK_01799	K16176	Energy metabolism	9102	mtmB; methylamine---corrinoid protein Co-methyltransferase [EC:2.1.1.248]	107
NZ_CP022121.1_2405	JBMCGNCK_02468	K16179	Energy metabolism	9102	mtbC; dimethylamine corrinoid protein	116
NZ_CP022121.1_1580	JBMCGNCK_01633	K00196	Energy metabolism, Xenobiotics biodegradation and metabolism	9102, 9111	cooF; anaerobic carbon-monoxide dehydrogenase iron sulfur subunit	63
NZ_CP022121.1_1585	JBMCGNCK_01638	K00198	Energy metabolism, Xenobiotics biodegradation and metabolism	9102, 9111	cooS, acsA; anaerobic carbon-monoxide dehydrogenase catalytic subunit [EC:1.2.7.4]	478
NZ_CP022121.1_2925	JBMCGNCK_03002	K00198	Energy metabolism, Xenobiotics biodegradation and metabolism	9102, 9111	cooS, acsA; anaerobic carbon-monoxide dehydrogenase catalytic subunit [EC:1.2.7.4]	415
NZ_CP022121.1_3205	JBMCGNCK_03285	K02588	Energy metabolism, Xenobiotics biodegradation and metabolism	9102, 9111	nifH; nitrogenase iron protein NifH	326
NZ_CP022121.1_2097	JBMCGNCK_02161	K02588	Energy metabolism, Xenobiotics biodegradation and metabolism	9102, 9111	nifH; nitrogenase iron protein NifH	99
NZ_CP022121.1_3616	JBMCGNCK_03704	K11180	Energy metabolism, Xenobiotics biodegradation and metabolism	9102, 9111	dsrA; dissimilatory sulfite reductase alpha subunit [EC:1.8.99.5]	366
NZ_CP022121.1_3615	JBMCGNCK_03703	K11181	Energy metabolism, Xenobiotics biodegradation and metabolism	9102, 9111	dsrB; dissimilatory sulfite reductase beta subunit [EC:1.8.99.5]	269
NZ_CP022121.1_121	JBMCGNCK_00122	K01546	Environmental information processing	9130	kdpA; potassium-transporting ATPase potassium-binding subunit	385
NZ_CP022121.1_122	JBMCGNCK_00123	K01547	Environmental information processing	9130	kdpB; potassium-transporting ATPase ATP-binding subunit [EC:7.2.2.6]	526
NZ_CP022121.1_125	JBMCGNCK_00126	K01548	Environmental information processing	9130	kdpC; potassium-transporting ATPase KdpC subunit	156
NZ_CP022121.1_3592	JBMCGNCK_03680	K02006	Environmental information processing	9130	cbiO; cobalt/nickel transport system ATP-binding protein	157
NZ_CP022121.1_3585	JBMCGNCK_03673	K02007	Environmental information processing	9130	cbiM; cobalt/nickel transport system permease protein	148
NZ_CP022121.1_411	JBMCGNCK_00421	K02020	Environmental information processing	9130	modA; molybdate transport system substrate-binding protein	115
NZ_CP022121.1_2409	JBMCGNCK_02472	K02073	Environmental information processing	9130	metQ; D-methionine transport system substrate-binding protein	56
NZ_CP022121.1_744	JBMCGNCK_00766	K07335	Environmental information processing	9130	bmpA, bmpB, tmpC; basic membrane protein A and related proteins	235
NZ_CP022121.1_117	JBMCGNCK_00118	K07646	Environmental information processing	9130	kdpD; two-component system, OmpR family, sensor histidine kinase KdpD [EC:2.7.13.3	483
NZ_CP022121.1_642	JBMCGNCK_00657	K07720	Environmental information processing	9130	yesN; two-component system, response regulator YesN	58
NZ_CP022121.1_3155	JBMCGNCK_03236	K07795	Environmental information processing	9130	tctC; putative tricarboxylic transport membrane protein	79
NZ_CP022121.1_1253	JBMCGNCK_01294	K09815	Environmental information processing	9130	znuA; zinc transport system substrate-binding protein	154
NZ_CP022121.1_1254	JBMCGNCK_01295	K09817	Environmental information processing	9130	znuC; zinc transport system ATP-binding protein [EC:7.2.2.20]	113
NZ_CP022121.1_1260	JBMCGNCK_01301	K15598	Environmental information processing	9130	thiY; putative hydroxymethylpyrimidine transport system substrate-binding protein	169
NZ_CP022121.1_2049	JBMCGNCK_02111	K18940	Environmental information processing	9130	arlS; two-component system, OmpR family, sensor histidine kinase ArlS [EC:2.7.13.3]	110
NZ_CP022121.1_932	JBMCGNCK_00959	K23059	Environmental information processing	9130	artP, artI; arginine/lysine/histidine transporter system substrate-binding protein	131
NZ_CP022121.1_934	JBMCGNCK_00961	K23060	Environmental information processing	9130	artR, artM; arginine/lysine/histidine transport system ATP-binding protein [EC:7.4.2.1]	165
NZ_CP022121.1_742	JBMCGNCK_00764	K23535	Environmental information processing	9130	nupB; general nucleoside transport system permease protein	277
NZ_CP022121.1_743	JBMCGNCK_00765	K23537	Environmental information processing	9130	nupA; general nucleoside transport system ATP-binding protein	274
NZ_CP022121.1_706	JBMCGNCK_00727	K01995	Environmental information processing, Cellular processes	9130, 9140	livG; branched-chain amino acid transport system ATP-binding protein	183
NZ_CP022121.1_967	JBMCGNCK_00994	K01996	Environmental information processing, Cellular processes	9130, 9140	livF; branched-chain amino acid transport system ATP-binding protein	151
NZ_CP022121.1_703	JBMCGNCK_00724	K01999	Environmental information processing, Cellular processes	9130, 9140	livK; branched-chain amino acid transport system substrate-binding protein	200
NZ_CP022121.1_962	JBMCGNCK_00989	K01999	Environmental information processing, Cellular processes	9130, 9140	livK; branched-chain amino acid transport system substrate-binding protein	71
NZ_CP022121.1_687	JBMCGNCK_00708	K02313	Environmental information processing, Cellular processes	9130, 9140	dnaA; chromosomal replication initiator protein	291
NZ_CP022121.1_2653	JBMCGNCK_02718	K02456	Environmental information processing, Cellular processes	9130, 9140	gspG; general secretion pathway protein G	20
NZ_CP022121.1_889	JBMCGNCK_00916	K02490	Environmental information processing, Cellular processes	9130, 9140	spo0F; two-component system, response regulator, stage 0 sporulation protein F	46
NZ_CP022121.1_363	JBMCGNCK_00371	K03092	Environmental information processing, Cellular processes	9130, 9140	rpoN; RNA polymerase sigma-54 factor	221
NZ_CP022121.1_238	JBMCGNCK_00246	K15580	Environmental information processing, Cellular processes, Human diseases	9130, 9140, 9160	oppA, mppA; oligopeptide transport system substrate-binding protein	250
NZ_CP022121.1_1285	JBMCGNCK_01330	K02040	Environmental information processing, Human diseases	9130, 9160	pstS; phosphate transport system substrate-binding protein	142
NZ_CP022121.1_845	JBMCGNCK_00871	K04771	Environmental information processing, Human diseases	9130, 9160	degP, htrA; serine protease Do [EC:3.4.21.107]	142
NZ_CP022121.1_3554	JBMCGNCK_03642	K17686	Environmental information processing, Organismal system, Human diseases	9130, 9150, 9160	copA, ctpA, ATP7; P-type Cu+ transporter [EC:7.2.2.8]	250
NZ_CP022121.1_3580	JBMCGNCK_03668	K16785	Environmental information processing, Protein families: signal and cellular processes	9130, 9183	ecfT; energy-coupling factor transport system permease protein	174
NZ_CP022121.1_2274	JBMCGNCK_02343	K18888	Environmental information processing, Protein families: signal and cellular processes	9130, 9183	efrB, efrF; ATP-binding cassette, subfamily B, multidrug efflux pump	276

NZ_CP022121.1_2689	JBMCGNCK_02754	K00566	Genetic information processing	9120	mnmA, trmU; tRNA-uridine 2-sulfurtransferase [EC:2.8.1.13]	225
NZ_CP022121.1_2567	JBMCGNCK_02632	K00962	Genetic information processing	9120	pnp, PNPT1; polyribonucleotide nucleotidyltransferase [EC:2.7.7.8]	476
NZ_CP022121.1_2936	JBMCGNCK_03016	K01866	Genetic information processing	9120	YARS, tyrS; tyrosyl-tRNA synthetase [EC:6.1.1.1]	313
NZ_CP022121.1_2509	JBMCGNCK_02573	K01867	Genetic information processing	9120	WARS, trpS; tryptophanyl-tRNA synthetase [EC:6.1.1.2]	232
NZ_CP022121.1_2733	JBMCGNCK_02800	K01868	Genetic information processing	9120	TARS, thrS; threonyl-tRNA synthetase [EC:6.1.1.3]	468
NZ_CP022121.1_76	JBMCGNCK_00077	K01869	Genetic information processing	9120	LARS, leuS; leucyl-tRNA synthetase [EC:6.1.1.4]	218
NZ_CP022121.1_75	JBMCGNCK_00076	K01869	Genetic information processing	9120	LARS, leuS; leucyl-tRNA synthetase [EC:6.1.1.4]	66
NZ_CP022121.1_1735	JBMCGNCK_01788	K01870	Genetic information processing	9120	IARS, ileS; isoleucyl-tRNA synthetase [EC:6.1.1.5]	662
NZ_CP022121.1_2682	JBMCGNCK_02747	K01872	Genetic information processing	9120	AARS, alaS; alanyl-tRNA synthetase [EC:6.1.1.7]	137
NZ_CP022121.1_2683	JBMCGNCK_02748	K01872	Genetic information processing	9120	AARS, alaS; alanyl-tRNA synthetase [EC:6.1.1.7]	61
NZ_CP022121.1_213	JBMCGNCK_00215	K01873	Genetic information processing	9120	VARS, valS; valyl-tRNA synthetase [EC:6.1.1.9]	609
NZ_CP022121.1_680	JBMCGNCK_00701	K01875	Genetic information processing	9120	SARS, serS; seryl-tRNA synthetase [EC:6.1.1.11]	315
NZ_CP022121.1_2694	JBMCGNCK_02760	K01876	Genetic information processing	9120	DARS2, aspS; aspartyl-tRNA synthetase [EC:6.1.1.12]	418
NZ_CP022121.1_1213	JBMCGNCK_01253	K01878	Genetic information processing	9120	glyQ; glycyl-tRNA synthetase alpha chain [EC:6.1.1.14]	292
NZ_CP022121.1_1214	JBMCGNCK_01254	K01879	Genetic information processing	9120	glyS; glycyl-tRNA synthetase beta chain [EC:6.1.1.14]	354
NZ_CP022121.1_1953	JBMCGNCK_02010	K01881	Genetic information processing	9120	PARS, proS; prolyl-tRNA synthetase [EC:6.1.1.15]	362
NZ_CP022121.1_1091	JBMCGNCK_01127	K01883	Genetic information processing	9120	CARS, cysS; cysteinyl-tRNA synthetase [EC:6.1.1.16]	307
NZ_CP022121.1_1096	JBMCGNCK_01132	K01886	Genetic information processing	9120	QARS, glnS; glutaminyl-tRNA synthetase [EC:6.1.1.18]	529
NZ_CP022121.1_881	JBMCGNCK_00908	K01887	Genetic information processing	9120	RARS, argS; arginyl-tRNA synthetase [EC:6.1.1.19]	378
NZ_CP022121.1_2728	JBMCGNCK_02795	K01889	Genetic information processing	9120	FARSA, pheS; phenylalanyl-tRNA synthetase alpha chain [EC:6.1.1.20]	251
NZ_CP022121.1_2727	JBMCGNCK_02794	K01890	Genetic information processing	9120	FARSB, pheT; phenylalanyl-tRNA synthetase beta chain [EC:6.1.1.20]	392
NZ_CP022121.1_2695	JBMCGNCK_02761	K01892	Genetic information processing	9120	HARS, hisS; histidyl-tRNA synthetase [EC:6.1.1.21]	269
NZ_CP022121.1_3407	JBMCGNCK_03494	K01972	Genetic information processing	9120	E6.5.1.2, ligA, ligB; DNA ligase (NAD+) [EC:6.5.1.2]	391
NZ_CP022121.1_1263	JBMCGNCK_01304	K02316	Genetic information processing	9120	dnaG; DNA primase [EC:2.7.7.101]	228
NZ_CP022121.1_2856	JBMCGNCK_02930	K02335	Genetic information processing	9120	polA; DNA polymerase I [EC:2.7.7.7]	432
NZ_CP022121.1_686	JBMCGNCK_00707	K02338	Genetic information processing	9120	dnaN; DNA polymerase III subunit beta [EC:2.7.7.7]	202
NZ_CP022121.1_500	JBMCGNCK_00512	K02343	Genetic information processing	9120	dnaX; DNA polymerase III subunit gamma/tau [EC:2.7.7.7]	201
NZ_CP022121.1_2392	JBMCGNCK_02455	K02433	Genetic information processing	9120	RSL1; aspartyl-tRNA(Asn)/glutamyl-tRNA(Gln) amidotransferase subunit A [EC:6.3.5.6	382
NZ_CP022121.1_3389	JBMCGNCK_03476	K02433	Genetic information processing	9120	RSL1; aspartyl-tRNA(Asn)/glutamyl-tRNA(Gln) amidotransferase subunit A [EC:6.3.5.6	240
NZ_CP022121.1_3388	JBMCGNCK_03475	K02434	Genetic information processing	9120	T112; aspartyl-tRNA(Asn)/glutamyl-tRNA(Gln) amidotransferase subunit B [EC:6.3.5.6	369
NZ_CP022121.1_2393	JBMCGNCK_02456	K02434	Genetic information processing	9120	T112; aspartyl-tRNA(Asn)/glutamyl-tRNA(Gln) amidotransferase subunit B [EC:6.3.5.6	344
NZ_CP022121.1_3390	JBMCGNCK_03477	K02435	Genetic information processing	9120	ATC; aspartyl-tRNA(Asn)/glutamyl-tRNA(Gln) amidotransferase subunit C [EC:6.3.5.6	60
NZ_CP022121.1_1102	JBMCGNCK_01143	K02863	Genetic information processing	9120	RP-L1, MRPL1, rplA; large subunit ribosomal protein L1	209
NZ_CP022121.1_1103	JBMCGNCK_01144	K02864	Genetic information processing	9120	RP-L10, MRPL10, rplJ; large subunit ribosomal protein L10	114
NZ_CP022121.1_1101	JBMCGNCK_01142	K02867	Genetic information processing	9120	RP-L11, MRPL11, rplK; large subunit ribosomal protein L11	115
NZ_CP022121.1_1148	JBMCGNCK_01189	K02871	Genetic information processing	9120	RP-L13, MRPL13, rplM; large subunit ribosomal protein L13	139
NZ_CP022121.1_1123	JBMCGNCK_01164	K02874	Genetic information processing	9120	RP-L14, MRPL14, rplN; large subunit ribosomal protein L14	101
NZ_CP022121.1_1132	JBMCGNCK_01173	K02876	Genetic information processing	9120	RP-L15, MRPL15, rplO; large subunit ribosomal protein L15	132
NZ_CP022121.1_1120	JBMCGNCK_01161	K02878	Genetic information processing	9120	RP-L16, MRPL16, rplP; large subunit ribosomal protein L16	150
NZ_CP022121.1_1143	JBMCGNCK_01184	K02879	Genetic information processing	9120	RP-L17, MRPL17, rplQ; large subunit ribosomal protein L17	91
NZ_CP022121.1_1129	JBMCGNCK_01170	K02881	Genetic information processing	9120	RP-L18, MRPL18, rplR; large subunit ribosomal protein L18	94
NZ_CP022121.1_1116	JBMCGNCK_01157	K02886	Genetic information processing	9120	RP-L2, MRPL2, rplB; large subunit ribosomal protein L2	216
NZ_CP022121.1_196	JBMCGNCK_00198	K02888	Genetic information processing	9120	RP-L21, MRPL21, rplU; large subunit ribosomal protein L21	85
NZ_CP022121.1_1118	JBMCGNCK_01159	K02890	Genetic information processing	9120	RP-L22, MRPL22, rplV; large subunit ribosomal protein L22	75
NZ_CP022121.1_1115	JBMCGNCK_01156	K02892	Genetic information processing	9120	RP-L23, MRPL23, rplW; large subunit ribosomal protein L23	65
NZ_CP022121.1_1124	JBMCGNCK_01165	K02895	Genetic information processing	9120	RP-L24, MRPL24, rplX; large subunit ribosomal protein L24	67
NZ_CP022121.1_852	JBMCGNCK_00878	K02897	Genetic information processing	9120	RP-L25, rplY; large subunit ribosomal protein L25	44
NZ_CP022121.1_195	JBMCGNCK_00197	K02899	Genetic information processing	9120	RP-L27, MRPL27, rpmA; large subunit ribosomal protein L27	81
NZ_CP022121.1_1121	JBMCGNCK_01162	K02904	Genetic information processing	9120	RP-L29, rpmC; large subunit ribosomal protein L29	40
NZ_CP022121.1_1113	JBMCGNCK_01154	K02906	Genetic information processing	9120	RP-L3, MRPL3, rplC; large subunit ribosomal protein L3	184
NZ_CP022121.1_1131	JBMCGNCK_01172	K02907	Genetic information processing	9120	RP-L30, MRPL30, rpmD; large subunit ribosomal protein L30	51
NZ_CP022121.1_895	JBMCGNCK_00922	K02909	Genetic information processing	9120	RP-L31, rpmE; large subunit ribosomal protein L31	51
NZ_CP022121.1_1114	JBMCGNCK_01155	K02926	Genetic information processing	9120	RP-L4, MRPL4, rplD; large subunit ribosomal protein L4	164
NZ_CP022121.1_1125	JBMCGNCK_01166	K02931	Genetic information processing	9120	RP-L5, MRPL5, rplE; large subunit ribosomal protein L5	144
NZ_CP022121.1_1128	JBMCGNCK_01169	K02933	Genetic information processing	9120	RP-L6, MRPL6, rplF; large subunit ribosomal protein L6	132
NZ_CP022121.1_1104	JBMCGNCK_01145	K02935	Genetic information processing	9120	RP-L7, MRPL12, rplL; large subunit ribosomal protein L7/L12	106
NZ_CP022121.1_788	JBMCGNCK_00809	K02939	Genetic information processing	9120	RP-L9, MRPL9, rplI; large subunit ribosomal protein L9	74
NZ_CP022121.1_1112	JBMCGNCK_01153	K02946	Genetic information processing	9120	RP-S10, MRPS10, rpsJ; small subunit ribosomal protein S10	93
NZ_CP022121.1_1140	JBMCGNCK_01181	K02948	Genetic information processing	9120	RP-S11, MRPS11, rpsK; small subunit ribosomal protein S11	120
NZ_CP022121.1_1108	JBMCGNCK_01149	K02950	Genetic information processing	9120	RP-S12, MRPS12, rpsL; small subunit ribosomal protein S12	106
NZ_CP022121.1_1139	JBMCGNCK_01180	K02952	Genetic information processing	9120	RP-S13, rpsM; small subunit ribosomal protein S13	103
NZ_CP022121.1_2566	JBMCGNCK_02631	K02956	Genetic information processing	9120	RP-S15, MRPS15, rpsO; small subunit ribosomal protein S15	85
NZ_CP022121.1_1385	JBMCGNCK_01436	K02959	Genetic information processing	9120	RP-S16, MRPS16, rpsP; small subunit ribosomal protein S16	88
NZ_CP022121.1_1122	JBMCGNCK_01163	K02961	Genetic information processing	9120	RP-S17, MRPS17, rpsQ; small subunit ribosomal protein S17	77
NZ_CP022121.1_785	JBMCGNCK_00806	K02963	Genetic information processing	9120	RP-S18, MRPS18, rpsR; small subunit ribosomal protein S18	39
NZ_CP022121.1_1117	JBMCGNCK_01158	K02965	Genetic information processing	9120	RP-S19, rpsS; small subunit ribosomal protein S19	110
NZ_CP022121.1_1941	JBMCGNCK_01998	K02967	Genetic information processing	9120	RP-S2, MRPS2, rpsB; small subunit ribosomal protein S2	223
NZ_CP022121.1_71	JBMCGNCK_00072	K02968	Genetic information processing	9120	RP-S20, rpsT; small subunit ribosomal protein S20	39
NZ_CP022121.1_54	JBMCGNCK_00055	K02970	Genetic information processing	9120	RP-S21, MRPS21, rpsU; small subunit ribosomal protein S21	68
NZ_CP022121.1_1119	JBMCGNCK_01160	K02982	Genetic information processing	9120	RP-S3, rpsC; small subunit ribosomal protein S3	163
NZ_CP022121.1_1141	JBMCGNCK_01182	K02986	Genetic information processing	9120	RP-S4, rpsD; small subunit ribosomal protein S4	178
NZ_CP022121.1_1130	JBMCGNCK_01171	K02988	Genetic information processing	9120	RP-S5, MRPS5, rpsE; small subunit ribosomal protein S5	149
NZ_CP022121.1_783	JBMCGNCK_00804	K02990	Genetic information processing	9120	RP-S6, MRPS6, rpsF; small subunit ribosomal protein S6	69
NZ_CP022121.1_1109	JBMCGNCK_01150	K02992	Genetic information processing	9120	RP-S7, MRPS7, rpsG; small subunit ribosomal protein S7	172
NZ_CP022121.1_1127	JBMCGNCK_01168	K02994	Genetic information processing	9120	RP-S8, rpsH; small subunit ribosomal protein S8	151
NZ_CP022121.1_1149	JBMCGNCK_01190	K02996	Genetic information processing	9120	RP-S9, MRPS9, rpsI; small subunit ribosomal protein S9	133
NZ_CP022121.1_1142	JBMCGNCK_01183	K03040	Genetic information processing	9120	rpoA; DNA-directed RNA polymerase subunit alpha [EC:2.7.7.6]	233
NZ_CP022121.1_1105	JBMCGNCK_01146	K03043	Genetic information processing	9120	rpoB; DNA-directed RNA polymerase subunit beta [EC:2.7.7.6]	844
NZ_CP022121.1_1106	JBMCGNCK_01147	K03046	Genetic information processing	9120	rpoC; DNA-directed RNA polymerase subunit beta' [EC:2.7.7.6]	912
NZ_CP022121.1_751	JBMCGNCK_00773	K03100	Genetic information processing	9120	lepB; signal peptidase I [EC:3.4.21.89]	72
NZ_CP022121.1_784	JBMCGNCK_00805	K03111	Genetic information processing	9120	ssb; single-strand DNA-binding protein	71
NZ_CP022121.1_2587	JBMCGNCK_02652	K03553	Genetic information processing	9120	recA; recombination protein RecA	248
NZ_CP022121.1_2461	JBMCGNCK_02525	K03602	Genetic information processing	9120	xseB; exodeoxyribonuclease VII small subunit [EC:3.1.11.6]	21
NZ_CP022121.1_892	JBMCGNCK_00919	K03628	Genetic information processing	9120	rho; transcription termination factor Rho	295
NZ_CP022121.1_984	JBMCGNCK_01012	K03701	Genetic information processing	9120	uvrA; excinuclease ABC subunit A	692
NZ_CP022121.1_2106	JBMCGNCK_02170	K04085	Genetic information processing	9120	tusA, sirA; tRNA 2-thiouridine synthesizing protein A [EC:2.8.1.-]	29
NZ_CP022121.1_1066	JBMCGNCK_01096	K04567	Genetic information processing	9120	KARS, lysS; lysyl-tRNA synthetase, class II [EC:6.1.1.6]	345
NZ_CP022121.1_859	JBMCGNCK_00885	K05592	Genetic information processing	9120	deaD, cshA; ATP-dependent RNA helicase DeaD [EC:3.6.4.13]	297
NZ_CP022121.1_1107	JBMCGNCK_01148	K07590	Genetic information processing	9120	RP-L7A, rplGB; large subunit ribosomal protein L7A	35
NZ_CP022121.1_350	JBMCGNCK_00358	K12573	Genetic information processing	9120	rnr, vacB; ribonuclease R [EC:3.1.13.1]	383
NZ_CP022121.1_2577	JBMCGNCK_02642	K12574	Genetic information processing	9120	rnj; ribonuclease J [EC:3.1.-.-]	403
NZ_CP022121.1_548	JBMCGNCK_00562	K13993	Genetic information processing	9120	HSP20; HSP20 family protein	59
NZ_CP022121.1_2589	JBMCGNCK_02654	K18682	Genetic information processing	9120	rny; ribonucrease Y [EC:3.1.-.-]	367
NZ_CP022121.1_835	JBMCGNCK_00861	K00937	Genetic information processing, Energy metabolism	9120, 9102	ppk1; polyphosphate kinase [EC:2.7.4.1]	240
NZ_CP022121.1_3587	JBMCGNCK_03675	K03072	Genetic information processing, Environmental information processing	9120, 9130	secD; preprotein translocase subunit SecD	158
NZ_CP022121.1_3588	JBMCGNCK_03676	K03074	Genetic information processing, Environmental information processing	9120, 9130	secF; preprotein translocase subunit SecF	175
NZ_CP022121.1_1384	JBMCGNCK_01435	K03106	Genetic information processing, Environmental information processing	9120, 9130	SRP54, ffh; signal recognition particle subunit SRP54 [EC:3.6.5.4]	307
NZ_CP022121.1_957	JBMCGNCK_00984	K03070	Genetic information processing, Environmental information processing, Cellular processes	9120, 9130, 9140	secA; preprotein translocase subunit SecA [EC:7.4.2.8]	621
NZ_CP022121.1_355	JBMCGNCK_00363	K03075	Genetic information processing, Environmental information processing, Cellular processes	9120, 9130, 9140	secG; preprotein translocase subunit SecG	52
NZ_CP022121.1_1133	JBMCGNCK_01174	K03076	Genetic information processing, Environmental information processing, Cellular processes	9120, 9130, 9140	secY; preprotein translocase subunit SecY	287
NZ_CP022121.1_1373	JBMCGNCK_01424	K03110	Genetic information processing, Environmental information processing, Cellular processes	9120, 9130, 9140	ftsY; fused signal recognition particle receptor	164
NZ_CP022121.1_2702	JBMCGNCK_02768	K03210	Genetic information processing, Environmental information processing, Cellular processes	9120, 9130, 9140	yajC; preprotein translocase subunit YajC	33
NZ_CP022121.1_689	JBMCGNCK_00710	K03217	Genetic information processing, Environmental information processing, Cellular processes	9120, 9130, 9140	yidC, spoIIIJ, OXA1, ccfA; YidC/Oxa1 family membrane protein insertase	104
NZ_CP022121.1_872	JBMCGNCK_00899	K04079	on processing, Environmental information processing, Cellular processes, Organismal system	20, 9130, 9140, 9150, 9	HSP90A, htpG; molecular chaperone HtpG	382
NZ_CP022121.1_1366	JBMCGNCK_01417	K03685	Genetic information processing, Human diseases	9120, 9160	rnc, DROSHA, RNT1; ribonuclease III [EC:3.1.26.3]	104
NZ_CP022121.1_1312	JBMCGNCK_01363	K01885	Genetic information processing, Metabolism of cofactors and vitamins	9120, 9108	EARS, gltX; glutamyl-tRNA synthetase [EC:6.1.1.17]	283
NZ_CP022121.1_3413	JBMCGNCK_03500	K03639	Genetic information processing, Metabolism of cofactors and vitamins	9120, 9108	moaA, CNX2; GTP 3',8-cyclase [EC:4.1.99.22]	190
NZ_CP022121.1_3565	JBMCGNCK_03653	K04487	Genetic information processing, Metabolism of cofactors and vitamins	9120, 9108	iscS, NFS1; cysteine desulfurase [EC:2.8.1.7]	247
NZ_CP022121.1_701	JBMCGNCK_00722	K01042	Genetic information processing, Metabolism of other amino acids	9120, 9106	selA; L-seryl-tRNA(Ser) seleniumtransferase [EC:2.9.1.1]	229
NZ_CP022121.1_467	JBMCGNCK_00478	K01874	Genetic information processing, Metabolism of other amino acids	9120, 9106	MARS, metG; methionyl-tRNA synthetase [EC:6.1.1.10]	437
NZ_CP022121.1_60	JBMCGNCK_00061	K04043	Genetic information processing, Organismal systems, Human diseases	9120, 9150, 9160	dnaK, HSPA9; molecular chaperone DnaK	460
NZ_CP022121.1_425	JBMCGNCK_00435	K04043	Genetic information processing, Organismal systems, Human diseases	9120, 9150, 9160	dnaK, HSPA9; molecular chaperone DnaK	414
NZ_CP022121.1_1400	JBMCGNCK_01453	K04043	Genetic information processing, Organismal systems, Human diseases	9120, 9150, 9160	dnaK, HSPA9; molecular chaperone DnaK	189
NZ_CP022121.1_245	JBMCGNCK_00253	K04077	Genetic information processing, Organismal systems, Human diseases	9120, 9150, 9160	groEL, HSPD1; chaperonin GroEL	391
NZ_CP022121.1_2600	JBMCGNCK_02665	K01005	Glycan biosynthesis and metabolism	9107	gT_U_V; polyisoprenyl-teichoic acid--peptidoglycan teichoic acid transferase [EC:2.7.8	121
NZ_CP022121.1_929	JBMCGNCK_00956	K01005	Glycan biosynthesis and metabolism	9107	gT_U_V; polyisoprenyl-teichoic acid--peptidoglycan teichoic acid transferase [EC:2.7.8	101
NZ_CP022121.1_1670	JBMCGNCK_01723	K01924	Glycan biosynthesis and metabolism	9107	murC; UDP-N-acetylmuramate--alanine ligase [EC:6.3.2.8]	230
NZ_CP022121.1_2515	JBMCGNCK_02579	K07258	Glycan biosynthesis and metabolism	9107	A, dacD; serine-type D-Ala-D-Ala carboxypeptidase (penicillin-binding protein 5/6) [EC	142
NZ_CP022121.1_1664	JBMCGNCK_01717	K08384	Glycan biosynthesis and metabolism	9107	spoVD; stage V sporulation protein D (sporulation-specific penicillin-binding protein)	180
NZ_CP022121.1_884	JBMCGNCK_00911	K11693	Glycan biosynthesis and metabolism	9107	mX, fmhB; peptidoglycan pentaglycine glycine transferase (the first glycine) [EC:2.3.2.1	165
NZ_CP022121.1_2757	JBMCGNCK_02825	K19068	Glycan biosynthesis and metabolism	9107	wbjC; UDP-2-acetamido-2,6-beta-L-arabino-hexul-4-ose reductase [EC:1.1.1.367]	194
NZ_CP022121.1_2721	JBMCGNCK_02788	K05366	Glycan biosynthesis and metabolism, Human diseases	9107, 9160	mrcA; penicillin-binding protein 1A [EC:2.4.1.129 3.4.16.4]	322
NZ_CP022121.1_206	JBMCGNCK_00208	K05515	Glycan biosynthesis and metabolism, Human diseases	9107, 9160	mrdA; penicillin-binding protein 2 [EC:3.4.16.4]	248
NZ_CP022121.1_3066	JBMCGNCK_03148	K01448	Human diseases	9160	amiABC; N-acetylmuramoyl-L-alanine amidase [EC:3.5.1.28]	75
NZ_CP022121.1_2054	JBMCGNCK_02116	K01448	Human diseases	9160	amiABC; N-acetylmuramoyl-L-alanine amidase [EC:3.5.1.28]	21
NZ_CP022121.1_1302	JBMCGNCK_01347	K02171	Human diseases	9160	blaI; BlaI family transcriptional regulator, penicillinase repressor	10

NZ_CP022121.1_66	JBMCGNCK_00067	K03596	Human diseases	9160	lepA; GTP-binding protein LepA	463
NZ_CP022121.1_1223	JBMCGNCK_01264	K07755	Human diseases	9160	AS3MT; arsenite methyltransferase [EC:2.1.1.137]	107
NZ_CP022121.1_1775	JBMCGNCK_01829	K14475	Human diseases	9160	ICP; inhibitor of cysteine peptidase	57
NZ_CP022121.1_2548	JBMCGNCK_02612	K00057	Lipid metabolism	9103	gpsA; glycerol-3-phosphate dehydrogenase (NAD(P)+) [EC:1.1.1.94]	189
NZ_CP022121.1_1361	JBMCGNCK_01412	K00645	Lipid metabolism	9103	fabD, MCAT, MCT1; [acyl-carrier-protein] S-malonyltransferase [EC:2.3.1.39]	183
NZ_CP022121.1_1359	JBMCGNCK_01410	K00648	Lipid metabolism	9103	fabH; 3-oxoacyl-[acyl-carrier-protein] synthase III [EC:2.3.1.180]	212
NZ_CP022121.1_3047	JBMCGNCK_03128	K00648	Lipid metabolism	9103	fabH; 3-oxoacyl-[acyl-carrier-protein] synthase III [EC:2.3.1.180]	174
NZ_CP022121.1_3046	JBMCGNCK_03127	K00648	Lipid metabolism	9103	fabH; 3-oxoacyl-[acyl-carrier-protein] synthase III [EC:2.3.1.180]	133
NZ_CP022121.1_2902	JBMCGNCK_02978	K01613	Lipid metabolism	9103	psd, PISD; phosphatidylserine decarboxylase [EC:4.1.1.65]	137
NZ_CP022121.1_1363	JBMCGNCK_01414	K02078	Lipid metabolism	9103	acpP; acyl carrier protein	45
NZ_CP022121.1_1360	JBMCGNCK_01411	K02371	Lipid metabolism	9103	fabK; enoyl-[acyl-carrier protein] reductase II [EC:1.3.1.9]	194
NZ_CP022121.1_1358	JBMCGNCK_01409	K03621	Lipid metabolism	9103	plsX; phosphate acyltransferase [EC:2.3.1.274]	173
NZ_CP022121.1_270	JBMCGNCK_00278	K00231	Metabolism of cofactors and vitamins	9108	PPOX, hemY; protoporphyrinogen/coproporphyrinogen III oxidase [EC:1.3.3.4 1.3.3.15]	184
NZ_CP022121.1_1901	JBMCGNCK_01958	K00323	Metabolism of cofactors and vitamins	9108	NNT; H+-translocating NAD(P) transhydrogenase [EC:1.6.1.2 7.1.1.1]	82
NZ_CP022121.1_1899	JBMCGNCK_01956	K00324	Metabolism of cofactors and vitamins	9108	pntA; H+-translocating NAD(P) transhydrogenase subunit alpha [EC:1.6.1.2 7.1.1.1]	259
NZ_CP022121.1_1318	JBMCGNCK_01369	K00763	Metabolism of cofactors and vitamins	9108	pncB, NAPRT1; nicotinate phosphoribosyltransferase [EC:6.3.4.21]	320
NZ_CP022121.1_1311	JBMCGNCK_01362	K00768	Metabolism of cofactors and vitamins	9108	obU, cobT; nicotinate-nucleotide--dimethylbenzimidazole phosphoribosyltransferase [	232
NZ_CP022121.1_1893	JBMCGNCK_01950	K00794	Metabolism of cofactors and vitamins	9108	ribH, RIB4; 6,7-dimethyl-8-ribityllumazine synthase [EC:2.5.1.78]	157
NZ_CP022121.1_1048	JBMCGNCK_01078	K00796	Metabolism of cofactors and vitamins	9108	folP; dihydropteroate synthase [EC:2.5.1.15]	214
NZ_CP022121.1_2466	JBMCGNCK_02530	K00858	Metabolism of cofactors and vitamins	9108	ppnK, NADK; NAD+ kinase [EC:2.7.1.23]	138
NZ_CP022121.1_2286	JBMCGNCK_02355	K00868	Metabolism of cofactors and vitamins	9108	pdxK, pdxY; pyridoxine kinase [EC:2.7.1.35]	141
NZ_CP022121.1_1050	JBMCGNCK_01080	K00950	Metabolism of cofactors and vitamins	9108	olK; 2-amino-4-hydroxy-6-hydroxymethyldihydropteridine diphosphokinase [EC:2.7.6.3	102
NZ_CP022121.1_2897	JBMCGNCK_02973	K01495	Metabolism of cofactors and vitamins	9108	GCH1, folE; GTP cyclohydrolase IA [EC:3.5.4.16]	178
NZ_CP022121.1_272	JBMCGNCK_00280	K01599	Metabolism of cofactors and vitamins	9108	hemE, UROD; uroporphyrinogen decarboxylase [EC:4.1.1.37]	291
NZ_CP022121.1_1863	JBMCGNCK_01920	K01599	Metabolism of cofactors and vitamins	9108	hemE, UROD; uroporphyrinogen decarboxylase [EC:4.1.1.37]	207
NZ_CP022121.1_2404	JBMCGNCK_02467	K01599	Metabolism of cofactors and vitamins	9108	hemE, UROD; uroporphyrinogen decarboxylase [EC:4.1.1.37]	201
NZ_CP022121.1_3232	JBMCGNCK_03313	K01599	Metabolism of cofactors and vitamins	9108	hemE, UROD; uroporphyrinogen decarboxylase [EC:4.1.1.37]	105
NZ_CP022121.1_641	JBMCGNCK_00656	K01599	Metabolism of cofactors and vitamins	9108	hemE, UROD; uroporphyrinogen decarboxylase [EC:4.1.1.37]	68
NZ_CP022121.1_1865	JBMCGNCK_01922	K01599	Metabolism of cofactors and vitamins	9108	hemE, UROD; uroporphyrinogen decarboxylase [EC:4.1.1.37]	25
NZ_CP022121.1_2016	JBMCGNCK_02075	K01599	Metabolism of cofactors and vitamins	9108	hemE, UROD; uroporphyrinogen decarboxylase [EC:4.1.1.37]	24
NZ_CP022121.1_643	JBMCGNCK_00658	K01599	Metabolism of cofactors and vitamins	9108	hemE, UROD; uroporphyrinogen decarboxylase [EC:4.1.1.37]	18
NZ_CP022121.1_1049	JBMCGNCK_01079	K01633	Metabolism of cofactors and vitamins	9108	B; 7,8-dihydroneopterin aldolase/epimerase/oxygenase [EC:4.1.2.25 5.1.99.8 1.13.11.8	55
NZ_CP022121.1_3056	JBMCGNCK_03137	K01698	Metabolism of cofactors and vitamins	9108	hemB, ALAD; porphobilinogen synthase [EC:4.2.1.24]	319
NZ_CP022121.1_3057	JBMCGNCK_03138	K01749	Metabolism of cofactors and vitamins	9108	hemC, HMBS; hydroxymethylbilane synthase [EC:2.5.1.61]	240
NZ_CP022121.1_3054	JBMCGNCK_03135	K01845	Metabolism of cofactors and vitamins	9108	hemL; glutamate-1-semialdehyde 2,1-aminomutase [EC:5.4.3.8]	297
NZ_CP022121.1_1188	JBMCGNCK_01228	K01950	Metabolism of cofactors and vitamins	9108	E6.3.5.1, NADSYN1, QNS1, nadE; NAD+ synthase (glutamine-hydrolysing) [EC:6.3.5.1]	363
NZ_CP022121.1_3509	JBMCGNCK_03595	K02188	Metabolism of cofactors and vitamins	9108	cbiD; cobalt-precorrin-5B (C1)-methyltransferase [EC:2.1.1.195]	225
NZ_CP022121.1_3502	JBMCGNCK_03588	K02189	Metabolism of cofactors and vitamins	9108	cbiG; cobalt-precorrin 5A hydrolase [EC:3.7.1.12]	39
NZ_CP022121.1_3507	JBMCGNCK_03593	K02191	Metabolism of cofactors and vitamins	9108	cbiT; cobalt-precorrin-6B (C15)-methyltransferase [EC:2.1.1.196]	71
NZ_CP022121.1_3511	JBMCGNCK_03597	K02224	Metabolism of cofactors and vitamins	9108	cobB-cbiA; cobyrinic acid a,c-diamide synthase [EC:6.3.5.9 6.3.5.11]	206
NZ_CP022121.1_3515	JBMCGNCK_03601	K02226	Metabolism of cofactors and vitamins	9108	cobC, phpB; alpha-ribazole phosphatase [EC:3.1.3.73]	76
NZ_CP022121.1_3513	JBMCGNCK_03599	K02227	Metabolism of cofactors and vitamins	9108	cbiB, cobD; adenosylcobinamide-phosphate synthase [EC:6.3.1.10]	172
NZ_CP022121.1_3516	JBMCGNCK_03602	K02231	Metabolism of cofactors and vitamins	9108	enosylcobinamide kinase / adenosylcobinamide-phosphate guanylyltransferase [EC:2.7	88
NZ_CP022121.1_3514	JBMCGNCK_03600	K02232	Metabolism of cofactors and vitamins	9108	cobQ, cbiP; adenosylcobyric acid synthase [EC:6.3.5.10]	280
NZ_CP022121.1_3535	JBMCGNCK_03622	K02492	Metabolism of cofactors and vitamins	9108	hemA; glutamyl-tRNA reductase [EC:1.2.1.70]	151
NZ_CP022121.1_3506	JBMCGNCK_03592	K03394	Metabolism of cofactors and vitamins	9108	cobI-cbiL; precorrin-2/cobalt-factor-2 C20-methyltransferase [EC:2.1.1.130 2.1.1.151]	69
NZ_CP022121.1_3508	JBMCGNCK_03594	K03399	Metabolism of cofactors and vitamins	9108	cbiE; cobalt-precorrin-7 (C5)-methyltransferase [EC:2.1.1.289]	109
NZ_CP022121.1_1060	JBMCGNCK_01090	K03525	Metabolism of cofactors and vitamins	9108	coaX; type III pantothenate kinase [EC:2.7.1.33]	238
NZ_CP022121.1_3414	JBMCGNCK_03501	K03750	Metabolism of cofactors and vitamins	9108	moeA; molybdopterin molybdotransferase [EC:2.10.1.1]	183
NZ_CP022121.1_3536	JBMCGNCK_03623	K03794	Metabolism of cofactors and vitamins	9108	sirB; sirohydrochlorin ferrochelatase [EC:4.99.1.4]	14
NZ_CP022121.1_3458	JBMCGNCK_03544	K03800	Metabolism of cofactors and vitamins	9108	lplA, lplJ, lipL1; lipoate---protein ligase [EC:6.3.1.20]	169
NZ_CP022121.1_3512	JBMCGNCK_03598	K04720	Metabolism of cofactors and vitamins	9108	cobD; threonine-phosphate decarboxylase [EC:4.1.1.81]	118
NZ_CP022121.1_3500	JBMCGNCK_03586	K05895	Metabolism of cofactors and vitamins	9108	cobK-cbiJ; precorrin-6A/cobalt-precorrin-6A reductase [EC:1.3.1.54 1.3.1.106]	83
NZ_CP022121.1_3501	JBMCGNCK_03587	K05934	Metabolism of cofactors and vitamins	9108	precorrin-3B C17-methyltransferase / cobalt-factor III methyltransferase [EC:2.1.1.131	163
NZ_CP022121.1_3504	JBMCGNCK_03590	K05936	Metabolism of cofactors and vitamins	9108	bM, cbiF; precorrin-4/cobalt-precorrin-4 C11-methyltransferase [EC:2.1.1.133 2.1.1.27	160
NZ_CP022121.1_1535	JBMCGNCK_01588	K05936	Metabolism of cofactors and vitamins	9108	bM, cbiF; precorrin-4/cobalt-precorrin-4 C11-methyltransferase [EC:2.1.1.133 2.1.1.27	82
NZ_CP022121.1_3510	JBMCGNCK_03596	K06042	Metabolism of cofactors and vitamins	9108	cobH-cbiC; precorrin-8X/cobalt-precorrin-8 methylmutase [EC:5.4.99.61 5.4.99.60]	124
NZ_CP022121.1_461	JBMCGNCK_00472	K06897	Metabolism of cofactors and vitamins	9108	hydropterin-6-yl-methyl-4-(beta-D-ribofuranosyl)aminobenzene 5'-phosphate synthase	175
NZ_CP022121.1_1741	JBMCGNCK_01794	K07141	Metabolism of cofactors and vitamins	9108	mocA; molybdenum cofactor cytidylyltransferase [EC:2.7.7.76]	36
NZ_CP022121.1_3594	JBMCGNCK_03682	K08260	Metabolism of cofactors and vitamins	9108	cbiZ; adenosylcobinamide hydrolase [EC:3.5.1.90]	25
NZ_CP022121.1_2771	JBMCGNCK_02839	K09457	Metabolism of cofactors and vitamins	9108	queF; 7-cyano-7-deazaguanine reductase [EC:1.7.1.13]	103
NZ_CP022121.1_2898	JBMCGNCK_02974	K10026	Metabolism of cofactors and vitamins	9108	queE; 7-carboxy-7-deazaguanine synthase [EC:4.3.99.3]	103
NZ_CP022121.1_212	JBMCGNCK_00214	K11754	Metabolism of cofactors and vitamins	9108	folC; dihydrofolate synthase / folylpolyglutamate synthase [EC:6.3.2.12 6.3.2.17]	214
NZ_CP022121.1_3533	JBMCGNCK_03620	K13542	Metabolism of cofactors and vitamins	9108	cobA-hemD; uroporphyrinogen III methyltransferase / synthase [EC:2.1.1.107 4.2.1.75	272
NZ_CP022121.1_1891	JBMCGNCK_01948	K14652	Metabolism of cofactors and vitamins	9108	,4-dihydroxy 2-butanone 4-phosphate synthase / GTP cyclohydrolase II [EC:4.1.99.12 3	354
NZ_CP022121.1_2522	JBMCGNCK_02586	K19221	Metabolism of cofactors and vitamins	9108	cobA, btuR; cob(I)alamin adenosyltransferase [EC:2.5.1.17]	85
NZ_CP022121.1_760	JBMCGNCK_00782	K22465	Metabolism of cofactors and vitamins	9108	bzaA_B; 5-hydroxybenzimidazole synthase [EC:4.1.99.23]	403
NZ_CP022121.1_759	JBMCGNCK_00781	K22465	Metabolism of cofactors and vitamins	9108	bzaA_B; 5-hydroxybenzimidazole synthase [EC:4.1.99.23]	313
NZ_CP022121.1_764	JBMCGNCK_00786	K25498	Metabolism of cofactors and vitamins	9108	bzaD; 5-methoxybenzimidazole methyltransferase	262
NZ_CP022121.1_2918	JBMCGNCK_02995	K00297	Metabolism of cofactors and vitamins, Energy metabolism	9108, 9102	metF, MTHFR; methylenetetrahydrofolate reductase (NADH) [EC:1.5.1.54]	353
NZ_CP022121.1_3166	JBMCGNCK_03246	K01491	Metabolism of cofactors and vitamins, Energy metabolism	9108, 9102	etrahydrofolate dehydrogenase (NADP+) / methenyltetrahydrofolate cyclohydrolase [E	196
NZ_CP022121.1_3167	JBMCGNCK_03247	K01500	Metabolism of cofactors and vitamins, Energy metabolism	9108, 9102	fchA; methenyltetrahydrofolate cyclohydrolase [EC:3.5.4.9]	119
NZ_CP022121.1_2364	JBMCGNCK_02433	K00287	Metabolism of cofactors and vitamins, Human diseases	9108, 9160	DHFR, folA; dihydrofolate reductase [EC:1.5.1.3]	75
NZ_CP022121.1_1362	JBMCGNCK_01413	K00059	Metabolism of cofactors and vitamins, Lipid metabolism	9108, 9103	fabG, OAR1; 3-oxoacyl-[acyl-carrier protein] reductase [EC:1.1.1.100]	147
NZ_CP022121.1_1365	JBMCGNCK_01416	K09458	Metabolism of cofactors and vitamins, Lipid metabolism	9108, 9103	fabF, OXSM, CEM1; 3-oxoacyl-[acyl-carrier-protein] synthase II [EC:2.3.1.179]	295
NZ_CP022121.1_2013	JBMCGNCK_02071	K01579	Metabolism of cofactors and vitamins, Metabolism of other amino acids	9108, 9106	panD; aspartate 1-decarboxylase [EC:4.1.1.11]	81
NZ_CP022121.1_2012	JBMCGNCK_02070	K01918	Metabolism of cofactors and vitamins, Metabolism of other amino acids	9108, 9106	panC; pantoate--beta-alanine ligase [EC:6.3.2.1]	178
NZ_CP022121.1_2464	JBMCGNCK_02528	K01662	Metabolism of cofactors and vitamins, Metabolism of terpenoids and polyketides	9108, 9109	dxs; 1-deoxy-D-xylulose-5-phosphate synthase [EC:2.2.1.7]	388
NZ_CP022121.1_1134	JBMCGNCK_01175	K00939	Metabolism of cofactors and vitamins, Nucleotide metabolism	9108, 9104	adk, AK; adenylate kinase [EC:2.7.4.3]	145
NZ_CP022121.1_2478	JBMCGNCK_02542	K01515	Metabolism of cofactors and vitamins, Nucleotide metabolism	9108, 9104	nudF; ADP-ribose pyrophosphatase [EC:3.6.1.13 3.6.1.-]	102
NZ_CP022121.1_2894	JBMCGNCK_02970	K03787	Metabolism of cofactors and vitamins, Nucleotide metabolism	9108, 9104	surE; 5'/3'-nucleotidase [EC:3.1.3.5 3.1.3.6]	192
NZ_CP022121.1_3465	JBMCGNCK_03551	K00602	Metabolism of cofactors and vitamins, Nucleotide metabolism, Human diseases	9108, 9104, 9160	horibosylaminoimidazolecarboxamide formyltransferase / IMP cyclohydrolase [EC:2.1.	368
NZ_CP022121.1_1236	JBMCGNCK_01277	K00384	Metabolism of other amino acids	9106	trxB, TRR; thioredoxin reductase (NADPH) [EC:1.8.1.9]	209
NZ_CP022121.1_42	JBMCGNCK_00043	K00384	Metabolism of other amino acids	9106	trxB, TRR; thioredoxin reductase (NADPH) [EC:1.8.1.9]	204
NZ_CP022121.1_819	JBMCGNCK_00845	K00384	Metabolism of other amino acids	9106	trxB, TRR; thioredoxin reductase (NADPH) [EC:1.8.1.9]	166
NZ_CP022121.1_776	JBMCGNCK_00798	K01008	Metabolism of other amino acids	9106	selD, SEPHS; selenide, water dikinase [EC:2.7.9.3]	119
NZ_CP022121.1_2789	JBMCGNCK_02858	K03851	Metabolism of other amino acids	9106	tpa; taurine-pyruvate aminotransferase [EC:2.6.1.77]	268
NZ_CP022121.1_1902	JBMCGNCK_01959	K03852	Metabolism of other amino acids	9106	xsc; sulfoacetaldehyde acetyltransferase [EC:2.3.3.15]	391
NZ_CP022121.1_1435	JBMCGNCK_01489	K05306	Metabolism of other amino acids	9106	phnX; phosphonoacetaldehyde hydrolase [EC:3.11.1.1]	123
NZ_CP022121.1_1667	JBMCGNCK_01720	K01925	Metabolism of other amino acids, Glycan biosynthesis and metabolism	9106, 9107	murD; UDP-N-acetylmuramoylalanine--D-glutamate ligase [EC:6.3.2.9]	226
NZ_CP022121.1_1930	JBMCGNCK_01987	K01921	Metabolism of other amino acids, Glycan biosynthesis and metabolism, Human diseases	9106, 9107, 9160	ddl; D-alanine-D-alanine ligase [EC:6.3.2.4]	200
NZ_CP022121.1_258	JBMCGNCK_00266	K01775	Metabolism of other amino acids, Human diseases	9106, 9160	alr; alanine racemase [EC:5.1.1.1]	204
NZ_CP022121.1_1952	JBMCGNCK_02009	K03526	Metabolism of terpenoids and polyketides	9109	:pE, ispG; (E)-4-hydroxy-3-methylbut-2-enyl-diphosphate synthase [EC:1.17.7.1 1.17.7.	234
NZ_CP022121.1_3595	JBMCGNCK_03683	K00525	Nucleotide metabolism	9104	E1.17.4.1A, nrdA, nrdE; ribonucleoside-diphosphate reductase alpha chain [EC:1.17.4.1	450
NZ_CP022121.1_2485	JBMCGNCK_02549	K00756	Nucleotide metabolism	9104	pdp; pyrimidine-nucleoside phosphorylase [EC:2.4.2.2]	210
NZ_CP022121.1_848	JBMCGNCK_00874	K00760	Nucleotide metabolism	9104	hprT, hpt, HPRT1; hypoxanthine phosphoribosyltransferase [EC:2.4.2.8]	94
NZ_CP022121.1_906	JBMCGNCK_00933	K00761	Nucleotide metabolism	9104	upp, UPRT; uracil phosphoribosyltransferase [EC:2.4.2.9]	156
NZ_CP022121.1_1791	JBMCGNCK_01845	K00762	Nucleotide metabolism	9104	pyrE; orotate phosphoribosyltransferase [EC:2.4.2.10]	148
NZ_CP022121.1_838	JBMCGNCK_00864	K01524	Nucleotide metabolism	9104	polyphosphatase / guanosine-5'-triphosphate,3'-diphosphate pyrophosphatase [EC:3.6	195
NZ_CP022121.1_3470	JBMCGNCK_03556	K01588	Nucleotide metabolism	9104	purE; 5-(carboxyamino)imidazole ribonucleotide mutase [EC:5.4.99.18]	105
NZ_CP022121.1_1790	JBMCGNCK_01844	K01591	Nucleotide metabolism	9104	pyrF; orotidine-5'-phosphate decarboxylase [EC:4.1.1.23]	171
NZ_CP022121.1_3537	JBMCGNCK_03625	K01923	Nucleotide metabolism	9104	purC; phosphoribosylaminoimidazole-succinocarboxamide synthase [EC:6.3.2.6]	229
NZ_CP022121.1_1067	JBMCGNCK_01097	K01923	Nucleotide metabolism	9104	purC; phosphoribosylaminoimidazole-succinocarboxamide synthase [EC:6.3.2.6]	132
NZ_CP022121.1_3467	JBMCGNCK_03553	K01933	Nucleotide metabolism	9104	purM; phosphoribosylformylglycinamidine cyclo-ligase [EC:6.3.3.1]	208
NZ_CP022121.1_882	JBMCGNCK_00909	K01937	Nucleotide metabolism	9104	pyrG, CTPS; CTP synthase [EC:6.3.4.2]	404
NZ_CP022121.1_3464	JBMCGNCK_03550	K01945	Nucleotide metabolism	9104	purD; phosphoribosylamine---glycine ligase [EC:6.3.4.13]	277
NZ_CP022121.1_3396	JBMCGNCK_03483	K01952	Nucleotide metabolism	9104	PFAS, purL; phosphoribosylformylglycinamidine synthase [EC:6.3.5.3]	852
NZ_CP022121.1_1788	JBMCGNCK_01842	K02823	Nucleotide metabolism	9104	pyrDII; dihydroorotate dehydrogenase electron transfer subunit	113
NZ_CP022121.1_1943	JBMCGNCK_02000	K09903	Nucleotide metabolism	9104	pyrH; uridylate kinase [EC:2.7.4.22]	191
NZ_CP022121.1_1695	JBMCGNCK_01748	K21636	Nucleotide metabolism	9104	nrdD; ribonucleoside-triphosphate reductase (formate) [EC:1.1.98.6]	527
NZ_CP022121.1_1038	JBMCGNCK_01068	K00940	ide metabolism, Environmental information processing, Xenobiotics biodegradation and met	9104, 9130, 9111	ndk, NME; nucleoside-diphosphate kinase [EC:2.7.4.6]	132
NZ_CP022121.1_1411	JBMCGNCK_01464	K01951	icleotide metabolism, Protein families: metabolism, Xenobiotics biodegradation and metaboli	9104, 9181, 9111	guaA, GMPS; GMP synthase (glutamine-hydrolysing) [EC:6.3.5.2]	344
NZ_CP022121.1_2598	JBMCGNCK_02663	K00088	Nucleotide metabolism, Xenobiotics biodegradation and metabolism	9104, 9111	IMPDH, guaB; IMP dehydrogenase [EC:1.1.1.205]	369
NZ_CP022121.1_243	JBMCGNCK_00251	K00760	Nucleotide metabolism, Xenobiotics biodegradation and metabolism	9104, 9111	hprT, hpt, HPRT1; hypoxanthine phosphoribosyltransferase [EC:2.4.2.8]	130
NZ_CP022121.1_3242	JBMCGNCK_03323	K00876	Nucleotide metabolism, Xenobiotics biodegradation and metabolism	9104, 9111	udk, UCK; uridine kinase [EC:2.7.1.48]	265
NZ_CP022121.1_1202	JBMCGNCK_01242	K01489	Nucleotide metabolism, Xenobiotics biodegradation and metabolism	9104, 9111	cdd, CDA; cytidine deaminase [EC:3.5.4.5]	43
NZ_CP022121.1_1072	JBMCGNCK_01102	K01951	Nucleotide metabolism, Xenobiotics biodegradation and metabolism	9104, 9111	guaA, GMPS; GMP synthase (glutamine-hydrolysing) [EC:6.3.5.2]	295
NZ_CP022121.1_1111	JBMCGNCK_01152	K02358	Organismal systems	9150	tuf, TUFM; elongation factor Tu	346
NZ_CP022121.1_422	JBMCGNCK_00432	K03695	Organismal systems	9150	clpB; ATP-dependent Clp protease ATP-binding subunit ClpB	628
NZ_CP022121.1_820	JBMCGNCK_00846	K07213	Organismal systems	9150	ATOX1, ATX1, copZ, golB; copper chaperone	24
NZ_CP022121.1_2941	JBMCGNCK_03021	K07213	Organismal systems	9150	ATOX1, ATX1, copZ, golB; copper chaperone	18
NZ_CP022121.1_2691	JBMCGNCK_02756	K03671	Organismal systems, Human diseases	9150, 9160	trxA; thioredoxin 1	69
NZ_CP022121.1_1237	JBMCGNCK_01278	K03671	Organismal systems, Human diseases	9150, 9160	trxA; thioredoxin 1	60
NZ_CP022121.1_43	JBMCGNCK_00044	K03671	Organismal systems, Human diseases	9150, 9160	trxA; thioredoxin 1	30
NZ_CP022121.1_2703	JBMCGNCK_02769	K00773	Protein families: genetic information and processing	9182	tgt; queuine tRNA-ribosyltransferase [EC:2.4.2.29]	378
NZ_CP022121.1_2507	JBMCGNCK_02571	K00974	Protein families: genetic information and processing	9182	cca; tRNA nucleotidyltransferase (CCA-adding enzyme) [EC:2.7.7.72 3.1.3.- 3.1.4.-]	369

NZ_CP022121.1_211	JBMCGNCK_00213	K01926	Protein families: genetic information and processing	9182	rex; redox-sensing transcriptional repressor	138
NZ_CP022121.1_1110	JBMCGNCK_01151	K02355	Protein families: genetic information and processing	9182	fusA, GFM, EFG; elongation factor G	558
NZ_CP022121.1_2892	JBMCGNCK_02968	K02355	Protein families: genetic information and processing	9182	fusA, GFM, EFG; elongation factor G	426
NZ_CP022121.1_2636	JBMCGNCK_02701	K02356	Protein families: genetic information and processing	9182	efp; elongation factor P	138
NZ_CP022121.1_1942	JBMCGNCK_01999	K02357	Protein families: genetic information and processing	9182	tsf, TSFM; elongation factor Ts	200
NZ_CP022121.1_682	JBMCGNCK_00703	K02469	Protein families: genetic information and processing	9182	gyrA; DNA gyrase subunit A [EC:5.6.2.2]	561
NZ_CP022121.1_683	JBMCGNCK_00704	K02470	Protein families: genetic information and processing	9182	gyrB; DNA gyrase subunit B [EC:5.6.2.2]	465
NZ_CP022121.1_1008	JBMCGNCK_01036	K02499	Protein families: genetic information and processing	9182	yabN; tetrapyrrole methylase family protein / MazG family protein	89
NZ_CP022121.1_1137	JBMCGNCK_01178	K02518	Protein families: genetic information and processing	9182	infA; translation initiation factor IF-1	66
NZ_CP022121.1_2561	JBMCGNCK_02626	K02519	Protein families: genetic information and processing	9182	infB, MTIF2; translation initiation factor IF-2	384
NZ_CP022121.1_2732	JBMCGNCK_02799	K02520	Protein families: genetic information and processing	9182	infC, MTIF3; translation initiation factor IF-3	101
NZ_CP022121.1_2558	JBMCGNCK_02623	K02600	Protein families: genetic information and processing	9182	nusA; transcription termination/antitermination protein NusA	238
NZ_CP022121.1_1100	JBMCGNCK_01141	K02601	Protein families: genetic information and processing	9182	nusG; transcription termination/antitermination protein NusG	136
NZ_CP022121.1_58	JBMCGNCK_00059	K02687	Protein families: genetic information and processing	9182	prmA; ribosomal protein L11 methyltransferase [EC:2.1.1.-]	144
NZ_CP022121.1_2562	JBMCGNCK_02627	K02834	Protein families: genetic information and processing	9182	rbfA; ribosome-binding factor A	57
NZ_CP022121.1_896	JBMCGNCK_00923	K02835	Protein families: genetic information and processing	9182	prfA, MTRF1, MRF1; peptide chain release factor 1	261
NZ_CP022121.1_1944	JBMCGNCK_02001	K02838	Protein families: genetic information and processing	9182	frr, MRRF, RRF; ribosome recycling factor	132
NZ_CP022121.1_1933	JBMCGNCK_01990	K03168	Protein families: genetic information and processing	9182	topA; DNA topoisomerase I [EC:5.6.2.1]	424
NZ_CP022121.1_1094	JBMCGNCK_01130	K03218	Protein families: genetic information and processing	9182	rlmB; 23S rRNA (guanosine2251-2'-O)-methyltransferase [EC:2.1.1.185]	94
NZ_CP022121.1_2729	JBMCGNCK_02796	K03437	Protein families: genetic information and processing	9182	spoU; RNA methyltransferase, TrmH family	124
NZ_CP022121.1_695	JBMCGNCK_00716	K03497	Protein families: genetic information and processing	9182	parB, spo0J; ParB family transcriptional regulator, chromosome partitioning protein	123
NZ_CP022121.1_1009	JBMCGNCK_01037	K03530	Protein families: genetic information and processing	9182	hupB; DNA-binding protein HU-beta	64
NZ_CP022121.1_209	JBMCGNCK_00211	K03569	Protein families: genetic information and processing	9182	mreB; rod shape-determining protein MreB and related proteins	256
NZ_CP022121.1_927	JBMCGNCK_00954	K03569	Protein families: genetic information and processing	9182	mreB; rod shape-determining protein MreB and related proteins	253
NZ_CP022121.1_1203	JBMCGNCK_01243	K03595	Protein families: genetic information and processing	9182	era, ERAL1; GTPase	176
NZ_CP022121.1_204	JBMCGNCK_00206	K03609	Protein families: genetic information and processing	9182	minD; septum site-determining protein MinD	202
NZ_CP022121.1_1065	JBMCGNCK_01095	K03624	Protein families: genetic information and processing	9182	greA; transcription elongation factor GreA	106
NZ_CP022121.1_2469	JBMCGNCK_02533	K03631	Protein families: genetic information and processing	9182	recN; DNA repair protein RecN (Recombination protein N)	144
NZ_CP022121.1_1937	JBMCGNCK_01994	K03667	Protein families: genetic information and processing	9182	hslU; ATP-dependent HslUV protease ATP-binding subunit HslU	303
NZ_CP022121.1_59	JBMCGNCK_00060	K03686	Protein families: genetic information and processing	9182	dnaJ; molecular chaperone DnaJ	216
NZ_CP022121.1_61	JBMCGNCK_00062	K03687	Protein families: genetic information and processing	9182	GRPE; molecular chaperone GrpE	62
NZ_CP022121.1_1081	JBMCGNCK_01117	K03696	Protein families: genetic information and processing	9182	clpC; ATP-dependent Clp protease ATP-binding subunit ClpC	539
NZ_CP022121.1_3393	JBMCGNCK_03480	K03698	Protein families: genetic information and processing	9182	cbf, cbf1; 3'-5' exoribonuclease [EC:3.1.-.-]	166
NZ_CP022121.1_2599	JBMCGNCK_02664	K03706	Protein families: genetic information and processing	9182	codY; transcriptional pleiotropic repressor	164
NZ_CP022121.1_1024	JBMCGNCK_01052	K03768	Protein families: genetic information and processing	9182	PPIB, ppiB; peptidyl-prolyl cis-trans isomerase B (cyclophilin B) [EC:5.2.1.8]	151
NZ_CP022121.1_702	JBMCGNCK_00723	K03833	Protein families: genetic information and processing	9182	selB, EEFSEC; selenocysteine-specific elongation factor	321
NZ_CP022121.1_2546	JBMCGNCK_02610	K03977	Protein families: genetic information and processing	9182	engA, der; GTPase	301
NZ_CP022121.1_246	JBMCGNCK_00254	K04078	Protein families: genetic information and processing	9182	groES, HSPE1; chaperonin GroES	68
NZ_CP022121.1_1632	JBMCGNCK_01685	K04083	Protein families: genetic information and processing	9182	hslO; molecular chaperone Hsp33	208
NZ_CP022121.1_1934	JBMCGNCK_01991	K04094	Protein families: genetic information and processing	9182	rmFO, gid; methylenetetrahydrofolate--tRNA-(uracil-5-)-methyltransferase [EC:2.1.1.7	248
NZ_CP022121.1_424	JBMCGNCK_00434	K05516	Protein families: genetic information and processing	9182	cbpA; curved DNA-binding protein	145
NZ_CP022121.1_954	JBMCGNCK_00981	K05808	Protein families: genetic information and processing	9182	yhbH; putative sigma-54 modulation protein	112
NZ_CP022121.1_468	JBMCGNCK_00479	K06284	Protein families: genetic information and processing	9182	rB family transcriptional regulator, transcriptional pleiotropic regulator of transition st	64
NZ_CP022121.1_755	JBMCGNCK_00777	K06942	Protein families: genetic information and processing	9182	ychF; ribosome-binding ATPase	230
NZ_CP022121.1_2860	JBMCGNCK_02934	K07040	Protein families: genetic information and processing	9182	yceD, ylbN; DUF177 domain-containing protein	66
NZ_CP022121.1_2678	JBMCGNCK_02743	K07447	Protein families: genetic information and processing	9182	ruvX; putative pre-16S rRNA nuclease [EC:3.1.-.-]	45
NZ_CP022121.1_1006	JBMCGNCK_01034	K07533	Protein families: genetic information and processing	9182	prsA; foldase protein PrsA [EC:5.2.1.8]	24
NZ_CP022121.1_192	JBMCGNCK_00194	K07574	Protein families: genetic information and processing	9182	yhbY; RNA-binding protein	70
NZ_CP022121.1_1084	JBMCGNCK_01120	K07736	Protein families: genetic information and processing	9182	carD; CarD family transcriptional regulator	140
NZ_CP022121.1_197	JBMCGNCK_00199	K08301	Protein families: genetic information and processing	9182	rng, cafA; ribonuclease G [EC:3.1.26.-]	329
NZ_CP022121.1_999	JBMCGNCK_01027	K09685	Protein families: genetic information and processing	9182	purR; purine operon repressor	140
NZ_CP022121.1_57	JBMCGNCK_00058	K09761	Protein families: genetic information and processing	9182	rsmE; 16S rRNA (uracil1498-N3)-methyltransferase [EC:2.1.1.193]	115
NZ_CP022121.1_1653	JBMCGNCK_01706	K09825	Protein families: genetic information and processing	9182	perR; Fur family transcriptional regulator, peroxide stress response regulator	65
NZ_CP022121.1_2395	JBMCGNCK_02458	K13628	Protein families: genetic information and processing	9182	iscA; iron-sulfur cluster assembly protein	10
NZ_CP022121.1_775	JBMCGNCK_00797	K18979	Protein families: genetic information and processing	9182	queG; epoxyqueuosine reductase [EC:1.17.99.6]	82
NZ_CP022121.1_62	JBMCGNCK_00063	K22447	Protein families: genetic information and processing	9182	cct, ths; archaeal chaperonin	392
NZ_CP022121.1_1316	JBMCGNCK_01367	K00666	Protein families: metabolism	9181	K00666; fatty-acyl-CoA synthase [EC:6.2.1.-]	617
NZ_CP022121.1_2766	JBMCGNCK_02834	K00903	Protein families: metabolism	9181	epsB, capB; protein-tyrosine kinase [EC:2.7.10.3]	87
NZ_CP022121.1_1567	JBMCGNCK_01620	K01262	Protein families: metabolism	9181	pepP; Xaa-Pro aminopeptidase [EC:3.4.11.9]	158
NZ_CP022121.1_1135	JBMCGNCK_01176	K01265	Protein families: metabolism	9181	map; methionyl aminopeptidase [EC:3.4.11.18]	216
NZ_CP022121.1_1936	JBMCGNCK_01993	K01419	Protein families: metabolism	9181	hslV, clpQ; ATP-dependent HslUV protease, peptidase subunit HslV [EC:3.4.25.2]	132
NZ_CP022121.1_2639	JBMCGNCK_02704	K03568	Protein families: metabolism	9181	tldD; TldD protein	287
NZ_CP022121.1_2638	JBMCGNCK_02703	K03592	Protein families: metabolism	9181	pmbA; PmbA protein	239
NZ_CP022121.1_971	JBMCGNCK_00998	K03797	Protein families: metabolism	9181	E3.4.21.102, prc, ctpA; carboxyl-terminal processing protease [EC:3.4.21.102]	161
NZ_CP022121.1_1298	JBMCGNCK_01343	K03797	Protein families: metabolism	9181	E3.4.21.102, prc, ctpA; carboxyl-terminal processing protease [EC:3.4.21.102]	90
NZ_CP022121.1_1438	JBMCGNCK_01492	K03798	Protein families: metabolism	9181	ftsH, hflB; cell division protease FtsH [EC:3.4.24.-]	413
NZ_CP022121.1_1037	JBMCGNCK_01067	K03798	Protein families: metabolism	9181	ftsH, hflB; cell division protease FtsH [EC:3.4.24.-]	399
NZ_CP022121.1_13	JBMCGNCK_00014	K06894	Protein families: metabolism	9181	yfhM; alpha-2-macroglobulin	475
NZ_CP022121.1_725	JBMCGNCK_00747	K08602	Protein families: metabolism	9181	pepF, pepB; oligoendopeptidase F [EC:3.4.24.-]	376
NZ_CP022121.1_1886	JBMCGNCK_01943	K12132	Protein families: metabolism	9181	prkC, stkP; eukaryotic-like serine/threonine-protein kinase [EC:2.7.11.1]	245
NZ_CP022121.1_3391	JBMCGNCK_03478	K19224	Protein families: metabolism	9181	lytE, cwlF; peptidoglycan DL-endopeptidase LytE [EC:3.4.-.-]	19
NZ_CP022121.1_734	JBMCGNCK_00756	K19689	Protein families: metabolism	9181	ampS, pepS, ampT; aminopeptidase [EC:3.4.11.-]	294
NZ_CP022121.1_1885	JBMCGNCK_01942	K20074	Protein families: metabolism	9181	prpC, phpP; PPM family protein phosphatase [EC:3.1.3.16]	91
NZ_CP022121.1_3579	JBMCGNCK_03667	K01552	Protein families: signal and cellular processes	9183	ecfA; energy-coupling factor transport system ATP-binding protein [EC:7.-.-.-]	301
NZ_CP022121.1_756	JBMCGNCK_00778	K01552	Protein families: signal and cellular processes	9183	ecfA; energy-coupling factor transport system ATP-binding protein [EC:7.-.-.-]	168
NZ_CP022121.1_2906	JBMCGNCK_02982	K01604	Protein families: signal and cellular processes	9183	mmdA; methylmalonyl-CoA decarboxylase subunit alpha [EC:7.2.4.3]	501
NZ_CP022121.1_2423	JBMCGNCK_02486	K01989	Protein families: signal and cellular processes	9183	K01989; putative tryptophan/tyrosine transport system substrate-binding protein	173
NZ_CP022121.1_334	JBMCGNCK_00342	K02003	Protein families: signal and cellular processes	9183	ABC.CD.A; putative ABC transport system ATP-binding protein	104
NZ_CP022121.1_2100	JBMCGNCK_02164	K02016	Protein families: signal and cellular processes	9183	ABC.FEV.S; iron complex transport system substrate-binding protein	124
NZ_CP022121.1_1532	JBMCGNCK_01585	K02016	Protein families: signal and cellular processes	9183	ABC.FEV.S; iron complex transport system substrate-binding protein	74
NZ_CP022121.1_1273	JBMCGNCK_01316	K02028	Protein families: signal and cellular processes	9183	ABC.PA.A; polar amino acid transport system ATP-binding protein [EC:7.4.2.1]	131
NZ_CP022121.1_1209	JBMCGNCK_01249	K02051	Protein families: signal and cellular processes	9183	ABC.SN.S; NitT/TauT family transport system substrate-binding protein	141
NZ_CP022121.1_2770	JBMCGNCK_02838	K02238	Protein families: signal and cellular processes	9183	comEC; competence protein ComEC	78
NZ_CP022121.1_55	JBMCGNCK_00056	K02503	Protein families: signal and cellular processes	9183	HINT1, hinT, hit; histidine triad (HIT) family protein	61
NZ_CP022121.1_388	JBMCGNCK_00398	K03282	Protein families: signal and cellular processes	9183	mscL; large conductance mechanosensitive channel	91
NZ_CP022121.1_1792	JBMCGNCK_01846	K03324	Protein families: signal and cellular processes	9183	yjbB; phosphate:Na+ symporter	225
NZ_CP022121.1_102	JBMCGNCK_00103	K03427	Protein families: signal and cellular processes	9183	hsdM; type I restriction enzyme M protein [EC:2.1.1.72]	408
NZ_CP022121.1_2357	JBMCGNCK_02426	K03522	Protein families: signal and cellular processes	9183	fixB, etfA; electron transfer flavoprotein alpha subunit	207
NZ_CP022121.1_77	JBMCGNCK_00078	K03699	Protein families: signal and cellular processes	9183	tlyC; magnesium and cobalt exporter, CNNM family	225
NZ_CP022121.1_1154	JBMCGNCK_01195	K06990	Protein families: signal and cellular processes	9183	MEMO1; MEMO1 family protein	224
NZ_CP022121.1_562	JBMCGNCK_00576	K07316	Protein families: signal and cellular processes	9183	mod; adenine-specific DNA-methyltransferase [EC:2.1.1.72]	211
NZ_CP022121.1_3348	JBMCGNCK_03431	K07316	Protein families: signal and cellular processes	9183	mod; adenine-specific DNA-methyltransferase [EC:2.1.1.72]	208
NZ_CP022121.1_1218	JBMCGNCK_01258	K07473	Protein families: signal and cellular processes	9183	dinJ; DNA-damage-inducible protein J	42
NZ_CP022121.1_2960	JBMCGNCK_03040	K08369	Protein families: signal and cellular processes	9183	ydjE; MFS transporter, putative metabolite:H+ symporter	269
NZ_CP022121.1_493	JBMCGNCK_00505	K08369	Protein families: signal and cellular processes	9183	ydjE; MFS transporter, putative metabolite:H+ symporter	178
NZ_CP022121.1_870	JBMCGNCK_00897	K09013	Protein families: signal and cellular processes	9183	sufC; Fe-S cluster assembly ATP-binding protein	163
NZ_CP022121.1_80	JBMCGNCK_00081	K14392	Protein families: signal and cellular processes	9183	panF; sodium/pantothenate symporter	101
NZ_CP022121.1_406	JBMCGNCK_00416	K15987	Protein families: signal and cellular processes	9183	hppA; K(+)-stimulated pyrophosphate-energized sodium pump [EC:7.1.3.2]	792
NZ_CP022121.1_354	JBMCGNCK_00362	K15987	Protein families: signal and cellular processes	9183	hppA; K(+)-stimulated pyrophosphate-energized sodium pump [EC:7.1.3.2]	418
NZ_CP022121.1_2368	JBMCGNCK_02437	K19091	Protein families: signal and cellular processes	9183	cas6; CRISPR-associated endoribonuclease Cas6 [EC:3.1.-.-]	29
NZ_CP022121.1_3443	JBMCGNCK_03529	K19115	Protein families: signal and cellular processes	9183	csh2; CRISPR-associated protein Csh2	237
NZ_CP022121.1_2983	JBMCGNCK_03064	K19156	Protein families: signal and cellular processes	9183	prlF, sohA; antitoxin PrlF	12
NZ_CP022121.1_2767	JBMCGNCK_02835	K19420	Protein families: signal and cellular processes	9183	epsA, capA; protein tyrosine kinase modulator	48
NZ_CP022121.1_1247	JBMCGNCK_01288	K25034	Protein families: signal and cellular processes	9183	btuF; cobalamin transport system substrate-binding protein	114
NZ_CP022121.1_721	JBMCGNCK_00743	K00179	Unclassified		iorA; indolepyruvate ferredoxin oxidoreductase, alpha subunit [EC:1.2.7.8]	409
NZ_CP022121.1_722	JBMCGNCK_00744	K00180	Unclassified		iorB; indolepyruvate ferredoxin oxidoreductase, beta subunit [EC:1.2.7.8]	119
NZ_CP022121.1_2358	JBMCGNCK_02427	K00313	Unclassified		fixC; electron transfer flavoprotein-quinone oxidoreductase [EC:1.5.5.-]	248
NZ_CP022121.1_2935	JBMCGNCK_03015	K00573	Unclassified		E2.1.1.77, pcm; protein-L-isoaspartate(D-aspartate) O-methyltransferase [EC:2.1.1.77	81
NZ_CP022121.1_1486	JBMCGNCK_01540	K01023	Unclassified		assT; arylsulfate sulfotransferase [EC:2.8.2.22]	287
NZ_CP022121.1_1879	JBMCGNCK_01936	K01462	Unclassified		PDF, def; peptide deformylase [EC:3.5.1.88]	46
NZ_CP022121.1_2842	JBMCGNCK_02915	K01463	Unclassified		bshB1; N-acetylglucosamine malate deacetylase 1 [EC:3.5.1.-]	28
NZ_CP022121.1_3213	JBMCGNCK_03293	K01533	Unclassified		copB; P-type Cu2+ transporter [EC:7.2.2.9]	388
NZ_CP022121.1_1389	JBMCGNCK_01440	K01537	Unclassified		ATP2C; P-type Ca2+ transporter type 2C [EC:7.2.2.10]	653
NZ_CP022121.1_978	JBMCGNCK_01005	K01993	Unclassified		ABC-2.TX; HlyD family secretion protein	101
NZ_CP022121.1_1462	JBMCGNCK_01516	K03744	Unclassified		lemA; LemA protein	103
NZ_CP022121.1_3619	JBMCGNCK_03707	K03744	Unclassified		lemA; LemA protein	62
NZ_CP022121.1_3412	JBMCGNCK_03499	K03753	Unclassified		mobB; molybdopterin-guanine dinucleotide biosynthesis adapter protein	81
NZ_CP022121.1_90	JBMCGNCK_00091	K03969	Unclassified		pspA; phage shock protein A	139
NZ_CP022121.1_3246	JBMCGNCK_03327	K04750	Unclassified		phnB; PhnB protein	103
NZ_CP022121.1_2254	JBMCGNCK_02323	K05937	Unclassified		K05937; uncharacterized protein	52
NZ_CP022121.1_715	JBMCGNCK_00737	K06207	Unclassified		typA, bipA; GTP-binding protein	418
NZ_CP022121.1_1629	JBMCGNCK_01682	K06923	Unclassified		K06923; uncharacterized protein	162
NZ_CP022121.1_601	JBMCGNCK_00615	K06926	Unclassified		K06926; uncharacterized protein	43
NZ_CP022121.1_818	JBMCGNCK_00844	K06929	Unclassified		K06929; uncharacterized protein	47
NZ_CP022121.1_1047	JBMCGNCK_01077	K06950	Unclassified		K06950; uncharacterized protein	60
NZ_CP022121.1_1386	JBMCGNCK_01437	K06960	Unclassified		K06960; uncharacterized protein	42

NZ_CP022121.1_1882	JBMCGNCK_01939	K06973	Unclassified		K06973; uncharacterized protein	125
NZ_CP022121.1_3271	JBMCGNCK_03354	K07006	Unclassified		K07006; uncharacterized protein	34
NZ_CP022121.1_3546	JBMCGNCK_03634	K07006	Unclassified		K07006; uncharacterized protein	33
NZ_CP022121.1_634	JBMCGNCK_00648	K07006	Unclassified		K07006; uncharacterized protein	14
NZ_CP022121.1_2105	JBMCGNCK_02169	K07112	Unclassified		K07112; uncharacterized protein	244
NZ_CP022121.1_973	JBMCGNCK_01000	K07138	Unclassified		K07138; uncharacterized protein	154
NZ_CP022121.1_2701	JBMCGNCK_02767	K08992	Unclassified		lapA; lipopolysaccharide assembly protein A	13
NZ_CP022121.1_2760	JBMCGNCK_02828	K09117	Unclassified		K09117; uncharacterized protein	33
NZ_CP022121.1_868	JBMCGNCK_00895	K09118	Unclassified		K09118; uncharacterized protein	455
NZ_CP022121.1_1193	JBMCGNCK_01233	K09126	Unclassified		K09126; uncharacterized protein	145
NZ_CP022121.1_1737	JBMCGNCK_01790	K09157	Unclassified		K09157; uncharacterized protein	447
NZ_CP022121.1_219	JBMCGNCK_00221	K03545	Unclassified: genetic information processing	9192	tig; trigger factor	215
NZ_CP022121.1_1510	JBMCGNCK_01564	K03704	Unclassified: genetic information processing	9192	cspA; cold shock protein	71
NZ_CP022121.1_858	JBMCGNCK_00884	K03704	Unclassified: genetic information processing	9192	cspA; cold shock protein	67
NZ_CP022121.1_1742	JBMCGNCK_01795	K07402	Unclassified: genetic information processing	9192	xdhC; xanthine dehydrogenase accessory factor	36
NZ_CP022121.1_53	JBMCGNCK_00054	K07403	Unclassified: genetic information processing	9192	nfeD; membrane-bound serine protease (ClpP class)	184
NZ_CP022121.1_647	JBMCGNCK_00662	K07484	Unclassified: genetic information processing	9192	K07484; transposase	208
NZ_CP022121.1_3144	JBMCGNCK_03226	K07491	Unclassified: genetic information processing	9192	rayT; REP-associated tyrosine transposase	130
NZ_CP022121.1_2915	JBMCGNCK_02992	K07576	Unclassified: genetic information processing	9192	K07576; metallo-beta-lactamase family protein	238
NZ_CP022121.1_499	JBMCGNCK_00511	K09747	Unclassified: genetic information processing	9192	ebfC; nucleoid-associated protein EbfC	72
NZ_CP022121.1_1798	JBMCGNCK_01852	K09777	Unclassified: genetic information processing	9192	remA; extracellular matrix regulatory protein A	91
NZ_CP022121.1_1079	JBMCGNCK_01115	K19411	Unclassified: genetic information processing	9192	mcsA; protein arginine kinase activator	81
NZ_CP022121.1_684	JBMCGNCK_00705	K24648	Unclassified: genetic information processing	9192	remB; extracellular matrix regulatory protein B	27
NZ_CP022121.1_466	JBMCGNCK_00477	K03424	Unclassified: metabolism	9191	tatD; TatD DNase family protein [EC:3.1.21.-]	144
NZ_CP022121.1_2356	JBMCGNCK_02425	K03521	Unclassified: metabolism	9191	fixA, etfB; electron transfer flavoprotein beta subunit	147
NZ_CP022121.1_25	JBMCGNCK_00026	K04026	Unclassified: metabolism	9191	eutL; ethanolamine utilization protein EutL	195
NZ_CP022121.1_2362	JBMCGNCK_02431	K04069	Unclassified: metabolism	9191	pflA, pflC, pflE; pyruvate formate lyase activating enzyme [EC:1.97.1.4]	154
NZ_CP022121.1_3564	JBMCGNCK_03652	K04488	Unclassified: metabolism	9191	iscU, nifU; nitrogen fixation protein NifU and related proteins	87
NZ_CP022121.1_749	JBMCGNCK_00771	K04767	Unclassified: metabolism	9191	acuB; acetoin utilization protein AcuB	110
NZ_CP022121.1_3497	JBMCGNCK_03583	K05919	Unclassified: metabolism	9191	dfx; superoxide reductase [EC:1.15.1.2]	68
NZ_CP022121.1_2192	JBMCGNCK_02259	K06221	Unclassified: metabolism	9191	dkgA; 2,5-diketo-D-gluconate reductase A [EC:1.1.1.346]	244
NZ_CP022121.1_3184	JBMCGNCK_03264	K06911	Unclassified: metabolism	9191	PIR; quercetin 2,3-dioxygenase [EC:1.13.11.24]	179
NZ_CP022121.1_1728	JBMCGNCK_01781	K06997	Unclassified: metabolism	9191	yggS, PROSC; PLP dependent protein	119
NZ_CP022121.1_2874	JBMCGNCK_02949	K07030	Unclassified: metabolism	9191	fakA; fatty acid kinase [EC:2.7.2.18]	295
NZ_CP022121.1_1506	JBMCGNCK_01560	K07444	Unclassified: metabolism	9191	ypsC; putative N6-adenine-specific DNA methylase [EC:2.1.1.-]	250
NZ_CP022121.1_323	JBMCGNCK_00331	K09121	Unclassified: metabolism	9191	rC; pyridinium-3,5-bisthiocarboxylic acid mononucleotide nickel chelatase [EC:4.99.1.1	132
NZ_CP022121.1_1238	JBMCGNCK_01279	K10670	Unclassified: metabolism	9191	; glycine/sarcosine/betaine reductase complex component A [EC:1.21.4.2 1.21.4.3 1.21	21
NZ_CP022121.1_3147	JBMCGNCK_03229	K10671	Unclassified: metabolism	9191	grdE; glycine reductase complex component B subunit alpha and beta [EC:1.21.4.2]	355
NZ_CP022121.1_1243	JBMCGNCK_01284	K10671	Unclassified: metabolism	9191	grdE; glycine reductase complex component B subunit alpha and beta [EC:1.21.4.2]	348
NZ_CP022121.1_1244	JBMCGNCK_01285	K10672	Unclassified: metabolism	9191	grdB; glycine reductase complex component B subunit gamma [EC:1.21.4.2]	271
NZ_CP022121.1_3146	JBMCGNCK_03228	K10672	Unclassified: metabolism	9191	grdB; glycine reductase complex component B subunit gamma [EC:1.21.4.2]	213
NZ_CP022121.1_1245	JBMCGNCK_01286	K10672	Unclassified: metabolism	9191	grdB; glycine reductase complex component B subunit gamma [EC:1.21.4.2]	30
NZ_CP022121.1_3145	JBMCGNCK_03227	K10672	Unclassified: metabolism	9191	grdB; glycine reductase complex component B subunit gamma [EC:1.21.4.2]	22
NZ_CP022121.1_3540	JBMCGNCK_03628	K12137	Unclassified: metabolism	9191	hyfB; hydrogenase-4 component B [EC:1.-.-.-]	257
NZ_CP022121.1_2330	JBMCGNCK_02399	K12264	Unclassified: metabolism	9191	norV; anaerobic nitric oxide reductase flavorubredoxin	288
NZ_CP022121.1_2808	JBMCGNCK_02880	K12267	Unclassified: metabolism	9191	msrAB; peptide methionine sulfoxide reductase msrA/msrB [EC:1.8.4.11 1.8.4.12]	170
NZ_CP022121.1_1503	JBMCGNCK_01557	K17992	Unclassified: metabolism	9191	hndB; NADP-reducing hydrogenase subunit HndB [EC:1.12.1.3]	39
NZ_CP022121.1_1505	JBMCGNCK_01559	K18332	Unclassified: metabolism	9191	hndD; NADP-reducing hydrogenase subunit HndD [EC:1.12.1.3]	353
NZ_CP022121.1_839	JBMCGNCK_00865	K18446	Unclassified: metabolism	9191	ygiF; triphosphatase [EC:3.6.1.25]	67
NZ_CP022121.1_2740	JBMCGNCK_02807	K19784	Unclassified: metabolism	9191	chrR, NQR; chromate reductase, NAD(P)H dehydrogenase (quinone)	119
NZ_CP022121.1_1315	JBMCGNCK_01366	K19814	Unclassified: metabolism	9191	eam; glutamate 2,3-aminomutase [EC:5.4.3.9]	265
NZ_CP022121.1_3235	JBMCGNCK_03316	K20038	Unclassified: metabolism	9191	cutC; choline trimethylamine-lyase [EC:4.3.99.4]	402
NZ_CP022121.1_1241	JBMCGNCK_01282	K21576	Unclassified: metabolism	9191	e/sarcosine/betaine reductase complex component C subunit alpha [EC:1.21.4.2 1.21.	239
NZ_CP022121.1_1240	JBMCGNCK_01281	K21577	Unclassified: metabolism	9191	e/sarcosine/betaine reductase complex component C subunit beta [EC:1.21.4.2 1.21.4	261
NZ_CP022121.1_86	JBMCGNCK_00087	K21577	Unclassified: metabolism	9191	e/sarcosine/betaine reductase complex component C subunit beta [EC:1.21.4.2 1.21.4	182
NZ_CP022121.1_1498	JBMCGNCK_01552	K22341	Unclassified: metabolism	9191	fdhF2; formate dehydrogenase (NAD+, ferredoxin) subunit [EC:1.17.1.11]	177
NZ_CP022121.1_3613	JBMCGNCK_03701	K23077	Unclassified: metabolism	9191	dsrC; dissimilatory sulfite reductase related protein	75
NZ_CP022121.1_394	JBMCGNCK_00404	K23876	Unclassified: metabolism	9191	gdC, fldI, hadI, lcdC; (R)-2-hydroxyacyl-CoA dehydratese activating ATPase [EC:5.6.1.9	63
NZ_CP022121.1_1976	JBMCGNCK_02033	K23876	Unclassified: metabolism	9191	gdC, fldI, hadI, lcdC; (R)-2-hydroxyacyl-CoA dehydratese activating ATPase [EC:5.6.1.9	58
NZ_CP022121.1_259	JBMCGNCK_00267	K23997	Unclassified: metabolism	9191	dependent NAD(P)H-hydrate dehydratase / NAD(P)H-hydrate epimerase [EC:4.2.1.13	238
NZ_CP022121.1_2108	JBMCGNCK_02172	K23997	Unclassified: metabolism	9191	dependent NAD(P)H-hydrate dehydratase / NAD(P)H-hydrate epimerase [EC:4.2.1.13	146
NZ_CP022121.1_3558	JBMCGNCK_03646	K02005	Unclassified: signaling and cellular processes	9193	ABC.CD.TX; HlyD family secretion protein	108
NZ_CP022121.1_1289	JBMCGNCK_01334	K02039	Unclassified: signaling and cellular processes	9193	phoU; phosphate transport system protein	126
NZ_CP022121.1_937	JBMCGNCK_00964	K03296	Unclassified: signaling and cellular processes	9193	TC.HAE1; hydrophobic/amphiphilic exporter-1 (mainly G- bacteria), HAE1 family	207
NZ_CP022121.1_489	JBMCGNCK_00501	K03307	Unclassified: signaling and cellular processes	9193	TC.SSS; solute:Na+ symporter, SSS family	62
NZ_CP022121.1_700	JBMCGNCK_00721	K06298	Unclassified: signaling and cellular processes	9193	gerM; germination protein M	52
NZ_CP022121.1_3065	JBMCGNCK_03147	K06298	Unclassified: signaling and cellular processes	9193	gerM; germination protein M	20
NZ_CP022121.1_708	JBMCGNCK_00729	K06306	Unclassified: signaling and cellular processes	9193	yaaH; spore germination protein	128
NZ_CP022121.1_690	JBMCGNCK_00711	K06346	Unclassified: signaling and cellular processes	9193	jag; spoIIIJ-associated protein	85
NZ_CP022121.1_1000	JBMCGNCK_01028	K06412	Unclassified: signaling and cellular processes	9193	spoVG; stage V sporulation protein G	85
NZ_CP022121.1_1443	JBMCGNCK_01497	K07321	Unclassified: signaling and cellular processes	9193	cooC; CO dehydrogenase maturation factor	108
NZ_CP022121.1_1156	JBMCGNCK_01197	K09767	Unclassified: signaling and cellular processes	9193	yajQ; cyclic-di-GMP-binding protein	110
NZ_CP022121.1_3358	JBMCGNCK_03441	K01560	Xenobiotics biodegradation and metabolism	9111	E3.8.1.2; 2-haloacid dehalogenase [EC:3.8.1.2]	110
NZ_CP022121.1_805	JBMCGNCK_00826	K01821	Xenobiotics biodegradation and metabolism	9111	praC, xylH; 4-oxalocrotonate tautomerase [EC:5.3.2.6]	40
NZ_CP022121.1_1539	JBMCGNCK_01592	K05898	Xenobiotics biodegradation and metabolism	9111	kstD; 3-oxosteroid 1-dehydrogenase [EC:1.3.99.4]	199

Table S11. Similarity of electron bifurcation proteins identified from D. formicoaceticum proteome against proteins described in previous studies

S. ovata	*Sporomusa ovata*
S. fructosivorans JJ	*Solidesulfovibrio fructosivorans JJ*
O. alaskensis G20	*Oleidesulfovibrio alaskensis G20*

	Query Prokka ID	Query NCBI ID	Query Description	Subject	Subject Description	Coverage	Identity	E-value	Score
StnA	JBMCGNCK_01549	WP_089609810.1	NADH-quinone oxidoreductase subunit E	WP_021167189.1	StnA (*S. ovata*)	84.0%	41.3%	3.00E-39	119
StnB	JBMCGNCK_01550	WP_242965405.1	NAD(P)H-quinone oxidoreductase subunit E	WP_021167190.1	StnB (*S. ovata*)	94.0%	57.3%	0	583
StnC	JBMCGNCK_01551	WP_089609812.1	FAD-dependent oxidoreductase	WP_021167191.1	StnC (*S.ovata*)	88.0%	40.7%	0	833
HndA	JBMCGNCK_01556	WP_089609814.1	NuoE-like protein	EFL52768.1	NADH dehydrogenase (ubiquinone) 24 dKa subunit (*S. fructosivorans JJ*)	91%	40.4%	1.00E-45	134
HndB	JBMCGNCK_01557	WP_157677371.1	(2Fe-2S) ferredoxin domain-containing protein	EFL52769.1	Putative NAD-reducing hydrogenase subunit (*S. fructosivorans JJ*)	90.0%	36.1%	4.00E-23	73.9
HndC	JBMCGNCK_01558	WP_242965405.1	NADH-quinone oxidoreductase subunit NuoF	EFL52770.1	NADH dehydrogenase (quinone) (*S. fructosivorans JJ*)	75.0%	62.0%	0	567
HndD	JBMCGNCK_01559	WP_089609815.1	[FeFe] hydrogenase, group A	EFL52771.1	Hydrogenase, Fe-only (*S. fructosivorans JJ*)	100.0%	54.3%	0	610
FloxA	JBMCGNCK_01698	WP_157677380.1	Hydrogenase iron-sulfur subunit	ABB38011.2	methyl-viologen-reducing hydrogenase delta subunit (*O. alaskensis G20*)	100.0%	52.2%	1.00E-26	85.5
FloxB	JBMCGNCK_01699	WP_089609931.1	4Fe-4S binding protein	ABB38012.1	4Fe-4S ferredoxin iron-sulfur binding domain-containing protein (*O. alaskensis G20*)	91%	32.67%	1.00E-55	172
FloxC	JBMCGNCK_01700	WP_198306669.1	4Fe-4S dicluster domain-containing protein	ABB38013.1	Putative hydrogenase (*O. alaskensis G20*)	96%	33.73%	1.00E-65	201
FloxD	JBMCGNCK_01701	WP_089609932.1	FAD/NAD(P)-binding protein	ABB38014.1	Dihydroorotate dehydrogenase, electron transfer subunit (*O. alaskensis G20*)	97%	47.5	1.00E-99	283
HdrA	JBMCGNCK_01696	WP_089609930.1	CoB--CoM heterodisulfide reductase iron-sulfur subunit A family protein	ABB38010.1	4Fe-4S ferredoxin iron-sulfur binding domain-containing protein (*O. alaskensis G20*)	99%	53.80%	2.00E-176	499
HdrA	JBMCGNCK_01697	WP_089609930.1	CoB--CoM heterodisulfide reductase iron-sulfur subunit A family protein	ABB38010.1	4Fe-4S ferredoxin iron-sulfur binding domain-containing protein (*O. alaskensis G20*)	98%	60.20%	7.00E-80	240
HdrB	JBMCGNCK_01695	WP_089609929.1	CoB--CoM heterodisulfide reductase iron-sulfur subunit B family protein	ABB38009.1	CoB--CoM heterodisulfide reductase subunit B (O. alaskensis G20)	92%	42.91%	1.00E-86	251
HdrC	JBMCGNCK_01694	WP_198306668.1	4Fe-4S dicluster domain-containing protein	ABB38018.1	Heterodisulfide reductase C subunit (*O. alaskensis G20*)	91%	37.90%	2.00E-49	149

Figure S1. Distribution of genes encoding TCA cycle in the *Dehalobacteriia*

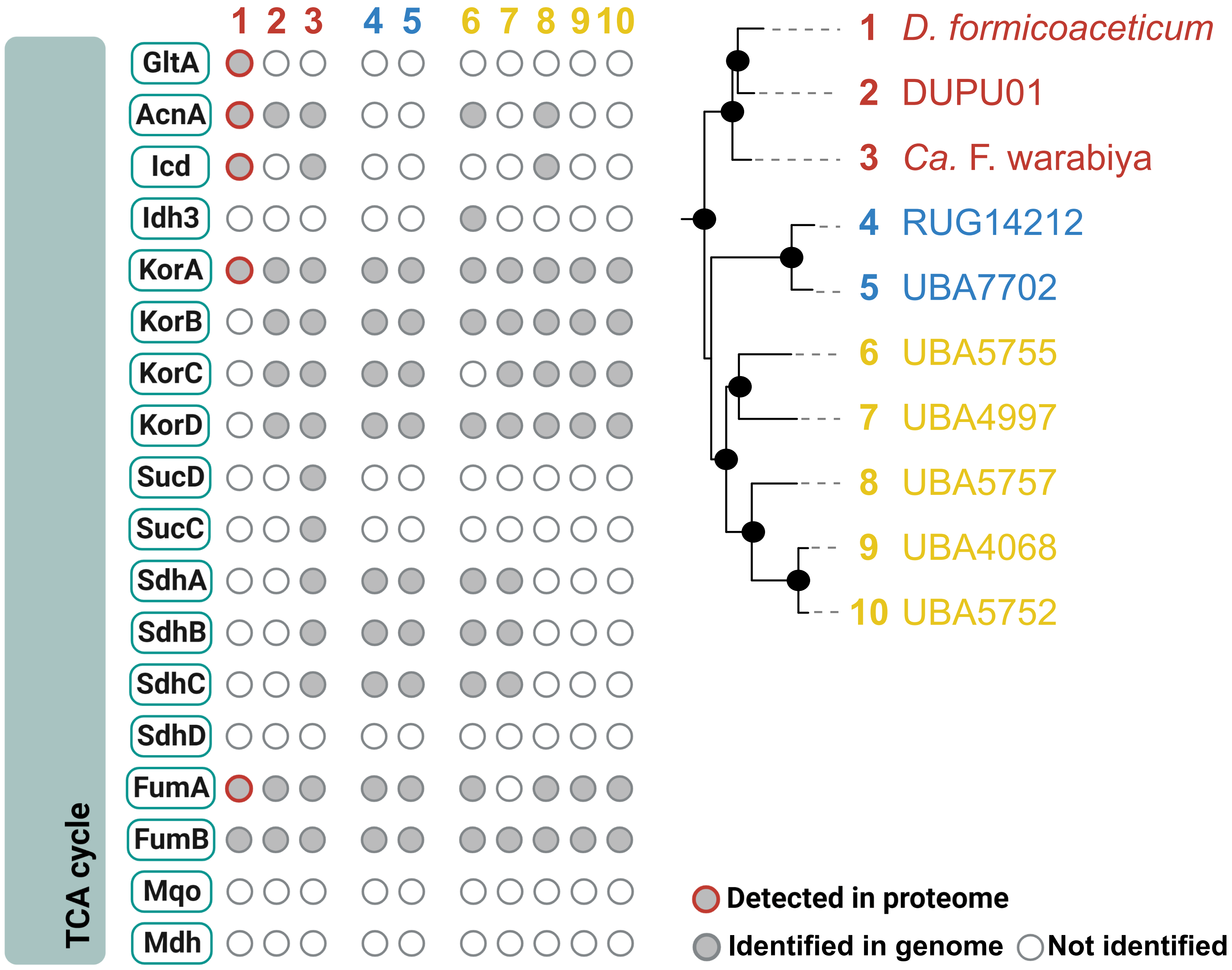

Supplement: Supplementary material 1 [file mgen-9-1039-s001.pdf]
